# Systems-level effects of ectopic galectin-7 reconstitution in cervical cancer and its microenvironment

**DOI:** 10.1186/s12885-016-2700-8

**Published:** 2016-08-24

**Authors:** Juan Carlos Higareda-Almaraz, Juan S. Ruiz-Moreno, Jana Klimentova, Daniela Barbieri, Raquel Salvador-Gallego, Regina Ly, Ilse A. Valtierra-Gutierrez, Christiane Dinsart, Gabriel A. Rabinovich, Jiri Stulik, Frank Rösl, Bladimiro Rincon-Orozco

**Affiliations:** 1 Division of Viral Transformation Mechanisms, German Cancer Research Center (DKFZ), Im Neuenheimer Feld 242, 69120 Heidelberg, Germany; 2 Institute for Diabetes and Cancer, Helmholtz Zentrum München, Deutsches Forschungszentrum für Gesundheit und Umwelt (GmbH), 85764 Neuherberg, Germany; 3 Department of Internal Medicine/Infectious Diseases and Respiratory Medicine, Charité, 10117 Berlin Germany; 4 Department of Molecular Pathology and Biology, Faculty of Military Health Sciences, University of Defense, 500 01 Hradec Králové, Czech Republic; 5 Unit of Microbiology, Department of Diagnostic Medicine and Prevention, S. Orsola-Malpighi University Hospital, Bologna, Italy; 6 Interfaculty Institute of Biochemistry, University of Tübingen, 72076 Tübingen, Germany; 7 Department Biologie II, Ludwig-Maximilians-Universität München, Planegg-Martinsried, Germany; 8 Division of Tumor Virology, German Cancer Research Center (DKFZ), 69,120 Heidelberg, Germany; 9 Laboratorio de Inmunopatología, Instituto de Biología y Medicina Experimental (IBYME), Consejo Nacional de Investigaciones Científicas y Técnicas, C1428 Ciudad de Buenos Aires, Argentina; 10 Microbiology School, Universidad Industrial de Santander, Carrera 27 con calle 9, 680,011 Bucaramanga, Colombia

**Keywords:** Galectin-7, Cervical cancer, Differential network analysis, Microenvironment crosstalk

## Abstract

**Background:**

Galectin-7 (Gal-7) is negatively regulated in cervical cancer, and appears to be a link between the apoptotic response triggered by cancer and the anti-tumoral activity of the immune system. Our understanding of how cervical cancer cells and their molecular networks adapt in response to the expression of Gal-7 remains limited.

**Methods:**

Meta-analysis of Gal-7 expression was conducted in three cervical cancer cohort studies and TCGA. *In silico* prediction and bisulfite sequencing were performed to inquire epigenetic alterations. To study the effect of Gal-7 on cervical cancer, we ectopically re-expressed it in the HeLa and SiHa cervical cancer cell lines, and analyzed their transcriptome and SILAC-based proteome. We also examined the tumor and microenvironment host cell transcriptomes after xenotransplantation into immunocompromised mice. Differences between samples were assessed with the Kruskall-Wallis, Dunn’s Multiple Comparison and T tests. Kaplan–Meier and log-rank tests were used to determine overall survival.

**Results:**

Gal-7 was constantly downregulated in our meta-analysis (*p* < 0.0001). Tumors with combined high Gal-7 and low galectin-1 expression (*p* = 0.0001) presented significantly better prognoses (*p* = 0.005). *In silico* and bisulfite sequencing assays showed *de novo* methylation in the Gal-7 promoter and first intron. Cells re-expressing Gal-7 showed a high apoptosis ratio (*p* < 0.05) and their xenografts displayed strong growth retardation (*p* < 0.001). Multiple gene modules and transcriptional regulators were modulated in response to Gal-7 reconstitution, both in cervical cancer cells and their microenvironments (FDR < 0.05 %). Most of these genes and modules were associated with tissue morphogenesis, metabolism, transport, chemokine activity, and immune response. These functional modules could exert the same effects in vitro and in vivo, even despite different compositions between HeLa and SiHa samples.

**Conclusions:**

Gal-7 re-expression affects the regulation of molecular networks in cervical cancer that are involved in diverse cancer hallmarks, such as metabolism, growth control, invasion and evasion of apoptosis. The effect of Gal-7 extends to the microenvironment, where networks involved in its configuration and in immune surveillance are particularly affected.

**Electronic supplementary material:**

The online version of this article (doi:10.1186/s12885-016-2700-8) contains supplementary material, which is available to authorized users.

## Background

Galectins are a family of carbohydrate-binding proteins involved in immune response, angiogenesis and cancer development [1]. Different members of the galectin family, such as galectin-1 (Gal-1) and galectin-7 (Gal-7), have been found to influence the pathogenesis of cervical cancer (CaCx). The expression of Gal-1 is functionally linked to histopathological grading in cervical cancer patients, namely by affecting the rate of proliferation, lymph node metastasis and tumor invasion [2]. Gal-1 is also the receptor for *Trichomonas vaginalis* [3], a sexually transmitted protozoan parasite and risk factor for cervical cancer [4]. In contrast, Gal-7 is preferentially expressed in stratified squamous epithelia from skin, genital and upper digestive track [5]. Gal-7 is a p53-inducible gene, which is upregulated in response to UVB radiation in normal human keratinocytes [6].

As an endogenous lectin, a fraction of Gal-7 is constitutively localized at the mitochondria. It has been found to interact with the anti-apoptotic protein Bcl-2, suggesting its regulatory role in apoptotic processes [7]. Importantly, increased Gal-7 expression has been shown as a positive predictive biomarker for clinical responses after adjuvant radiation therapy in cervical cancer patients [8]. While a plethora of distinct properties of Gal-7 are known, an integrative analysis of the molecular mechanisms with which Gal-7 expression shapes the tumorigenic process has not yet been performed.

**Fig. 1:**
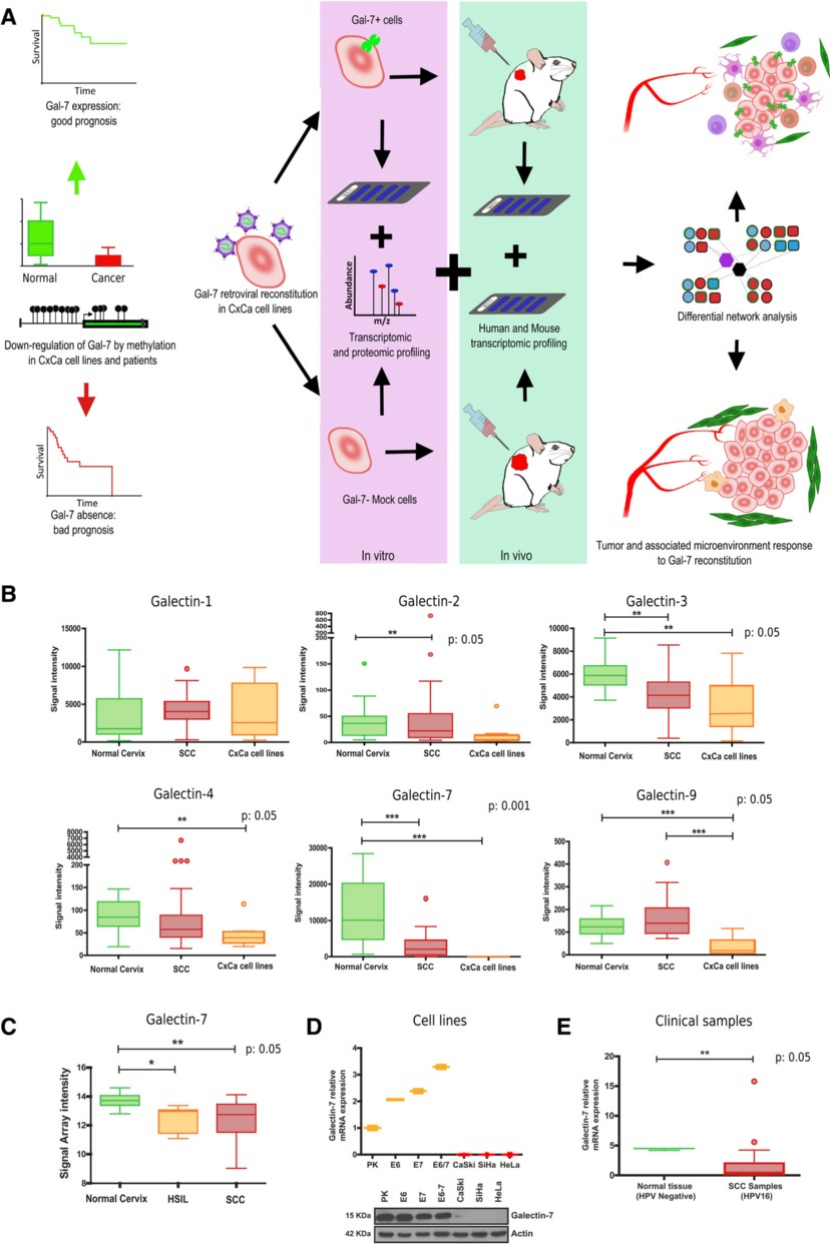
Study design and galectin expression in cervical cancer. **a** Pipeline of the complete experimental approach.** b** Gene expression profiles of galectin family members in clinical samples (normal cervix and squamous cell carcinoma, SCC) and CaCx cell lines obtained from the Scotto cohort. **c** Gal-7 transcription in normal cervical tissue, high grade squamous intraepithelial lesions (HSIL) and squamous cell carcinomas (SCC) from the Zhai cohort. **d** qPCR and Western blot analysis of Gal-7 in primary keratinocytes (PK), HPV 16 E6, E7 and E6/E7 immortalized human keratinocytes, and CaCx cells (CaSki, SiHa, HeLa). **e** Analysis of Gal-7 expression in clinical samples (derived from HPV16 positive SCCs) and normal tissue. (^B^Kruskall-Wallis Test, *P* < 0.0001, Dunn’s Multiple Comparison Test *P* < 0.05; ^E^T Test, *P* < 0.05, *** means highly significant, ** moderately significant)

In the present study, we performed an integrative analysis of the impact of Gal-7 reconstitution in cervical cancer cells and their microenvironment at the systems level *in silico,* in vitro*,* and in a mouse model. For that purpose, we conducted a meta-analysis of a whole spectrum of clinical data in which cervical cancer patients showed a significant longer life span when tumors had simultaneous high Gal-7 and low Gal-1 expression. To validate these observations in the biological system, we ectopically expressed Gal-7 in CaCx cell lines and evaluated them through transcriptomics, SILAC-based proteomics, gene methylation profiling, and network analysis. We identified numerous circuits implicated in cancer hallmarks that were affected by Gal-7 re-expression (Fig. 1a). These results suggest a bi-directional regulation between the tumor and its microenvironment where Gal-7 could be a critical mediator.

## Methods

### Galectin expression profiles in independent cohorts of cervical cancer

To analyze the expression profiles of the galectin genes in cervical epithelium, we used the data from the differential expression profiles established by Scotto et al. [9] (Gene Expression Omnibus NCBI, GEO accession number GSE9750). The cohort consists of three groups of 24 normal cervical epithelium samples, a panel of nine CaCx cell lines and 28 squamous cervical cancer samples (SCC). Clinical samples represent the different cancer stages as suggested by the International Federation of Gynecology and Obstetrics (FIGO) [10]. The differential expression analyses were held using the reported signal intensity information for each galectin gene. Comparisons among groups were performed using a Kruskall-Wallis test followed by a post-hoc Dunn’s multiple comparison test. To test the Gal-7 profile through the CaCx progression, we used the cohort analysis established by Zhai et al. [11], (GEO accession number GDS3292) composed of 10 normal cervix, 7 high-grade squamous intraepithelial lesion (HSIL) samples and 21 SCCs. The Zhai cohort was used to study the modulation of Gal-7 along the process of transformation towards malignancy.

### Hierarchical clustering and survival analysis

To study the expression profiles of Gal-7 and Gal-1 genes, the data compiled in a Provisional cohort (November 2014) for Cervical Squamous Cell Carcinoma and Endocervical Adenocarcinoma from The Cancer Genome Atlas (CESC-TCGA) [12] were used. This consisted of 185 RNA-Seq samples from patients in different tumor stage and grading. RNA-Seq Expectation-Maximization (RSEM) [13] data normalized by TCGA were used directly. The comparisons among groups were performed using a Kruskall-Wallis test followed by a post-hoc Dunn’s multiple comparison tests.

Unsupervised Hierarchical clustering was performed on a centered Pearson correlation coefficient algorithm and a complete linkage method to compare the expression patterns of the Gal-7 and Gal-1 genes across the CESC-TCGA data set. Overall survival (OS) was defined from the day of the sample intake to the patient’s death. Data of the patients who had survived until the end of the observation period were censored at their last follow-up visit. The OS curve was plotted using the clusters obtained from the hierarchical clustering analysis, using the Kaplan-Meier method. A log-rank test was used to compare the survival curves. All the statistical analysis and graphics were performed using R environment, version 3.0.1 (2013-05-16) “Good Sport”.

### CESC-TCGA data methylation analysis

The relationship between Gal-7 methylation and mRNA expression was analyzed using the CESC-TCGA Methylation data versus mRNA expression. To confirm the correlations, the Spearman’s rank correlation coefficient was used, with a two-tailed *P*-value and an alpha = 0.05.

### Analysis of methylation

Bisulfite-PCR-pyrosequencing focused on 13 CpG sites between nt −294 and nt +132 of the Gal-7 gene (the target region encompasses the promoter, the first exon and the first intron of the gene; the Eukaryotic Promoter Database ID: LGALS7 (chr19:39,262,157–39,266,157) was employed. PCR primers were designed on the *in silico*-converted target sequence, with the correspondent sequencing primers, to generate two products (Additional file 1: Table S2). Total DNA (500 ng) extracted from cells underwent bisulfite conversion by using the EZ DNA Methylation^TM^ Kit (Zymo reasearch), following manufacturer’s instruction. Two PCR reactions were performed in a final volume of 50 μl with 200 mM dNTPs mix, 500 nM each primer, 2.5 mM MgCl_2_, 0.62 U Hot Start GoTaq® DNA polymerase (Promega) and 5 μl of bisulfite-converted DNA, under the following conditions: 95 °C for 2 min, 50 cycles of 95 °C for 30 s, 56 °C for 30 s, 72 °C for 30 s and final extension at 72 °C for 10 min. For the sequencing reactions of the PCR products, the PyroMark™ Q24 System (Qiagen) was used following manufacturer’s instructions (400 nM of one sequencing primer/reaction). The final pyrograms were analyzed using the allele quantification (AQ) mode in the PyroMark™ Q24 Software, to determine the proportion of C/T (forward sequencing) and, hence, the methylation frequency of the target CpG sites.

### Cell culture, azacytidine treatment

HPV16 immortalized human keratinocytes, HeLa, SiHa and CaSki Gal-7 positive and control cells as well as 293 T cells were maintained under standard conditions in Dulbecco’s modified Eagle medium supplemented with 10 % Fetal Calf Serum and 1 % penicillin/streptomycin (P/S). Azacytidine (5′-Aza; Cayman) was dissolved in DMSO (Merck). Treatment was done for four days using 1 (HeLa) to 10 (SiHa and CaSki) μmol/L.

### Gal-7 cloning and retrovirus construction

The Human Gal-7 gene cloned in the pEF1 vector [14] was amplified by PCR using primers containing the restriction sites XhoI and BamHI: Gal-7-XhoI-F: 5′-GAGCTCGAGCCGCCATGTCCAACGTCCCCCACAA-3′ and Gal-7-BamHI-R: 5′-GCGCGGATCCTCAGAAGATCCTCACG-3′ under the following parameters: denaturation at 98 °C for 3 min, followed by 30 cycles at 94 °C 30 s, 58 °C 45 s, 72 °C 1 min and a final extension at 72 °C for 10 min. Subsequently, 2 μg of Gal-7 insert DNA was digested with XhoI and BamHI enzymes (Fermentas, Sankt Leon-Rot) for 1 h at 37 °C and purified using the QIAquick® PCR Purification Kit (Qiagen, Hilden). Afterwards, the digested product was ligated into pLXSN viral vector previously digested with XhoI and BamHI and dephosphorylated. Subcloning was further validated by sequencing of the resulting pLXSN-Gal-7 construct (GATC Biotech, Germany). For production of viral particles, 293 T cells were transfected with the construct pLXSN-Gal7 together with the corresponding retroviral packaging vectors pVSV.G [15] and pSVΨ [16]. Viral particles containing empty pLXSN vector were also produced as control. 293 T cell supernatant was harvested and filtered through a 0.45 μm filter (Minisart Plus, Sigma-Aldrich). Filtered supernatant was mixed with 5 μg/ml of the cationic polymer Polybrene® and used for infection of CaSki, HeLa and SiHa cells. After infection, the cells were subjected to antibiotic selection with G418 for two weeks (Life Technologies). Gal-7 expression was finally analyzed by Western blot.

### Protein extraction and Western blotting

Gal-7+ cervical tumor cell lines and the respective control cells were collected, washed in 1 × PBS and resuspended in RIPA buffer (20 mM Tris pH 7.5; 150 mM NaCl; 1 mM Na_2_EDTA; 1 mM EGTA; 1 % NP-40; 1 % sodium deoxycholate) containing 1× complete protease inhibitor cocktail (Roche Diagnostics, Mannheim). Protein extraction was performed by incubating for 30 min on ice and subsequently centrifuging for 30 min at 4 °C and 13,000 rpm. Supernatants were quantified using the Bio-Rad Protein Assay Dye Reagent Concentrate (Bio-Rad, Munich). 80 μg protein/lane was used for Western blotting. After transfer, the PDVF filters were incubated with the following antibodies: anti-human Galectin-7, epr4287 (Genetex, USA), anti-Tubulin (G8), sc-55,529 (Santa Cruz Biotechnology, USA). Blots were developed using the Western lightning plus-ECL system (Perkin Elmer, Rodgau).

### Colony formation assay and mitochondrial membrane potential measurements

The colony formation assay was essentially performed as described by Rotem et al. [17]. HeLa cells were cultured at 500, CaSki and SiHa at 1×10^3^ cells in 6-well plates. Cells were cultured for 7 days or until overlapping colonies started to appear. Cells were then briefly washed with DPBS and fixed for 30 min with a 10 % glutaraldehyde solution. Colonies were stained with 6 % crystal violet for 30 min. Each well in the plate was photographed with a Stereomicroscope (Olympus, Hamburg) and the colonies were automatically counted using the image processor software ImageJ.

To measure mitochondrial membrane potential, 15,000 cells from each cell line were plated in 100 μl normal DMEM in 96-well μClear black plates. The next day, the cells were treated with different concentrations of HA14-1 (10 μM, 25 μM and 40 μM) for 24 h. Afterwards, 100 μL/well of 30nM JC-1 Solution (Life Technologies, Darmstadt) were added and the cells were incubated for 30 min at 37 °C in the dark. After washing the plates twice with 1× Dilution Buffer solution (PBS + 2 % FCS) fluorescence measurement was performed by reading the plate in a Synergy 2 Multi-Mode microplate reader with the following settings: excitation: 475 nm, emission at 530 (monomers emission) and 590 nm (aggregate emission). A decrease in the ratio between the fluorescence of monomers and aggregates indicates mitochondrial depolarization and cell death.

### Animal tumor model

7–8 week-old female nude Balb/c mice (Janvier Labs, St Berthevin, France) were maintained under pathogen-free conditions. 5×10^5^ (HeLa and HeLa Gal-7) or 5×10^6^ (SiHa and SiHa Gal-7) cells were suspended in 100 μl or 200 μl ice-cold PBS and subcutaneously implanted into the flanks of mice (5 mice/group). Tumor sizes were measured with an electronic digital caliper (Farnell, Germany) three times a week and the tumor volume was calculated according to the formula: V = 1/2 × length × width^2^ (mm^3^). Animals were killed according to the animal welfare act when a tumor volume reached a volume of 1500 mm^3^. The animals at the German Cancer Research Center were maintained in compliance with German and European statutes and all animal experiments were undertaken with the approval of the responsible Animal Ethics Committee.

### RNA extraction, reverse transcription and quantitative PCR analysis

RNA was extracted from cells using the RNeasy Mini Kit (Qiagen) according to the manufacturer’s instructions. One μg of RNA was reverse transcribed using RevertAid Reverse Transcriptase (Thermo Scientific, USA) and dT22primers according to the manufacturer’s protocol. The resulting cDNA was used for quantitative PCR analyses using the CFX96 Touch™ Real-Time PCR Detection System (BioRad, USA), the iTaq Universal SYBR Green (BioRad, USA) and the primers described in Additional file 1: Table S3.

### Gene expression profiling analysis

RNA was extracted from tissue culture cells using the RNeasy Mini Kit (Qiagen) according to the manufacturer’s instructions. A total of 500 ng of RNA from every sample was labeled and hybridized in the HumanHT-12v4 expression BeadChip (Illumina) following standard procedures of the Genomic Core facility at the DKFZ. For the tumors, fresh-frozen xenotransplants isolated from nude mice were homogenized in a Precellys® 24 tissue homogenizer (Bertin Tech. USA). Subsequently, RNA from tumors was isolated using standard TRIZOL procedure (Invitrogen) and Direct-zol RNA (Zymo Research) following the manufacturer’s instructions. Samples within the groups were pooled. A total of 500 ng of RNA from every pooled group was labeled and hybridized in the HumanHT-12v4 expression BeadChip (for human genes) or the mouseWG-6v2 expression BeadChip (for mouse genes) following standard procedures of the Genomic Core facility at the DKFZ. Bioinformatic data analysis was performed using Chipster [18] software version 3.1. mRNAs with a fold change of at least 2 between Gal-7 negative and Gal-7 positive cells or tumors were considered significant and were used for further analysis.

### Cell lysis and protein digestion for mass spectrometry analysis

Cell pellets were resuspended in 1 % (w/v) sodium deoxycholate (SDC) and 50 mM ammonium bicarbonate, boiled at 99 °C for 5 min and cooled to 4 °C. The lysates were then treated with benzonase (Sigma-Aldrich, Germany) in a final concentration of 150 U/mL for 60 min on ice. Insoluble material was removed by centrifugation at 14,000 G, 15 min, 4 °C. Protein concentration was determined by bicinchoninic acid protein assay kit (Sigma-Aldrich, Germany). Corresponding light and heavy lysates were mixed in 1:1 protein ratio. Lysates were then reduced with 10 mM dithiothreitol at 37 °C for 60 min, alkylated with 20 mM iodoacetamide at RT for 30 min in the dark and the unreacted iodoacetamide was quenched with further 10 mM dithiothreitol at RT for 15 min. The samples were diluted with 50 mM ammonium bicarbonate to decrease the concentration of SDC to 0.5 % and digested with sequencing grade trypsin (Promega, USA) overnight at 37 C. SDC was removed by the modified phase transfer protocol [19]. Briefly, ethyl-acetate was added and the digested product was acidified by trifluoroacetic acid (TFA) to a final concentration of ca. 2 % (v/v). The mixtures were vortexed vigorously for 1 min, centrifuged at 14,000 G for 5 min and the upper organic layer was removed. The extraction was repeated with fresh portion of ethyl-acetate. The aqueous phases were desalted on Empore™ extraction cartridges (Sigma-Aldrich, Germany) and dried in vacuum.

### SILAC labeling

HeLa and SiHa cells as well as their Gal-7 reconstituted counterparts were SILAC labeled by cultivating the cells for 5 passages in DMEM media (without glutamine, arginine and lysine; Silantes 282,986,444) containing 10 % (v/v) dialyzed FBS. Isotopically labeled L-lysine [^13^C_6_^15^N_2_ - labeled] 0.798 mM and L-arginine [^13^C_6_^15^N_4_ - labeled] 0.398 mM (Silantes, Germany) were added to the DMEM media (Fig. 4b). Unlabeled L-proline was added to a final concentration of 2.61 mM (300 mg/L) to prevent arginine-proline conversion [20].

### Peptide separation and mass spectrometry analysis

Peptides were separated by two-dimensional liquid chromatography (LC). In the first dimension, reversed phase LC under high-pH mobile phase conditions was performed using Alliance 2695 LC system (Waters, UK). The mobile phases were (A) water, (B) acetonitrile (ACN) and (C) 200 mM ammonium formate pH 10. Dried peptide mixtures were dissolved in 25 % C and 4 % ACN, and an aliquot of 200 μg was loaded on trap column (Gemini C18, 2 × 4 mm) and column (Gemini C18, 3 μm, 110 Å, 2 × 150 mm; Phenomenex, USA). Peptide separation was performed by linear gradient from 5 to 55 % of B in 62 min with constant 10 % of C. Flow rate was 0.16 mL/min, the column was kept at 40 °C and the separation was monitored at 215 nm. Fractions were collected manually in 2-min intervals over the sample elution window from 10th to 52nd min, acidified with TFA and dried in vacuum. The last 2 fractions were combined with the first 2 fractions in sequential order. Second dimension of the separation was performed on an Ultimate 3000 RSLCnano system (Dionex, USA) coupled on-line through Nanospray Flex ion source with Q-Exactive mass spectrometer (Thermo Scientific, Germany). Fractions were dissolved in 2 % ACN/0.05 % TFA and loaded on capillary trap column (C18 PepMap100, 3 μm, 100 Å, 0.075 × 20 mm; Dionex) by 5 μL/min of 2 % ACN/0.05 % TFA for 5 min. Then they were separated on capillary column (C18 PepMap RSLC, 2 μm, 100 Å, 0.075 × 150 mm; Dionex) by step linear gradient of mobile phase B (80 % ACN/0.1 % FA) over mobile phase A (0.1 % FA) from 4 to 34 % B in 48 min and from 34 to 55 % B in 10 min at flow rate of 300 nL/min. The column was kept at 40 °C and the eluent was monitored at 215 nm. Spraying voltage was 1.75 kV and heated capillary temperature was 275 °C. The mass spectrometer operated in the positive ion mode performing survey MS (at 350–1650 m/z) and data-dependent MS/MS scans on 10 most intense precursors with dynamic exclusion window of 30 s. MS scans were acquired with the resolution of 70,000 from 10^6^ accumulated charges; maximum fill time was 100 ms. The intensity threshold for triggering MS/MS was set at 5×10^4^ for ions with z ≥ 2 and the isolation window was 1.6 Da. Normalized collision energy for HCD fragmentation was 27 units. MS/MS spectra were acquired with the resolution of 17,500 from 10^5^ accumulated charges; maximum fill time was 100 ms.

### Protein identification and quantification

Database search and quantification were performed by Proteome Discoverer v.1.4 software (Thermo Scientific). The reference proteome set of *Homo sapiens* containing canonic and isoform sequences was downloaded from UniProt [21] (http://www.uniprot.org/) on Aug 18th 2014 and merged with the common contaminants file downloaded from the MaxQuant web page (http://www.coxdocs.org/doku.php?id=maxquant:common:download_and_installation); the merged database contained 89,252 sequences. The search parameters were as follows: digestion with trypsin, max. 2 missed cleavages, allowed peptide mass tolerance of 10 ppm, fragment mass tolerance of 0.02 Da, fixed carbamidomethylation of cysteine, variable modifications: oxidation of methionine, acetylation of protein N-term and SILAC labels Arg10 a Lys8. The strict target value of FDR for a decoy database search of 0.01 was applied (high confidence). Only unique peptides were considered for quantification and the heavy to light ratios were normalized on protein median for each replicate (Additional file 2: Supplementary File 1).

For relative protein quantification only protein groups with a minimum of 2 identified peptides in all three replicates and a minimum of 1 quantified peptide per each replicate were considered. Log2 values of protein ratios from each replicate were then subjected to the ranking test to find the most significantly regulated proteins [22] taking the protein groups found in top (T) or bottom (B) groups in all three replicates (TTT or BBB). The false discovery rate (FDR) was evaluated by non-parametric estimate as an average number of proteins in the “false” groups (TTB, TBT, BTT, BBT, BTB and TBB). The significance cut-off of the FDR was set around 5 %. For analysis, only proteins with a log2 fold change value of 1.2 or higher were considered (Additional file 3: Supplementary File 2). The mass spectrometry proteomics data have been deposited to the ProteomeXchange Consortium [23] via the PRIDE partner repository with the dataset identifier PXD001806.

### Pathway and GO enrichment analysis

We performed an enrichment analysis of pathway-based sets of proteins considering all the nodes of our extended network. Enrichment was done employing ConsensusPathDB, of the Max Planck Institute for Molecular Genetics, by using the overrepresentation analysis online tool. As input, we uploaded the UniProt protein identifiers of all the elements of the extended network. We searched against pathways as defined by Reactome [24] and KEGG [25], with a minimal overlap with the input list of 5 and a *p*-value cutoff of 0.001.

Also, employing the same website and the same analysis tool, we performed an enrichment analysis based on Gene Ontology (GO) [26] level 3 category of biological processes. For this analysis, we considered only the identified core proteins and set the *p*-value cutoff on 0.001.

### Network construction

Network reconstruction was performed with the aid of the Cytoscape Plugin, BisoGenet [27], using the identified proteins as bait nodes and adding edges with the following parameters: Organism > Homo sapiens, protein identifiers only; Data Settings > protein-protein interactions; all data sources and all experimental methods; method > By adding edges connecting input nodes and as Output > Proteins.

The iRegulon plugin was used to predict their transcriptional regulators using the default setting. Only predicted transcriptional regulators with normalized enrichment scores (NES) >3 were used.

## Results

### Gal-7 is downregulated in squamous cervical cancer, high-grade squamous intraepithelial lesions and cervical cancer cell lines

To get an insight into the galectin status of high-risk human-papillomavirus (HPV)-induced tumors, we first examined the expression of nine different members of this gene family on microarrays from the Scotto cohort [9] (Additional file 4: Figure S1). A significant down-regulation of Gal-7 expression was observed in fresh samples derived from squamous cell carcinoma (SCC), as well as established HPV16/18-positive CaCx cell lines (Fig. 1b). Considering the signal intensity of the microarrays, Gal-2 and Gal-3 transcription was also affected to some extent in SCC/CaCx samples compared to normal cervical tissue (Fig. 1b). However, in contrast to Gal-7, these two galectins are not directly related to tumorigenesis [28]. Only Gal-7 negative regulation showed a high statistical significance (Fig. 1b). This could be further supported by the Zhai cohort [11] (Additional file 4: Figure S1. B), where Gal-7 expression was also reduced in high-grade squamous intraepithelial lesions (HSIL) and in cervical cancer samples (Fig. 1c). Hence, the gradual downregulation of Gal-7 in premalignant lesions and a marked reduction in SCCs suggest that it might play a role in the development of cervical cancer. We validated our observations from the meta-analysis by qPCR and Western blot (Fig. 1d). For this purpose, we also included human keratinocytes that were separately immortalized by the E6-, E7- and E6/E7 oncoproteins of HPV 16 [29]. While CaSki, SiHa, and HeLa cells showed almost a complete suppression of their mRNA steady-state levels, Gal-7 was increased in immortalized cells when compared with primary keratinocytes (Fig. 1d). However, no significant differences among the same cells could be discerned at the protein level (Fig. 1d, below). Moreover, consistent with the microarray data (Fig. 1b), clinical specimens obtained from SCC patients also demonstrated a significant decrease (*p* < 0.05) of the Gal-7 mRNA in biopsies when compared to normal control samples (Fig. 1e). Altogether, these data imply that Gal-7 downregulation correlates with cervical cancer progression.

### Mutually exclusive expression of Gal-7 and Gal-1 determines clinical outcome and overall survival

**Fig. 2:**
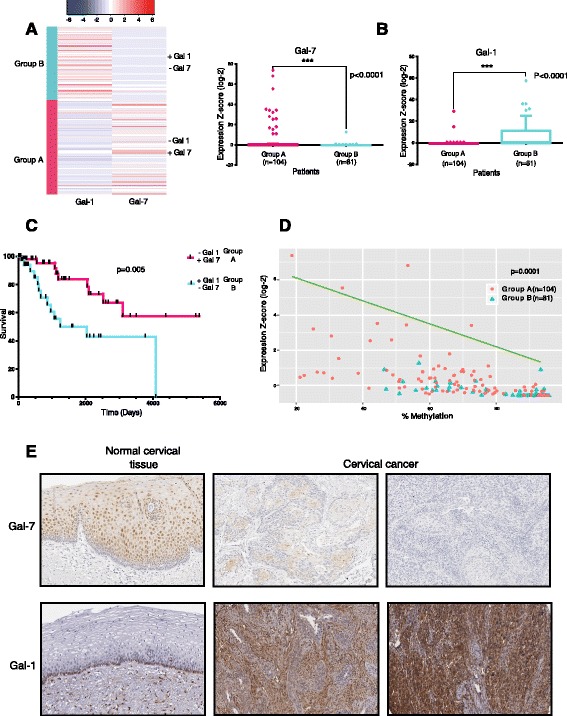
Analysis of Gal-1/-7 expression and survival in the TCGA-CESC cohort. **a** Hierarchical clustering of Gal-7 and Gal-1 expression in a 185-patient panel of CESC from the TCGA. **b** Gal-7 and Gal-1 expression in TCGA-CESC patients. **c** Kaplan-Meier curve for 5000 days of overall survival in the CESC panel of TCGA. Censored events were marked with vertical black lines. **d** Inverse correlation of Gal-7 expression and methylation. **e** Immunohistological staining of Gal-1 and Gal-7 in normal and cervical cancer tissue sections (immunohistochemistry images were taken from the Human Protein Atlas Project). Gal-1 was detected using the HPA000646 antibody. Gal-7 was detected using the HPA001549 antibody. (^B^Mann Whitney test *P* < 0.0001 Gaussian Approximation two-tailed *P*-value; *** means highly significant. ^C^log-rank test; *p* = 0.005. ^D^ *P* < 0.0001, Spearman r, two tailed *P* value, alpha = 0.05)

In order to confirm the biological significance of the negative regulation of Gal-7 in a clinical context, we analyzed a cohort of patients to determine the correlation between their survival rate and the absence or presence of Gal-7 and Gal-1. Since Gal-1 is considered as a pro-tumorigenic galectin [30], we anticipated a mutually exclusive expression with respect to Gal-7 as an indication of positive clinical outcome in cervical cancer. In order to prove this assumption, we used Illumina 450 k data from the Cervical Squamous Cell Carcinoma project of The Cancer Genome Atlas (CESC-TCGA; *n* = 185) [31] and performed an Unsupervised Hierarchical Clustering analysis (UHC) (Fig. 2a). Here, two significantly differentiated clusters (*p* = 0.0001) were obtained (Fig. 2b). Group A was integrated by 104 patients expressing high levels of Gal-7 and low levels of Gal-1. In contrast, group B revealed just the opposite composition (low Gal-7 and high Gal-1 expression, data derived from 81 patients). Figure 2e shows a representative immunohistochemical staining of Gal-1/Gal-7 in normal versus tumor tissue (obtained from the Human Protein Atlas) [32]. To address the clinical significance of the UHC analysis, we also assessed the overall survival in this cohort through a Kaplan-Meier survival analysis. Intriguingly, Group A had a significantly higher overall survival rate when compared to group B (*p* < 0.0001) (Fig. 2c). These results suggest that a mutually exclusive expression of Gal-7 and Gal-1 has beneficial prognostic value in CESC patients.

Tumors are known to downregulate incompatible genes through epigenetic mechanisms, such as gene methylation [33]. Therefore, we analyzed if this was the mechanism behind Gal-7 repression in the cohort. We found a high correlation (*p* = 0.001) between the clinical outcome and the degree of expression of the Gal-7 gene, which in turn inversely correlated with methylation (Fig. 2d) between positions −499 to +100 relative to the initiation site (Additional file 1: Table S1). Our data suggest that Gal-7 negative regulation is a biological phenomenon with a strong impact in the outcome of cervical cancer patients.

### Hypermethylation is responsible for reduced Gal-7 expression in CaCx cells

**Fig. 3:**
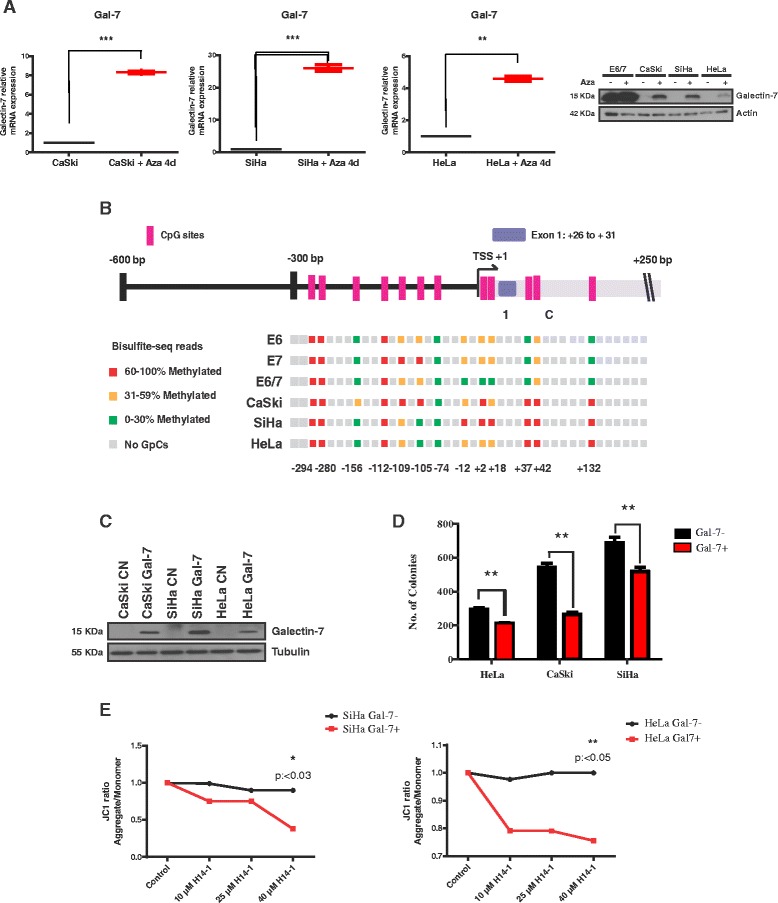
Methylation analysis and re-expression of Gal-7 in CaCx cell lines. **a** qPCR and Western blot of Gal-7 four days after 5′-Azacyditine (Aza) treatment. Actin was used as the loading and transfer control. **b** Above: schematic overview of the Gal-7 gene: the locations of CpG sites, the transcriptional start site (TSS) and the first exon are indicated. Below: percentage of methylation after bisulfite pyrosequencing analysis. **c** Western blot after reconstitution of Gal-7 in CaCx cells and mock infected control cells. Tubulin was used as the loading and transfer control. **d** Colony formation assay of Gal-7+ CaCx cells (Gal-7+, red) versus mock-infected cells (Gal-7-, black). **e** Induction of apoptosis due to loss of the mitochondrial transmembrane potential after incubating Gal-7+ and Gal-7- CaCx cells with increasing amounts of H14-1. (^C^Kruskall-Wallis *P* < 0.0001, Dunn’s Multiple Comparison Test *P* < 0.05; ^E, F G^ *T* Test, *P* < 0.05, ** means moderated significant)

As is indicated by the meta-analysis, we examined whether the Gal-7 gene became *de novo* methylated during the multi-step progression to cervical cancer. For this purpose, cells were treated with 5′-azacytidine as a demethylating agent. Re-expression of the Gal-7 gene could be observed after demethylation (Fig. 3a). Protein recovery was not as strong as in HPV16-immortalized keratinocytes (Fig. 3a, right panel), implying that additional mechanisms control endogenous Gal-7 expression or protein stability in CaCx cells [34].

*In silico* analysis using the Eukaryotic Promoter Database [35] revealed several CpG sites at the 5′end as well as inside the gene (Fig. 3b). After bisulfite-sequencing, a methylation pattern from positions −300 to −12 was almost identical in all cell lines (Fig. 3b). Cervical carcinoma cells revealed strong hypermethylation of CpG sites localized within the first intron (at positions +2 to +132), whereas the same region was almost methylation-free in HPV16 Gal-7+ immortalized keratinocytes. These data suggest a gradual de novo methylation of Gal-7 during HPV-induced carcinogenesis.

### Reconstitution of Gal-7 in CaCx cells conferred sensitivity to apoptosis and anchorage-independence

We next examined the phenotypic changes of CaCx cells after retrovirus-mediated Gal-7 reconstitution in vitro (Fig. 3c). Low-attachment and anchorage-independence are well-known and stringent in vitro parameters for transformation [17]. In a tumor formation assay in vitro, colony formation capacity was found to be significantly lower in Gal-7+ cells than in mock-transduced controls (Fig. 3d). Moreover, re-expression also enhances the susceptibility to apoptotic stimuli, which is consistent with the pro-apoptotic function of Gal-7 in keratinocytes [36]. This was further confirmed by treating cells with the specific Bcl-2 inhibitor and apoptosis inductor HA14-1 [37]. Subsequent staining with the lipophilic cationic dye JC-1 revealed lower cell viability as determined by the loss of the mitochondrial transmembrane potential (ΔΨm) (Fig. 3e). Our data shows that Gal-7-expressing cells are more susceptible not only to intrinsic, but also to extrinsic apoptotic signals [14]. We show that the expression of Gal-7 decreases cell viability and induces a apoptotic response in transfected cells.

### Impact of Gal-7 re-expression on cellular networks of CaCx cells

**Fig. 4:**
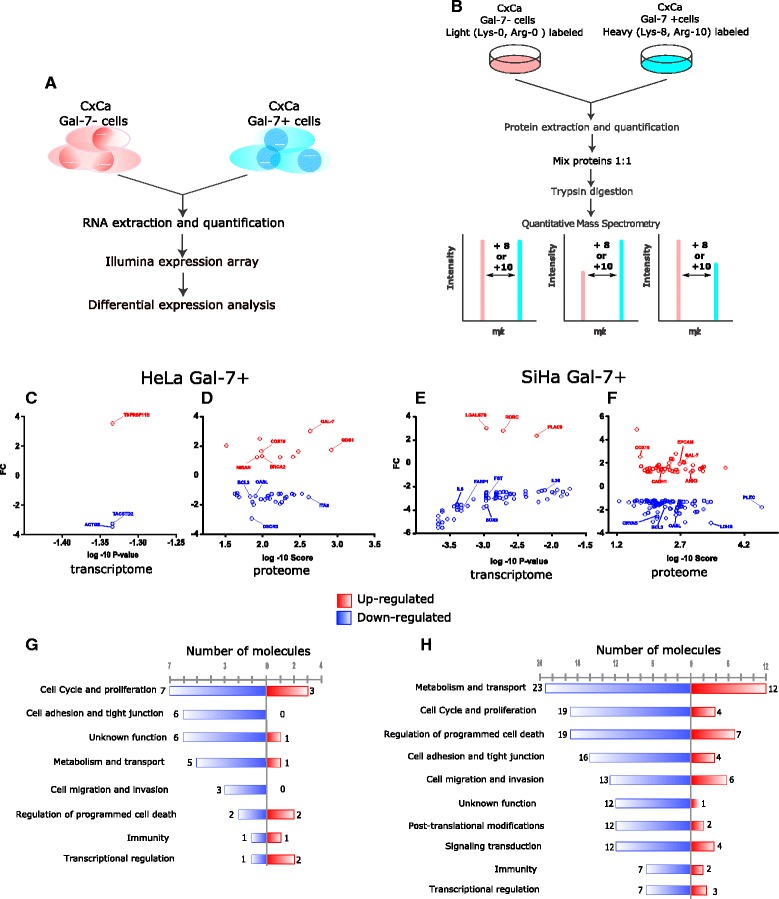
Changes in gene and protein expression in Gal-7+ CaCx cell lines. **a** Experimental strategy for differential gene expression analysis in Gal-7+ CaCx cell lines. **b** SILAC proteome experiments for the profiling of Gal-7+ cell lines. **c**-**f** Differentially expressed transcripts in HeLa (**c**) and SiHa (**e**) Gal-7+ cell lines in vitro with respect to Gal-7- controls. The X axis is the *P* value in log10 scale; the Y axis is the fold change. Differentially expressed proteins between HeLa (**d**) and SiHa (**f**) Gal-7+ cell lines in vitro are shown aside. The X axis represents the score in log10 scale (the identification score was obtained from the average score reported by triplicate identification experiments) and the Y axis is the fold change (FC). **g**, **h** Functional modules affected in Gal-7+ HeLa (**g**) and SiHa (**h**) cells (red: up-regulation, blue: down-regulation)

Having shown that Gal-7 re-expression negatively affects cell growth and impairs colony formation by means of apoptotic signals, we hypothesized that there are deep changes in the cellular networks in response to Gal-7 re-introduction. In order to analyze this question at system-level, we combined the results from microarray expression profiling (Fig. 4a) with a SILAC-based proteomic approach (Fig. 4b). In total, 213 candidate genes were identified in vitro as being differentially expressed either at the RNA or protein level (38 in HeLa Gal-7+ and 185 in SiHa Gal-7+ cells versus mock-transduced Gal-7- control cells). Surprisingly, only three genes were differentially regulated in HeLa Gal-7+ cells at the transcriptome level (Fig. 4c). The proteomic analysis of HeLa/HeLa Gal-7+ revealed 35 proteins that were differentially expressed in three biological replicates (FDR 5 %, Fig. 4d). In the case of SiHa/SiHa Gal-7+ cells, 60 mRNAs were found to be differentially transcribed (Fig. 4e). The proteomic analysis showed 125 differentially regulated proteins (FDR 5 %, Fig. 4f). The difference between transcriptome and proteome suggests that there are post-transcriptional regulation mechanisms affecting protein expression levels.

To study the biological processes in which the identified molecules are involved, we performed a functional analysis of proteins and transcripts using levels 3 and 4 of the “Biological processes” Gene Ontology (GO) [38] through which 120 GOs were obtained (q-value = 0.001). We condensed the redundant GOs to obtain “functional modules” by pooling the GO domains according to their participation in pathways and cancer hallmarks [39]. As summarized in Fig. 4g and h (Additional file 1: Table S4 and S7), HeLa/HeLa Gal-7+ and SiHa/SiHa Gal-7+ shared eight functional modules, but their composition and the extent of regulation between them was different. Moreover, two additional modules were recognized in SiHa, namely “Signal transduction” and “Post-translational modifications” (Fig. 4h). Notably, although we studied the same cancer entity, our proteo-transcriptomic data show that re-expression of Gal-7 can trigger different cell-context dependent responses but leads to a convergent phenotype, as reflected in specific biological processes and cancer hallmarks.

### Mouse microenvironment pressure exerts differential gene expression in Gal-7+ tumors

**Fig. 5:**
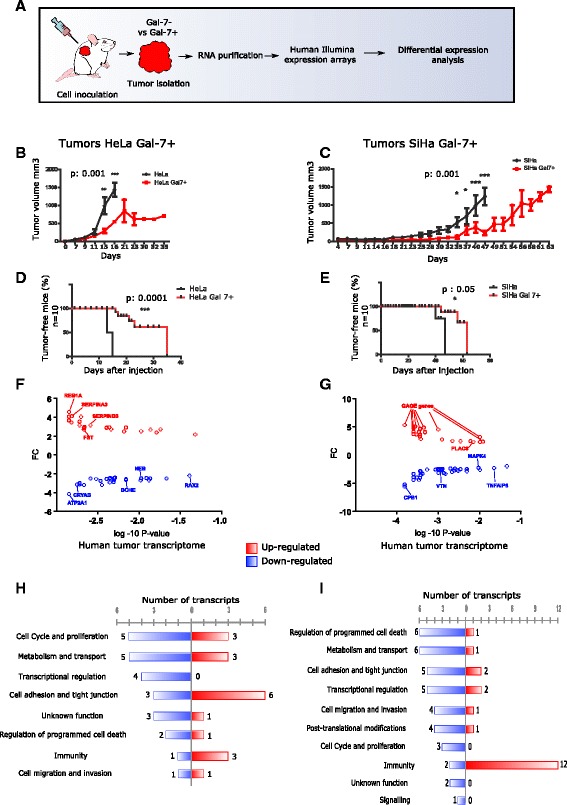
The microenvironment induces changes in Gal-7+ tumors. **a** Experimental strategy. **b** and **c** Growth of Gal-7+ and Gal-7- Hela and SiHa tumors. **d** and **e** Incidence of tumors in injected mice. **f**, **g** Gene profiling analyses in Gal-7+ and Gal-7- HeLa and SiHa tumors. X axis: *P* value in log10 scale, Y axis: fold change (FC). **h**, **i** Functional modules and differentially expressed transcripts in Gal-7+ HeLa. **h** and SiHa (**i**) tumors in vivo. (^B C D E^ ***p < 0.001; **p < 0.01; *p < 0.05 Student’s *t* test). (*red*: up-regulation, *blue*: down-regulation)

To investigate if Gal-7 reconstitution impairs the tumorigenicity of xenografts, HeLa and SiHa Gal-7 + cells, as well as their mock Gal-7- control cells, were subcutaneously injected into athymic nude mice and tumor growth was monitored. The tumors were excised when they reached a volume between 800–1000 mm^3^ and were used for transcriptome analysis (Fig. 5a). Consistent with the lower colony formation capacity and higher sensitivity to apoptotic signals under in vitro conditions, both HeLa Gal-7+ (Fig. 5b, d) and SiHa Gal-7+ cells (Fig. 5c, e) showed a significant impairment in their tumor formation capacity.

**Fig. 6:**
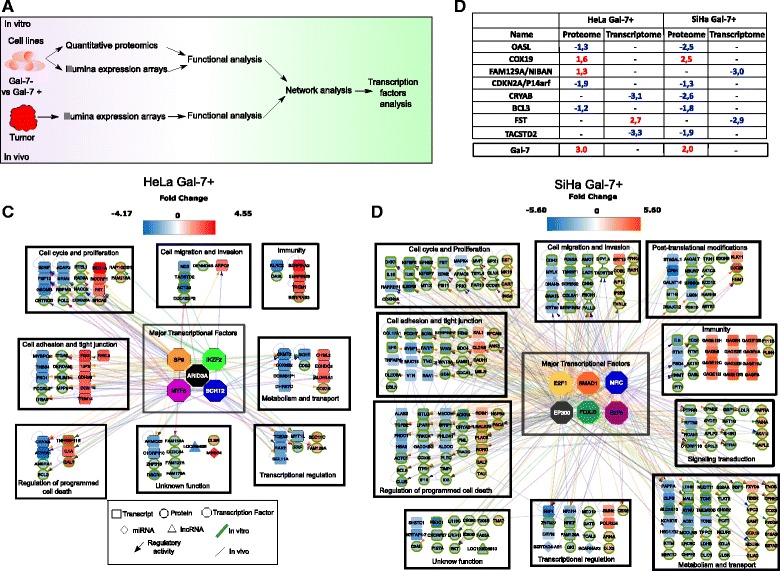
Differential network analysis of Gal-7+ CaCx cells and tumors. **a** Integrative analysis strategy. **b** Common candidates that were differentially regulated at the transcriptional or translational level compared against their Gal-7- controls. Numbers indicate fold changes. **c**, **d** Differential network analysis in Gal-7+ HeLa and SiHa cells and tumors. Color depth represents the fold change with respect to the control. *Red*: up-regulation, *blue*: down-regulation. Major transcription factors (MTF) are shown in the center. The colored lines derived from MTFs show the transcriptional relationship. The shape of the symbols indicates the following: square for transcript, circle for protein, hexagon for TF, diamond for miRNA, and triangle for lncRNA. Wide, green shape edges represent that the information for this molecule was obtained in vitro, while the thin, black edges mean in vivo molecule information

To understand this phenomenon at the molecular level, we performed microarray gene profiling to monitor changes that could be linked to decreased tumorigenicity and animal survival. A total of 42 differentially expressed genes were found in HeLa Gal-7+ cells (Fig. 5f) that could be divided in 8 functional modules (Fig. 5h, Additional file 1: Table S4). In SiHa Gal-7+ cells, 58 genes were differentially regulated (Fig. 5g), and these could be split into 10 functional modules (Fig. 5i, Additional file 1: Table S7), where only PLAC8 and TCN1 were in common. On the other hand, Gal-7 re-expression led to the identification of 8 genes that were regulated in both CaCx cell lines, either at transcriptional or translational level (OASL, COX19, FAM129A/NIBAN, CDKN2A/p14arf, CRYAB, BCL3, FST and TACSTD2) (Fig. 6b). We validated their expression by qPCR, and observed whether the same genes were affected when Gal-7 reconstituted HPV16-positive CaSki cells were examined (Additional file 5: Figure S2). Our data indicate that Gal-7 has a general impact on these network components, which are shared among the three different cervical carcinoma cell lines. These results highlight the complex influence of the microenvironment on the modulation of gene expression in comparison to the in vitro growth.

Living systems are dynamic and complex. Their behavior may be hard to predict from the properties of individual parts, since their interactions produce emergent properties [40, 41]. To get a more accurate insight into the emergent properties of the Gal-7-dependent regulation, we performed an integrative analysis using the transcriptome and proteome biological layers (Fig. 6a). We used the Cytoscape software [42] to build a network for each cell line, and we made a prediction of transcriptional factors (TFs) with iRegulon [43]. The entities were grouped according to their participation in each synthetic module. In HeLa Gal-7+ cells (Fig. 6c), five major TFs are able to control about 60 % of all the genes in the network; these are SP9, ARID3A, MYF5, IKZF2 and SCRT2 (Additional file 1: Table S5). When the function of each individual module was dissected, we found an up-regulation of several candidates involved in the impairment of growth and proliferation (cell cycle and proliferation/regulation of programmed cell death modules), but also in cell adhesion, migration, metabolism, tissue organization and integrity.

The integrative analysis of SiHa Gal-7+ cells (Fig. 6d) showed 6 major TFs that are able to regulate up to 65 % of the genes within this network: E2F1, E2F6, EP300, SMAD1, NFIC and FOXJ3 (Additional file 1: Table S6). Despite having different members, we found a regulation of the same modules that were present in HeLa Gal-7. This could account for the similar phenotypes showed by our xenotransplantation analysis. Notably, there were also changes in genes involved in post-translational modifications and signaling transduction, two modules that were not identified in the Hela Gal-7 network (Additional file 1: Table S7).

### Gal-7 re-expression in tumors modulates transcriptional changes in their associated microenvironment

**Fig. 7:**
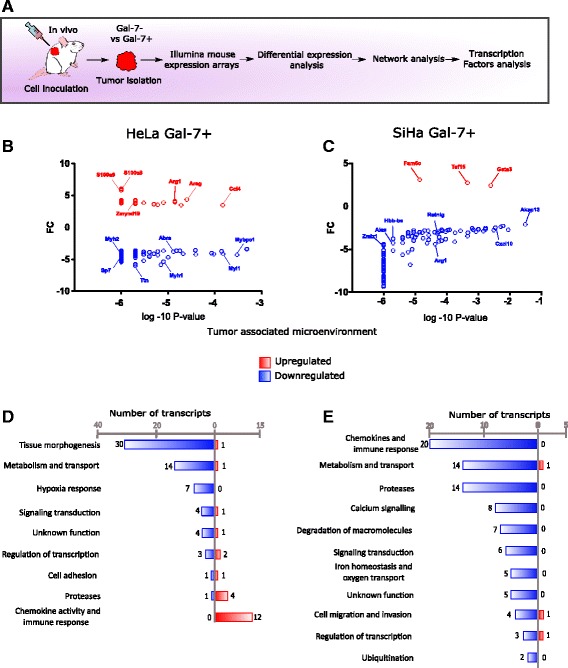
Differential gene profiling of the Gal-7+ tumor associated microenvironment. **a** Experimental strategy. **b**, **c** Differential gene profiles of the of Gal-7+ HeLa and SiHa TAM with respect to controls. X axis: *P* value in log10 scale; Y axis: fold change (FC). **d**, **e** Modules and differentially expressed transcripts in Gal-7+ HeLa (**d**) and SiHa (**e**) TAM (up-regulation in *red*, down-regulation in *blue*)

Xenografting of human cancer cells into mice gives us a unique opportunity to study the crosstalk between the tumors and their microenvironment. To follow that purpose, we analyzed how Gal-7 reconstitution shapes the behavior of the microenvironment and the infiltration of immune cells into tumor tissues. To selectively obtain the gene expression profile of infiltrates (e.g. tumor-associated-epithelial, stromal, vascular and immune cells), we followed the competitive cross-species hybridization of microarray experiment (CHME) strategy [44] of the xenografts (Fig. 7a). In the HeLa-Gal-7+ Tumor associated microenvironment (TAM), a total of 87 differentially regulated mouse genes were revealed (Fig. 7b). Subsequent functional module analysis assembled these genes into 9 modules (Fig. 7d), where “tissue morphogenesis”, “metabolism and transport”, and “chemokine activity and immune response” comprised most of the differentially expressed genes. A module related to “hypoxia response” appears only in this TAM. In the SiHa Gal-7+ TAM, 91 differentially regulated genes were found (Fig. 7c). 11 functional modules could be clustered (Fig. 7e), mostly showing a strong down-regulation in their differentially expressed genes. Notably, while in the HeLa-Gal-7+ TAM the genes related to “chemokine activity and immune response” and “proteases” were over-regulated, in the corresponding modules of the SiHa-Gal-7+ TAM all the genes were down-regulated. Also remarkable is the inclusion of two additional modules in the SiHa-Gal-7+ microenvironment: “calcium signaling” and “degradation of macromolecules”. These results suggest that the mechanisms that determine the growth impairment phenotype in response to Gal-7 reconstitution depend on the cellular context, supporting the notion of the uniqueness of cancer cells.

**Fig. 8:**
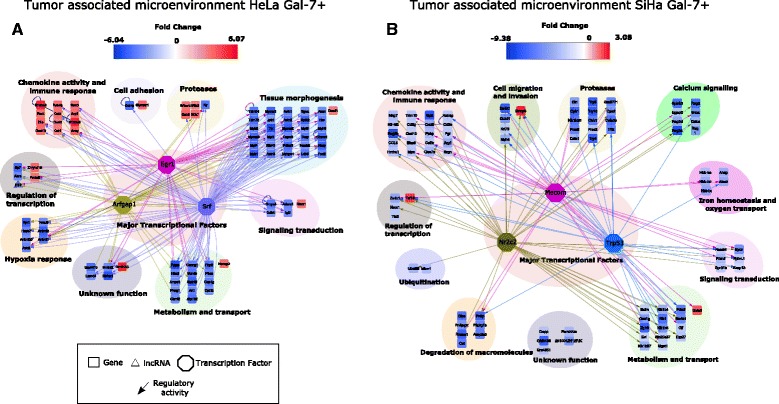
Differential network analysis of the Gal-7+ tumor associated microenvironment. Differential network analysis in Gal-7+ HeLa (**a**) and SiHa (**b**) TAM. Color depth represents the fold change. MTFs are shown in the center. The colored lines derived from MTFs show the transcriptional relationship. The shape of the symbols indicates the following: square for transcript, hexagon for TF, and triangle for lncRNA (up-regulation in *red*, down-regulation in *blue*)

We made a network and TF analysis of the TAM’s of the Gal-7+ tumors. In the HeLa-Gal7+ TAM, regulation of all 9 modules could be attributed mainly to three mouse TFs: Arfgap1, Egr1 and Srf (Fig. 8a and Additional file 1: Table S8). Interestingly, in the “Regulation of transcription” module we found the down-regulation of the Abra gene, which activates Srf and Egr1 [45, 46]. This confirmed our TF prediction and could provide an explanation for the generalized down-regulation of genes. In the “chemokine activity and immune response” module, an up-regulation of several genes (Additional file 1: Table S9) could be discerned, arguing for a wide immune host response against the Gal-7 expressing tumor.

To further support the potential immune mechanism in this system, differentially expressed genes were also analyzed using the immunological genome project (IGP) database [47]. The HeLa Gal-7+ mouse microenvironment was enriched with the expression of Stfa1, Stfa2, Retnlg, S100a8 and S100a9. The two latter marker genes are constitutively expressed by neutrophils, activated monocytes and macrophages. We also identified IL1β, CXCL2, CCL3, CCL4 and Arg1, characteristic genes expressed in macrophages, NK cells, and activated polymorph-nuclear neutrophils, respectively. Moreover, Galnt9 is present only in macrophages and monocytes (Additional file 6: Figure S3B), while Zmynd19 expression is found within hematopoietic stem and multi-lineage progenitor cells (Additional file 6: Figure S3A). The SiHa Gal-7+ microenvironment network analysis showed strong repression in all the functional modules. The major TFs that drove the network were Trp53, Nr2c2 and Mecom (Fig. 8b and Additional file 1: Table S10). A detailed description of some important genes is provided in Additional file 1: Table S11.

Using the IGP database, we mapped the up-regulated genes in the SiHa Gal-7+ mouse microenvironment. Here, we found that the expression of Taf15 (TATA Box Binding Protein (TBP)-Associated Factor), amongst others, was restricted to B-cells, dendritic and NK cells, but not to stromal cells. Whether SiHa Gal-7+ cells induce an environment that suppresses the infiltration of tumor-associated stromal and immune cells awaits further elucidation. Altogether, these data suggest a possible role of the innate cytotoxicity and an adaptive antibody and cytokine secretion mechanism in the growth impairment of SiHa Gal-7+ tumors.

## Discussion

Cervical cancer is a highly robust and flexible network [48] that is shaped by particular microenvironments [49]. Consequently, the combination of clinical data and integrative system-level approaches [50–53] is necessary to tackle the inherent complexity of the disease [54, 55]. In fact, the network of CaCx cells is deeply influenced by the expression of the E6 and E7 high-risk-HPV oncoproteins, which always attack hubs to get selective advantage [56, 57]. These events occur not only via interfering with the p53 and the retinoblastoma (pRB) pathways [58], but also through a direct evasion of anti-tumorigenic responses by 1) impairing central immunomodulatory molecules including type I interferons [59], IL-1β [60], and chemokines [61], and 2) acquiring resistance to immune (CTL and NK)-mediated apoptosis [62, 63].

### Gal-7 is incompatible with the malignant phenotype in cervical cancer

Gal-7 possesses well known functions in pro-apoptotic response [7, 64, 65], regulation of cellular proliferation [66], and epithelial homeostasis [67]. In our study, several lines of evidence pinpointed Gal-7 as incompatible with the selection process during cervical carcinogenesis. Such phenomenon is clearly visible in the negative regulation of the gene at the transcript and protein level, in clinical samples as well as in cell lines. As shown by bisulfite sequencing in our cell lines and meta-analysis of methylation arrays from clinical samples, the absence of Gal-7 could be attributed to an intragenic *de novo* methylation event, a mode of gene silencing also observed in gastric cancer [68]. Gal-7 expression, however, could be restored by treating cell lines with a demethylating agent (Fig. 3a).

As depicted in our results, Gal-7 reconstitution does not lead to the suppression of the tumorigenesis, but rather results in a significant reduction of colony formation capacity and an increased sensitivity towards apoptosis in vitro, consistent with observations in prostate cancer cells [65] and in colon carcinoma cells [69]. Xenotransplantation in nu/nu mice also showed a strong tumor growth retardation, a phenotype in common with Gal-7+ colon carcinoma cells in SCID mice [69]. Interestingly, other cancer models display an opposite behavior. Gal-7 is over-expressed in lymphoma cells and is related to promoting tumorigenesis by induction of MMP9 [70] and in ovarian cancer it is associated with cell proliferation and poor prognosis [71].

### Gal-7 reconstitution triggers changes in proteins and mRNA in the cell lines and tumor

Our results demonstrated that re-expression of Gal-7 in the HeLa and SiHa cell lines triggers a cascade of changes at both transcript and protein levels. In vitro, HeLa Gal-7+ only yielded 3 differentially expressed genes and 35 proteins, while in SiHa Gal-7+ we found 60 differentially expressed transcripts and 125 proteins. Several studies show significant differences between the abundance ratio of the messenger RNA transcript and the corresponding protein product [72, 73], a phenomenon that could be mainly due to post-transcriptional and translational regulation, as well as protein and mRNA turnover [74].

Within tumors, a continuous selection exists for cells with the highest survival and proliferative advantage; moreover, microenvironmental conditions provide selective pressure for tumor evolution and the progression to invasion [75]. One of the most probable mechanisms for inhibition of the tumor growth could be the expression of genes with “asymmetric activity” capable of suppressing tumorigenicity, without affecting in vitro growth [76].

Three central points can be extracted from the present work, the first of which is related to the robustness of the cancer network. Despite the gene composition, there is a powerful convergence in the functional modules towards the hallmarks of cancer, as suggested by our previous work [48]. A factor to be discussed is if these functional modules are a response to the imminent cell destruction, a desperate response to survive, or a combination of both. An example in HeLa Gal-7+ cells is the case of over-expression in vitro (protein) and in vivo (transcript) of SERPIN3B, a protease inhibitor that might act in protecting cancer cells through inhibition of apoptotic signals [77, 78] and is capable of inducing epithelial-mesenchymal transition [79]. Another one is the over-expression of GAGE genes in SiHa Gal-7+ cells, whereas their expression in normal tissues is limited to the germ cells of immune-privileged organs [80]. It is possible to speculate whether this response is an attempt to escape the immune modulation.

### System-level response in Gal-7 network is context dependent

We selected the SiHa and HeLa cell lines because they are transformed by the most frequent high-risk HPV types (HPV16 and HPV 18, respectively). The E6/E7 oncoproteins of both HPV types possess subtle differences in their interaction partners [81], in the affinity of these interactions [82] and, consequently, in the mechanisms of transformation [83, 84]. These differences could potentially generate a chain reaction in the whole cell network, and each cell line could give a particular response due to its specific background. Although tumors of similar types and clinical outcomes can have patterns of gene expression or mutations that are strikingly different, these behaviors recurrently target the same pathway or network [55]. Which leads us to central point number two: while each cancer is as unique as its patient, there is convergence towards a very similar phenotype.

Although Gal-7 affects the specific cervical cancer networks in different ways, we identified a group of eight molecules that are regulated in both cell lines: CRYAB, TACSTD2, BCL-3, COX19, OASL, CDKN2A/p14ARF, NIBAN/FAM129A, and FST. All these proteins have been previously described as key regulators of tumorigenesis in some cancers (Table 1).

**Table 1:** Common proteins between Hela and SiHa Gal-7+ cell networks

Name	Process	Model	Reference
CRYAB	Associated with poor prognosis and metastasis, negative regulator of TRAIL-mediated apoptosis, related to hypoxic survival and metastasis	Several	[101–103]
TACSTD2	Correlated with malignant progression, apoptosis resistance and proliferation	Cervical	[104–106]
BCL-3	Negative regulator of apoptosis	Several	[107, 108]
COX19	Biogenesis and regulation of mitochondrial respiratory homeostasis	Several	[109, 110]
OASL	Displays antiviral activity, responsive to type I interferon signal	Several	[111, 112]
CDKN2A	Overexpression is used as a biomarker for progressing lesions	Cervical	[113]
NIBAN/FAM129A	Anti-apoptotic activity	Several	[114–116]
FST	Attenuates rRNA synthesis and trigger FST-mediated apoptosis	Cervical, Breast	[117, 118]

Common regulated proteins in the HeLa and Siha Gal-7+ cell networks and their associated functions in different cancer models

In our differential network analysis, we predict the TFs that can potentially regulate the respective modules. The HeLa Gal-7+ network can be controlled by five major TFs: SP9, ARID3A, MYF5, IKZF2 and SCRT2, while in SiHa Gal-7+ cells, six major TFs could be identified: E2F1, E2F6, EP300, SMAD1, NFIC and FOXJ3 (Additional file 1: Tables S5 and S6). Surprisingly, we found a deep connection involving E2F TFs in both cell lines. In HeLa-Gal-7+, ARID3A, also known as E2FBP1, is a TF involved in the control of cell cycle progression by the RB1/E2F1 pathway, and is induced in response to p53 [85, 86]. In the SiHa-Gal-7+ network, E2F1, E2F6, and FOXJ3 are involved in pathways of cell cycle control, where FOXJ3 may be involved in regulating a network of zinc finger–binding proteins that may affect gene expression themselves in response to the E2F pathway [87, 88]. E2F TFs are part of a wide network involved in differentiation, apoptosis and immune response [89–91].

### Gal-7 plays a role in the crosstalk between cancer cells and the tumor associated microenvironment

Since a tumor is always composed of heterogeneous cells in a context-dependent manner [92], expression profiling of infiltrates provides an insight into the bi-directional communication between transformed cells and the microenvironment. Our CHME strategy allowed us to monitor the expression profile of genes that were indicative of the infiltration of potential tumor-associated epithelial, stromal and immune cells [93, 94].

The integrative analysis of Gal-7+ TAM cells revealed deep changes, presumably activated by the interaction of the tumor and microenvironment networks. In the HeLa Gal-7+ TAM, the chemokine and immune responses stood heavily stimulated. Meanwhile, 30 genes related to tissue morphogenesis, metabolism and transport, and hypoxia response were down-regulated. Excitingly, we found the interplay between Abra (STARS) and its targets, Srf and Egr1. These three TFs have an important role in the remodeling, homeostasis and metabolism of skeletal muscle [95], and appear to be under control of AKT [96]. In the SiHa Gal-7+ TAM, there is not an obvious interplay between the predicted and regulated TFs. Nevertheless, we observed the negative regulation of almost 97 % of all the modules, and specifically of some genes that promote carcinogenesis, such as CCL5, CXCL9 and CXCL10 (see Additional file 1: Table S11). These genes are characteristic of tumor-associated macrophages and stromal fibroblasts.

It is noteworthy that the role of the immune system is highlighted by the expression of specific genes of NK cells in the HeLa Gal-7+ TAM. Meanwhile, in SiHa Gal-7+ TAM, we found evidences of activated monocytes and the infiltration of macrophages. This would indicate that the innate and adaptive immune system, stroma cells, and fibroblasts play a complex leading role in impairing the growth of Gal-7+ tumors.

**Fig. 9:**
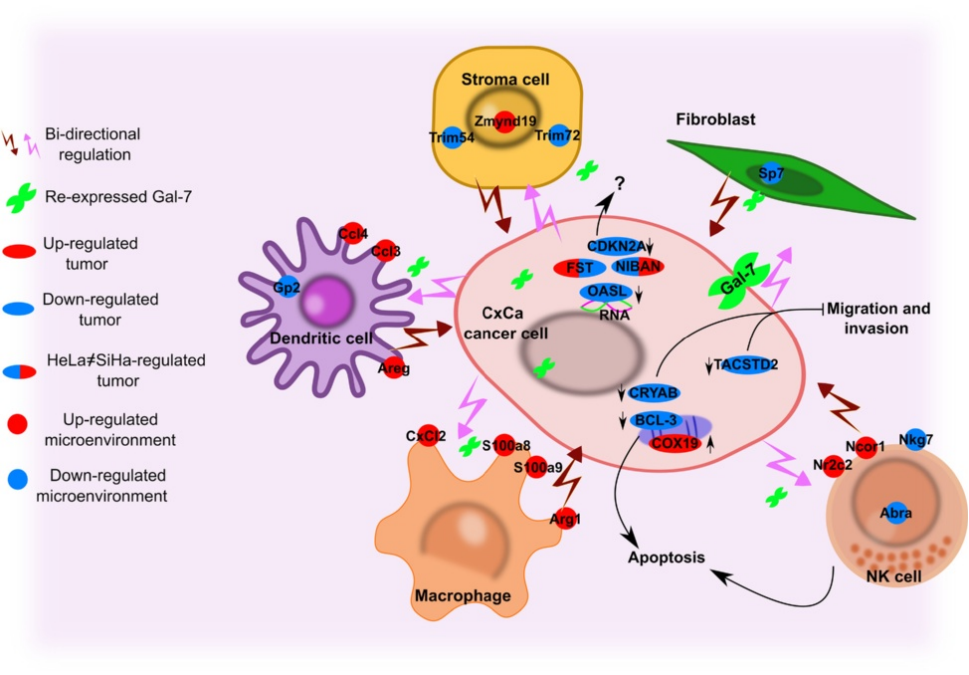
Bidirectional crosstalk between Gal-7+ tumors and their associated microenvironments. The expression of genes related to stromal cells, cancer associated fibroblasts, and immune cells results in an aggressive crosstalk that could potentially decide the fate of the tumor: proliferation and immune evasion, or immune destruction. The role of the common proteins in the Gal-7 network remains partially unknown. The color of the participant transcripts and proteins represents differential expression (up-regulation in *red*, down-regulation in *blue*)

These observations sustain the third central point of our work: there is an aggressive negotiation between the tumor and the TAM, where constant changes in both networks shape their overall behavior and fate. We summarize our three central points in Fig. 9. Supporting our empirical observations, formal mathematical modeling demonstrated that tumor mass evolution is the integrated result of the dynamics of two linked complex systems, tumor cell population and tumor microenvironment [97], in addition to emergent properties caused by the genomic background [98] and clinically significant macro-environmental variables [99, 100].

Numerous studies reinforce the hypothesis of a complex control by the stem cells over their niche, allowing for immunologic privilege. This effect allows them to exert their multipotentiality and proliferate at a different rate than the rest of the tissue [85–87]. Excitingly, it is known that secreted galectins are able to exert immunomodulation in the stem cell niche [88]. For instance, Gal-9 has recently been shown to prevent allo-reactivity of immune cells in a non-cancerous model [89], and has been shown to be a major mediator of the anti-proliferative and functional effects of multipotent mesenchymal stromal cells on T and B cells [90]. Moreover, Gal-3 is over-secreted in pancreatic cancer stem-like cells [91]. The interaction with stem cells could explain why Gal-1 and Gal-3 were found to drive a strong immunomodulation in cancer in a previous work [1].

Based on the previous evidence, we speculate that galectins (including Gal-1 and Gal-7) could act like molecular masterminds behind the regulation of immune activity in different cancers, particularly through interactions with stem cells. A delicate equilibrium in galectin expression could regulate immunosurveillance, while its disturbance by tumors and cancer stem cells would lead to immune escape, tumor growth and metastasis. In our Gal-7+ xenografts, the re-expression of Gal-7 could have either restored immunosurveillance or counteracted any immune escape mechanisms, which would explain the immune signature found in the TAM. Future studies should confirm if Gal-7 plays a direct role in the recruitment of immune cells, as well as whether they interact in any way with cancer stem cells to achieve such results.

## Conclusions

Gal-7 expression presents an interesting correlation with a significantly better survival rate of patients diagnosed with cervical cancer. This phenomenon highlights the biological importance of Gal-7 repression in CaCx.

The cellular networks reconstructed by Gal-7 expression prove three central hypotheses implicated in the emergency of properties of the Gal-7 network: 1) Despite the gene composition, there is a convergence in functional modules that potentially govern the retardation of growth in CaCx Gal-7+ cells. 2) Regardless of their anatomical and cellular origin, each cancer is as unique as its patient. 3) A tumor-microenvironment cross-talk in response to Gal-7 reconstitution leads to an alteration of regulation and interaction networks, providing a great insight into molecular basis of the Gal-7 network.

Integrating several biological layers of information and comparing the variability of the dynamics and the complexity of the networks, using tools of the emergent field of differential network biology, is essential to gain definitive insights into cancer biology at the molecular level.

## Additional files

Additional file 1: Additional Tables. **Table S1**. Genomic ranges of methylation sites within the Gal-7 gene obtained from TCGA-CESC data. **Table S2.** Primers used for the CpG methylation analysis by bisulfite-PCR-pyrosequencing. **Table S3.** Primers used for the quantification of genes commonly affected in HeLa and SiHa Gal-7+ cells. **Table S4.** Relevant cancer related genes and proteins differentially regulated in the HeLa Gal-7+ cell network. **Table S5.** Major Transcriptional factors identified in the HeLa Gal-7+ cell network. **Table S6.** Major Transcriptional factors identified in the SiHa Gal-7+ cell network. **Table S7.** Relevant cancer related genes and proteins differentially regulated in the SiHa Gal-7+ cell network. **Table S8.** Major Transcriptional factors identified in the HeLa Gal-7+ mouse microenvironment network. **Table S9.** Relevant differentially regulated genes identified in the HeLa Gal-7+ microenvironment network. **Table S10.** Major Transcriptional factors identified in the HeLa Gal-7+ mouse microenvironment network. **Table S11.** Relevant differentially regulated genes identified in the SiHa Gal-7+ microenvironment network. (DOCX 150 kb) Additional file 2:
**Supplementary File 1.** Ms/Ms identification spectra and SILAC quantification of representative peptides of the common proteins between HeLa Gal-7+ and SiHa Gal-7+ CxCa cells. (PDF 1703 kb) Additional file 3:
**Supplementary File 2.** Significant and total proteins identified by SILAC in HeLa Gal-7+ and SiHa Gal-7+ CxCa cells. (XLSX 9105 kb) Additional file 4: Figure S1. Patient data of the Scotto and Zhai cohort. (PDF 63 kb) Additional file 5: Figure S2. Transcriptional analysis of the genes identified by the integrative analysis in Gal-7+ CxCa cells. (PDF 343 kb) Additional file 6: Figure S3. Gene mapping of the mouse microenvironment and immune cells in accordance with The Immunological Genome Project. (PDF 263 kb)

## Abbreviations

AQ: Allele quantification
CaCx: Cervical cancer
CESC: Cervical squamous cell carcinoma and endocervical adenocarcinoma
FDR: False discovery rate
FIGO: Federation of gynecology and obstetrics
Gal-1: Galectin-1
Gal-7: Galectin-7
GEO: Gene expression omnibus
SCC: Squamous cervical cancer
GO: Gene ontology
HCD: Higher-energy collisional dissociation
HPV16/18: Human Papillomavirus 16/18
HSIL: High-grade squamous intraepithelial lesion
MS/MS: Tandem mass spectrometry
NES: Normalized enrichment scores
OS: Overall survival
RSEM: RNA-Seq expectation-maximization
SILAC: Stable isotope labeling with amino acids in cell culture
TCGA: The Cancer Genome Atlas

## References

[1] Rabinovich GA, Croci DO (2012). Regulatory circuits mediated by lectin-glycan interactions in autoimmunity and cancer. Immunity.

[2] Kim HJ, Do IG, Jeon HK, Cho YJ, Park YA, Choi JJ, Sung CO, Lee YY, Choi CH, Kim TJ (2013). Galectin 1 expression is associated with tumor invasion and metastasis in stage IB to IIA cervical cancer. Hum Pathol.

[3] Okumura CY, Baum LG, Johnson PJ (2008). Galectin-1 on cervical epithelial cells is a receptor for the sexually transmitted human parasite Trichomonas vaginalis. Cell Microbiol.

[4] Tao L, Han L, Li X, Gao Q, Pan L, Wu L, Luo Y, Wang W, Zheng Z, Guo X (2014). Prevalence and risk factors for cervical neoplasia: a cervical cancer screening program in Beijing. BMC Public Health.

[5] Saussez S, Kiss R (2006). Galectin-7. Cell Mol Life Sci.

[6] Bernerd F, Sarasin A, Magnaldo T (1999). Galectin-7 overexpression is associated with the apoptotic process in UVB-induced sunburn keratinocytes. Proc Natl Acad Sci U S A.

[7] Villeneuve C, Baricault L, Canelle L, Barboule N, Racca C, Monsarrat B, Magnaldo T, Larminat F (2011). Mitochondrial proteomic approach reveals galectin-7 as a novel BCL-2 binding protein in human cells. Mol Biol Cell.

[8] Tsai CJ, Sulman EP, Eifel PJ, Jhingran A, Allen PK, Deavers MT, Klopp AH (2013). Galectin-7 levels predict radiation response in squamous cell carcinoma of the cervix. Gynecol Oncol.

[9] Scotto L, Narayan G, Nandula SV, Arias-Pulido H, Subramaniyam S, Schneider A, Kaufmann AM, Wright JD, Pothuri B, Mansukhani M (2008). Identification of copy number gain and overexpressed genes on chromosome arm 20q by an integrative genomic approach in cervical cancer: potential role in progression. Genes Chromosomes Cancer.

[10] Kosary CL (1994). FIGO stage, histology, histologic grade, age and race as prognostic factors in determining survival for cancers of the female gynecological system: an analysis of 1973–87 SEER cases of cancers of the endometrium, cervix, ovary, vulva, and vagina. Semin Surg Oncol.

[11] Zhai Y, Kuick R, Nan B, Ota I, Weiss SJ, Trimble CL, Fearon ER, Cho KR (2007). Gene expression analysis of preinvasive and invasive cervical squamous cell carcinomas identifies HOXC10 as a key mediator of invasion. Cancer Res.

[12] Hudson TJ, Anderson W, Artez A, Barker AD, Bell C, Bernabe RR, Bhan MK, Calvo F, Eerola I, Gerhard DS (2010). International network of cancer genome projects. Nature.

[13] Li B, Dewey CN (2011). RSEM: accurate transcript quantification from RNA-Seq data with or without a reference genome. BMC Bioinformatics.

[14] Kuwabara I, Kuwabara Y, Yang R-Y, Schuler M, Green DR, Zuraw BL, Hsu DK, Liu F-T (2002). Galectin-7 (PIG1) exhibits pro-apoptotic function through JNK activation and mitochondrial cytochrome cRelease. J Biol Chem.

[15] Stone SC, Rossetti RA, Bolpetti A, Boccardo E, de Araujo Souza PS, Lepique AP (2014). HPV16-associated tumors control myeloid cell homeostasis in lymphoid organs, generating a suppressor environment for T cells. J Leukoc Biol.

[16] Piersma SJ (2011). Immunosuppressive tumor microenvironment in cervical cancer patients. Cancer Microenviron.

[17] Rotem A, Janzer A, Izar B, Ji Z, Doench JG, Garraway LA, Struhl K (2015). Alternative to the soft-agar assay that permits high-throughput drug and genetic screens for cellular transformation. Proc Natl Acad Sci U S A.

[18] Kallio MA, Tuimala JT, Hupponen T, Klemelä P, Gentile M, Scheinin I, Koski M, Käki J (2011). Korpelainen EI Chipster: user-friendly analysis software for microarray and other high-throughput data. BMC Genomics.

[19] Masuda T, Tomita M, Ishihama Y (2008). Phase transfer surfactant-aided trypsin digestion for membrane proteome analysis. J Proteome Res.

[20] Fabrik I, Link M, Hartlova A, Dankova V, Rehulka P, Stulik J (2014). Application of SILAC labeling to primary bone marrow-derived dendritic cells reveals extensive GM-CSF-dependent arginine metabolism. J Proteome Res.

[21] UniProt C (2015). UniProt: a hub for protein information. Nucleic Acids Res.

[22] Zhou Y, Cras-Meneur C, Ohsugi M, Stormo GD, Permutt MA (2007). A global approach to identify differentially expressed genes in cDNA (two-color) microarray experiments. Bioinformatics.

[23] Vizcaino JA, Deutsch EW, Wang R, Csordas A, Reisinger F, Rios D, Dianes JA, Sun Z, Farrah T, Bandeira N (2014). ProteomeXchange provides globally coordinated proteomics data submission and dissemination. Nat Biotechnol.

[24] Croft D, Mundo AF, Haw R, Milacic M, Weiser J, Wu G, Caudy M, Garapati P, Gillespie M, Kamdar MR (2014). The Reactome pathway knowledgebase. Nucleic Acids Res.

[25] Kanehisa M, Goto S, Sato Y, Kawashima M, Furumichi M, Tanabe M (2014). Data, information, knowledge and principle: back to metabolism in KEGG. Nucleic Acids Res.

[26] Gene Ontology C, Blake JA, Dolan M, Drabkin H, Hill DP, Li N, Sitnikov D, Bridges S, Burgess S, Buza T (2013). Gene Ontology annotations and resources. Nucleic Acids Res.

[27] Martin A, Ochagavia ME, Rabasa LC, Miranda J, Fernandez-de-Cossio J, Bringas R (2010). BisoGenet: a new tool for gene network building, visualization and analysis. BMC Bioinformatics.

[28] Eisa NH, Ebrahim MA, Ragab M, Eissa LA, El-Gayar AM (2015). Galectin-3 and matrix metalloproteinase-9: perspective in management of hepatocellular carcinoma. J Oncol Pharm Pract.

[29] Aguilar-Lemarroy A, Gariglio P, Whitaker NJ, Eichhorst ST, zur Hausen H, Krammer PH, Rosl F (2002). Restoration of p53 expression sensitizes human papillomavirus type 16 immortalized human keratinocytes to CD95-mediated apoptosis. Oncogene.

[30] Huang EY, Chen YF, Chen YM, Lin IH, Wang CC, Su WH, Chuang PC, Yang KD (2012). A novel radioresistant mechanism of galectin-1 mediated by H-Ras-dependent pathways in cervical cancer cells. Cell Death Dis.

[31] Tomczak K, Czerwinska P, Wiznerowicz M (2015). The Cancer Genome Atlas (TCGA): an immeasurable source of knowledge. Contemp Oncol.

[32] Uhlen M, Oksvold P, Fagerberg L, Lundberg E, Jonasson K, Forsberg M, Zwahlen M, Kampf C, Wester K, Hober S (2010). Towards a knowledge-based Human Protein Atlas. Nat Biotechnol.

[33] Delpu Y, Cordelier P, Cho WC, Torrisani J (2013). DNA methylation and cancer diagnosis. Int J Mol Sci.

[34] Ermakova E, Miller MC, Nesmelova IV, Lopez-Merino L, Berbis MA, Nesmelov Y, Tkachev YV, Lagartera L, Daragan VA, Andre S (2013). Lactose binding to human galectin-7 (p53-induced gene 1) induces long-range effects through the protein resulting in increased dimer stability and evidence for positive cooperativity. Glycobiology.

[35] Dreos R, Ambrosini G, Cavin Perier R, Bucher P (2013). EPD and EPDnew, high-quality promoter resources in the next-generation sequencing era. Nucleic Acids Res.

[36] Lee JS, Lee Y, Jeon B, Jeon Y, Yoo H, Kim TY (2012). EC-SOD induces apoptosis through COX-2 and galectin-7 in the epidermis. J Dermatol Sci.

[37] Gonzalez-Rodilla I, Verna V, Munoz AB, Estevez J, Boix M, Schneider J (2011). Expression of the apoptosis-related genes Bcl-2 and p53 in clinical samples from endometrial carcinoma patients. Anticancer Res.

[38] Gene Ontology C (2015). Gene Ontology Consortium: going forward. Nucleic Acids Res.

[39] Hanahan D, Weinberg RA (2011). Hallmarks of cancer: the next generation. Cell.

[40] Bhalla US, Iyengar R (1999). Emergent properties of networks of biological signaling pathways. Science.

[41] Hornberg JJ, Bruggeman FJ, Westerhoff HV, Lankelma J (2006). Cancer: a systems biology disease. Biosystems.

[42] Shannon P, Markiel A, Ozier O, Baliga NS, Wang JT, Ramage D, Amin N, Schwikowski B, Ideker T (2003). Cytoscape: a software environment for integrated models of biomolecular interaction networks. Genome Res.

[43] Janky R, Verfaillie A, Imrichova H, Van de Sande B, Standaert L, Christiaens V, Hulselmans G, Herten K, Naval Sanchez M, Potier D (2014). iRegulon: from a gene list to a gene regulatory network using large motif and track collections. PLoS Comput Biol.

[44] Park ES, Kim SJ, Kim SW, Yoon SL, Leem SH, Kim SB, Kim SM, Park YY, Cheong JH, Woo HG (2011). Cross-species hybridization of microarrays for studying tumor transcriptome of brain metastasis. Proc Natl Acad Sci U S A.

[45] Arai A, Spencer JA, Olson EN (2002). STARS, a striated muscle activator of Rho signaling and serum response factor-dependent transcription. J Biol Chem.

[46] Kim MJ, Kang JH, Chang SY, Jang HJ, Ryu GR, Ko SH, Jeong IK, Kim MS, Jo YH (2008). Exendin-4 induction of Egr-1 expression in INS-1 beta-cells: interaction of SRF, not YY1, with SRE site of rat Egr-1 promoter. J Cell Biochem.

[47] Mehan MR, Ostroff R, Wilcox SK, Steele F, Schneider D, Jarvis TC, Baird GS, Gold L, Janjic N (2013). Highly multiplexed proteomic platform for biomarker discovery, diagnostics, and therapeutics. Adv Exp Med Biol.

[48] Higareda-Almaraz JC, Valtierra-Gutierrez IA, Hernandez-Ortiz M, Contreras S, Hernandez E, Encarnacion-Guevara S (2013). Analysis and prediction of pathways in HeLa cells by integrating biological levels of organization with systems-biology approaches. PLoS One.

[49] Marsh JL, Jackman CP, Tang SN, Shankar S, Srivastava RK (2014). Embelin suppresses pancreatic cancer growth by modulating tumor immune microenvironment. Front Biosci (Landmark Ed).

[50] Kristensen VN, Lingjaerde OC, Russnes HG, Vollan HK, Frigessi A, Borresen-Dale AL (2014). Principles and methods of integrative genomic analyses in cancer. Nat Rev Cancer.

[51] Mitra S, Das S, Chakrabarti J (2013). Systems biology of cancer biomarker detection. Cancer Biomark.

[52] Kreeger PK, Lauffenburger DA (2010). Cancer systems biology: a network modeling perspective. Carcinogenesis.

[53] Hanash S (2004). Integrated global profiling of cancer. Nat Rev Cancer.

[54] Gulati S, Cheng TM, Bates PA (2013). Cancer networks and beyond: interpreting mutations using the human interactome and protein structure. Semin Cancer Biol.

[55] Krogan NJ, Lippman S, Agard DA, Ashworth A, Ideker T (2015). The cancer cell map initiative: defining the hallmark networks of cancer. Mol Cell.

[56] Higareda-Almaraz JC, Enriquez-Gasca Mdel R, Hernandez-Ortiz M, Resendis-Antonio O, Encarnacion-Guevara S (2011). Proteomic patterns of cervical cancer cell lines, a network perspective. BMC Syst Biol.

[57] Greaves M (2015). Evolutionary determinants of cancer. Cancer Discovery.

[58] Moody CA, Laimins LA (2010). Human papillomavirus oncoproteins: pathways to transformation. Nat Rev Cancer.

[59] Rincon-Orozco B, Halec G, Rosenberger S, Muschik D, Nindl I, Bachmann A, Ritter TM, Dondog B, Ly R, Bosch FX (2009). Epigenetic silencing of interferon-kappa in human papillomavirus type 16-positive cells. Cancer Res.

[60] Niebler M, Qian X, Hofler D, Kogosov V, Kaewprag J, Kaufmann AM, Ly R, Bohmer G, Zawatzky R, Rosl F (2013). Post-translational control of IL-1beta via the human papillomavirus type 16 E6 oncoprotein: a novel mechanism of innate immune escape mediated by the E3-ubiquitin ligase E6-AP and p53. PLoS Pathog.

[61] Hacke K, Rincon-Orozco B, Buchwalter G, Siehler SY, Wasylyk B, Wiesmuller L, Rosl F (2010). Regulation of MCP-1 chemokine transcription by p53. Mol Cancer.

[62] Medema JP, de Jong J, Peltenburg LT, Verdegaal EM, Gorter A, Bres SA, Franken KL, Hahne M, Albar JP, Melief CJ (2001). Blockade of the granzyme B/perforin pathway through overexpression of the serine protease inhibitor PI-9/SPI-6 constitutes a mechanism for immune escape by tumors. Proc Natl Acad Sci U S A.

[63] Vogt M, Butz K, Dymalla S, Semzow J, Hoppe-Seyler F (2006). Inhibition of Bax activity is crucial for the antiapoptotic function of the human papillomavirus E6 oncoprotein. Oncogene.

[64] Barkan B, Cox AD, Kloog Y (2013). Ras inhibition boosts galectin-7 at the expense of galectin-1 to sensitize cells to apoptosis. Oncotarget.

[65] Labrie M, Vladoiu M, Leclerc BG, Grosset AA, Gaboury L, Stagg J, St-Pierre Y (2015). A mutation in the carbohydrate recognition domain drives a phenotypic switch in the role of galectin-7 in prostate cancer. PLoS One.

[66] Chen HL, Chiang PC, Lo CH, Lo YH, Hsu DK, Chen HY, Liu FT. Galectin-7 regulates keratinocyte proliferation and differentiation through JNK-miR-203-p63 signaling. J Invest Dermatol. 2015. 10.1038/JID.2015.366 PMC4803640 26763438

[67] Gendronneau G, Sidhu SS, Delacour D, Dang T, Calonne C, Houzelstein D, Magnaldo T, Poirier F (2008). Galectin-7 in the control of epidermal homeostasis after injury. Mol Biol Cell.

[68] Kim SJ, Hwang JA, Ro JY, Lee YS, Chun KH (2013). Galectin-7 is epigenetically-regulated tumor suppressor in gastric cancer. Oncotarget.

[69] Ueda S, Kuwabara I, Liu FT (2004). Suppression of tumor growth by galectin-7 gene transfer. Cancer Res.

[70] Demers M, Magnaldo T, St-Pierre Y (2005). A novel function for galectin-7: promoting tumorigenesis by up-regulating MMP-9 gene expression. Cancer Res.

[71] Kim HJ, Jeon HK, Lee JK, Sung CO, Do IG, Choi CH, Kim TJ, Kim BG, Bae DS, Lee JW (2013). Clinical significance of galectin-7 in epithelial ovarian cancer. Anticancer Res.

[72] Griffin TJ, Gygi SP, Ideker T, Rist B, Eng J, Hood L, Aebersold R (2002). Complementary profiling of gene expression at the transcriptome and proteome levels in Saccharomyces cerevisiae. Molecular & cellular proteomics: MCP.

[73] Taniguchi Y, Choi PJ, Li GW, Chen H, Babu M, Hearn J, Emili A, Xie XS (2010). Quantifying E. coli proteome and transcriptome with single-molecule sensitivity in single cells. Science.

[74] Vogel C, Marcotte EM (2012). Insights into the regulation of protein abundance from proteomic and transcriptomic analyses. Nat Rev Genet.

[75] Polyak K, Haviv I, Campbell IG (2009). Co-evolution of tumor cells and their microenvironment. Trends in genetics : TIG.

[76] Klein G, Imreh S, Zabarovsky ER (2007). Why do we not all die of cancer at an early age?. Adv Cancer Res.

[77] Ahmed ST, Darnell JE (2009). Serpin B3/B4, activated by STAT3, promote survival of squamous carcinoma cells. Biochem Biophys Res Commun.

[78] Turato C, Buendia MA, Fabre M, Redon MJ, Branchereau S, Quarta S, Ruvoletto M, Perilongo G, Grotzer MA, Gatta A (2012). Over-expression of SERPINB3 in hepatoblastoma: a possible insight into the genesis of this tumour?. Eur J Cancer.

[79] Quarta S, Vidalino L, Turato C, Ruvoletto M, Calabrese F, Valente M, Cannito S, Fassina G, Parola M, Gatta A (2010). SERPINB3 induces epithelial-mesenchymal transition. J Pathol.

[80] Gjerstorff MF, Ditzel HJ (2008). An overview of the GAGE cancer/testis antigen family with the inclusion of newly identified members. Tissue Antigens.

[81] White EA, Sowa ME, Tan MJ, Jeudy S, Hayes SD, Santha S, Munger K, Harper JW, Howley PM (2012). Systematic identification of interactions between host cell proteins and E7 oncoproteins from diverse human papillomaviruses. Proc Natl Acad Sci U S A.

[82] White EA, Howley PM (2013). Proteomic approaches to the study of papillomavirus-host interactions. Virology.

[83] White EA, Walther J, Javanbakht H, Howley PM (2014). Genus beta human papillomavirus E6 proteins vary in their effects on the transactivation of p53 target genes. J Virol.

[84] White EA, Kramer RE, Hwang JH, Pores Fernando AT, Naetar N, Hahn WC, Roberts TM, Schaffhausen BS, Livingston DM, Howley PM (2015). Papillomavirus E7 oncoproteins share functions with polyomavirus small T antigens. J Virol.

[85] Ma K, Araki K, Ichwan SJ, Suganuma T, Tamamori-Adachi M, Ikeda MA (2003). E2FBP1/DRIL1, an AT-rich interaction domain-family transcription factor, is regulated by p53. Molecular cancer research : MCR.

[86] Peeper DS, Shvarts A, Brummelkamp T, Douma S, Koh EY, Daley GQ, Bernards R (2002). A functional screen identifies hDRIL1 as an oncogene that rescues RAS-induced senescence. Nat Cell Biol.

[87] Grant GD, Gamsby J, Martyanov V, Brooks L, George LK, Mahoney JM, Loros JJ, Dunlap JC, Whitfield ML (2012). Live-cell monitoring of periodic gene expression in synchronous human cells identifies Forkhead genes involved in cell cycle control. Mol Biol Cell.

[88] Grant GD, Brooks L, Zhang X, Mahoney JM, Martyanov V, Wood TA, Sherlock G, Cheng C, Whitfield ML (2013). Identification of cell cycle-regulated genes periodically expressed in U2OS cells and their regulation by FOXM1 and E2F transcription factors. Mol Biol Cell.

[89] Martin K, Trouche D, Hagemeier C, Sorensen TS, La Thangue NB, Kouzarides T (1995). Stimulation of E2F1/DP1 transcriptional activity by MDM2 oncoprotein. Nature.

[90] DeGregori J, Kowalik T, Nevins JR (1995). Cellular targets for activation by the E2F1 transcription factor include DNA synthesis- and G1/S-regulatory genes. Mol Cell Biol.

[91] Kowalik TF, DeGregori J, Schwarz JK, Nevins JR (1995). E2F1 overexpression in quiescent fibroblasts leads to induction of cellular DNA synthesis and apoptosis. J Virol.

[92] Yoshihara K, Shahmoradgoli M, Martinez E, Vegesna R, Kim H, Torres-Garcia W, Trevino V, Shen H, Laird PW, Levine DA (2013). Inferring tumour purity and stromal and immune cell admixture from expression data. Nat Commun.

[93] Purdom E, Restall C, Busuttil RA, Schluter H, Boussioutas A, Thompson EW, Anderson RL, Speed TP, Haviv I (2013). Determining epithelial contribution to in vivo mesenchymal tumour expression signature using species-specific microarray profiling analysis of xenografts. Genet Res.

[94] Iorns E, Clarke J, Ward T, Dean S, Lippman M (2012). Simultaneous analysis of tumor and stromal gene expression profiles from xenograft models. Breast Cancer Res Treat.

[95] Lamon S, Wallace MA, Russell AP (2014). The STARS signaling pathway: a key regulator of skeletal muscle function. Pflugers Archiv : European journal of physiology.

[96] Reynolds TH, Merrell E, Cinquino N, Gaugler M, Ng L (2012). Disassociation of insulin action and Akt/FOXO signaling in skeletal muscle of older Akt-deficient mice. Am J Physiol Regul Integr Comp Physiol.

[97] Wolfrom CM, Laurent M, Deschatrette J (2014). Can we negotiate with a tumor?. PLoS One.

[98] Heng HH, Bremer SW, Stevens JB, Ye KJ, Liu G, Ye CJ (2009). Genetic and epigenetic heterogeneity in cancer: a genome-centric perspective. J Cell Physiol.

[99] Grizzi F, Chiriva-Internati M (2006). Cancer: looking for simplicity and finding complexity. Cancer Cell Int.

[100] Grizzi F, Di Ieva A, Russo C, Frezza EE, Cobos E, Muzzio PC, Chiriva-Internati M (2006). Cancer initiation and progression: an unsimplifiable complexity. Theor Biol Med Model.

[101] Qin H, Ni Y, Tong J, Zhao J, Zhou X, Cai W, Liang J, Yao X (2014). Elevated expression of CRYAB predicts unfavorable prognosis in non-small cell lung cancer. Med Oncol.

[102] Volkmann J, Reuning U, Rudelius M, Hafner N, Schuster T, Becker VRA, Weimer J, Hilpert F, Kiechle M, Durst M (2013). High expression of crystallin alphaB represents an independent molecular marker for unfavourable ovarian cancer patient outcome and impairs TRAIL- and cisplatin-induced apoptosis in human ovarian cancer cells. Int J Cancer.

[103] van de Schootbrugge C, Schults EM, Bussink J, Span PN, Grenman R, Pruijn GJ, Kaanders JH, Boelens WC (2014). Effect of hypoxia on the expression of alphaB-crystallin in head and neck squamous cell carcinoma. BMC Cancer.

[104] Liu X, Li S, Yi F (2014). Trop2 gene: a novel target for cervical cancer treatment. J Cancer Res Clin Oncol.

[105] Guerra E, Trerotola M, Aloisi AL, Tripaldi R, Vacca G, La Sorda R, Lattanzio R, Piantelli M, Alberti S (2013). The Trop-2 signalling network in cancer growth. Oncogene.

[106] Trerotola M, Cantanelli P, Guerra E, Tripaldi R, Aloisi AL, Bonasera V, Lattanzio R, de Lange R, Weidle UH, Piantelli M (2013). Upregulation of Trop-2 quantitatively stimulates human cancer growth. Oncogene.

[107] Wakefield A, Soukupova J, Montagne A, Ranger J, French R, Muller WJ, Clarkson RW (2013). Bcl3 selectively promotes metastasis of ERBB2-driven mammary tumors. Cancer Res.

[108] Chang TP, Vancurova I (2014). Bcl3 regulates pro-survival and pro-inflammatory gene expression in cutaneous T-cell lymphoma. Biochim Biophys Acta.

[109] Leary SC, Cobine PA, Nishimura T, Verdijk RM, de Krijger R, de Coo R, Tarnopolsky MA, Winge DR, Shoubridge EA (2013). COX19 mediates the transduction of a mitochondrial redox signal from SCO1 that regulates ATP7A-mediated cellular copper efflux. Mol Biol Cell.

[110] Bode M, Woellhaf MW, Bohnert M, van der Laan M, Sommer F, Jung M, Zimmermann R, Schroda M, Herrmann JM (2015). Redox-regulated dynamic interplay between Cox19 and the copper-binding protein Cox11 in the intermembrane space of mitochondria facilitates biogenesis of cytochrome c oxidase. Mol Biol Cell.

[111] Ishibashi M, Wakita T, Esumi M (2010). 2′,5′-Oligoadenylate synthetase-like gene highly induced by hepatitis C virus infection in human liver is inhibitory to viral replication in vitro. Biochem Biophys Res Commun.

[112] Marques J, Anwar J, Eskildsen-Larsen S, Rebouillat D, Paludan SR, Sen G, Williams BR, Hartmann R (2008). The p59 oligoadenylate synthetase-like protein possesses antiviral activity that requires the C-terminal ubiquitin-like domain. J Gen Virol.

[113] von Keyserling H, Kuhn W, Schneider A, Bergmann T, Kaufmann AM (2012). p16INK(4)a and p14ARF mRNA expression in Pap smears is age-related. Mod Pathol.

[114] Adachi H, Majima S, Kon S, Kobayashi T, Kajino K, Mitani H, Hirayama Y, Shiina H, Igawa M, Hino O (2004). Niban gene is commonly expressed in the renal tumors: a new candidate marker for renal carcinogenesis. Oncogene.

[115] Ito S, Fujii H, Matsumoto T, Abe M, Ikeda K, Hino O (2010). Frequent expression of Niban in head and neck squamous cell carcinoma and squamous dysplasia. Head Neck.

[116] Ji H, Ding Z, Hawke D, Xing D, Jiang BH, Mills GB, Lu Z (2012). AKT-dependent phosphorylation of Niban regulates nucleophosmin- and MDM2-mediated p53 stability and cell apoptosis. EMBO Rep.

[117] Sengupta D, Bhargava DK, Dixit A, Sahoo BS, Biswas S, Biswas G, Mishra SK (2014). ERRbeta signalling through FST and BCAS2 inhibits cellular proliferation in breast cancer cells. Br J Cancer.

[118] Gao X, Wei S, Lai K, Sheng J, Su J, Zhu J, Dong H, Hu H, Xu Z (2010). Nucleolar follistatin promotes cancer cell survival under glucose-deprived conditions through inhibiting cellular rRNA synthesis. J Biol Chem.

