# Supplementary material for: Systems-level effects of ectopic galectin-7 reconstitution in cervical cancer and its microenvironment
Source: BMC Cancer. 2016 Aug 24;16(1):680. doi: 10.1186/s12885-016-2700-8 (PMC4997669; doi:10.1186/s12885-016-2700-8)
Supplement: Additional file 1: — Additional Tables. Table S1. Genomic ranges of methylation sites within the Gal-7 gene obtained from TCGA-CESC data. Table S2. Primers used for the CpG methylation analysis by bisulfite-PCR-pyrosequencing. Table S3. Primers used for the quantification of genes commonly affected in HeLa and SiHa Gal-7+ cells. Table S4. Relevant cancer related genes and proteins differentially regulated in the HeLa Gal-7+ cell network. Table S5. Major Transcriptional factors identified in the HeLa Gal-7+ cell network. Table S6. Major Transcriptional factors identified in the SiHa Gal-7+ cell network. Table S7. Relevant cancer related genes and proteins differentially regulated in the SiHa Gal-7+ cell network. Table S8. Major Transcriptional factors identified in the HeLa Gal-7+ mouse microenvironment network. Table S9. Relevant differentially regulated genes identified in the HeLa Gal-7+ microenvironment network. Table S10. Major Transcriptional factors identified in the HeLa Gal-7+ mouse microenvironment network. Table S11. Relevant differentially regulated genes identified in the SiHa Gal-7+ microenvironment network. (DOCX 150 kb) [file 12885_2016_2700_MOESM1_ESM.docx]

**Systems-level effects of ectopic galectin-7 reconstitution in cervical cancer and its microenvironment**

**Higareda-Almaraz et al, 2016**

**Supplementary Tables**

**Table S1 Genomic ranges of methylation sites within the Gal-7 gene obtained from TCGA-CESC data (see Fig. 9D).** The area of methylated sites is depicted within the range of -499 to +100 relative to the initiation site. The genomic range of Gal-7 on chromosome 19 was 38,774,016-38,773-476

| Composite Element REF | Genomic ranges | Sequence |
| --- | --- | --- |
| cg07900259 | chr19:38,773,946-38,774,066 | AACCTCCTGCCTGGACTGATCCGTGCTGAAATCTAAGCTTCTGCCTCTGTAATGGAACAG**[CG]**GCCACAATAGGAGCACTAGTGACTGACAGGGTGGCTTCATGGGTTCACAGGGGGCCTGCC |
| cg03793778 | chr19:38,774,020-38,774,141 | GCACTAGTGACTGACAGGGTGGCTTCATGGGTTCACAGGGGGCCTGCCTGATCCCCTCAG**[CG]**CTGCACCCCTGGGTCCTGGAGTGTGCCCAGTTCATGGAAGTCCTCAGTCAGGAGGGGTGA |
| cg26776656 | chr19:38,774,356-38,774,476 | CACCTCTGCTCGCACCTCTCCTGTGGGTTCTGTCTCTGGGGCTGGCTGTCTCACTCTGTC**[CG]**TCTCTGCCCCTAACTCTGTCTACCACATATTCCTGTCCCACTTCTCTCCTTCAAGTCTCT |

**Table S2 Primers used for the CpG methylation analysis by bisulfite-PCR-pyrosequencing.** Primers in bold are biotinylated at the 5´-end. Sequencing primers (S1-S6) have been designed for forward sequencing

| **Amplicon**  **(Length)** | **PCR primer** | **Sequence (5’-3’)** | **Sequencing primer**  **5’-3’** | **Target CpGs**  **position (see Fig. 3B)** |
| --- | --- | --- | --- | --- |
| **Gal7 Promoter**  **(338 bp)** | Gal7met prom_Fw | AGGGTTTGGAGTTTTTATTTAGATTGG | **S**1: ATGAAATGAGATAGAGGTTT  **S**2: AGTTTTTAGGAGAGGG  **S**3: GTTTTTAAGAAGAGGTGTTATT | -294; -280;  -156;  -112;-109; -105; -74 |
|  | **Gal7met prom_bRw** | **ACCCACCACCCACACCTAA** |  |  |
| **Gal7 Ex_Int**  **(191 bp)** | Gal7met EI_Fw | AAGGGGTGGGGTGAGTTATT | **S**4: AGGTTTTTAGTAGGTTTTATTATT  **S**5: TGAGTGTTTTAGGGG | -12; +2; +18;  +37; +42 |
|  | **Gal7met EI_bRw** | **ACACACTCACTACCCACTTAC** |  |  |
|  | **PCR Primer** | **Sequence (5’-3’)** | **Sequencing Primer**  **5’-3’** | **Target CpGs**  **position (see Fig. 3B)** |
| **Gal7 Promoter**  **(338 bp)** | Gal7met prom_Fw | AGGGTTTGGAGTTTTTATTTAGATTGG | **S**1: ATGAAATGAGATAGAGGTTT  **S**2: AGTTTTTAGGAGAGGG  **S**3: GTTTTTAAGAAGAGGTGTTATT | -294; -280;  -156;  -112;-109; -105; -74 |
|  | **Gal7met prom_bRw** | **ACCCACCACCCACACCTAA** |  |  |
| **Gal7 Ex_Int**  **(253 bp)** | Gal7met EI_Fw | GTGGGGTGAGTTATTAGGGTTAGTT | **S**4: AGGTTTTTAGTAGGTTTTATTATT  **S**5: TGAGTGTTTTAGGGG  **S**6: TTGGTTTGGGTTGTG | -12; +2; +18;  +37; +42;  +132 |
|  | **Gal7met EI_bRw** | **CCACAAAAAAATAAAAAAAACAATC** |  |  |

| **Gene** | **Oligo sequence**  **(5´-3´)** |
| --- | --- |
| **hCRYAB-F728** | **GGGGTCCTCACTGTGAATGG** |
| **hCRYAB-R780** | **TTCACGGGTGATGGGAATGG** |
|  |  |
| **hFST-F402** | **GGATGGGAAAACCTACCGCA** |
| **hFST-R473** | **ACTTCCAGTTCTGGCTGCTC** |
|  |  |
| **hOASL-F598** | **TTCGTGAAACATCGGCCAAC** |
| **hOASL-R770** | **GGTGAAGCCTTCGTCCAACA** |
|  |  |
| **hCDKN2a-F4** | **AGAGGGGGAGAACAGACAAC** |
| **hCDKN2a-R175** | **ACCGTAACTATTCGGTGCGTT** |
|  |  |
| **hBCL3-F1534** | **TACACCCCCTCTTCTGAGCGT** |
| **hBCL3-R1628** | **CATTCCGGACAGGGACTGGT** |
|  |  |
| **hTACSTD2-F960** | **TCACCAACCGGAGAAAGTCG** |
| **hTACSTD2-R1118** | **AGGAAGCGTGACTCACTTGG** |
|  |  |
| **hFAM129A-F1355** | **CGCTGCATTCCGTGAAGATG** |
| **hFAM129A-R1637** | **TTCTTTCGGATGGTGCTGCT** |
|  |  |
| **hCOX19-F2126** | **AGTGTACCCAGGGTCGGG** |
| **hCOX19-R2209** | **ACCTTTGGTCGACGGTCTTC** |

**Table S3 Primers used for the quantification of genes commonly affected in HeLa and SiHa Gal-7+ cells.** Reserve and forward primers were designed for qPCR amplification

**Table S4 Relevant cancer related genes and proteins differentially regulated in the HeLa Gal-7+ cell network**

| Name | Molecule | FC | Module | Cancer Related Function | | Reference |
| --- | --- | --- | --- | --- | --- | --- |
| REG1A | Transcript | 4,55 | Cell cycle and proliferation | Related with better prognosis expression in esophageal squamous cell carcinoma patients related to negative patients | [1] | |
| RAP1GDS1 | Protein | 1,73 |  | Intermediary in the induction of apoptosis in Jurkat cells mediated by TG2 | [2] | |
| BRCA2 | Protein | 1,25 |  | Most important tumor suppressors and promotes genome stability | [3] | |
| NCCRP1 | Transcript | 2,97 |  | Silencing of NCCRP1 led to a decrement in HeLa cell proliferation | [4] | |
| RTEL1 | Protein | -1,43 |  | Amplification of RTEL1 promotes the carcinogenesis in hepatocellular carcinoma | [5] | |
| RAD9A | Protein | -1,97 |  | Responsible of radiation resistance in lung cancer cells | [6] | |
| CDKN2A/p14ARF | Protein | -1,92 |  | Is a hallmark of cervical cancer and is in fact used as a biomarker | [7] | |
| GRM3 | Transcript | -2,53 |  | High frequency mutated in melanoma, suggested like to be a driver | [8] | |
| RBPMS | Protein | -1,46 |  | Induced by a mutate version of HDAC2, its upregulation is related with carcinogenesis progress in colorectal cancer cells | [9] | |
| ARPC2 | Transcript | 2,39 | Cell migration & invasion | Depletion led to reduced migration of pancreatic cancer cells | [10] | |
| FGG | Transcript | 3,45 | Cell adhesion & tight junction | Inhibitor of Epithelial-Mesenchymal Transition, methylated in lung cancer | [11] | |
| TJP3 | Transcript | 2,51 |  | Tight junction protein, downregulated in gastric cancer | [12] | |
| CDH26 | Transcript | 2,86 |  | Adhesion protein, methylated in colon cancer | [13] | |
| PODXL2 | Protein | -1,79 |  | Overexpression associated with development of aggressive forms of prostate and breast cancer | [14, 15] | |
| ITGA6 | Protein | -1,55 |  | Crucial for the *in vitro* survival and proliferation of stem-cell-like breast cancer cells | [16] | |
| ITGA5 | Protein | -1,46 |  | Promote breast cancer metastasis, its upregulation correlates with poor survival prognosis | [17] | |

| TF Name | Cancer Related Function | Regulated Genes | Reference |
| --- | --- | --- | --- |
| SP9 | Atypical Sp1-like TF that is not well characterized yet | 35 |  |
| ARID3A | High expression correlated with good prognosis in colorectal carcinoma | 25 | [18] |
| MYF5 | Block the transformation of cells in mouse embryonic rhabdomyosarcoma | 16 | [19] |
| IKZF2 | Modulator of Ras and PI3K signaling in Acute Lymphoblastic Leukemia, inactivation drive tumor cell proliferation | 20 | [20] |
| SCRT2 | Prevents the re-entry into the cell cycle after mitosis in neurons | 15 | [21] |

**Table S5 Major Transcriptional factors identified in the HeLa Gal-7+ cell network**

**Table S6 Major Transcriptional factors identified in the SiHa Gal-7+ cell network**

| TF Name | Cancer Related Function | Regulated Genes | Reference |
| --- | --- | --- | --- |
| E2F1 | Promotes transcription of a big group of genes essential for cell cycle progression | 66 | [22] |
| E2F6 |  | 49 |  |
| EP300 | Histone acetyltransferase and regulates transcription via chromatin remodeling, BRCA is one of their effectors | 55 | [23] |
| SMAD1 | Diminished and delayed Smad phosphorylation and nuclear localization in CxCa | 92 | [24] |
| NFIC | NFIC-Ski interactions mediate the inhibition of the HPV16 URR by TGF-β | 56 | [25] |
| FOXJ3 | Regulator of cell growth, proliferation, differentiation, and longevity, his inhibition by MiR-517a-3p accelerates the proliferation and invasion of cancer cells | 63 | [26, 27] |

**Table S7 Relevant cancer related genes and proteins differentially regulated in the SiHa Gal-7+ cell network**

| Name | Molecule | FC | Module | Cancer Related Function | Reference |
| --- | --- | --- | --- | --- | --- |
| GAGE2E | Transcript | 2,34 | Immunity | Antigens frequently expressed in many different types of cancer, in normal tissues limited to germ cells. GAGE antigens are recognized by autologous cytolytic T-lymphocytes | [28] |
| GAGE12I | Transcript | 3,70 |  |  |  |
| GAGE2A | Transcript | 3,27 |  |  |  |
| GAGE12J | Transcript | 4,86 |  |  |  |
| GAGE2B | Transcript | 3,94 |  |  |  |
| GAGE12G | Transcript | 5,05 |  |  |  |
| GAGE5 | Transcript | 4,05 |  |  |  |
| GAGE4 | Transcript | 4,71 |  |  |  |
| GAGE12H | Transcript | 3,19 |  |  |  |
| GAGE12C | Transcript | 4,51 |  |  |  |
| GAGE12B | Transcript | 4,06 |  |  |  |
| GAGE6 | Transcript | 4,66 |  |  |  |
| IL-8 | Transcript | -3,47 |  | Proangiogenic cytokine overexpressed in human cancers, secreted by cervical carcinoma cells under hypoxia, stimulating autocrine viability supporting tumor microenvironment and treatment resistance | [29] |
| EPCAM | Protein | 2,07 | Cell adhesion and tight junction | Related to cancer progression, metastasis, and poor prognosis in Ovarian and Brest cancer | [30, 31] |
| CADH1 | Protein | 1,40 |  | Marker of better prognosis in ovarian serous carcinoma | [32] |
| TNFAIP6 | Transcript | -2,32 |  | Related with the control of the inflammatory response and is overexpressed in ovarian cancer | [33] |
| VTN | Transcript | -2,47 |  | Favors *in vitro* migration of diverse cancer cells | [34] |
| MUC16 | Transcript | -2,66 |  | Overexpression related with poor prognosis in digestive-tract adenocarcinoma | [35] |
| KAL1 | Transcript | 2,42 |  | Potential tumor-suppressive role in oral carcinoma | [36] |
| CLD8 | Transcript | 3,59 |  | Frequently downregulated in colorectal cancer, downregulation and cytoplasmic localization is related to a bad prognosis in renal cancer | [37, 38] |
| PLAC8 | Transcript | 2,44 | Regulation of programmed cell death | Its expression in the female tract is necessary to control infections, and is regulator of the autophagic response during pancreatic cancer progression | [39, 40] |
| TCN1 | Transcript | -4,47 | Metabolism and transport | Enhance the expression of downstream genes of the Wnt/beta-catenin pathway, promoting cancer progression in gastric cancer | [41] |

**Table S8 Major Transcriptional factors identified in the HeLa Gal-7+ mouse microenvironment network**

| TF Name | Cancer Related Function | Regulated Genes | Reference |
| --- | --- | --- | --- |
| Arfgap1 | Their loss of function is related to genome instability and carcinogenesis in several models | 44 | [42] |
| Egr1 | Responsive element of Srf network | 40 | [43] |
| Srf | Increments in the SRF can promote carcinogenesis and metastasis in gastric and prostate cancer | 116 | [44, 45] |

**Table S9 Relevant differentially regulated genes identified in the HeLa Gal-7+ microenvironment network**

| Name | FC | Module | Cancer Related Function | Reference |
| --- | --- | --- | --- | --- |
| Ccl3 | 3,83 | Chemokine activity and immune response | Immature dendritic cells (iDC)-attracting chemokines, favoring immune response | [46] |
| Ccl4 | 3,46 |  |  |  |
| CxCl2 | 3,49 |  | Expression can contribute to cause chemoresistance and metastasis in breast cancer | [47] |
| CxCl13 | 3,94 |  | Responsible for B-cell recruitment into the tumor remnant, leading to accelerated malignant progression | [48] |
| IL1β | 4,24 |  | Cytokines, inductors of the Ccl and CxCl chemokines | [49] |
| Areg | 4,36 |  | Is overexpressed in EGFR positive microenvironment in colon cancer xenograft tumors | [50] |
| Retnlg | 3,88 |  | Is overexpressed in response to NFATc1 in skin cancer | [49] |
| S100A8 | 6,07 |  | Neutrophil stimulation and migration to inflammatory sites | [51] |
| S100A9 | 5,78 |  |  |  |
| Saa3 | 3,83 |  | Activator of the acute phase response in inflammation | [52] |
| Arg1 | 3,95 |  | Upregulation is a hallmark of macrophage activation and infiltration | [53] |
| Ear2 | 3,70 |  | Transcriptional repressor of IL-17 expression in Th-17 differentiated CD4+ T cells | [54] |
| Sp7 | -4,90 | Regulation of transcription | Marker for bone progenitor cells and is related to the expression of chemokines in the microenvironment | [55] |
| H19 | -4,14 |  | LncRNA that promotes tumorigenesis in murine mesenchymal cells, overexpressed in gastric and bladder cancer | [56-58] |
| Abra | -3,81 |  | Initiates SRF-dependent transcription | [59] |
| Zymnd19 | 4,05 |  | High expression in hematopoietic stem cells and in multi-lineage progenitor cells | [60] |
| Actc1 | -4,28 | Tissue morphogenesis | Responsive genes to Srf and Egr1 | [61] |
| Actn2 | -4,75 |  |  |  |
| Actn3 | -4,35 |  |  |  |
| Myf6 | -3,83 |  |  |  |
| Myh1 | -5,38 |  |  |  |
| Myh2 | -4,05 |  |  |  |
| Myh8 | -4,10 |  |  |  |
| Myl1 | -4,19 |  |  |  |
| Myl4 | -4,55 |  |  |  |
| Mylk2 | -5,15 |  |  |  |
| Myom1 | -5,22 |  |  |  |
| Myom2 | -5,31 |  |  |  |
| Myom3 | -4,83 |  |  |  |
| Trim72 | -4,75 |  | Overexpression induce wound healing in vitro in lung cells | [62] |
| Ttn | -6,04 |  | Can act like a "mini-drive" gene in breast cancer | [63] |
| Galnt9 | -5,24 | Metabolism and transport | Overexpressed in neuroblastoma patients | [64] |

**Table S10 Major Transcriptional factors identified in the HeLa Gal-7+ mouse microenvironment network**

| TF Name | Cancer Related Function | Regulated Genes | Reference |
| --- | --- | --- | --- |
| Trp53 | Inactivation in melanoma model exacerbates immunosuppression, expanding lymphoid-like stromal network, enhancing tumor progression | 29 | [65] |
| Nr2c2 | Tumor suppressor that suppresses PCa initiation through promoting DNA repair and maintaining genome integrity | 42 | [66] |
| Mecom | Oncogenic transcription factor in murine and human myeloid leukemia, regulate the hematopoietic stem cell pool hierarchically | 34 | [67] |

**Table S11 Relevant differentially regulated genes identified in the SiHa Gal-7+ microenvironment network**

| Name | FC | Module | Cancer Related Function | Reference |
| --- | --- | --- | --- | --- |
| Ccl5 | -3,75 | Chemokine activity and immune response | Predominant role in cancer progression, metastasis and inflammation-promoting carcinogenesis | [68-70] |
| Cxcl9 | -4,26 |  | Related to tumor progression and disease-free survival in oral cavity squamous carcinoma | [71] |
| Cxcl10 | -2,49 |  | Promotes bone metastasis of different cancer cells | [72] |
| Zmiz1 | -4,47 | Regulation of transcription | Implicated in cutaneous cancer induction in mouse | [73] |
| Ncor1 | -2,84 |  | Related with cancer development | [74] |
| Tfe3 | -2,69 |  | Overexpression shows aggressive behavior in renal carcinomas | [75] |
| Taf15 | 2,73 |  | Contradictory information | [76, 77] |

**Supplementary references**

1. Sato Y, Motoyama S, Nanjo H, Ito S, Yoshino K, Sasaki T, Kuribayashi K, Nagaki Y, Imai K, Saito H *et al*: **REG1A expression status suggests chemosensitivity among advanced thoracic esophageal squamous cell carcinoma patients treated with esophagectomy followed by adjuvant chemotherapy**. *Annals of surgical oncology* 2013, **20**(9):3044-3051.

2. Hsieh YF, Liu GY, Lee YJ, Yang JJ, Sandor K, Sarang Z, Bononi A, Pinton P, Tretter L, Szondy Z *et al*: **Transglutaminase 2 contributes to apoptosis induction in Jurkat T cells by modulating Ca2+ homeostasis via cross-linking RAP1GDS1**. *PloS one* 2013, **8**(12):e81516.

3. Yata K, Bleuyard JY, Nakato R, Ralf C, Katou Y, Schwab RA, Niedzwiedz W, Shirahige K, Esashi F: **BRCA2 coordinates the activities of cell-cycle kinases to promote genome stability**. *Cell reports* 2014, **7**(5):1547-1559.

4. Kallio HT, M.; Jänis, J.; Pan, P. W.; Laurila, E.; Kallioniemi, A.; Kilpinen, S.; Tuominen, V. J.; Isola, J.; Valjakka, J.; Pastorekova, S.; Pastorek, J. and Parkkila, S.: **Characterization of Non-Specific Cytotoxic Cell Receptor Protein 1: A New Member of the Lectin-Type Subfamily of F-Box Proteins**. *PloS one* 2011, **6**(11):e27152.

5. Wu XS, S.; Nabi, Z. and Ding, H.: **Generation of a mouse model for studying the role of upregulated RTEL1 activity in tumorigenesis**. *Transgenic Res* 2012, **21**(5):1109-1115.

6. Guo W. F.; Lin RXH, J.; Zhou, Z.; Yang, J.; Guo, G. Z. and Wang, S. Q.: **Identification of Differentially Expressed Genes Contributing to Radioresistance in Lung Cancer Cells using Microarray Analysis**. *Radiat Res* 2005, **164**(1):27-35.

7. von Keyserling HKWS, A.; Bergmann, T. and Kaufmann, A. M.: **p16INK⁴a and p14ARF mRNA expression in Pap smears is age-related**. *Modern pathology : an official journal of the United States and Canadian Academy of Pathology, Inc* 2012, **25**(3):465-470.

8. Prickett TDW, X.; Cardenas-Navia, I.; Teer, J. K.; Lin, J. C.; Walia, V.; Gartner, J.; Jiang, J.; Cherukuri, P. F.; Molinolo, A.; Davies, M. A.; Gershenwald, J. E.; Stemke-Hale, K.; Rosenberg, S. A.; Margulies, E. H. and Samuels, Y.: **Exon capture analysis of G protein-coupled receptors identifies activating mutations in GRM3 in melanoma**. *Nature genetics* 2011, **43**(11):1119-1126.

9. Ropero SB, E.; Alaminos, M.; Arango, D.; Schwartz, S. Jr. and Esteller, M.: **Transforming pathways unleashed by a HDAC2 mutation in human cancer**. *Oncogene* 2008, **27**(28):4008-4012.

10. Rauhala HET, S.; Niemelä, S. and Kallioniemi, A.: **Silencing of the ARP2/3 complex disturbs pancreatic cancer cell migration**. *Anticancer Res* 2013, **33**(1):45-52.

11. Wang HM, C. A.; Fei T.; Wang, G.; Zhang, F.; Liu, X. S.: **A systematic approach identifies FOXA1 as a key factor in the loss of epithelial traits during the epithelial-to-mesenchymal transition in lung cancer**. *BMC Genomics* 2013, **14**(680).

12. Qu Y, Ray PS, Li J, Cai Q, Bagaria SP, Moran C, Sim MS, Zhang J, Turner RR, Zhu Z *et al*: **High levels of secreted frizzled-related protein 1 correlate with poor prognosis and promote tumourigenesis in gastric cancer**. *Eur J Cancer* 2013, **49**(17):3718-3728.

13. Ghoshal K, Motiwala T, Claus R, Yan P, Kutay H, Datta J, Majumder S, Bai S, Majumder A, Huang T *et al*: **HOXB13, a target of DNMT3B, is methylated at an upstream CpG island, and functions as a tumor suppressor in primary colorectal tumors**. *PloS one* 2010, **5**(4):e10338.

14. Casey G, Neville PJ, Liu X, Plummer SJ, Cicek MS, Krumroy LM, Curran AP, McGreevy MR, Catalona WJ, Klein EA *et al*: **Podocalyxin variants and risk of prostate cancer and tumor aggressiveness**. *Human molecular genetics* 2006, **15**(5):735-741.

15. Sizemore S, Cicek M, Sizemore N, Ng KP, Casey G: **Podocalyxin increases the aggressive phenotype of breast and prostate cancer cells in vitro through its interaction with ezrin**. *Cancer research* 2007, **67**(13):6183-6191.

16. Cariati M, Naderi A, Brown JP, Smalley MJ, Pinder SE, Caldas C, Purushotham AD: **Alpha-6 integrin is necessary for the tumourigenicity of a stem cell-like subpopulation within the MCF7 breast cancer cell line**. *International journal of cancer Journal international du cancer* 2008, **122**(2):298-304.

17. Qin L, Chen X, Wu Y, Feng Z, He T, Wang L, Liao L, Xu J: **Steroid receptor coactivator-1 upregulates integrin alpha(5) expression to promote breast cancer cell adhesion and migration**. *Cancer research* 2011, **71**(5):1742-1751.

18. Song M, Kim H, Kim WK, Hong SP, Lee C, Kim H: **High expression of AT-rich interactive domain 3A (ARID3A) is associated with good prognosis in colorectal carcinoma**. *Annals of surgical oncology* 2014, **21 Suppl 4**:S481-489.

19. Nitzki F, Zibat A, Frommhold A, Schneider A, Schulz-Schaeffer W, Braun T, Hahn H: **Uncommitted precursor cells might contribute to increased incidence of embryonal rhabdomyosarcoma in heterozygous Patched1-mutant mice**. *Oncogene* 2011, **30**(43):4428-4436.

20. Holmfeldt L, Wei L, Diaz-Flores E, Walsh M, Zhang J, Ding L, Payne-Turner D, Churchman M, Andersson A, Chen SC *et al*: **The genomic landscape of hypodiploid acute lymphoblastic leukemia**. *Nature genetics* 2013, **45**(3):242-252.

21. Rodriguez-Aznar E, Barrallo-Gimeno A, Nieto MA: **Scratch2 prevents cell cycle re-entry by repressing miR-25 in postmitotic primary neurons**. *The Journal of neuroscience : the official journal of the Society for Neuroscience* 2013, **33**(12):5095-5105.

22. Münger K, Phelps WC, Bubb V, Howley PM, Schlegel R: **The E6 and E7 genes of the human papillomavirus type 16 together are necessary and sufficient for transformation of primary human keratinocytes**. *J Virol* 1989, **63**(10):4417-4421.

23. Raychaudhuri S, Loew C, Korner R, Pinkert S, Theis M, Hayer-Hartl M, Buchholz F, Hartl FU: **Interplay of Acetyltransferase EP300 and the Proteasome System in Regulating Heat Shock Transcription Factor 1**. *Cell* 2014, **156**(5):975-985.

24. Altomare DV, R.; Pirisi, L. and Creek, K. E.: **Partial loss of Smad signaling during in vitro progression of HPV16-immortalized human keratinocytes**. *BMC cancer* 2013, **13**(424).

25. Baldwin A, Pirisi L, Creek KE: **NFI-Ski Interactions Mediate Transforming Growth Factor   Modulation of Human Papillomavirus Type 16 Early Gene Expression**. *Journal of virology* 2004, **78**(8):3953-3964.

26. Landgren H, Carlsson P: **FoxJ3, a novel mammalian forkhead gene expressed in neuroectoderm, neural crest, and myotome**. *Developmental dynamics : an official publication of the American Association of Anatomists* 2004, **231**(2):396-401.

27. Jin J, Zhou S, Li C, Xu R, Zu L, You J, Zhang B: **MiR-517a-3p accelerates lung cancer cell proliferation and invasion through inhibiting FOXJ3 expression**. *Life sciences* 2014, **108**(1):48-53.

28. Gjerstorff MF, Ditzel HJ: **An overview of the GAGE cancer/testis antigen family with the inclusion of newly identified members**. *Tissue antigens* 2008, **71**(3):187-192.

29. Stone SC, Rossetti RA, Lima AM, Lepique AP: **HPV associated tumor cells control tumor microenvironment and leukocytosis in experimental models**. *Immunity, inflammation and disease* 2014, **2**(2):63-75.

30. Spizzo G, Went P, Dirnhofer S, Obrist P, Moch H, Baeuerle PA, Mueller-Holzner E, Marth C, Gastl G, Zeimet AG: **Overexpression of epithelial cell adhesion molecule (Ep-CAM) is an independent prognostic marker for reduced survival of patients with epithelial ovarian cancer**. *Gynecologic oncology* 2006, **103**(2):483-488.

31. Osta WAC, Y.; Mikhitarian, K.; Mitas, M.; Salem, M.; Hannun, Y. A.; Cole, D. J.; and Gillanders W. E.: **EpCAM Is Overexpressed in Breast Cancer and Is a Potential Target for Breast Cancer Gene Therapy**. *Cancer research* 2004, **64**(16):5818–5824.

32. Shim HS, Yoon BS, Cho NH: **Prognostic significance of paired epithelial cell adhesion molecule and E-cadherin in ovarian serous carcinoma**. *Human pathology* 2009, **40**(5):693-698.

33. Sasaroli DG, P. A.; Pathak, H. B.; Hammond, R.; Kougioumtzidou, E.; Katsaros, D.; Buckanovich, R.; Devarajan, K.; Sandaltzopoulos, R.; Godwin, A. K.; Scholler, N. and Coukos, G.: **Novel surface targets and serum biomarkers from the ovarian cancer vasculature**. *Cancer Biology & Therapy* 2014, **12**(3):169-180.

34. Rea VE, Lavecchia A, Di Giovanni C, Rossi FW, Gorrasi A, Pesapane A, de Paulis A, Ragno P, Montuori N: **Discovery of new small molecules targeting the vitronectin-binding site of the urokinase receptor that block cancer cell invasion**. *Molecular cancer therapeutics* 2013, **12**(8):1402-1416.

35. Streppel MM, Vincent A, Mukherjee R, Campbell NR, Chen SH, Konstantopoulos K, Goggins MG, Van Seuningen I, Maitra A, Montgomery EA: **Mucin 16 (cancer antigen 125) expression in human tissues and cell lines and correlation with clinical outcome in adenocarcinomas of the pancreas, esophagus, stomach, and colon**. *Human pathology* 2012, **43**(10):1755-1763.

36. Liu J, Cao W, Chen W, Xu L, Zhang C: **Decreased expression of Kallmann syndrome 1 sequence gene (KAL1) contributes to oral squamous cell carcinoma progression and significantly correlates with poorly differentiated grade**. *Journal of oral pathology & medicine : official publication of the International Association of Oral Pathologists and the American Academy of Oral Pathology* 2014.

37. Grone J, Weber B, Staub E, Heinze M, Klaman I, Pilarsky C, Hermann K, Castanos-Velez E, Ropcke S, Mann B *et al*: **Differential expression of genes encoding tight junction proteins in colorectal cancer: frequent dysregulation of claudin-1, -8 and -12**. *International journal of colorectal disease* 2007, **22**(6):651-659.

38. Osunkoya AO, Cohen C, Lawson D, Picken MM, Amin MB, Young AN: **Claudin-7 and claudin-8: immunohistochemical markers for the differential diagnosis of chromophobe renal cell carcinoma and renal oncocytoma**. *Human pathology* 2009, **40**(2):206-210.

39. Johnson RM, Kerr MS, Slaven JE: **Plac8-dependent and inducible NO synthase-dependent mechanisms clear Chlamydia muridarum infections from the genital tract**. *Journal of immunology* 2012, **188**(4):1896-1904.

40. Kinsey C, Balakrishnan V, O'Dell MR, Huang JL, Newman L, Whitney-Miller CL, Hezel AF, Land H: **Plac8 links oncogenic mutations to regulation of autophagy and is critical to pancreatic cancer progression**. *Cell reports* 2014, **7**(4):1143-1155.

41. Kim B, Koo H, Yang S, Bang S, Jung Y, Kim Y, Kim J, Park J, Moon RT, Song K *et al*: **TC1(C8orf4) correlates with Wnt/beta-catenin target genes and aggressive biological behavior in gastric cancer**. *Clin Cancer Res* 2006, **12**(11 Pt 1):3541-3548.

42. Sangar FS, A. S.; Umaña-Diaz, C.; Clapéron, A.; Desbois-Mouthon, C.; Calmel, C.; Mauger, O.; Zaanan, A.; Miquel, C.; Fléjou, J. F. and Praz, F.: **Involvement of small ArfGAP1 (SMAP1), a novel Arf6-specific GTPase-activating protein, in microsatellite instability oncogenesis**. *Oncogene* 2014, **33**(21):2758-2767.

43. Kim MJ, Kang JH, Chang SY, Jang HJ, Ryu GR, Ko SH, Jeong IK, Kim MS, Jo YH: **Exendin-4 induction of Egr-1 expression in INS-1 beta-cells: interaction of SRF, not YY1, with SRE site of rat Egr-1 promoter**. *Journal of cellular biochemistry* 2008, **104**(6):2261-2271.

44. Zhao XH, L.; Li, T.; Lu, Y.; Miao, Y.; Liang, S.; Guo, H.; Bai, M.; Xie, H.; Luo, G.; Zhou, L.; Shen, G.; Guo, C.; Bai, F.; Sun, S.; Wu, K.; Nie, Y. and Fan, D.: **SRF expedites metastasis and modulates the epithelial to mesenchymal transition by regulating miR-199a-5p expression in human gastric cancer**. *Cell Death Differ* 2014, **21**(12):1900-1913.

45. Verone ARD, K.; Godoy, A.; Yadav, N.; Bakin, A.; Koochekpour, S.; Jin, J. P. and Heemers, H. V.: **Androgen-responsive serum response factor target genes regulate prostate cancer cell migration**. *Carcinogenesis* 2013, **34**(8):1737-1746.

46. Wong LB, E.; Edwards, P. and Kalinski, P.: **IL-18–Primed Helper NK Cells Collaborate with Dendritic Cells to Promote Recruitment of Effector CD8+ T Cells to the Tumor Microenvironment**. *Cancer research* 2013, **73**(15):4653-4662.

47. Acharyya SO, T.; Vanharanta, S.; Malladi, S.; Kim, J.; Morris, PG.; Manova-Todorova, K.; Leversha, M.; Hogg, N.; Seshan, V. E.; Norton, L.; Brogi, E. and Massagué, J.: **A CXCL1 Paracrine Network Links Cancer Chemoresistance and Metastasis**. *Cell* 2012, **150**(1):165-178.

48. Ammirante MS, S.; Kang, Y.; Jamieson, C. A. and Karin, M.: **Tissue injury and hypoxia promote malignant progression of prostate cancer by inducing CXCL13 expression in tumor myofibroblasts**. *Proceedings of the National Academy of Sciences of the United States of America* 2014, **111**(41):14776-14781.

49. Tripathi P, Wang Y, Coussens M, Manda KR, Casey AM, Lin C, Poyo E, Pfeifer JD, Basappa N, Bates CM *et al*: **Activation of NFAT signaling establishes a tumorigenic microenvironment through cell autonomous and non-cell autonomous mechanisms**. *Oncogene* 2014, **33**(14):1840-1849.

50. Mustafi R, Dougherty U, Shah H, Dehghan H, Gliksberg A, Wu J, Zhu H, Joseph L, Hart J, Dive C *et al*: **Both stromal cell and colonocyte epidermal growth factor receptors control HCT116 colon cancer cell growth in tumor xenografts**. *Carcinogenesis* 2012, **33**(10):1930-1939.

51. Ryckman C, Vandal K, Rouleau P, Talbot M, Tessier PA: **Proinflammatory Activities of S100: Proteins S100A8, S100A9, and S100A8/A9 Induce Neutrophil Chemotaxis and Adhesion**. *The Journal of Immunology* 2003, **170**(6):3233-3242.

52. Faty AF, P. and Commans, S.: **The Acute Phase Protein Serum Amyloid A Induces Lipolysis and Inflammation in Human Adipocytes through Distinct Pathways**. *PloS one* 2012, **7**(4):e34031.

53. Pesce JT, Ramalingam TR, Mentink-Kane MM, Wilson MS, El Kasmi KC, Smith AM, Thompson RW, Cheever AW, Murray PJ, Wynn TA: **Arginase-1-expressing macrophages suppress Th2 cytokine-driven inflammation and fibrosis**. *PLoS pathogens* 2009, **5**(4):e1000371.

54. Hermann-Kleiter N, Gruber T, Lutz-Nicoladoni C, Thuille N, Fresser F, Labi V, Schiefermeier N, Warnecke M, Huber L, Villunger A *et al*: **The nuclear orphan receptor NR2F6 suppresses lymphocyte activation and T helper 17-dependent autoimmunity**. *Immunity* 2008, **29**(2):205-216.

55. Liu Y, Strecker S, Wang L, Kronenberg MS, Wang W, Rowe DW, Maye P: **Osterix-cre labeled progenitor cells contribute to the formation and maintenance of the bone marrow stroma**. *PloS one* 2013, **8**(8):e71318.

56. Shoshani O, Massalha H, Shani N, Kagan S, Ravid O, Madar S, Trakhtenbrot L, Leshkowitz D, Rechavi G, Zipori D: **Polyploidization of murine mesenchymal cells is associated with suppression of the long noncoding RNA H19 and reduced tumorigenicity**. *Cancer research* 2012, **72**(24):6403-6413.

57. Zhang EB, Han L, Yin DD, Kong R, De W, Chen J: **c-Myc-induced, long, noncoding H19 affects cell proliferation and predicts a poor prognosis in patients with gastric cancer**. *Medical oncology* 2014, **31**(5):914.

58. Luo M, Li Z, Wang W, Zeng Y, Liu Z, Qiu J: **Upregulated H19 contributes to bladder cancer cell proliferation by regulating ID2 expression**. *The FEBS journal* 2013, **280**(7):1709-1716.

59. Arai A, Spencer JA, Olson EN: **STARS, a striated muscle activator of Rho signaling and serum response factor-dependent transcription**. *The Journal of biological chemistry* 2002, **277**(27):24453-24459.

60. Shay TaK, J.: **Immunological Genome Project and systems immunology**. *Trends in immunology* 2013, **34**(12):602-609.

61. Luxenburg C, Pasolli HA, Williams SE, Fuchs E: **Developmental roles for Srf, cortical cytoskeleton and cell shape in epidermal spindle orientation**. *Nature cell biology* 2011, **13**(3):203-214.

62. Kim CK, T.; Wang, S.; Nishi, M.; Nagre, N.; Zhou, B.; Flodby, P.; Shilo, K.; Ghadiali, S. N.; Takeshima, H.; Hubmayr, R. D. and Zhao, X.: **TRIM72 is required for effective repair of alveolar epithelial cell wounding**. *Am J Physiol Lung Cell Mol Physiol* 2014, **307**(6):L449-L459.

63. Ostrow SLB, R.; DeGregori, J.; Yeger-Lotem, E. and Hershberg R.: **Cancer Evolution Is Associated with Pervasive Positive Selection on Globally Expressed Genes**. *Plos Genet* 2014, **10**(3).

64. Berois N, Gattolliat CH, Barrios E, Capandeguy L, Douc-Rasy S, Valteau-Couanet D, Benard J, Osinaga E: **GALNT9 gene expression is a prognostic marker in neuroblastoma patients**. *Clinical chemistry* 2013, **59**(1):225-233.

65. Guo GM, L.; Rodriguez, P.; Del Valle, L.; Ochoa, A.; and Cui, Y.: **Trp53 Inactivation in the Tumor Microenvironment Promotes Tumor Progression by Expanding the Immunosuppressive Lymphoid-like Stromal Network**. *Cancer research* 2013, **73**(6):1668-1675.

66. Lin JL, S. O.; Lee, Y. F.; Miyamoto, H.; Yang, D. R.; Li, G. and Chang, C.: **TR4 nuclear receptor functions as a tumor suppressor for prostate tumorigenesis via modulation of DNA damage/repair system.** *Carcinogenesis* 2014, **35**(6):1399-1406.

67. Yuasa HO, Y.; Iwama, A.; Nishikata, I.; Sugiyama, D.; Perkins, A.; Mucenski, M. L.; Suda, T. and Morishita, K.: **Oncogenic transcription factor Evi1 regulates hematopoietic stem cell proliferation through GATA-2 expression**. *EMBO J* 2005, **24**(11):1976-1987.

68. Luboshits GS, S.; Kaplan, O.; Engelberg, S.; Nass, D.; Lifshitz-Mercer, B.; Chaitchik, S.; Keydar, I.; and Ben-Baruch, A.: **Elevated Expression of the CC Chemokine Regulated on Activation, Normal T Cell Expressed and Secreted (RANTES) in Advanced Breast Carcinoma**. *Cancer research* 1999(59):4681–4687.

69. Mi Z, Bhattacharya SD, Kim VM, Guo H, Talbot LJ, Kuo PC: **Osteopontin promotes CCL5-mesenchymal stromal cell-mediated breast cancer metastasis**. *Carcinogenesis* 2011, **32**(4):477-487.

70. Yi EH, Lee CS, Lee JK, Lee YJ, Shin MK, Cho CH, Kang KW, Lee JW, Han W, Noh DY *et al*: **STAT3-RANTES autocrine signaling is essential for tamoxifen resistance in human breast cancer cells**. *Molecular cancer research : MCR* 2013, **11**(1):31-42.

71. Chang KP, Wu CC, Fang KH, Tsai CY, Chang YL, Liu SC, Kao HK: **Serum levels of chemokine (C-X-C motif) ligand 9 (CXCL9) are associated with tumor progression and treatment outcome in patients with oral cavity squamous cell carcinoma**. *Oral oncology* 2013, **49**(8):802-807.

72. Lee JH, Kim HN, Kim KO, Jin WJ, Lee S, Kim HH, Ha H, Lee ZH: **CXCL10 promotes osteolytic bone metastasis by enhancing cancer outgrowth and osteoclastogenesis**. *Cancer research* 2012, **72**(13):3175-3186.

73. Rogers LM, Riordan JD, Swick BL, Meyerholz DK, Dupuy AJ: **Ectopic expression of Zmiz1 induces cutaneous squamous cell malignancies in a mouse model of cancer**. *The Journal of investigative dermatology* 2013, **133**(7):1863-1869.

74. Doig CL, Singh PK, Dhiman VK, Thorne JL, Battaglia S, Sobolewski M, Maguire O, O'Neill LP, Turner BM, McCabe CJ *et al*: **Recruitment of NCOR1 to VDR target genes is enhanced in prostate cancer cells and associates with altered DNA methylation patterns**. *Carcinogenesis* 2013, **34**(2):248-256.

75. Macher-Goeppinger S, Roth W, Wagener N, Hohenfellner M, Penzel R, Haferkamp A, Schirmacher P, Aulmann S: **Molecular heterogeneity of TFE3 activation in renal cell carcinomas**. *Modern pathology : an official journal of the United States and Canadian Academy of Pathology, Inc* 2012, **25**(2):308-315.

76. Ballarino M, Jobert L, Dembele D, de la Grange P, Auboeuf D, Tora L: **TAF15 is important for cellular proliferation and regulates the expression of a subset of cell cycle genes through miRNAs**. *Oncogene* 2013, **32**(39):4646-4655.

77. Martini ALS, R.; Janssen, H.; Bilhou-Nabera, C.; Corveleyn, A.; Somers, R.; Aventin, A.; Foa, R.; Hagemeijer, A.; Mecucci, C.; Marynen, P.: **Recurrent Rearrangement of the Ewing’s Sarcoma Gene, EWSR1, or Its Homologue, TAF15, with the Transcription Factor CIZ-NMP4 in Acute Leukemia**. *Cancer research* 2002(62):5408–5412.
