# Supplementary material for: Systems-level effects of ectopic galectin-7 reconstitution in cervical cancer and its microenvironment
Source: BMC Cancer. 2016 Aug 24;16(1):680. doi: 10.1186/s12885-016-2700-8 (PMC4997669; doi:10.1186/s12885-016-2700-8)

Systems-level effects of ectopic galectin-7 reconstitution in cervical cancer and its microenvironment

Higareda-Almaraz , *et al.* 2016

- **Supplementary file 1. Ms/Ms identification and SILAC quantification spectra of representative peptides of the common proteins to HeLa Gal-7+ and SiHa Gal-7+ CxCa cell lines.**

HeLa VS HeLa Gal-7+ Cell lines

B-cell lymphoma 3 protein OS=Homo sapiens GN=BCL3 PE=1 SV=2 - [BCL3_HUMAN]

Identified peptides:

QTPLHLAVITTLPSVVR
LLVTAGASPMALDR
CPAGAMDEGPVDLR
NCHNDTPLMVAR
HGQTAAHLACEHR
ELDIYNNLR
VIDILR
GPGRPVPPSPAPGGS
GLLPLVR

Coverage | ProteinCard

B-cell lymphoma 3 protein OS=Homo sapiens GN=BCL3 PE=1 SV=2 - [BCL3_HUMAN]

☐ Annotate PTMs reported in Uniprot

☐ Show only PTMs

☐ Include PSMs that are filtered Out

Coverage: 23.57%

Found Modifications:

C Carbamidomethyl (C)
L Label:13C(6)15N(4) (R)

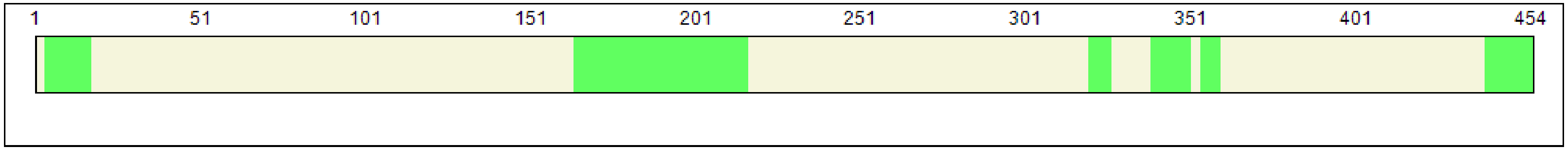

Sequence | Modification List

	1	11	21	31	41	51	61	71	81	91	101
1	MPR**CPAGAMD** (C)	**EGPVDLR**TRP	KAAGLPGAAL	PLRKRPLRAP	SPEPAAPRGA	AGLVVPLDPL	RGGCDLPAVP	GPPHGLARPE	ALYYPGALLP	LYPTRAMGSP	FPLVNLPTPL
111	YPMMCPMEHP	LSADIAMATR	ADEDGDTPLH	IAVVQGNLPA	VHRLVNLFQQ	GGR**ELDIYNN**	**LRQTPLHLAV**	**ITTLPSVVRL** (L)	**LVTAGASPMA**	**LDRHGQTAAH**	**LACEHR**SPTC (C)
221	LRALLDSAAP	GTLDLEARNY	DGLTALHVAV	NTECQETVQL	LLERGADIDA	VDIKSGRSPL	IHAVENNSLS	MVQLLLQHGA	NVNAQMYSGS	SALHSASGR**G**	**LLPLVR**TLVR (L)
331	SGADSSLK**NC** (C)	**HNDTPLMVAR**	SRR**VIDILR**G (L)	KATRPASTSQ	PDPSPDRSAN	TSPESSSRLS	SNGLLSASPS	SSPSQSPPRD	PPGFPMAPPN	FFLPSPSPPA	FLPFAGVLR**G**
441	**PGRPVPPSPA**	**PGGS**									

Sequence: QTPLHLAVITTLPSVVR, R17-Label:13C(6)15N(4) (10.00827 Da)
Charge: +3, Monoisotopic m/z: 619.03851 Da (-1 mmu/-1.62 ppm), MH+: 1855.10099 Da, RT: 42.78 min,
Identified with: Mascot (v1.30); IonScore:35, Exp Value:1.7E-002, Ions matched by search engine: 8/184
Fragment match tolerance used for search: 0.02 Da

#1	b^{+}	b^{2+}	b^{3+}	Seq.	y^{+}	y^{2+}	y^{3+}	#2
1	129.06586	65.03657	43.69347	Q				17
2	230.11354	115.56041	77.37603	T	1727.04541	864.02634	576.35332	16
3	327.16631	164.08679	109.72695	P	1625.99773	813.50250	542.67076	15
4	440.25038	220.62883	147.42164	L	1528.94496	764.97612	510.31984	14
5	577.30929	289.15828	193.10795	H	1415.86089	708.43408	472.62515	13
6	690.39336	345.70032	230.80264	L	1278.80198	639.90463	426.93884	12
7	761.43048	381.21888	254.48168	A	1165.71791	583.36259	389.24415	11
8	860.49890	430.75309	287.50448	V	1094.68079	547.84403	365.56511	10
9	973.58297	487.29512	325.19917	I	995.61237	498.30982	332.54231	9
10	1074.63065	537.81896	358.88173	T	882.52830	441.76779	294.84762	8
11	1175.67833	588.34280	392.56429	T	781.48062	391.24395	261.16506	7
12	1288.76240	644.88484	430.25898	L	680.43294	340.72011	227.48250	6
13	1385.81517	693.41122	462.60991	P	567.34887	284.17807	189.78781	5
14	1472.84720	736.92724	491.62058	S	470.29610	235.65169	157.43688	4
15	1571.91562	786.46145	524.64339	V	383.26407	192.13567	128.42621	3
16	1670.98404	835.99566	557.66620	V	284.19565	142.60146	95.40340	2
17				R-Label:13C(6)15N(4)	185.12723	93.06725	62.38059	1

QTPLHLAVITTLPSVVR

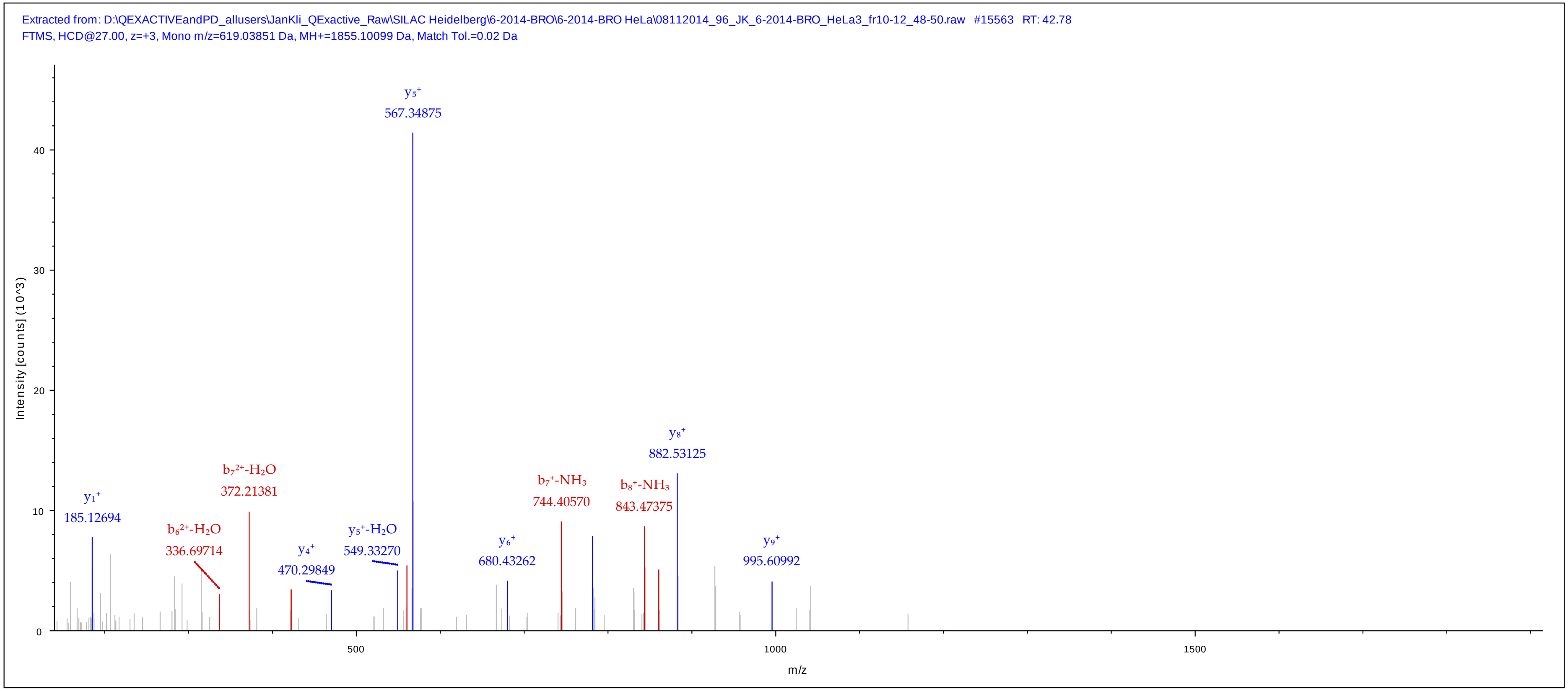

QTPLHLAVITTLPSVVR

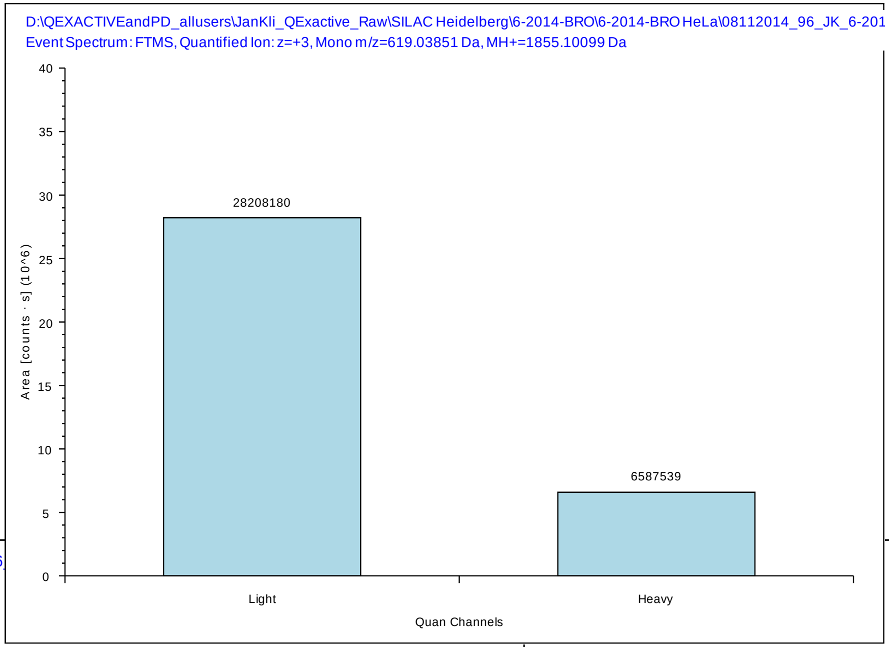

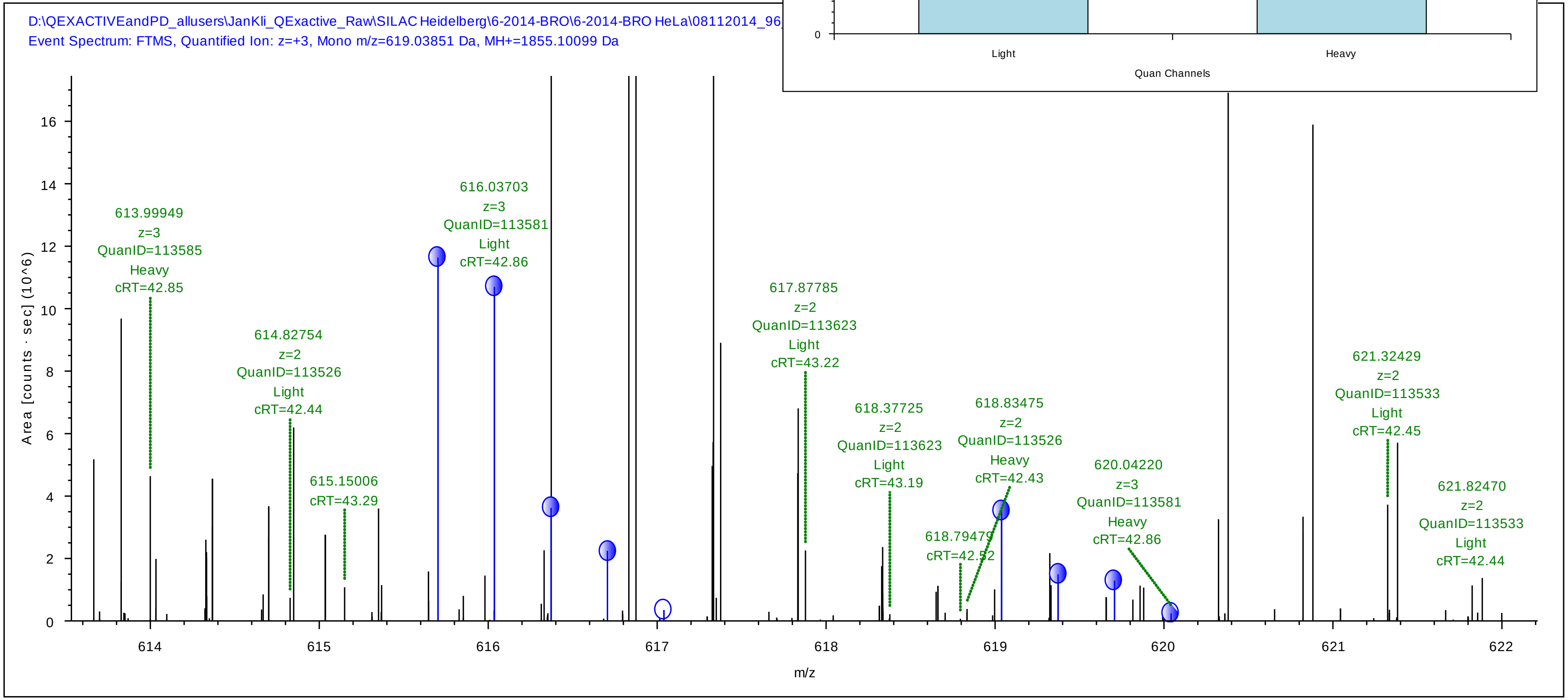

Sequence: GLLPLVR, Charge: +2, Monoisotopic m/z: 384.26022 Da (-0.33 mmu/-0.86 ppm), MH+: 767.51317 Da, RT: 34.75 min,
Identified with: Mascot (v1.30); IonScore:43, Exp Value:2.9E-003, Ions matched by search engine: 5/50
Fragment match tolerance used for search: 0.02 Da

#1	b^+	b^{2+}	Seq.	y^+	y^{2+}	#2
1	58.02875	29.51801	G			7
2	171.11282	86.06005	L	710.49236	355.74982	6
3	284.19689	142.60208	L	597.40829	299.20778	5
4	381.24966	191.12847	P	484.32422	242.66575	4
5	494.33373	247.67050	L	387.27145	194.13936	3
6	593.40215	297.20471	V	274.18738	137.59733	2
7			R	175.11896	88.06312	1

GLLPLVR

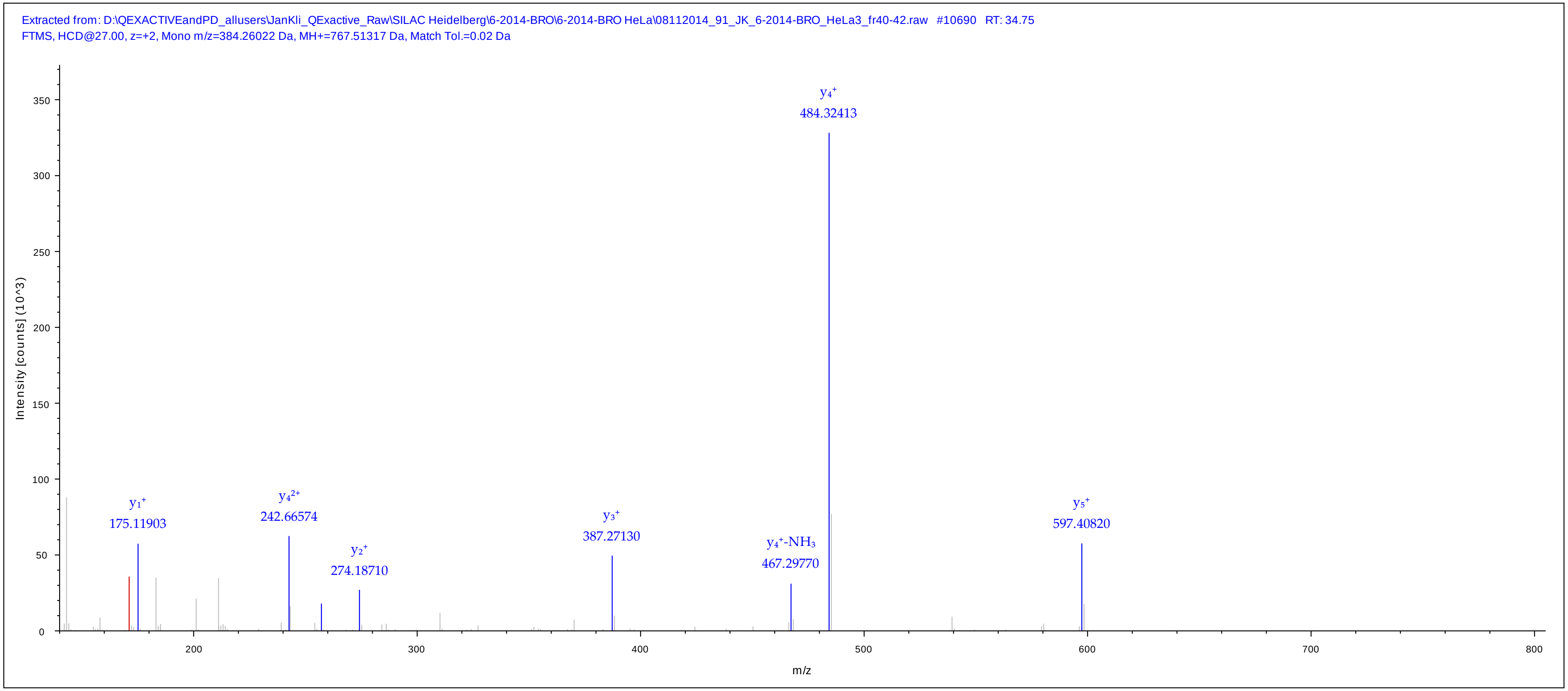

Extracted from: D:\QEXACTIVEandPD_allusers\JanKli_QExactive_Raw\SILAC Heidelberg\6-2014-BRO\6-2014-BRO HeLa\08112014_91_JK_6-2014-BRO_HeLa3_fr40-42.raw #10690 RT: 34.75
FTMS, HCD@27.00, z=+2, Mono m/z=384.26022 Da, MH+=767.51317 Da, Match Tol.=0.02 Da
Intensity [counts] (10^3)
m/z
y4+ 484.32413
y1+ 175.11903
y42+ 242.66574
y2+ 274.18710
y3+ 387.27130
y4+-NH3 467.29770
y5+ 597.40820

GLLPLVR

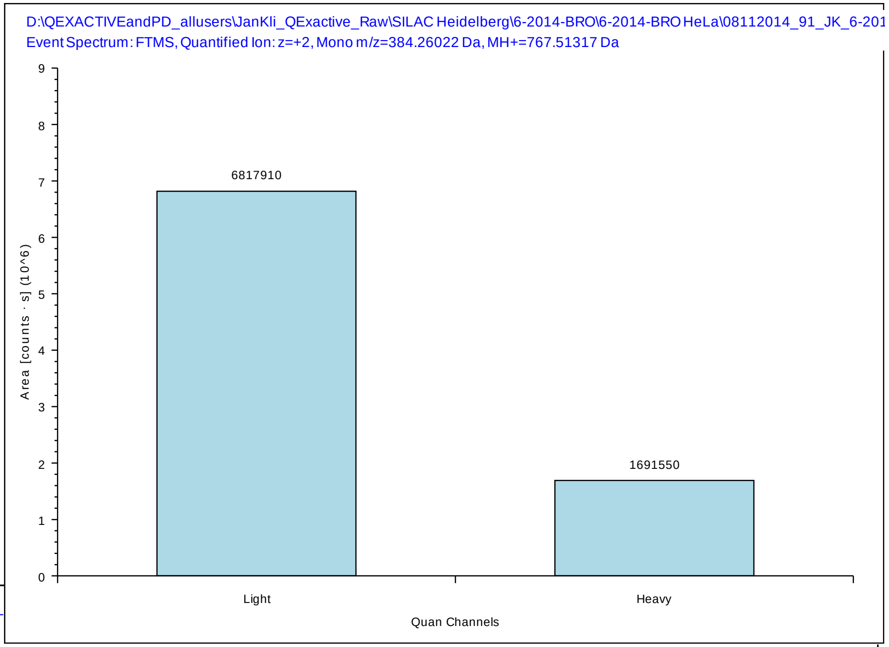

D:\QEXACTIVEandPD_allusers\JanKli_QExactive_Raw\SILAC Heidelberg\6-2014-BRO\6-2014-BRO HeLa\08112014_91_JK_6-201
Event Spectrum: FTMS, Quantified Ion: z=+2, Mono m/z=384.26022 Da, MH+=767.51317 Da
Area [counts · s] (10^6)
6817910
1691550
Light
Heavy
Quan Channels

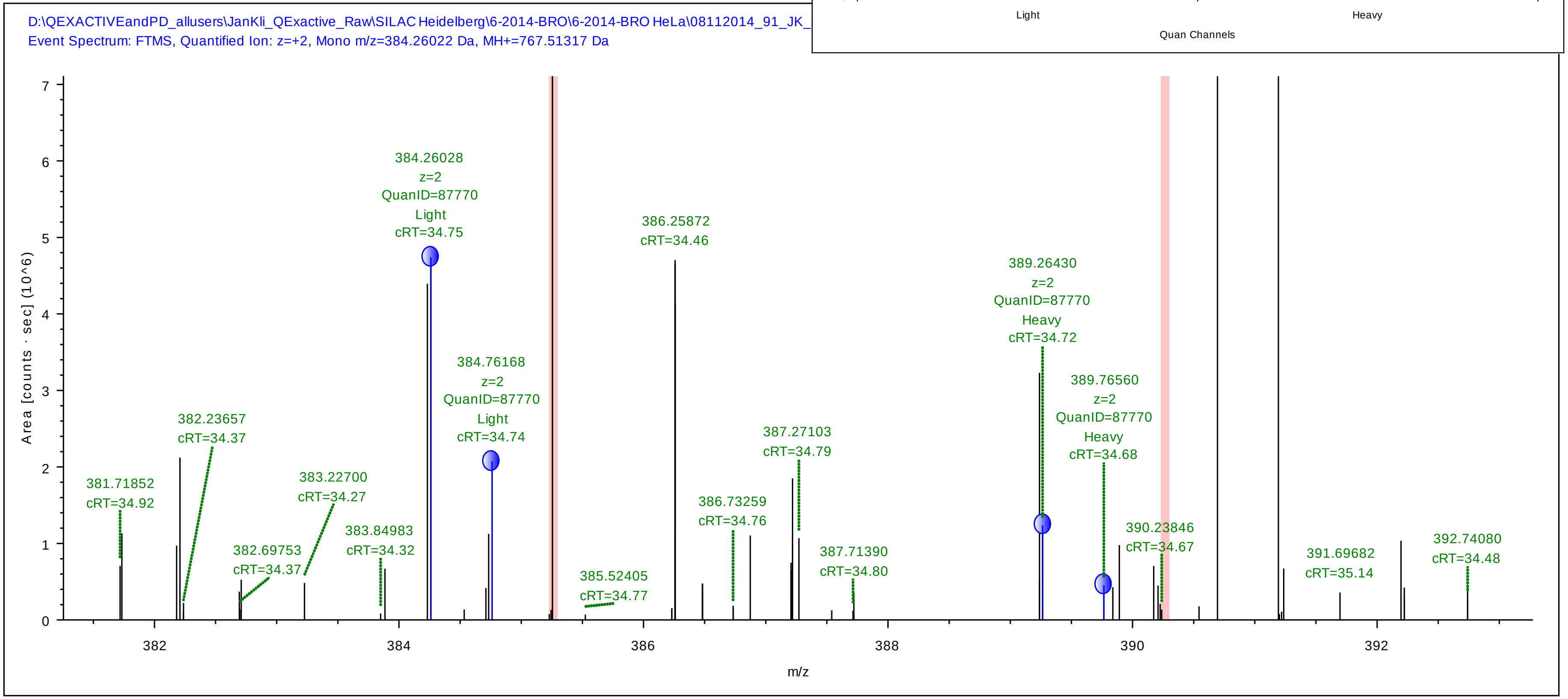

D:\QEXACTIVEandPD_allusers\JanKli_QExactive_Raw\SILAC Heidelberg\6-2014-BRO\6-2014-BRO HeLa\08112014_91_JK_
Event Spectrum: FTMS, Quantified Ion: z=+2, Mono m/z=384.26022 Da, MH+=767.51317 Da
Area [counts · sec] (10^6)
384.26028 z=2 QuanID=87770 Light cRT=34.75
386.25872 cRT=34.46
389.26430 z=2 QuanID=87770 Heavy cRT=34.72
384.76168 z=2 QuanID=87770 Light cRT=34.74
389.76560 z=2 QuanID=87770 Heavy cRT=34.68
382.23657 cRT=34.37
387.27103 cRT=34.79
381.71852 cRT=34.92
383.22700 cRT=34.27
386.73259 cRT=34.76
390.23846 cRT=34.67
392.74080 cRT=34.48
382.69753 cRT=34.37
383.84983 cRT=34.32
387.71390 cRT=34.80
391.69682 cRT=35.14
385.52405 cRT=34.77
m/z

Cyclin-dependent kinase inhibitor 2A, isoform 4 OS=Homo sapiens GN=CDKN2A PE=1 SV=2 - [CD2A2_HUMAN]

Identified peptides:

CPGGLPGHAGGAAPGR
RPGHDDGQRPSGGAAAAPR
VFVVHIPR
LGQQPLPR
RCPGGLPGHAGGAAPGR
VRVFVVHIPR
RFLVTLR
FLVTLR

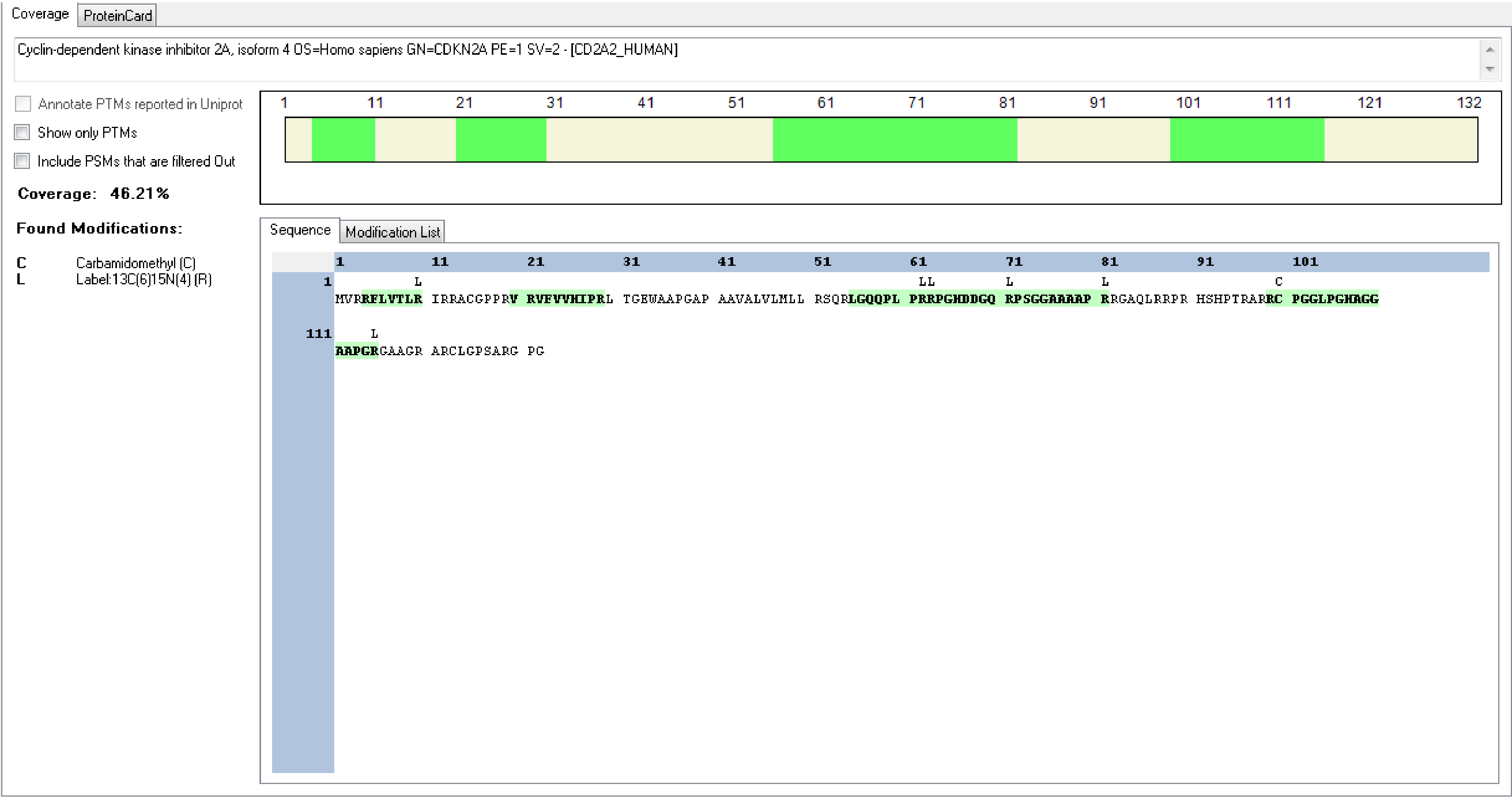
Coverage
ProteinCard
Cyclin-dependent kinase inhibitor 2A, isoform 4 OS=Homo sapiens GN=CDKN2A PE=1 SV=2 - [CD2A2_HUMAN]
Annotate PTMs reported in Uniprot
Show only PTMs
Include PSMs that are filtered Out
Coverage: 46.21%
Found Modifications:
C Carbamidomethyl (C)
L Label:13C(6)15N(4) (R)
1 11 21 31 41 51 61 71 81 91 101 111 121 132
Sequence
Modification List
1 11 21 31 41 51 61 71 81 91 101
1
MVRRFLVTLR IRRACGPPRV RVFVVHIPRL TGEWAAPGAP AAVALVLMLL RSQRLGQQPL PRRPGHDDGQ RPSGGAAAAP RRGAQLRRPR HSHPTRARRC PGGLPGHAGG
111
AAPGRGAAGR ARCLGPSARG PG

Sequence: CPGGLPGHAGGAAPGR, C1-Carbamidomethyl (57.02146 Da), R16-Label:13C(6)15N(4) (10.00827 Da)
Charge: +3, Monoisotopic m/z: 481.23761 Da (-0.36 mmu/-0.75 ppm), MH+: 1441.69828 Da, RT: 16.12 min,
Identified with: Mascot (v1.30); IonScore:56, Exp Value:4.9E-004, Ions matched by search engine: 11/122
Fragment match tolerance used for search: 0.02 Da

#1	b^{+}	b^{2+}	b^{3+}	Seq.	y^{+}	y^{2+}	y^{3+}	#2
1	161.03793	81.02260	54.35083	C-Carbamidomethyl				16
2	258.09070	129.54899	86.70175	P	1281.66870	641.33799	427.89442	15
3	315.11217	158.05972	105.70891	G	1184.61593	592.81160	395.54349	14
4	372.13364	186.57046	124.71606	G	1127.59446	564.30087	376.53634	13
5	485.21771	243.11249	162.41075	L	1070.57299	535.79013	357.52918	12
6	582.27048	291.63888	194.76168	P	957.48892	479.24810	319.83449	11
7	639.29195	320.14961	213.76883	G	860.43615	430.72171	287.48357	10
8	776.35086	388.67907	259.45514	H	803.41468	402.21098	268.47641	9
9	847.38798	424.19763	283.13418	A	666.35577	333.68152	222.79011	8
10	904.40945	452.70836	302.14133	G	595.31865	298.16296	199.11107	7
11	961.43092	481.21910	321.14849	G	538.29718	269.65223	180.10391	6
12	1032.46804	516.73766	344.82753	A	481.27571	241.14149	161.09675	5
13	1103.50516	552.25622	368.50657	A	410.23859	205.62293	137.41771	4
14	1200.55793	600.78260	400.85749	P	339.20147	170.10437	113.73867	3
15	1257.57940	629.29334	419.86465	G	242.14870	121.57799	81.38775	2
16				R-Label:13C(6)15N(4)	185.12723	93.06725	62.38059	1

CPGGLPGHAGGAAPGR

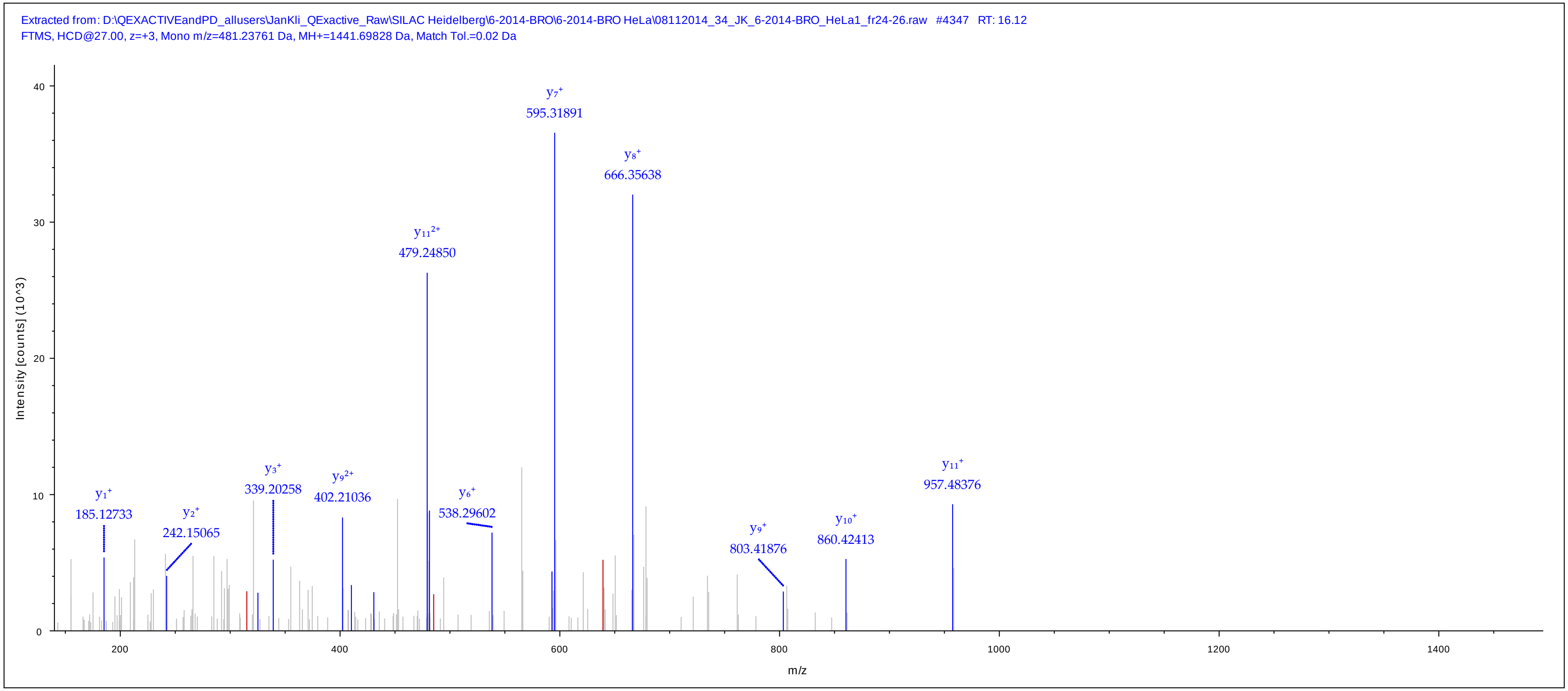

CPGGLPGHAGGAAPGR

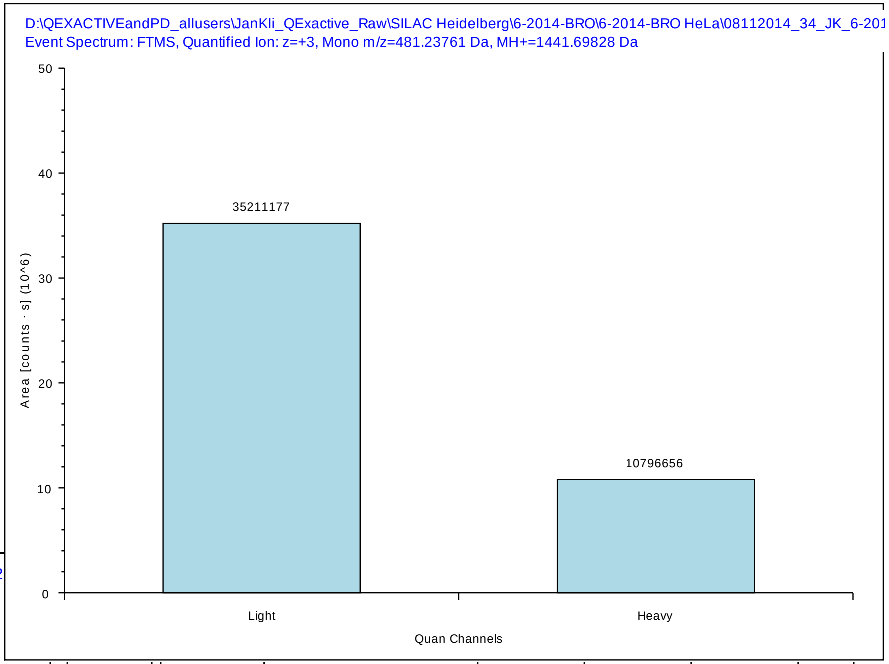

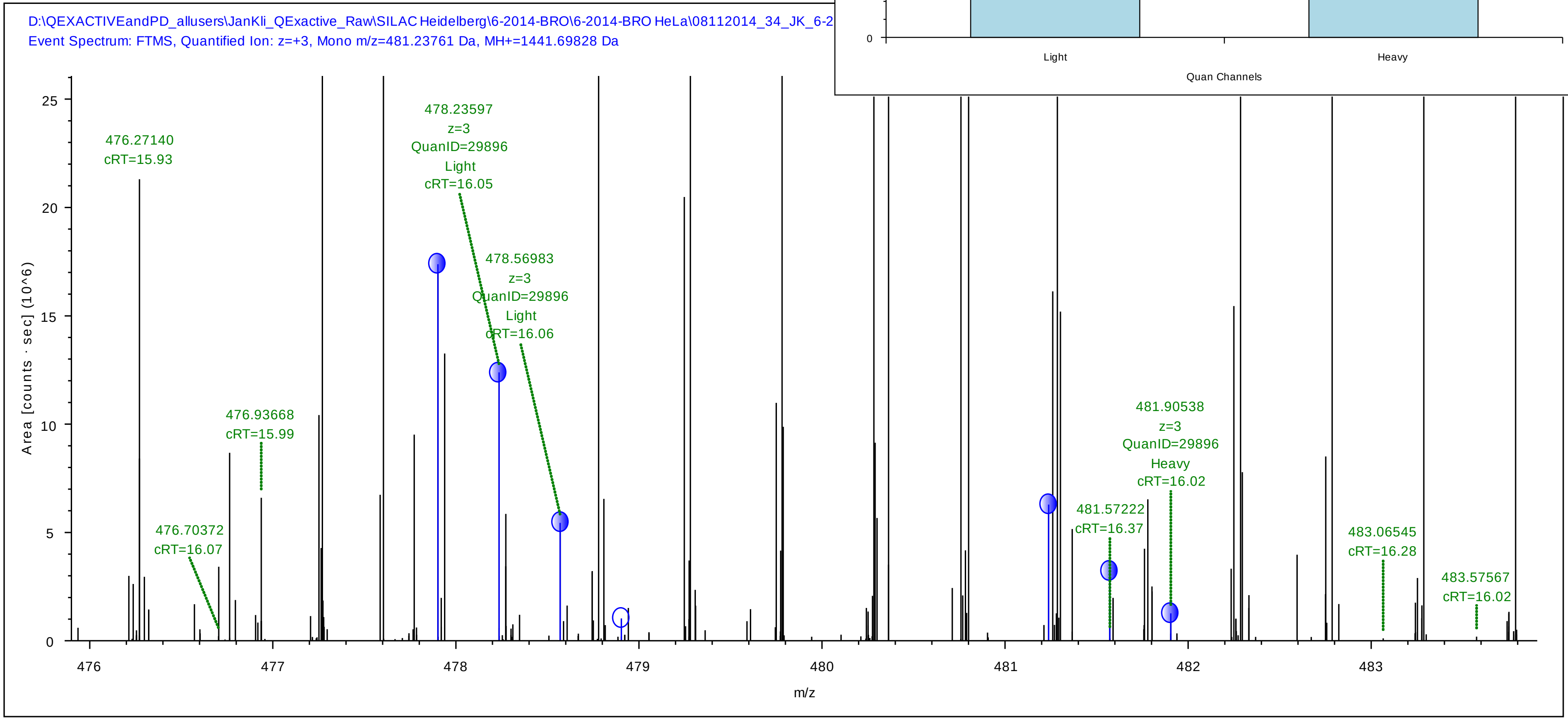

Sequence: VFVVHIPR, Charge: +2, Monoisotopic m/z: 483.79849 Da (+0.66 mmu/+1.36 ppm), MH+: 966.58971 Da, RT: 27.52 min,
Identified with: Mascot (v1.30); IonScore:55, Exp Value:5.5E-004, Ions matched by search engine: 7/58
Fragment match tolerance used for search: 0.02 Da

#1	b^{+}	b^{2+}	Seq.	y^{+}	y^{2+}	#2
1	100.07570	50.54149	V			8
2	247.14412	124.07570	F	867.51997	434.26362	7
3	346.21254	173.60991	V	720.45155	360.72941	6
4	445.28096	223.14412	V	621.38313	311.19520	5
5	582.33987	291.67357	H	522.31471	261.66099	4
6	695.42394	348.21561	I	385.25580	193.13154	3
7	792.47671	396.74199	P	272.17173	136.58950	2
8			R	175.11896	88.06312	1

VFVVHIPR

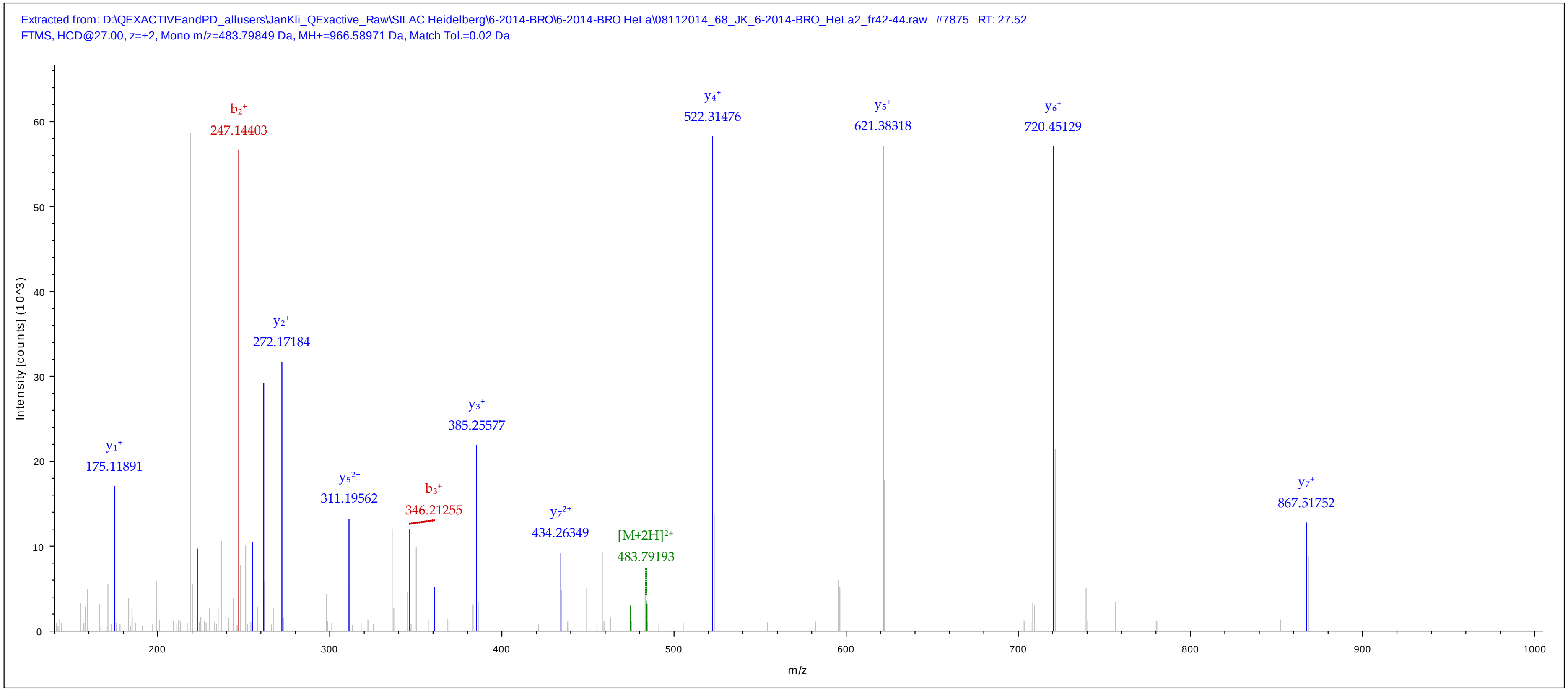

Extracted from: D:\QEXACTIVEandPD_allusers\JanKli_QExactive_Raw\SILAC Heidelberg\6-2014-BRO\6-2014-BRO HeLa\08112014_68_JK_6-2014-BRO_HeLa2_fr42-44.raw #7875 RT: 27.52
FTMS, HCD@27.00, z=+2, Mono m/z=483.79849 Da, MH+=966.58971 Da, Match Tol.=0.02 Da
Intensity [counts] (10^3)
m/z
y1+ 175.11891
b2+ 247.14403
y2+ 272.17184
y52+ 311.19562
b3+ 346.21255
y3+ 385.25577
y72+ 434.26349
[M+2H]2+ 483.79193
y4+ 522.31476
y5+ 621.38318
y6+ 720.45129
y7+ 867.51752

VFVVHIPR

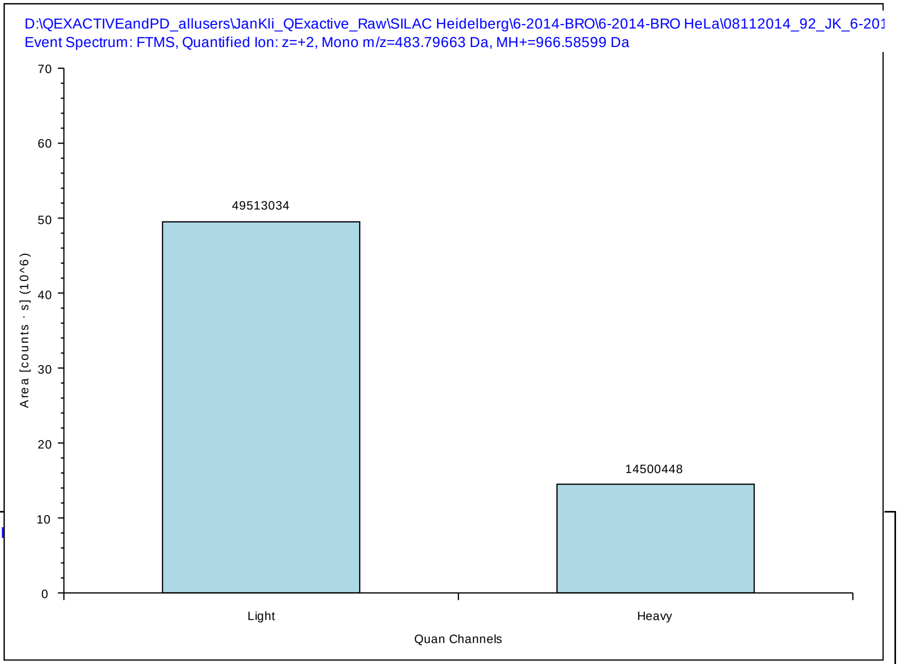
D:\QEXACTIVEandPD_allusers\JanKli_QExactive_Raw\SILAC Heidelberg\6-2014-BRO\6-2014-BRO HeLa\08112014_92_JK_6-201
Event Spectrum: FTMS, Quantified Ion: z=+2, Mono m/z=483.79663 Da, MH+=966.58599 Da
Area [counts · s] (10^6)
49513034
14500448
Light
Heavy
Quan Channels

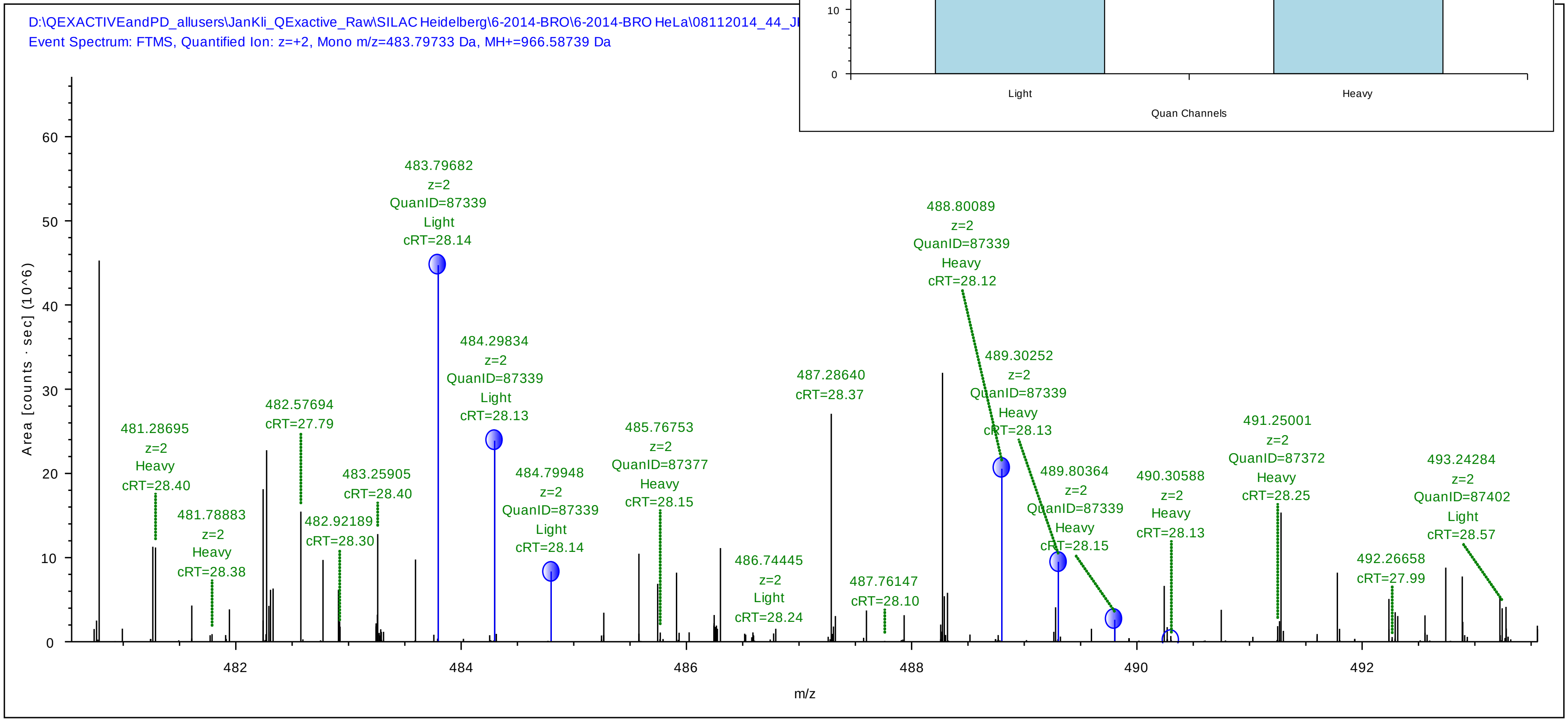
D:\QEXACTIVEandPD_allusers\JanKli_QExactive_Raw\SILAC Heidelberg\6-2014-BRO\6-2014-BRO HeLa\08112014_44_JI
Event Spectrum: FTMS, Quantified Ion: z=+2, Mono m/z=483.79733 Da, MH+=966.58739 Da
Area [counts · sec] (10^6)
483.79682 z=2 QuanID=87339 Light cRT=28.14
484.29834 z=2 QuanID=87339 Light cRT=28.13
484.79948 z=2 QuanID=87339 Light cRT=28.14
488.80089 z=2 QuanID=87339 Heavy cRT=28.12
489.30252 z=2 QuanID=87339 Heavy cRT=28.13
489.80364 z=2 QuanID=87339 Heavy cRT=28.15
490.30588 z=2 Heavy cRT=28.13
481.28695 z=2 Heavy cRT=28.40
481.78883 z=2 Heavy cRT=28.38
482.57694 cRT=27.79
482.92189 cRT=28.30
483.25905 cRT=28.40
485.76753 z=2 QuanID=87377 Heavy cRT=28.15
486.74445 z=2 Light cRT=28.24
487.28640 cRT=28.37
487.76147 cRT=28.10
491.25001 z=2 QuanID=87372 Heavy cRT=28.25
492.26658 cRT=27.99
493.24284 z=2 QuanID=87402 Light cRT=28.57
m/z

Sequence: FLVTLR, Charge: +2, Monoisotopic m/z: 374.73926 Da (-0.19 mmu/-0.51 ppm), MH+: 748.47124 Da, RT: 31.29 min,
Identified with: Mascot (v1.30); IonScore:31, Exp Value:1.1E-001, Ions matched by search engine: 5/40
Fragment match tolerance used for search: 0.02 Da

#1	b^+	b^{2+}	Seq.	y^+	y^{2+}	#2
1	148.07570	74.54149	F			6
2	261.15977	131.08352	L	601.40320	301.20524	5
3	360.22819	180.61773	V	488.31913	244.66320	4
4	461.27587	231.14157	T	389.25071	195.12899	3
5	574.35994	287.68361	L	288.20303	144.60515	2
6			R	175.11896	88.06312	1

FLVTLR

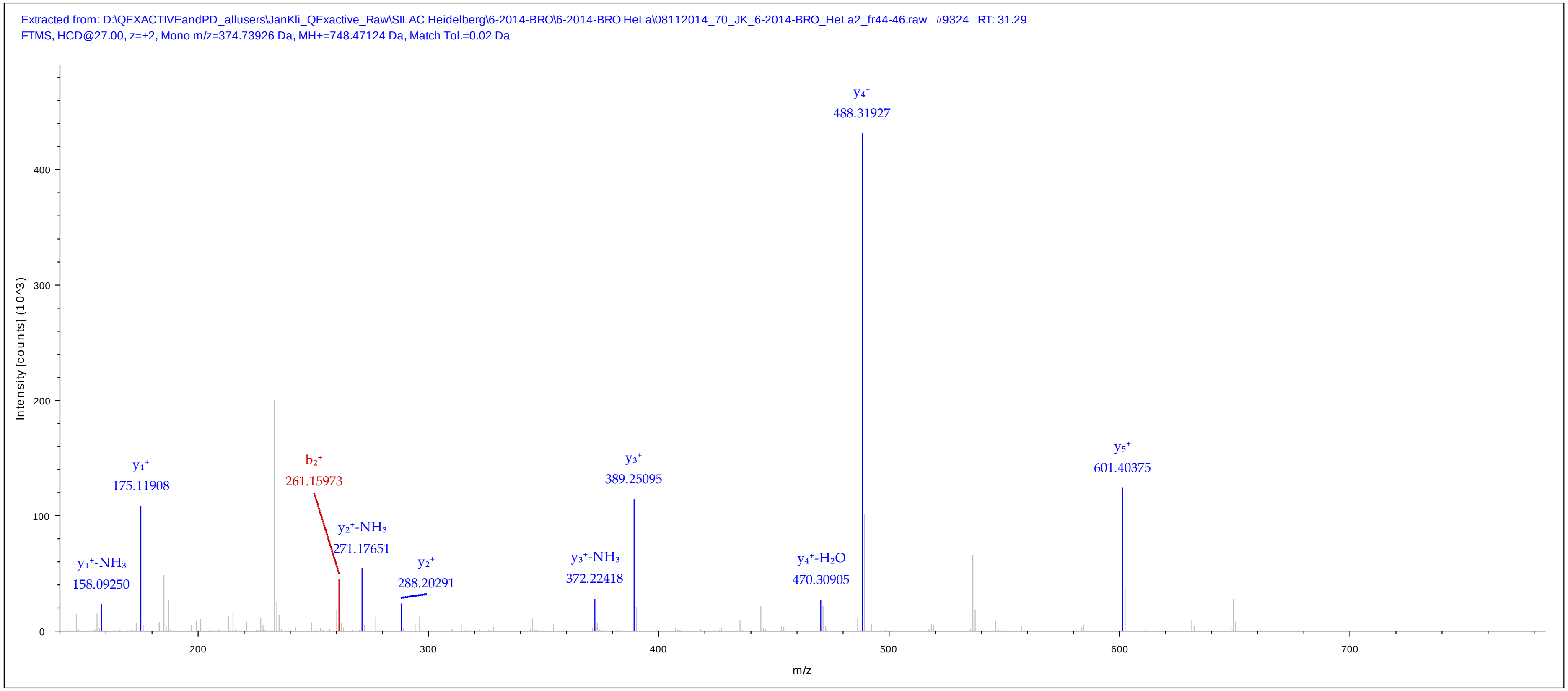

Extracted from: D:\QEXACTIVEandPD_allusers\JanKli_QExactive_Raw\SILAC Heidelberg\6-2014-BRO\6-2014-BRO HeLa\08112014_70_JK_6-2014-BRO_HeLa2_fr44-46.raw #9324 RT: 31.29
FTMS, HCD@27.00, z=+2, Mono m/z=374.73926 Da, MH+=748.47124 Da, Match Tol.=0.02 Da
Intensity [counts] (10^3)
m/z
y4+ 488.31927
y1+ 175.11908
b2+ 261.15973
y3+ 389.25095
y5+ 601.40375
y2+-NH3 271.17651
y1+-NH3 158.09250
y2+ 288.20291
y3+-NH3 372.22418
y4+-H2O 470.30905

FLVTLR

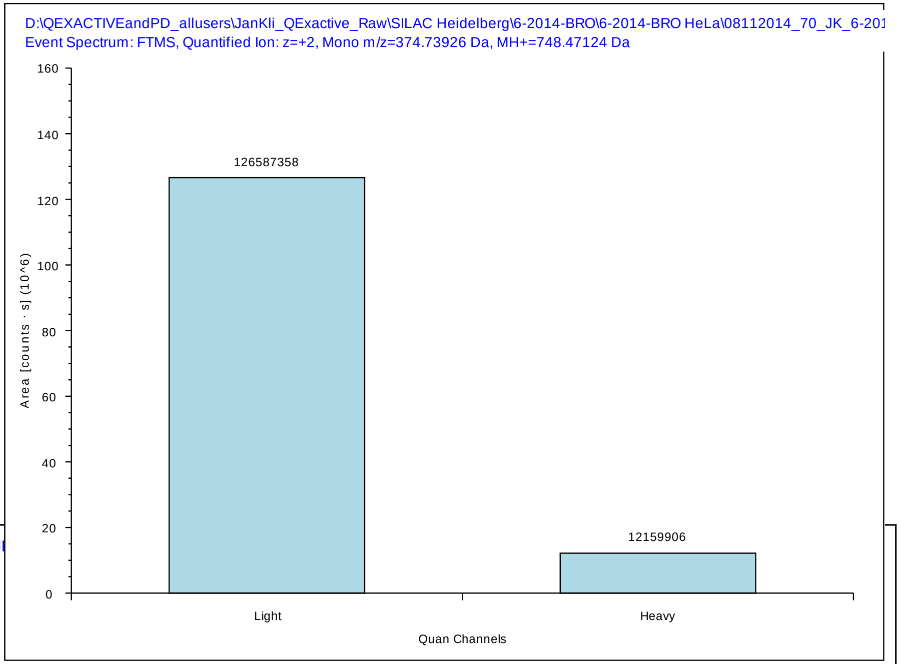

D:\QEXACTIVEandPD_allusers\JanKli_QExactive_Raw\SILAC Heidelberg\6-2014-BRO\6-2014-BRO HeLa\08112014_70_JK_6-201
Event Spectrum: FTMS, Quantified Ion: z=+2, Mono m/z=374.73926 Da, MH+=748.47124 Da
Area [counts · s] (10^6)
126587358
12159906
Light
Heavy
Quan Channels

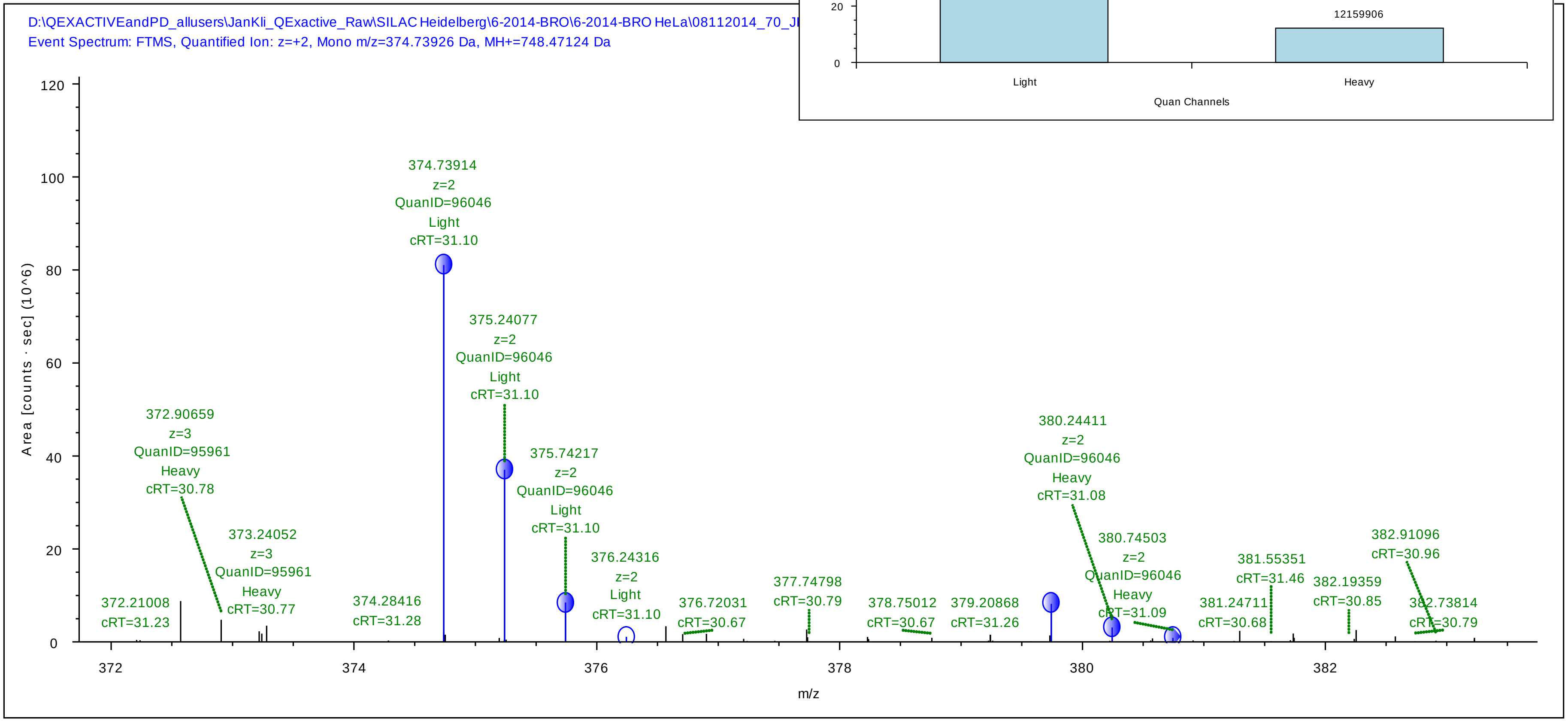

D:\QEXACTIVEandPD_allusers\JanKli_QExactive_Raw\SILAC Heidelberg\6-2014-BRO\6-2014-BRO HeLa\08112014_70_JI
Event Spectrum: FTMS, Quantified Ion: z=+2, Mono m/z=374.73926 Da, MH+=748.47124 Da
Area [counts · sec] (10^6)
374.73914 z=2 QuanID=96046 Light cRT=31.10
375.24077 z=2 QuanID=96046 Light cRT=31.10
375.74217 z=2 QuanID=96046 Light cRT=31.10
376.24316 z=2 Light cRT=31.10
372.90659 z=3 QuanID=95961 Heavy cRT=30.78
373.24052 z=3 QuanID=95961 Heavy cRT=30.77
372.21008 cRT=31.23
374.28416 cRT=31.28
376.72031 cRT=30.67
377.74798 cRT=30.79
378.75012 cRT=30.67
379.20868 cRT=31.26
380.24411 z=2 QuanID=96046 Heavy cRT=31.08
380.74503 z=2 QuanID=96046 Heavy cRT=31.09
381.24711 cRT=30.68
381.55351 cRT=31.46
382.19359 cRT=30.85
382.91096 cRT=30.96
382.73814 cRT=30.79
m/z

Cytochrome c oxidase assembly protein COX19 OS=Homo sapiens GN=COX19 PE=1 SV=1 - [COX19_HUMAN]

Identified peptides:

GSFPLDHLGECK
LGFGDLTSGK
STAMNFGTK
LMLQEPLEK
KLMLQEPLEK

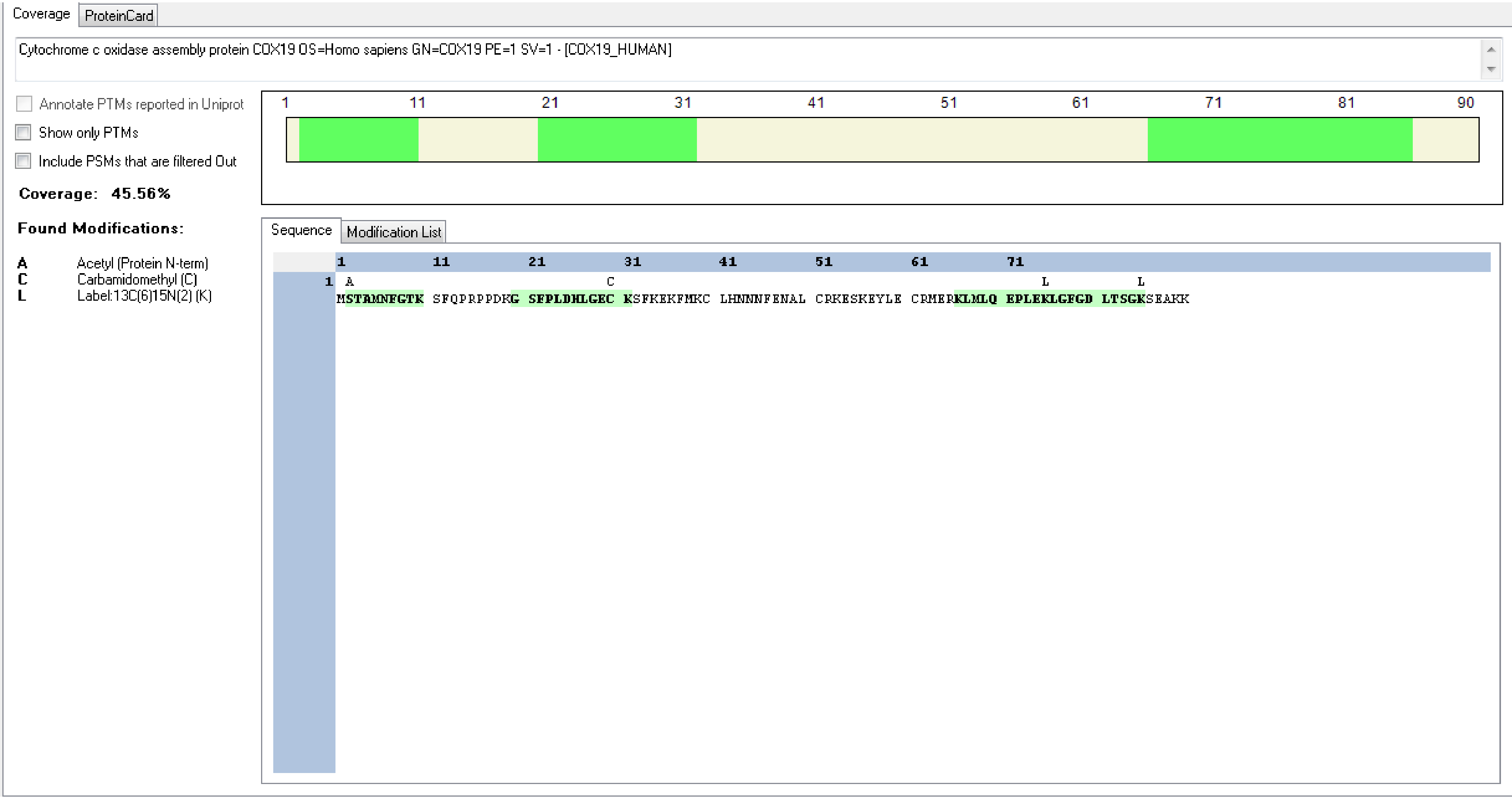

Coverage
ProteinCard
Cytochrome c oxidase assembly protein COX19 OS=Homo sapiens GN=COX19 PE=1 SV=1 - [COX19_HUMAN]
Annotate PTMs reported in Uniprot
Show only PTMs
Include PSMs that are filtered Out
Coverage: 45.56%
1 11 21 31 41 51 61 71 81 90
Found Modifications:
A Acetyl (Protein N-term)
C Carbamidomethyl (C)
L Label:13C(6)15N(2) (K)
Sequence
Modification List
1 11 21 31 41 51 61 71
1
MSTAMNFGTK SFQPRPPDKG SFPLDHLGEC KSFKEKFMKC LHNNNFENAL CRKESKEYLE CRMERKLMLQ EPLEKLGFGD LTSGKSEAKK

Sequence: LGFGDLTSGK, Charge: +2, Monoisotopic m/z: 497.76285 Da (-1.01 mmu/-2.03 ppm), MH+: 994.51842 Da, RT: 32.94 min,
Identified with: Mascot (v1.30); IonScore:93, Exp Value:1.0E-007, Ions matched by search engine: 8/78
Fragment match tolerance used for search: 0.02 Da

#1	b^{+}	b^{2+}	Seq.	y^{+}	y^{2+}	#2
1	114.09135	57.54931	L			10
2	171.11282	86.06005	G	881.43637	441.22182	9
3	318.18124	159.59426	F	824.41490	412.71109	8
4	375.20271	188.10499	G	677.34648	339.17688	7
5	490.22966	245.61847	D	620.32501	310.66614	6
6	603.31373	302.16050	L	505.29806	253.15267	5
7	704.36141	352.68434	T	392.21399	196.61063	4
8	791.39344	396.20036	S	291.16631	146.08679	3
9	848.41491	424.71109	G	204.13428	102.57078	2
10			K	147.11281	74.06004	1

LGFGDLTSGK

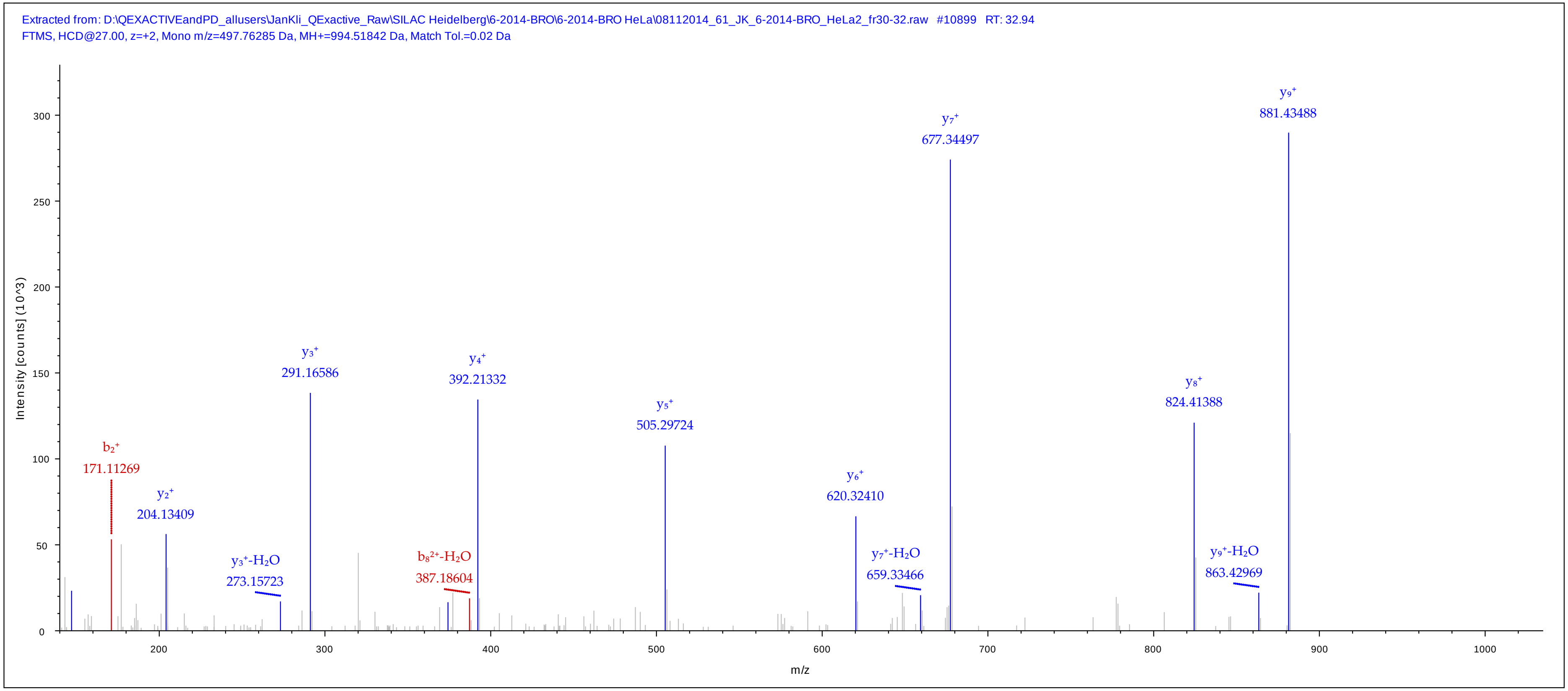
Extracted from: D:\QEXACTIVEandPD_allusers\JanKli_QExactive_Raw\SILAC Heidelberg\6-2014-BRO\6-2014-BRO HeLa\08112014_61_JK_6-2014-BRO_HeLa2_fr30-32.raw #10899 RT: 32.94
FTMS, HCD@27.00, z=+2, Mono m/z=497.76285 Da, MH+=994.51842 Da, Match Tol.=0.02 Da
Intensity [counts] (10^3)
m/z
b2+ 171.11269
y2+ 204.13409
y3+-H2O 273.15723
y3+ 291.16586
b8 2+-H2O 387.18604
y4+ 392.21332
y5+ 505.29724
y6+ 620.32410
y7+-H2O 659.33466
y7+ 677.34497
y8+ 824.41388
y9+-H2O 863.42969
y9+ 881.43488

LGFGDLTSGK

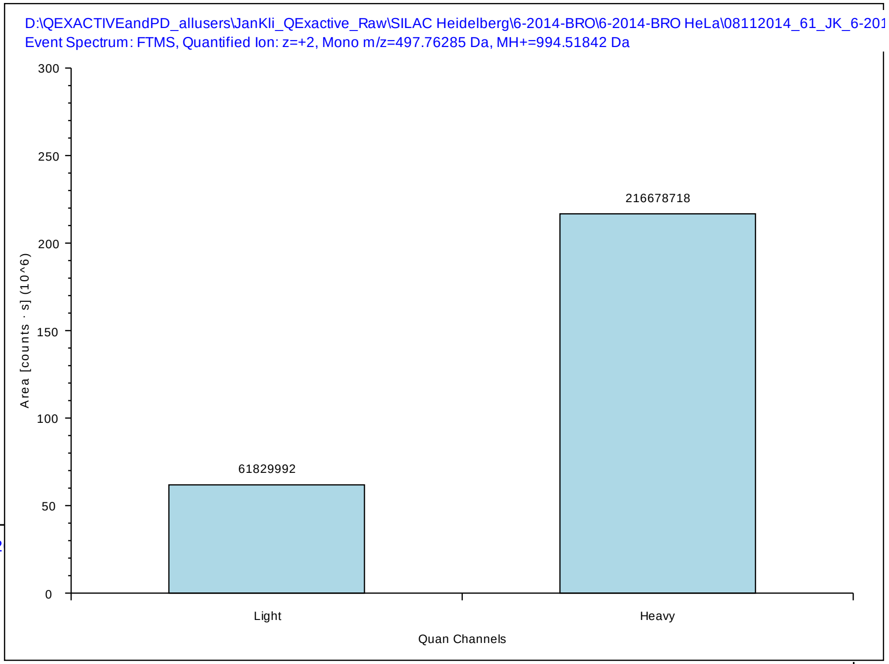
D:\QEXACTIVEandPD_allusers\JanKli_QExactive_Raw\SILAC Heidelberg\6-2014-BRO\6-2014-BRO HeLa\08112014_61_JK_6-201
Event Spectrum: FTMS, Quantified Ion: z=+2, Mono m/z=497.76285 Da, MH+=994.51842 Da
300
250
200
150
100
50
0
Area [counts · s] (10^6)
61829992
216678718
Light
Heavy
Quan Channels

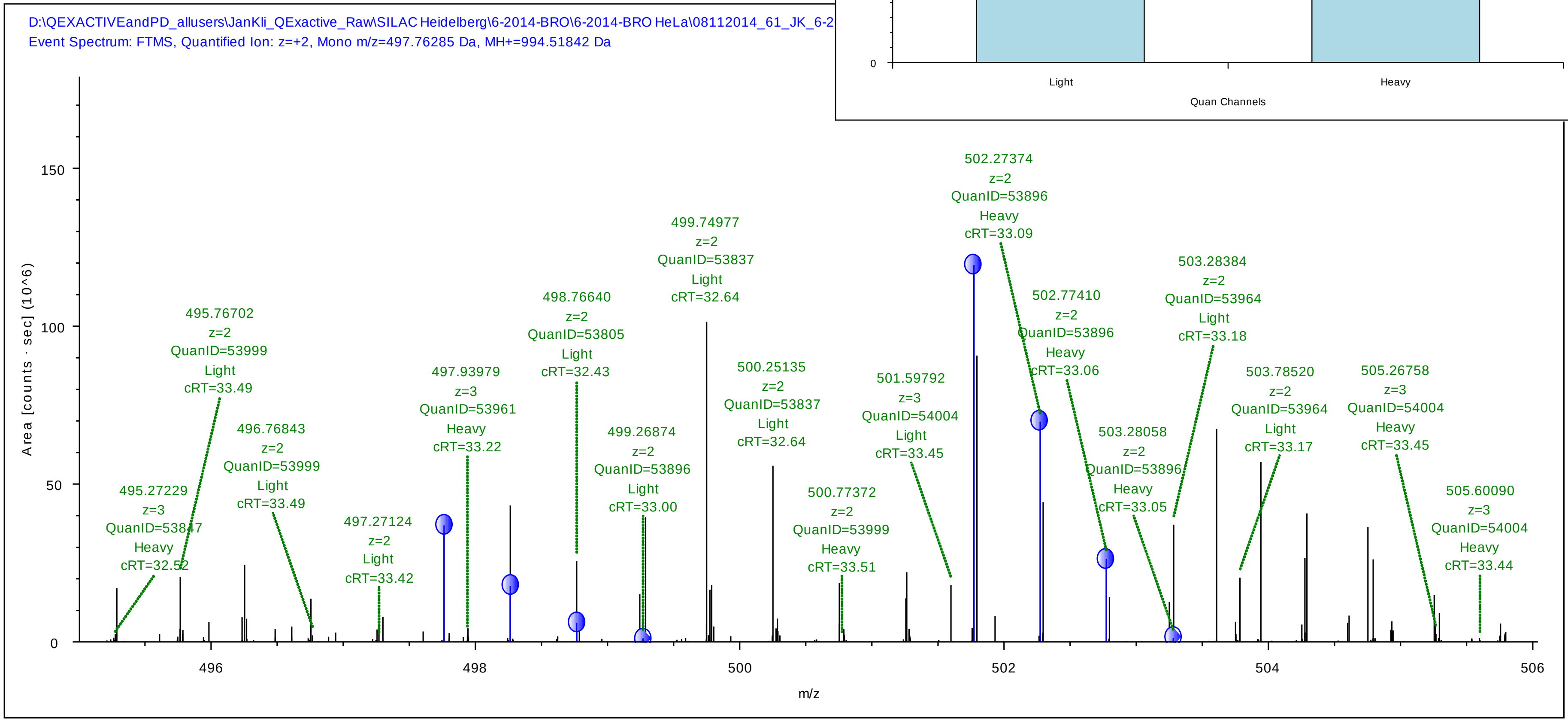
D:\QEXACTIVEandPD_allusers\JanKli_QExactive_Raw\SILAC Heidelberg\6-2014-BRO\6-2014-BRO HeLa\08112014_61_JK_6-2
Event Spectrum: FTMS, Quantified Ion: z=+2, Mono m/z=497.76285 Da, MH+=994.51842 Da
Area [counts · sec] (10^6)
150
100
50
0
495.27229 z=3 QuanID=53847 Heavy cRT=32.52
495.76702 z=2 QuanID=53999 Light cRT=33.49
496.76843 z=2 QuanID=53999 Light cRT=33.49
497.27124 z=2 Light cRT=33.42
497.93979 z=3 QuanID=53961 Heavy cRT=33.22
498.76640 z=2 QuanID=53805 Light cRT=32.43
499.26874 z=2 QuanID=53896 Light cRT=33.00
499.74977 z=2 QuanID=53837 Light cRT=32.64
500.25135 z=2 QuanID=53837 Light cRT=32.64
500.77372 z=2 QuanID=53999 Heavy cRT=33.51
501.59792 z=3 QuanID=54004 Light cRT=33.45
502.27374 z=2 QuanID=53896 Heavy cRT=33.09
502.77410 z=2 QuanID=53896 Heavy cRT=33.06
503.28058 z=2 QuanID=53896 Heavy cRT=33.05
503.28384 z=2 QuanID=53964 Light cRT=33.18
503.78520 z=2 QuanID=53964 Light cRT=33.17
505.26758 z=3 QuanID=54004 Heavy cRT=33.45
505.60090 z=3 QuanID=54004 Heavy cRT=33.44
496
498
500
502
504
506
m/z

2'-5'-oligoadenylate synthase-like protein OS=Homo sapiens GN=OASL PE=1 SV=2 - [OASL_HUMAN]

Identified peptides:

ALMQELYSTPASR
ACGGPGNFCPSFSELQR
LDSFVAQWLQPHR
QQIEDQQGLPK
GYPDFNLIVNPYEPIRK
ERPIILDPADPTLNVAEGYR
NPDGGSYAYAINPNSFILGLK
GTAEPITVTIVPAYR
EWKEEVLDAVR
ALGPSLPNSQPPPEVYVSLIK

Coverage | ProteinCard

2'-5'-oligoadenylate synthase-like protein OS=Homo sapiens GN=OASL PE=1 SV=2 - [OASL_HUMAN]

- [ ] Annotate PTMs reported in Uniprot
- [ ] Show only PTMs
- [ ] Include PSMs that are filtered Out

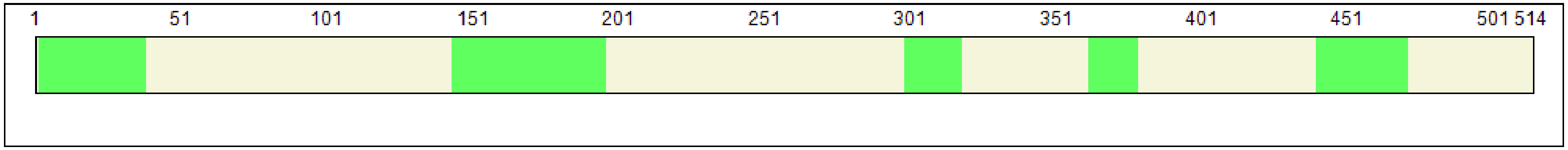

Coverage: 30.93%

Found Modifications:

**A**	Acetyl (Protein N-term)
**C**	Carbamidomethyl (C)
**L**	Label:13C(6)15N(2) (K)
**M**	Label:13C(6)15N(4) (R)

Sequence | Modification List

```
     1         11        21        31        41        51        61        71        81        91        101
  1   A         M            M
     MALMQELYST PASRLDSFVA QWLQPHREWK EEVLDAVRTV EEFLRQEHFQ GKRGLDQDVR VLKVVKVGSF GNGTVLRSTR EVELVAFLSC FHSFQEAAKH HKDVLRLIWK

111                                                                  C      C
     TMWQSQDLLD LGLEDLRMEQ RVPDALVFTI QTRGTAEPIT VTIVPAYRAL GPSLPNSQPP PEVYVSLIKA CGGPGNFCPS FSELQRNFVK HRPTKLKSLL RLVKHWYQQY

221
     VKARSPRANL PPLYALELLT IYAWEMGTEE DENFMLDEGF TTVMDLLLEY EVICIYWTKY YTLHNAIIED CVRKQLKKER PIILDPADPT LNVAEGYRWD IVAQRASQCL

331
     KQDCCYDNRE NPISSWNVKR ARDIHLTVEQ RGYPDFNLIV NPYEPIRKVK EKIRRTRGYS GLQRLSFQVP GSERQLLSSR CSLAKYGIFS HTHIYLLETI PSEIQVFVKN

441                              L
     PDGGSYAYAI NPNSFILGLK QQIEDQQGLP KKQQQLEFQG QVLQDWLGLG IYGIQDSDTL ILSKKKGEAL FPAS
```

Sequence: ALMQELYSTPASR, A1-Acetyl (42.01057 Da), R13-Label:13C(6)15N(4) (10.00827 Da)
Charge: +2, Monoisotopic m/z: 759.87659 Da (-1.9 mmu/-2.49 ppm), MH+: 1518.74590 Da, RT: 49.55 min,
Identified with: Mascot (v1.30); IonScore:78, Exp Value:3.5E-006, Ions matched by search engine: 8/128
Fragment match tolerance used for search: 0.02 Da

#1	b^+	b^{2+}	Seq.	y^+	y^{2+}	#2
1	114.05496	57.53112	A-Acetyl			13
2	227.13903	114.07315	L	1405.70200	703.35464	12
3	358.17953	179.59340	M	1292.61793	646.81260	11
4	486.23811	243.62269	Q	1161.57743	581.29235	10
5	615.28071	308.14399	E	1033.51885	517.26306	9
6	728.36478	364.68603	L	904.47625	452.74176	8
7	891.42810	446.21769	Y	791.39218	396.19973	7
8	978.46013	489.73370	S	628.32886	314.66807	6
9	1079.50781	540.25754	T	541.29683	271.15205	5
10	1176.56058	588.78393	P	440.24915	220.62821	4
11	1247.59770	624.30249	A	343.19638	172.10183	3
12	1334.62973	667.81850	S	272.15926	136.58327	2
13			R-Label:13C(6)15N(4)	185.12723	93.06725	1

ALMQELYSTPASR

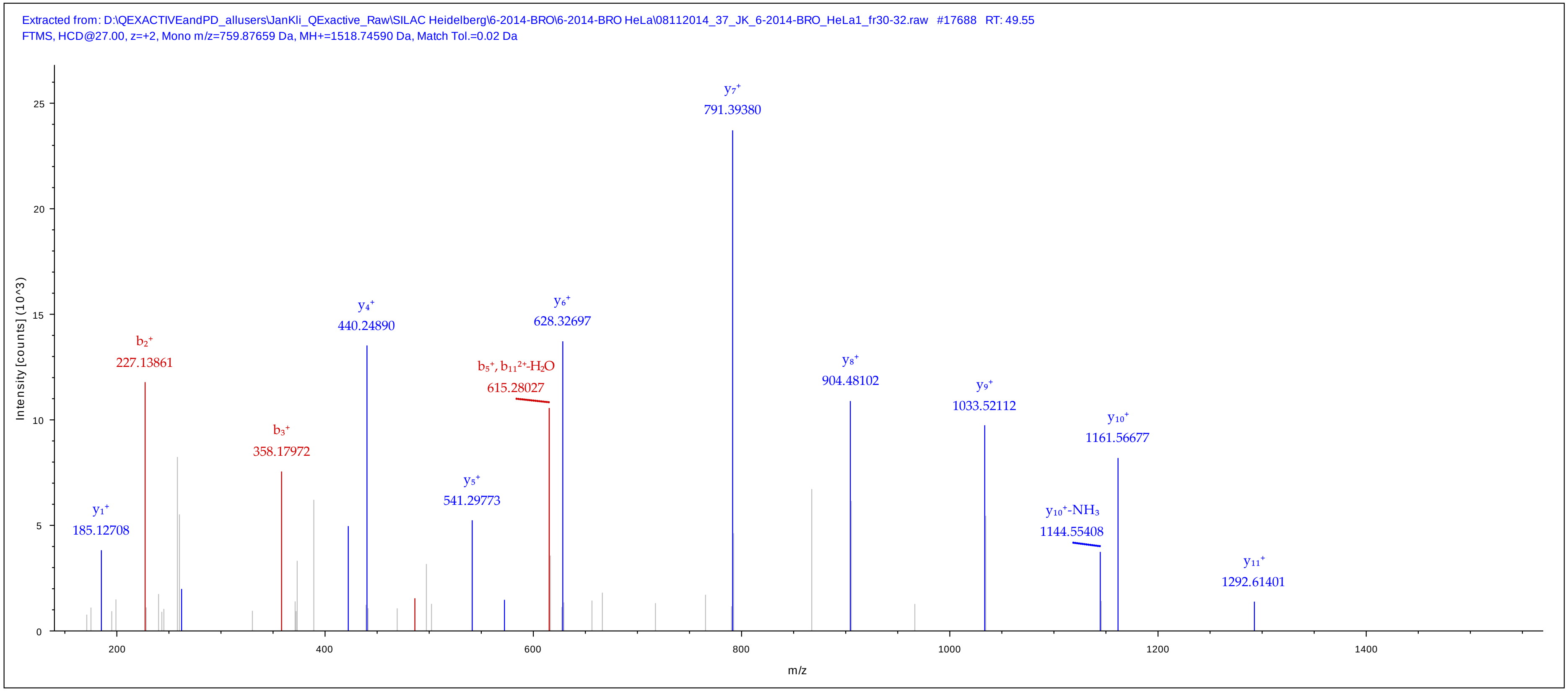

ALMQELYSTPASR

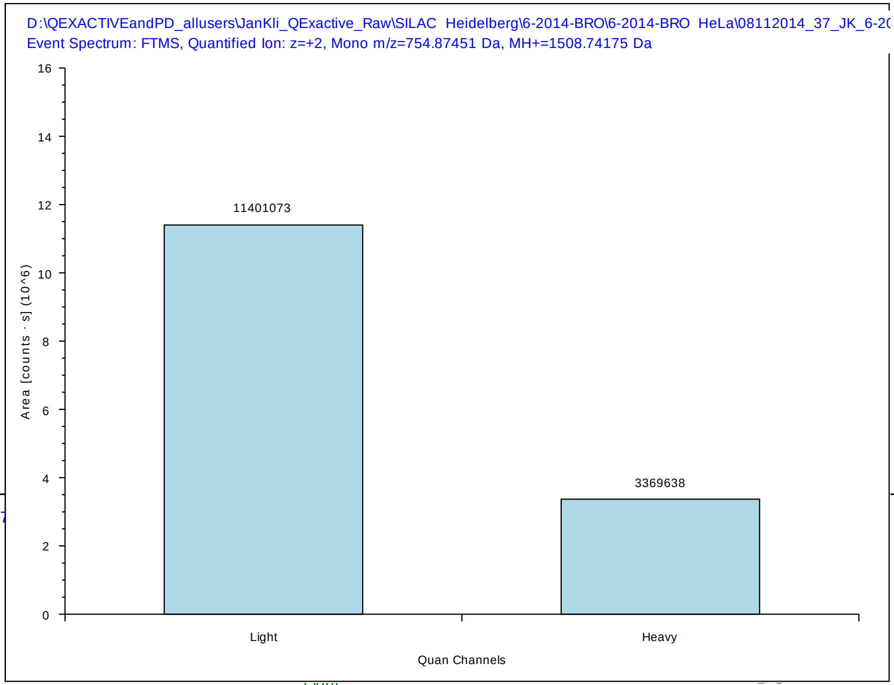

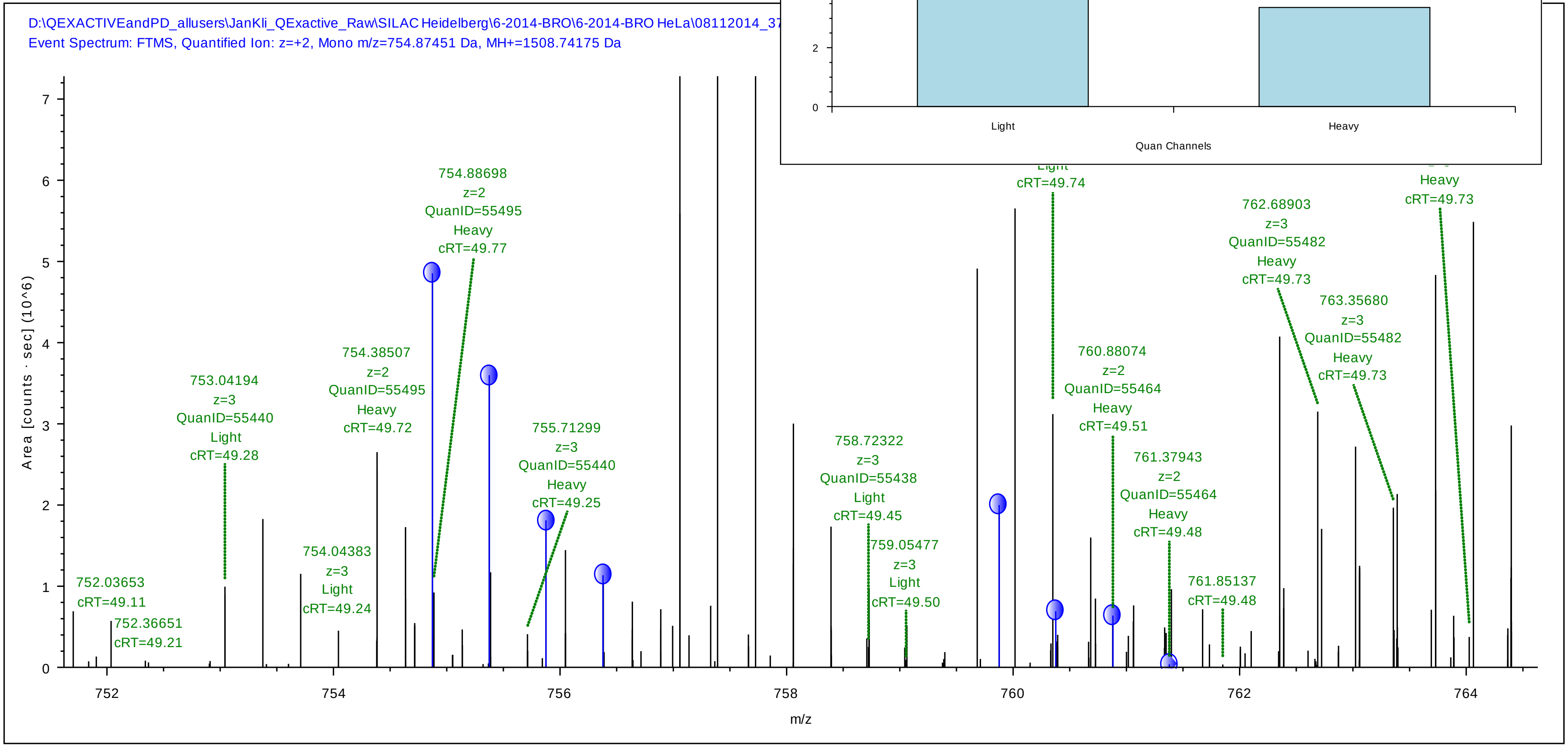

Sequence: ACGGPGNFCPSFSELQR, C2-Carbamidomethyl (57.02146 Da), C9-Carbamidomethyl (57.02146 Da)
Charge: +2, Monoisotopic m/z: 942.40723 Da (-4.62 mmu/-4.91 ppm), MH+: 1883.80718 Da, RT: 36.43 min,
Identified with: Mascot (v1.30); IonScore:55, Exp Value:1.4E-004, Ions matched by search engine: 13/154
Fragment match tolerance used for search: 0.02 Da

#1	b^{+}	b^{2+}	Seq.	y^{+}	y^{2+}	#2
1	72.04440	36.52584	A			17
2	232.07505	116.54116	C-Carbamidomethyl	1812.77930	906.89329	16
3	289.09652	145.05190	G	1652.74865	826.87796	15
4	346.11799	173.56263	G	1595.72718	798.36723	14
5	443.17076	222.08902	P	1538.70571	769.85649	13
6	500.19223	250.59975	G	1441.65294	721.33011	12
7	614.23516	307.62122	N	1384.63147	692.81937	11
8	761.30358	381.15543	F	1270.58854	635.79791	10
9	921.33423	461.17076	C-Carbamidomethyl	1123.52012	562.26370	9
10	1018.38700	509.69714	P	963.48946	482.24837	8
11	1105.41903	553.21316	S	866.43669	433.72198	7
12	1252.48745	626.74737	F	779.40466	390.20597	6
13	1339.51948	670.26338	S	632.33624	316.67176	5
14	1468.56208	734.78468	E	545.30421	273.15574	4
15	1581.64615	791.32672	L	416.26161	208.63444	3
16	1709.70473	855.35601	Q	303.17754	152.09241	2
17			R	175.11896	88.06312	1

ACGGPGNFCPSFSELQR

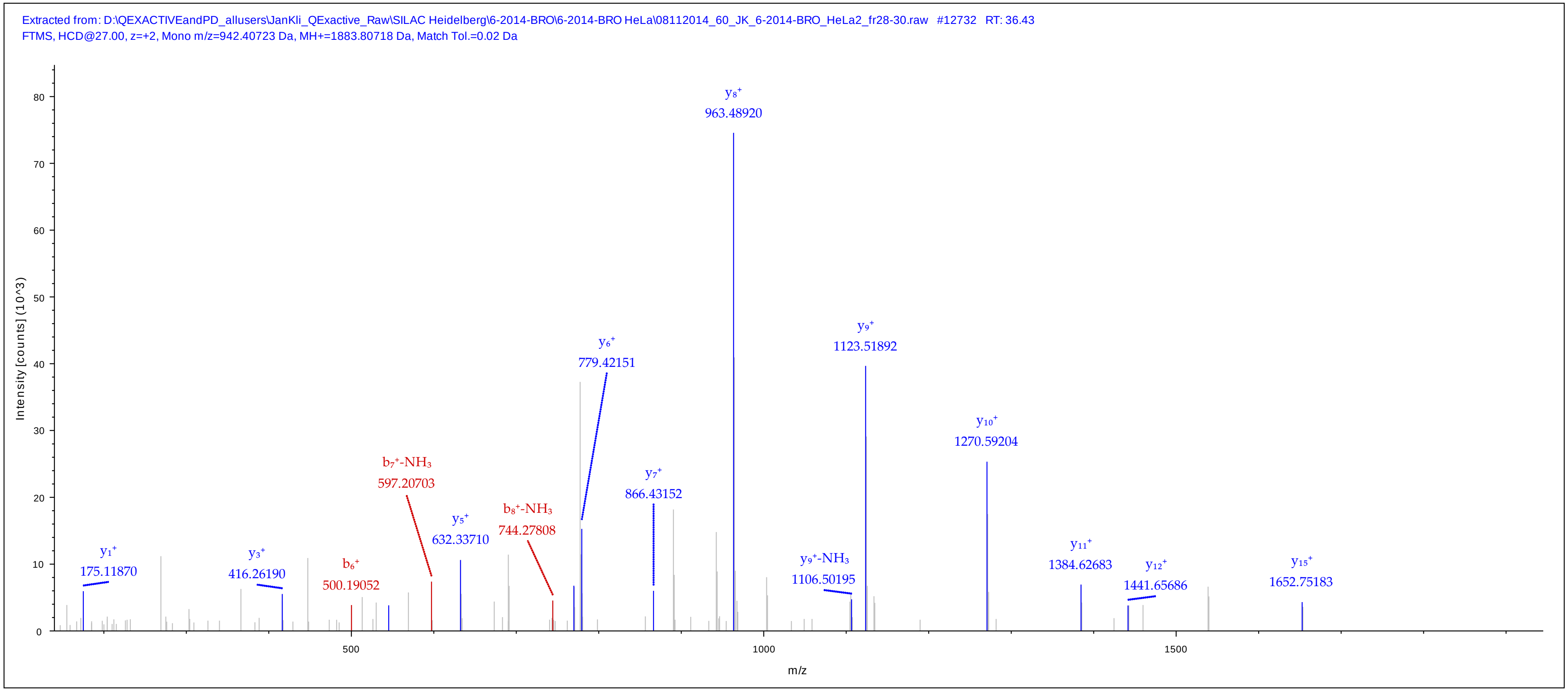

ACGGPGNFCPSFSELQR

Not used for quantification
(missing heavy channel)

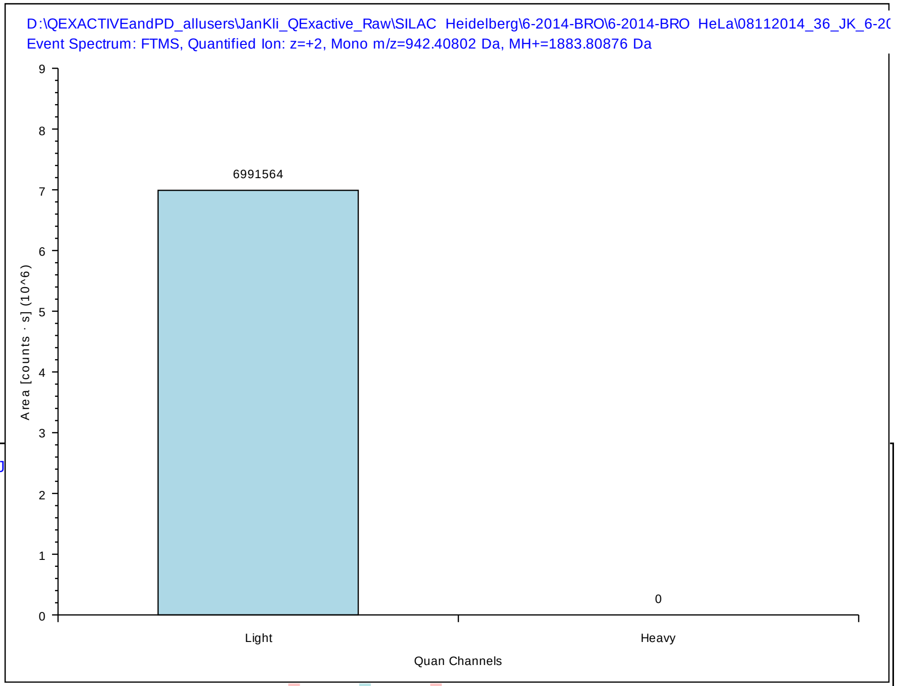

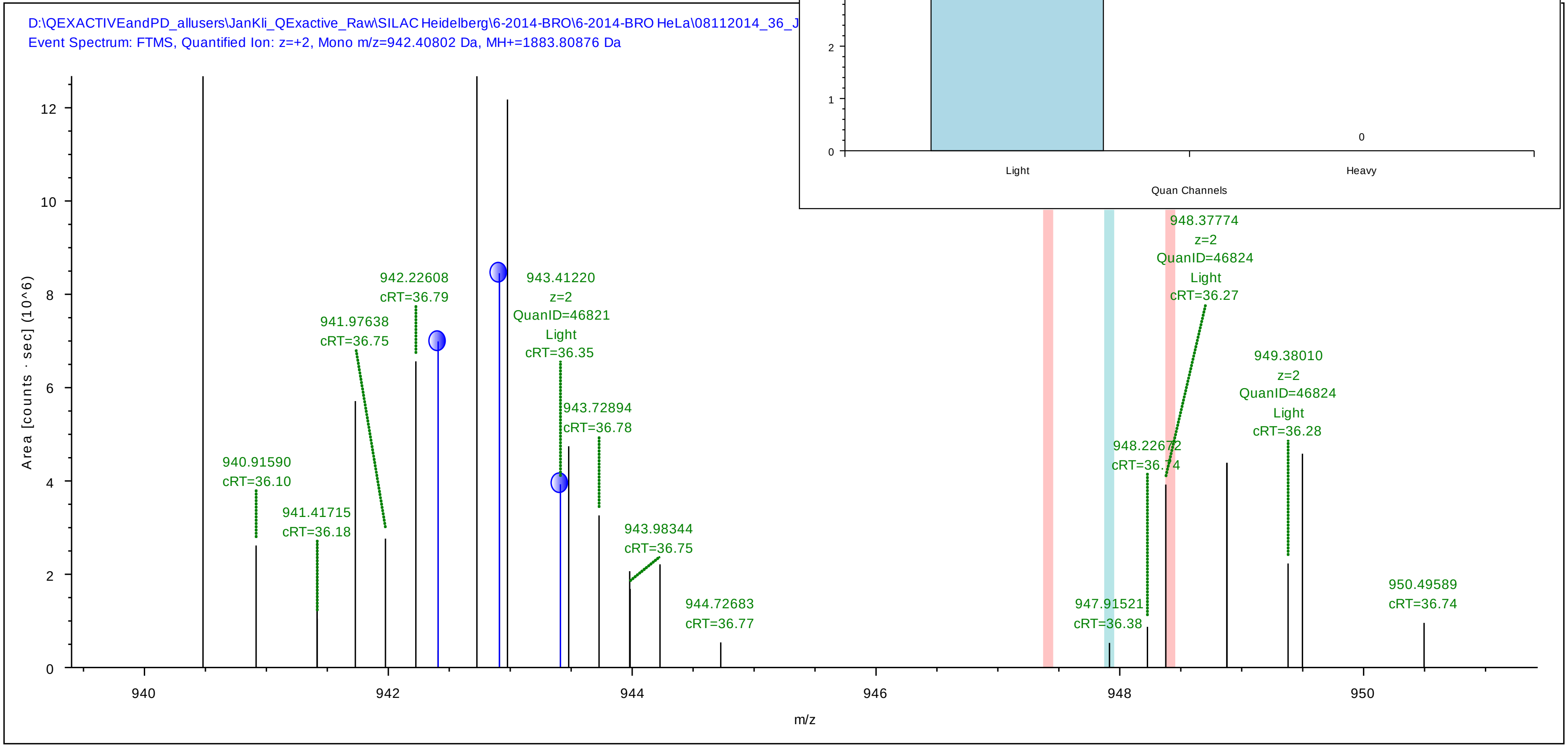

Sequence: LDSFVAQWLQPHR, R13-Label:13C(6)15N(4) (10.00827 Da)
Charge: +3,   Monoisotopic m/z: 536.28247 Da (-1.21 mmu/-2.25 ppm),   MH+: 1606.83286 Da,   RT: 42.74 min,
Identified with: Mascot (v1.30); IonScore:70, Exp Value:2.8E-005, Ions matched by search engine: 8/110
Fragment match tolerance used for search: 0.02 Da

#1	b^{+}	b^{2+}	b^{3+}	Seq.	y^{+}	y^{2+}	y^{3+}	#2
1	114.09135	57.54931	38.70197	L				13
2	229.11830	115.06279	77.04428	D	1493.75240	747.37984	498.58898	12
3	316.15033	158.57880	106.05496	S	1378.72545	689.86636	460.24667	11
4	463.21875	232.11301	155.07777	F	1291.69342	646.35035	431.23599	10
5	562.28717	281.64722	188.10057	V	1144.62500	572.81614	382.21318	9
6	633.32429	317.16578	211.77961	A	1045.55658	523.28193	349.19038	8
7	761.38287	381.19507	254.46581	Q	974.51946	487.76337	325.51134	7
8	947.46219	474.23473	316.49225	W	846.46088	423.73408	282.82514	6
9	1060.54626	530.77677	354.18694	L	660.38156	330.69442	220.79870	5
10	1188.60484	594.80606	396.87313	Q	547.29749	274.15238	183.10401	4
11	1285.65761	643.33244	429.22405	P	419.23891	210.12309	140.41782	3
12	1422.71652	711.86190	474.91036	H	322.18614	161.59671	108.06690	2
13				R-Label:13C(6)15N(4)	185.12723	93.06725	62.38059	1

LDSFVAQWLQPHR

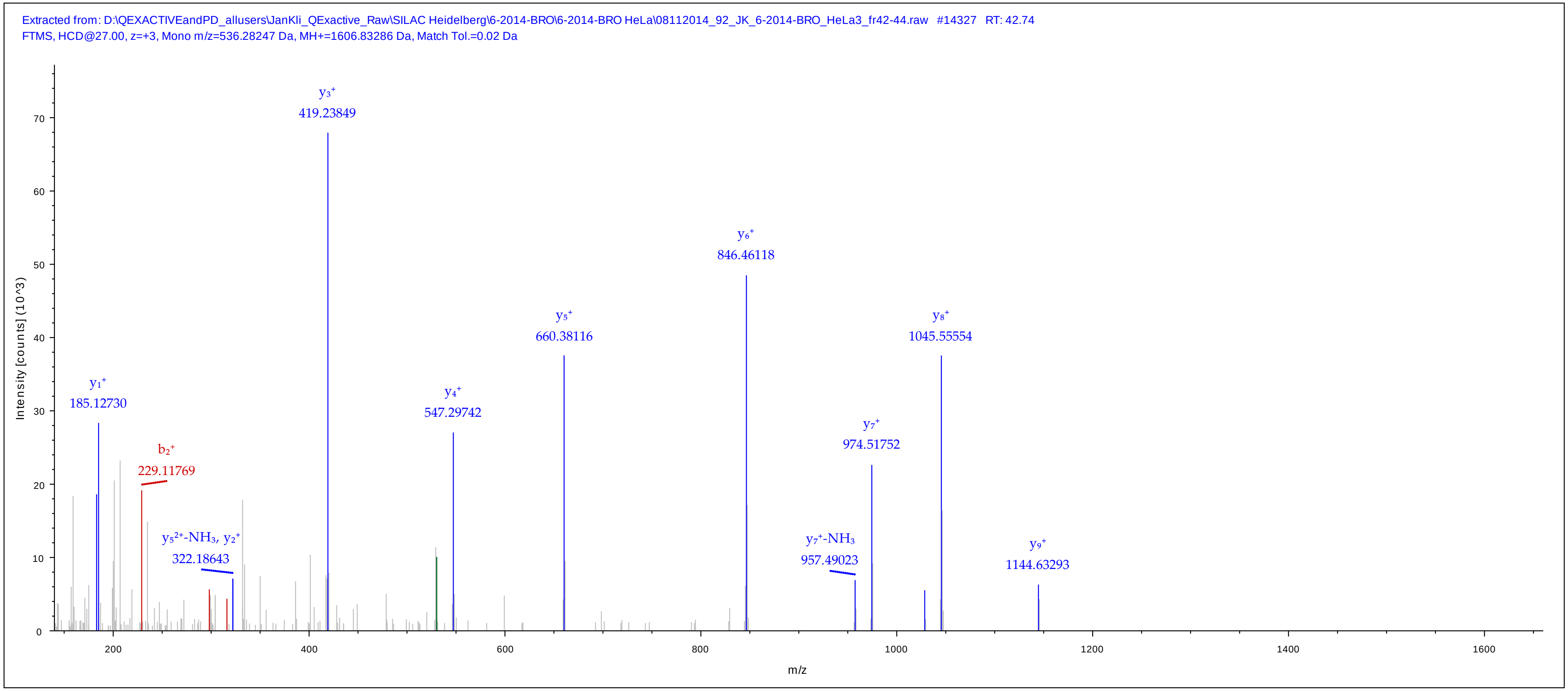

LDSFVAQWLQPHR

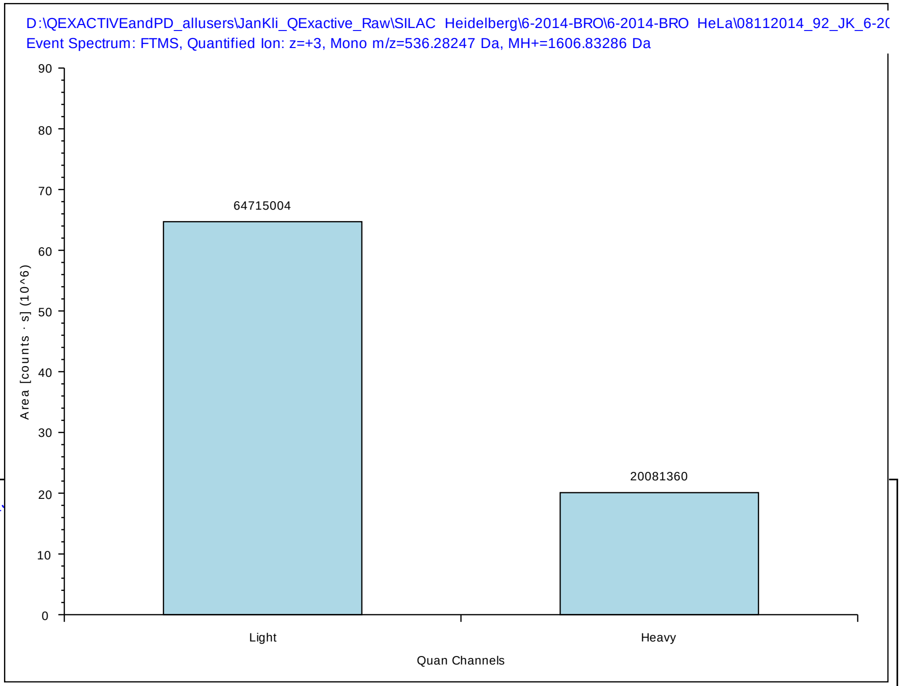

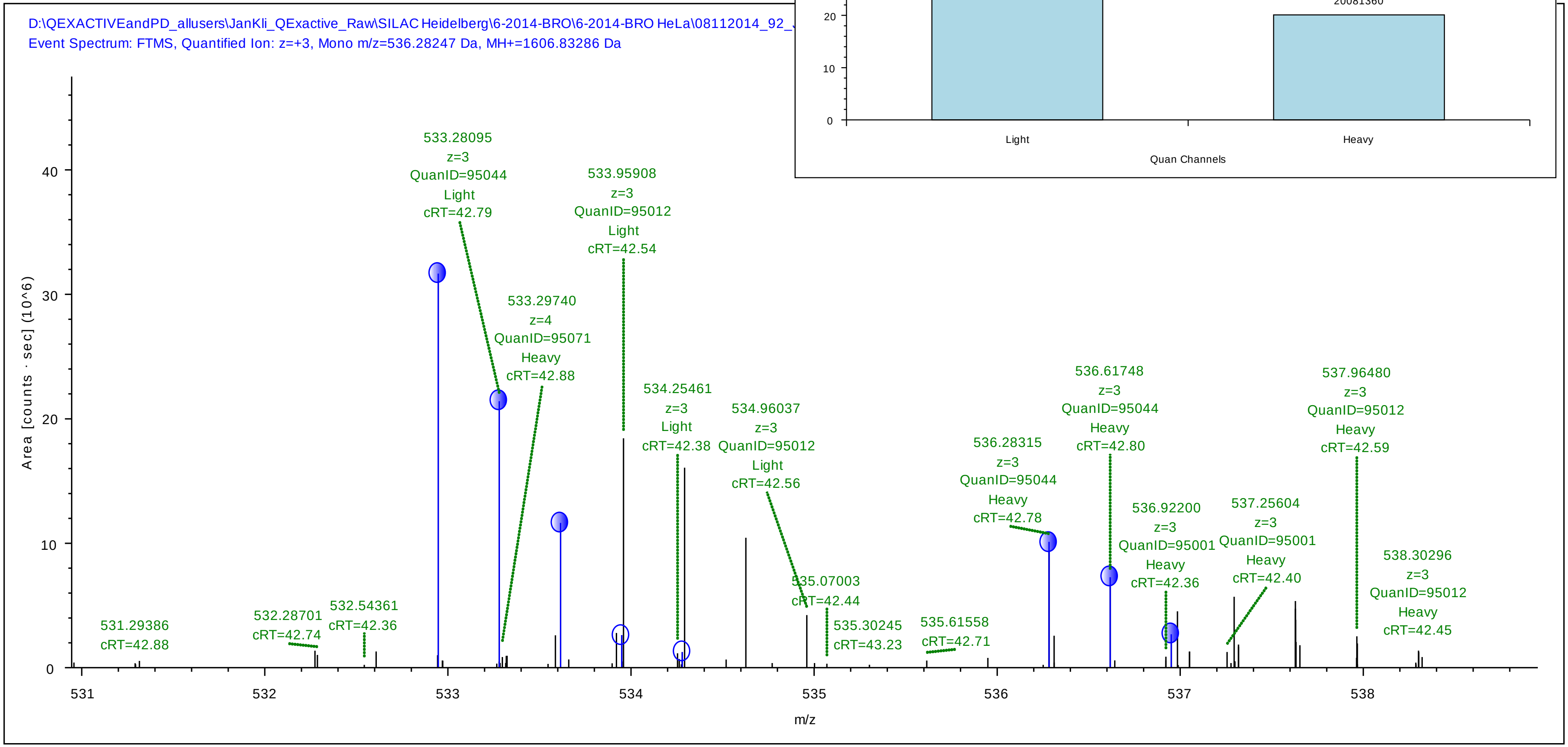

Sequence: QQIEDQQGLPK, K11-Label:13C(6)15N(2) (8.01420 Da)
Charge: +2, Monoisotopic m/z: 646.34038 Da (+0.11 mmu/+0.17 ppm), MH+: 1291.67348 Da, RT: 20.16 min,
Identified with: Mascot (v1.30); IonScore:31, Exp Value:2.1E-001, Ions matched by search engine: 8/102
Fragment match tolerance used for search: 0.02 Da

#1	b^+	b^{2+}	Seq.	y^+	y^{2+}	#2
1	129.06586	65.03657	Q			11
2	257.12444	129.06586	Q	1163.61468	582.31098	10
3	370.20851	185.60789	I	1035.55610	518.28169	9
4	499.25111	250.12919	E	922.47203	461.73965	8
5	614.27806	307.64267	D	793.42943	397.21835	7
6	742.33664	371.67196	Q	678.40248	339.70488	6
7	870.39522	435.70125	Q	550.34390	275.67559	5
8	927.41669	464.21198	G	422.28532	211.64630	4
9	1040.50076	520.75402	L	365.26385	183.13556	3
10	1137.55353	569.28040	P	252.17978	126.59353	2
11			K-Label:13C(6)15N(2)	155.12701	78.06714	1

QQIEDQQGLPK

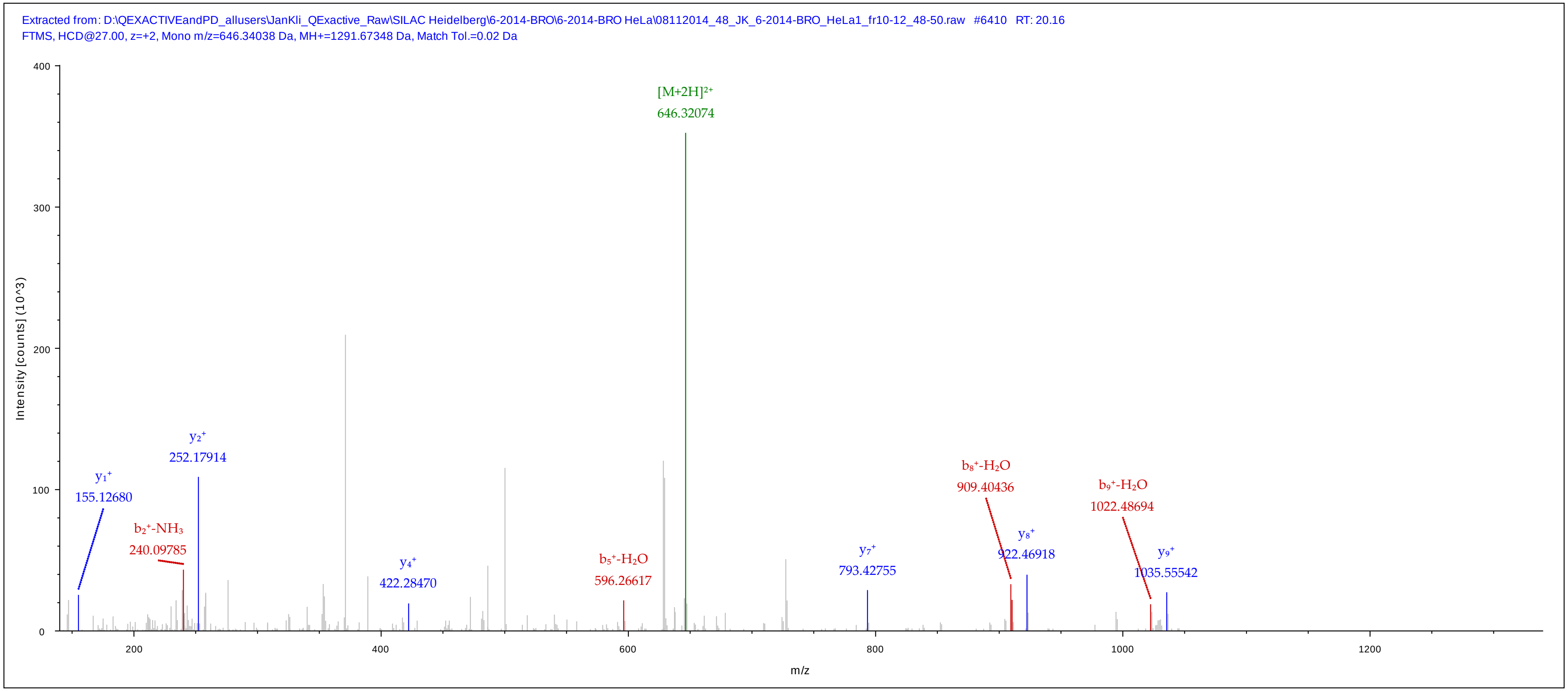

QQIEDQQGLPK

Not used for quantification
(no quantification values)

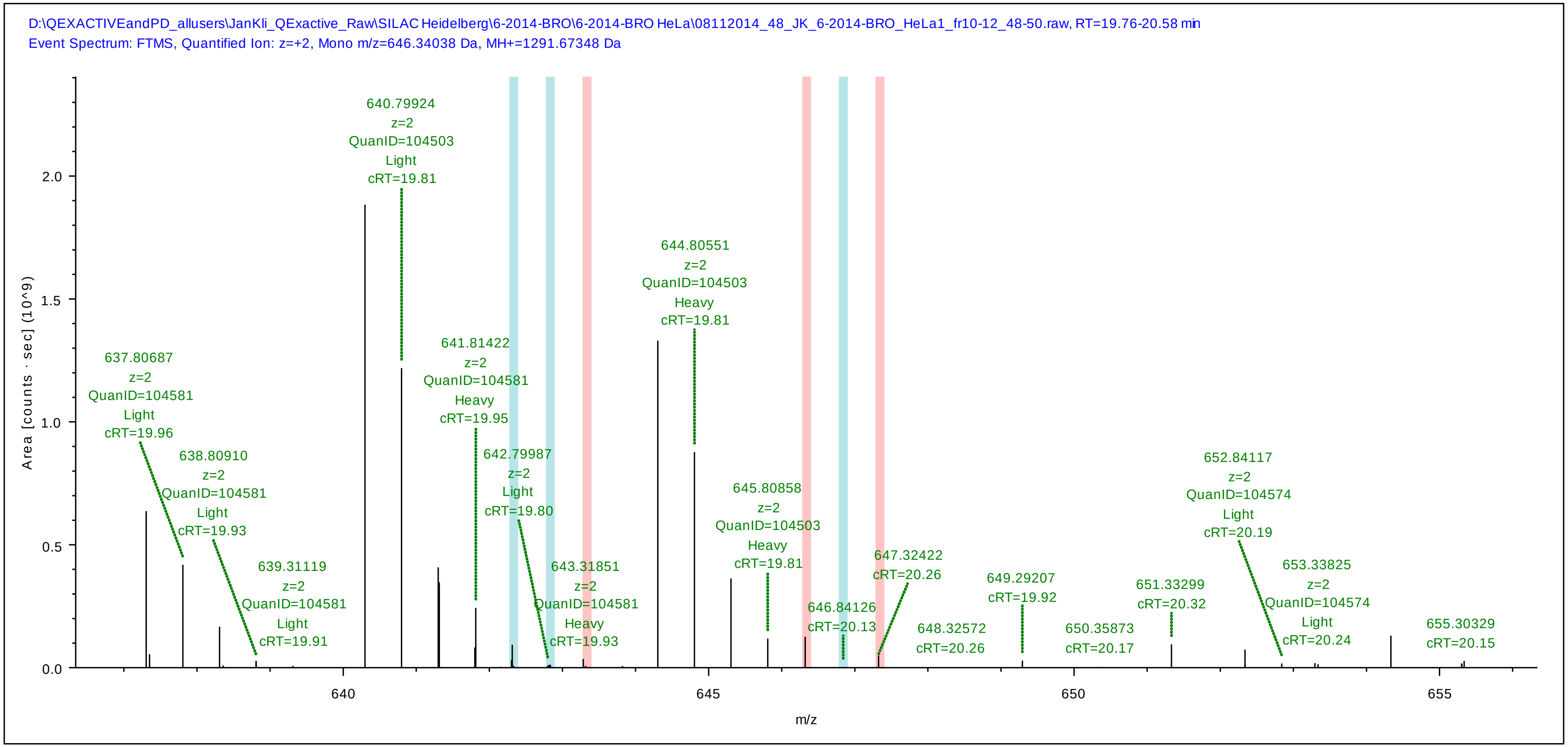

Sequence: GYPDFNLIVNPYEPIRK, Charge: +3,   Monoisotopic m/z: 679.02502 Da (-1.43 mmu/-2.11 ppm),   MH+: 2035.06052 Da,   RT: 43.65 min,
Identified with: Mascot (v1.30); IonScore:25, Exp Value:1.0E+000, Ions matched by search engine: 6/168
Fragment match tolerance used for search: 0.02 Da
Fragments used for search: b; b-H_2O; b-NH_3; y; y-H_2O; y-NH_3

#1	b^+	b^{2+}	b^{3+}	Seq.	y^+	y^{2+}	y^{3+}	#2
1	58.02875	29.51801	20.01443	G				17
2	221.09207	111.04967	74.36887	Y	1978.04334	989.52531	660.01930	16
3	318.14484	159.57606	106.71980	P	1814.98002	907.99365	605.66486	15
4	433.17179	217.08953	145.06211	D	1717.92725	859.46726	573.31393	14
5	580.24021	290.62374	194.08492	F	1602.90030	801.95379	534.97162	13
6	694.28314	347.64521	232.09923	N	1455.83188	728.41958	485.94881	12
7	807.36721	404.18724	269.79392	L	1341.78895	671.39811	447.93450	11
8	920.45128	460.72928	307.48861	I	1228.70488	614.85608	410.23981	10
9	1019.51970	510.26349	340.51142	V	1115.62081	558.31404	372.54512	9
10	1133.56263	567.28495	378.52573	N	1016.55239	508.77983	339.52231	8
11	1230.61540	615.81134	410.87665	P	902.50946	451.75837	301.50800	7
12	1393.67872	697.34300	465.23109	Y	805.45669	403.23198	269.15708	6
13	1522.72132	761.86430	508.24529	E	642.39337	321.70032	214.80264	5
14	1619.77409	810.39068	540.59621	P	513.35077	257.17902	171.78844	4
15	1732.85816	866.93272	578.29090	I	416.29800	208.65264	139.43752	3
16	1888.95928	944.98328	630.32461	R	303.21393	152.11060	101.74283	2
17				K	147.11281	74.06004	49.70912	1

GYPDFNLIVNPYEPIRK

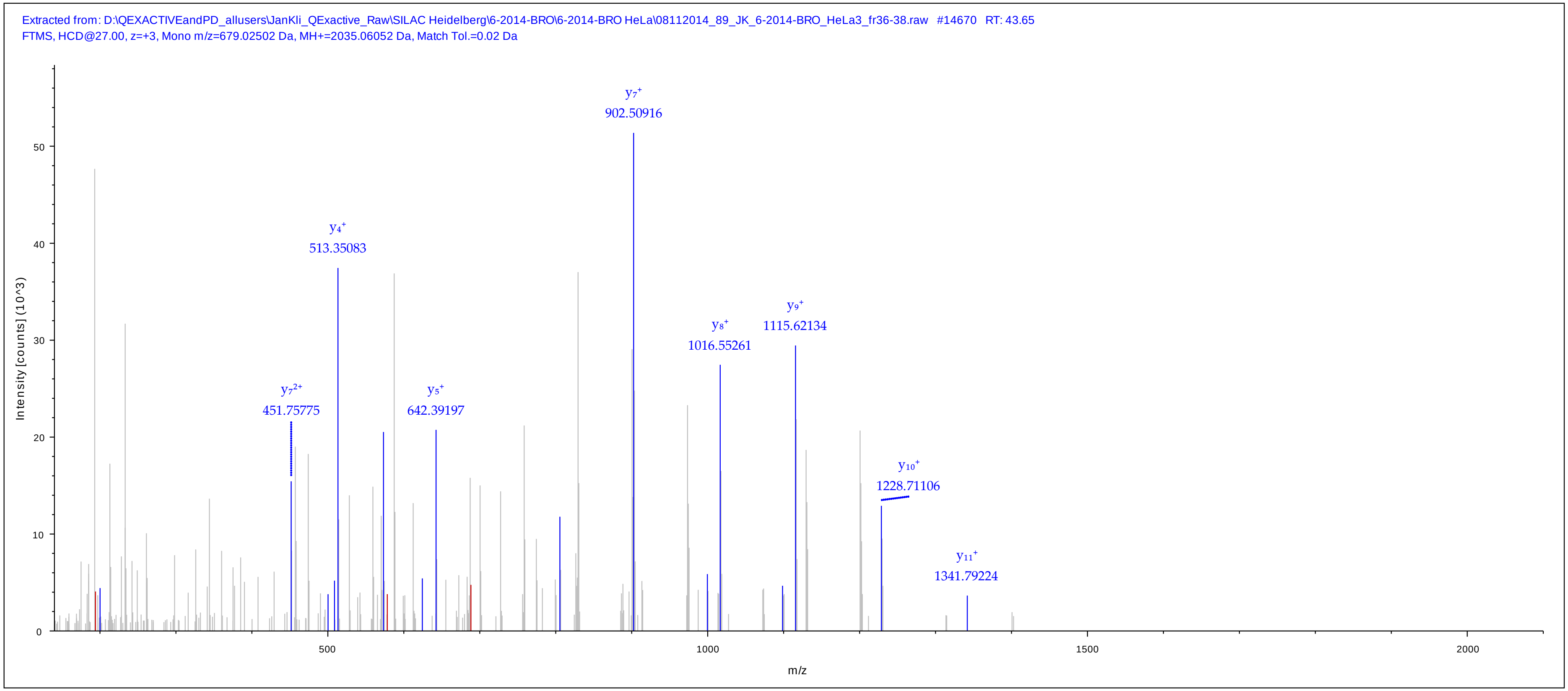

GYPDFNLIVNPYEPIRK

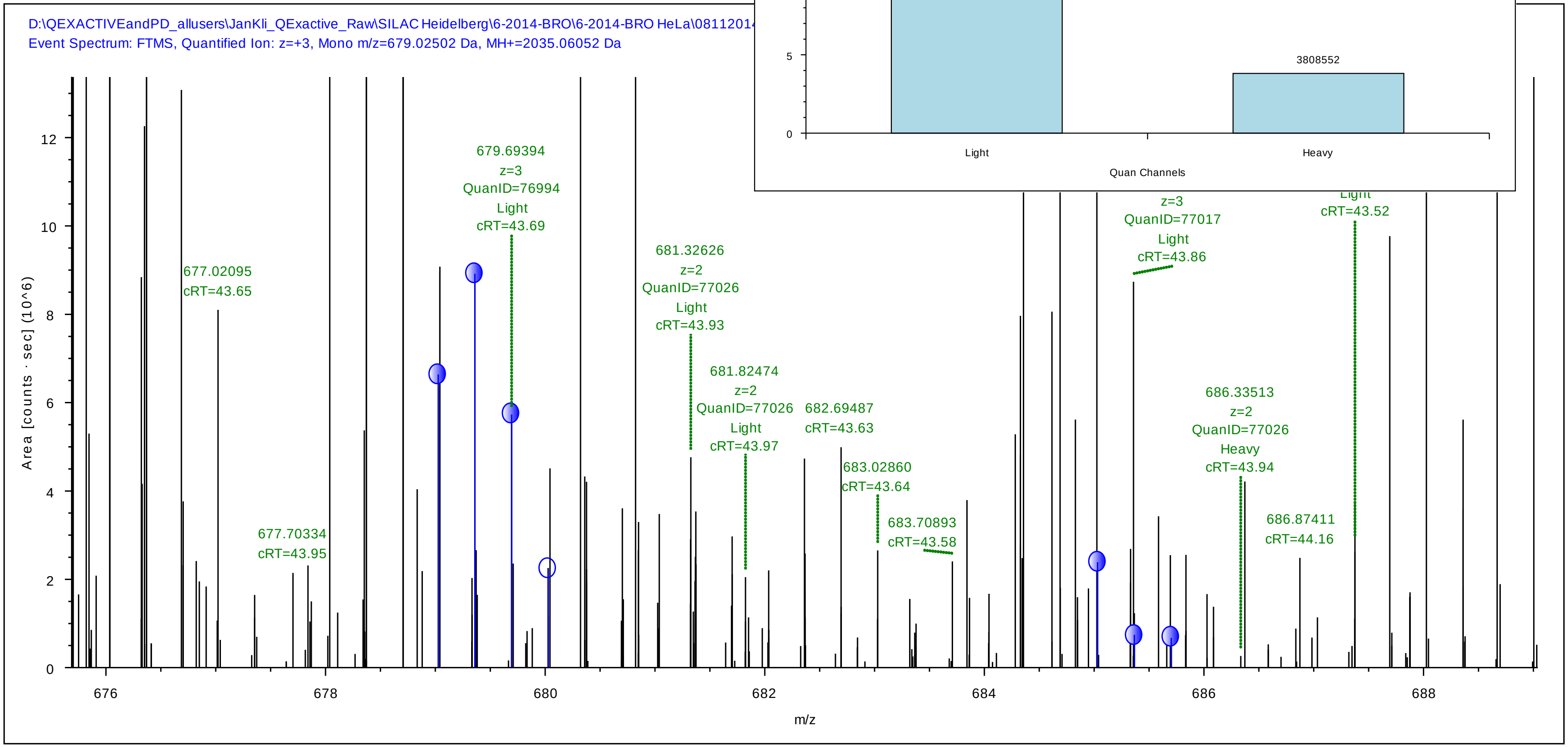

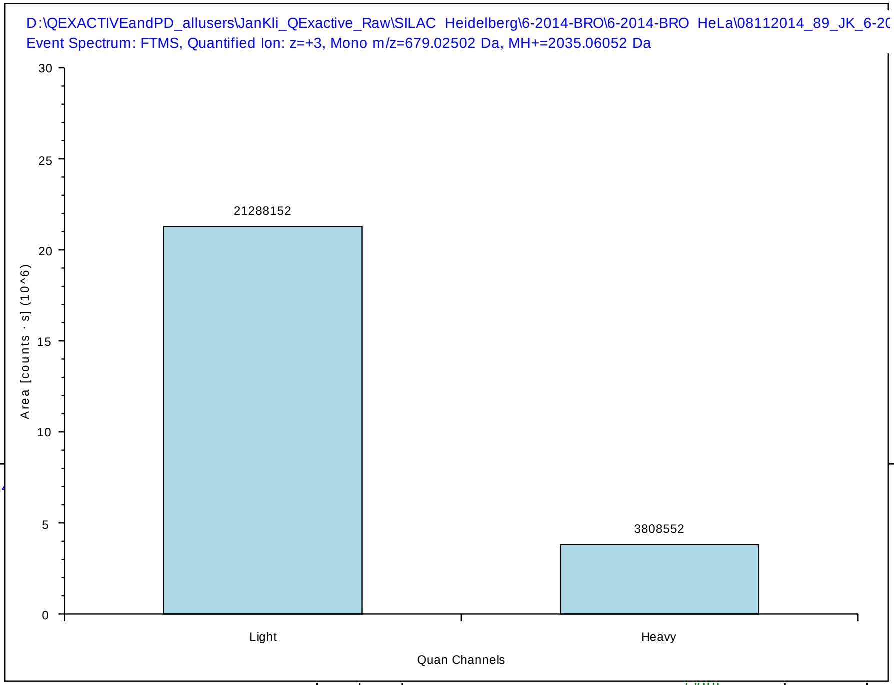

Sequence: ERPIILDPADPTLNVAEGYR, Charge: +3, Monoisotopic m/z: 747.05951 Da (-2.62 mmu/-3.51 ppm), MH+: 2239.16397 Da, RT: 40.38 min,
Identified with: Mascot (v1.30); IonScore:86, Exp Value:7.3E-007, Ions matched by search engine: 16/220
Fragment match tolerance used for search: 0.02 Da
Fragments used for search: b; b-H_2O; b-NH_3; y; y-H_2O; y-NH_3

#1	b^+	b^{2+}	b^{3+}	Seq.	y^+	y^{2+}	y^{3+}	#2
1	130.04988	65.52858	44.02148	E				20
2	286.15100	143.57914	96.05518	R	2110.12923	1055.56825	704.04793	19
3	383.20377	192.10552	128.40611	P	1954.02811	977.51769	652.01422	18
4	496.28784	248.64756	166.10080	I	1856.97534	928.99131	619.66330	17
5	609.37191	305.18959	203.79549	I	1743.89127	872.44927	581.96861	16
6	722.45598	361.73163	241.49018	L	1630.80720	815.90724	544.27392	15
7	837.48293	419.24510	279.83249	D	1517.72313	759.36520	506.57923	14
8	934.53570	467.77149	312.18342	P	1402.69618	701.85173	468.23691	13
9	1005.57282	503.29005	335.86246	A	1305.64341	653.32534	435.88599	12
10	1120.59977	560.80352	374.20477	D	1234.60629	617.80678	412.20695	11
11	1217.65254	609.32991	406.55570	P	1119.57934	560.29331	373.86463	10
12	1318.70022	659.85375	440.23826	T	1022.52657	511.76692	341.51371	9
13	1431.78429	716.39578	477.93295	L	921.47889	461.24308	307.83115	8
14	1545.82722	773.41725	515.94726	N	808.39482	404.70105	270.13646	7
15	1644.89564	822.95146	548.97006	V	694.35189	347.67958	232.12215	6
16	1715.93276	858.47002	572.64910	A	595.28347	298.14537	199.09934	5
17	1844.97536	922.99132	615.66330	E	524.24635	262.62681	175.42030	4
18	1901.99683	951.50205	634.67046	G	395.20375	198.10551	132.40610	3
19	2065.06015	1033.03371	689.02490	Y	338.18228	169.59478	113.39894	2
20				R	175.11896	88.06312	59.04450	1

ERPIILDPADPTLNVAEGYR

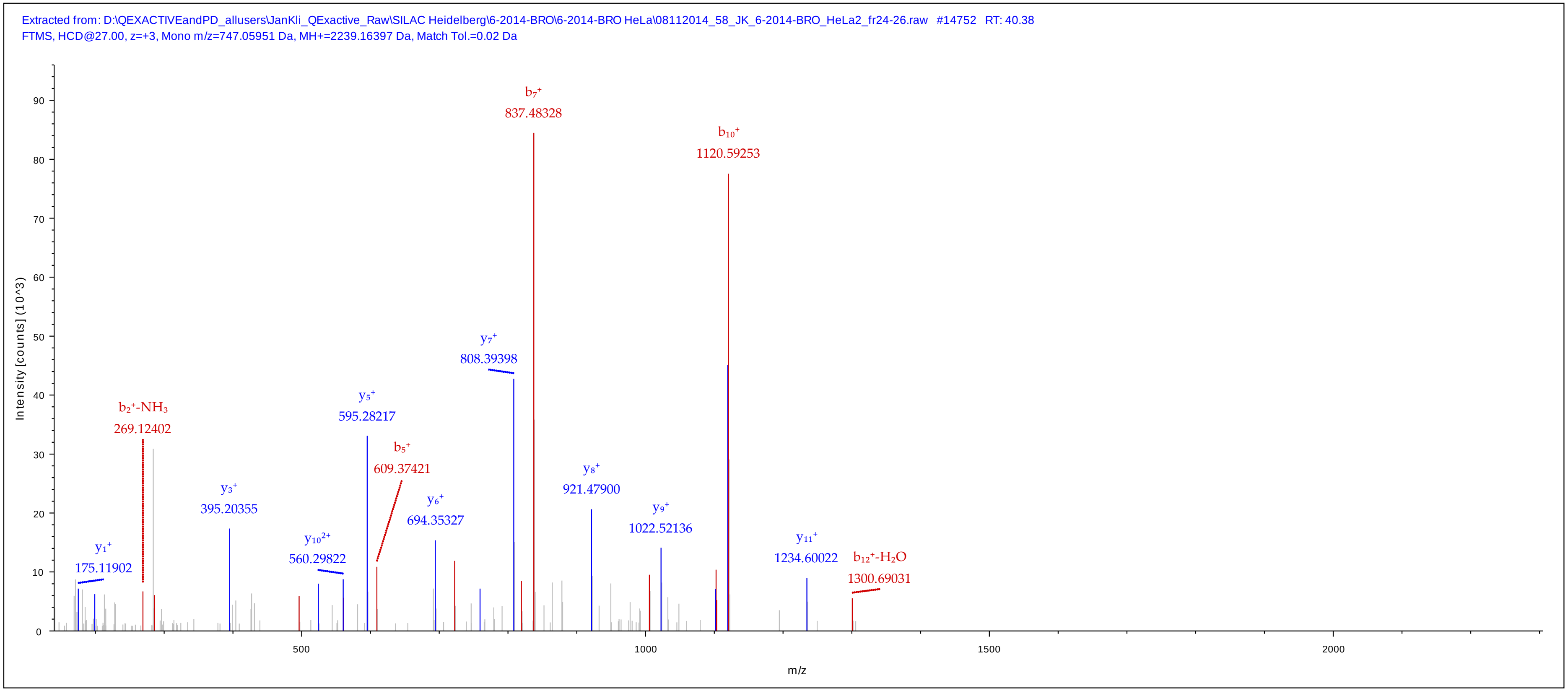

ERPIILDPADPTLNVAEGYR

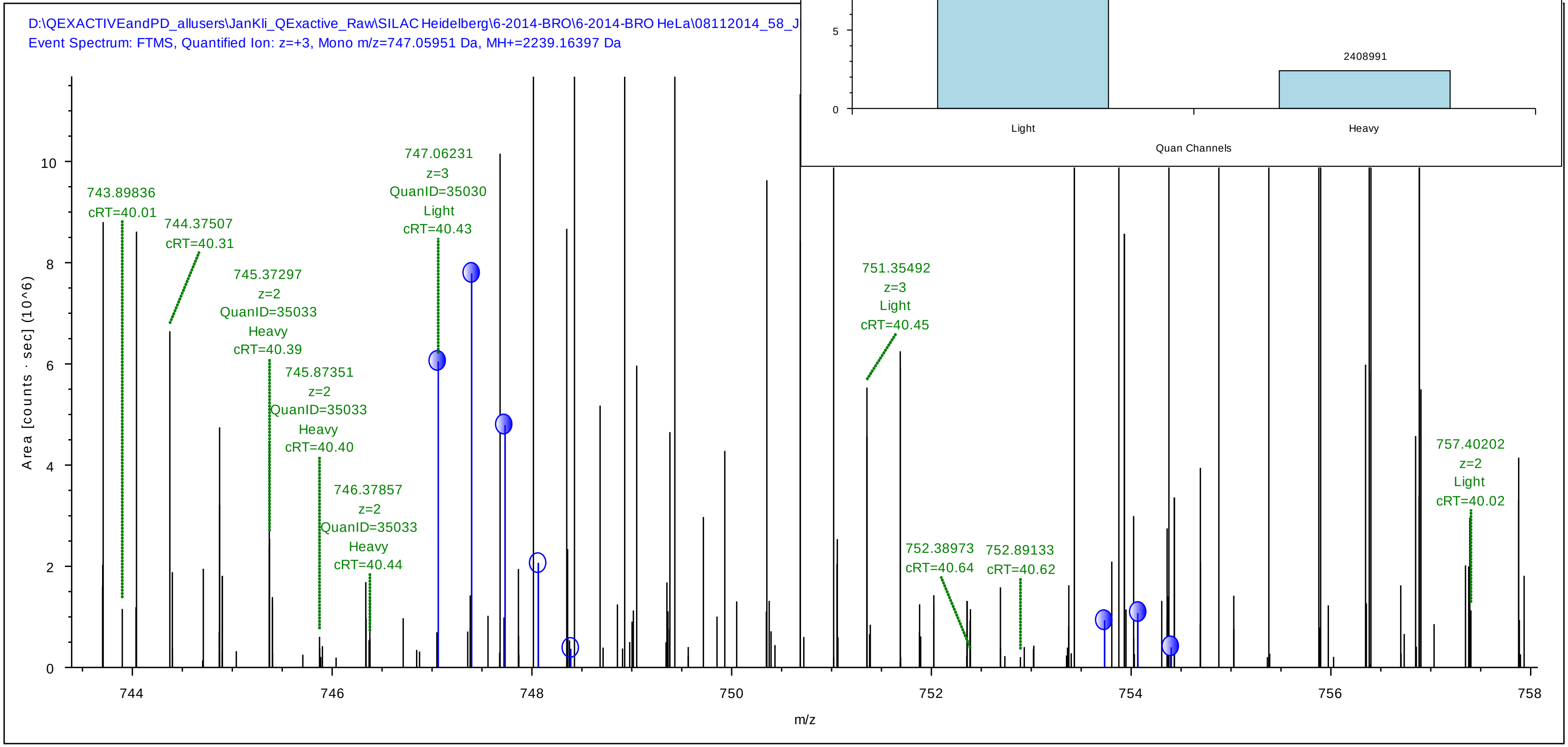

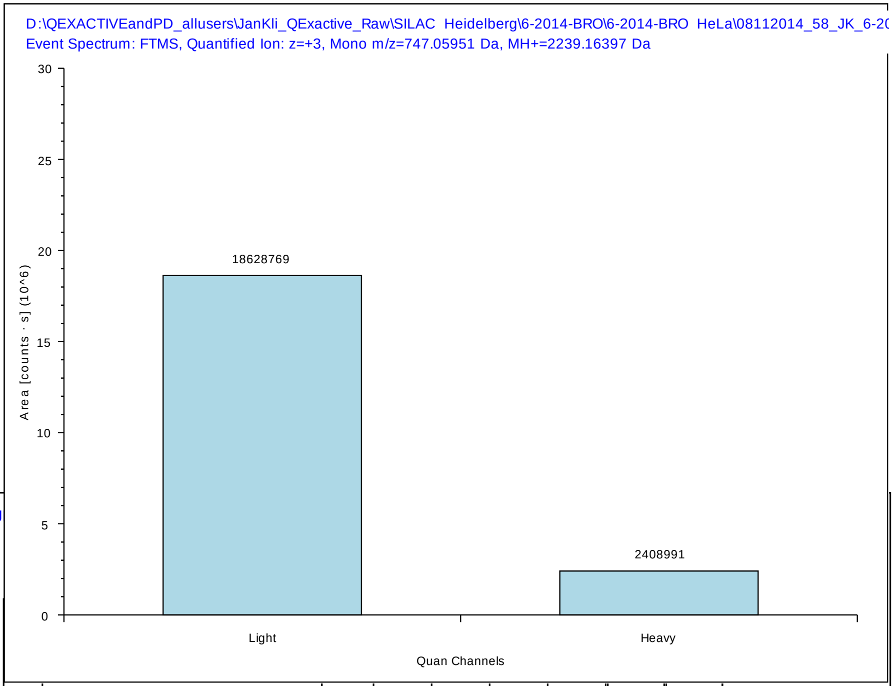

Sequence: NPDGGSYAYAINPNSFILGLK, Charge: +2, Monoisotopic m/z: 1106.05737 Da (-0.34 mmu/-0.3 ppm), MH+: 2211.10747 Da, RT: 51.35 min,
Identified with: Mascot (v1.30); IonScore:73, Exp Value:1.6E-005, Ions matched by search engine: 12/224
Fragment match tolerance used for search: 0.02 Da
Fragments used for search: b; b-H_2O; b-NH_3; y; y-H_2O; y-NH_3

#1	b^+	b^{2+}	Seq.	y^+	y^{2+}	#2
1	115.05021	58.02874	N			21
2	212.10298	106.55513	P	2097.06521	1049.03624	20
3	327.12993	164.06860	D	2000.01244	1000.50986	19
4	384.15140	192.57934	G	1884.98549	942.99638	18
5	441.17287	221.09007	G	1827.96402	914.48565	17
6	528.20490	264.60609	S	1770.94255	885.97491	16
7	691.26822	346.13775	Y	1683.91052	842.45890	15
8	762.30534	381.65631	A	1520.84720	760.92724	14
9	925.36866	463.18797	Y	1449.81008	725.40868	13
10	996.40578	498.70653	A	1286.74676	643.87702	12
11	1109.48985	555.24856	I	1215.70964	608.35846	11
12	1223.53278	612.27003	N	1102.62557	551.81642	10
13	1320.58555	660.79641	P	988.58264	494.79496	9
14	1434.62848	717.81788	N	891.52987	446.26857	8
15	1521.66051	761.33389	S	777.48694	389.24711	7
16	1668.72893	834.86810	F	690.45491	345.73109	6
17	1781.81300	891.41014	I	543.38649	272.19688	5
18	1894.89707	947.95217	L	430.30242	215.65485	4
19	1951.91854	976.46291	G	317.21835	159.11281	3
20	2065.00261	1033.00494	L	260.19688	130.60208	2
21			K	147.11281	74.06004	1

NPDGGSYAYAINPNSFILGLK

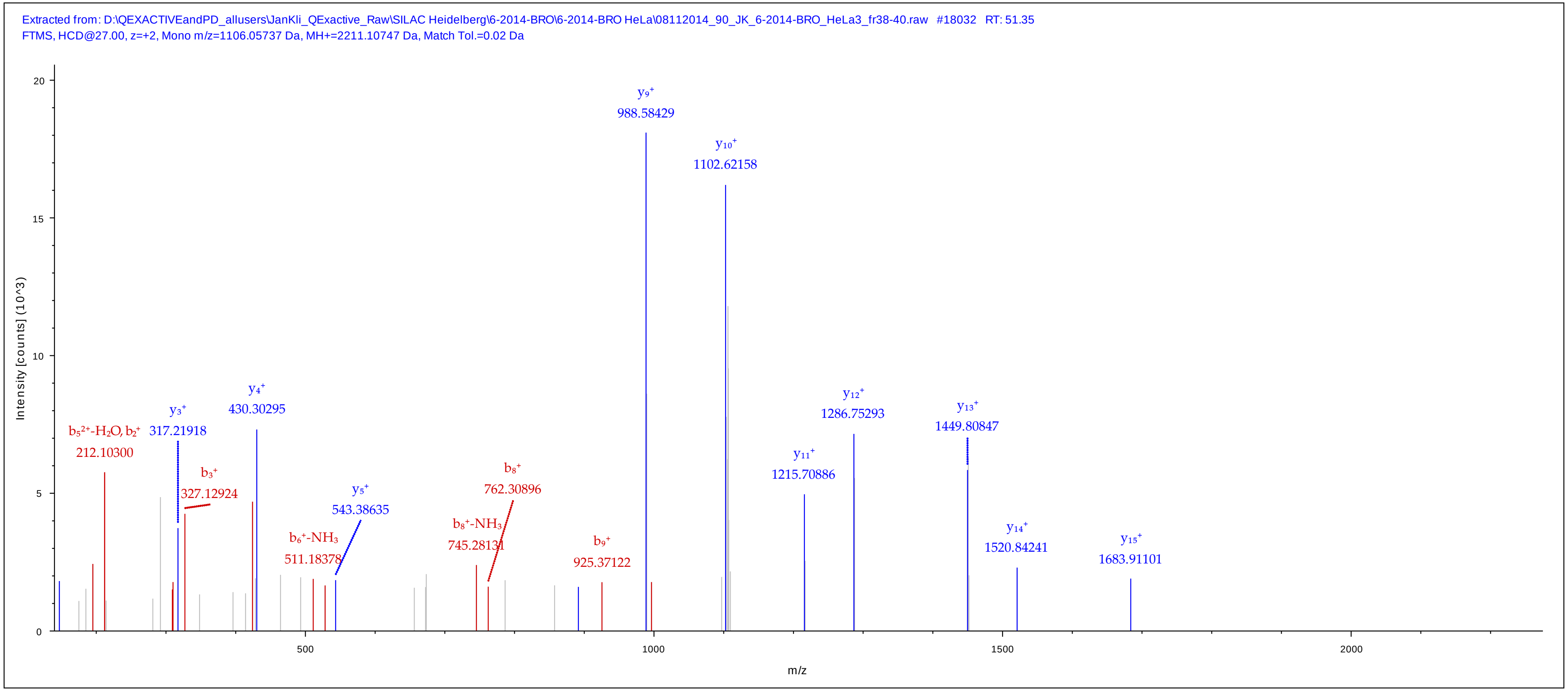

NPDGGSYAYAINPNSFILGLK

Not used for quantification
(missing heavy channel)

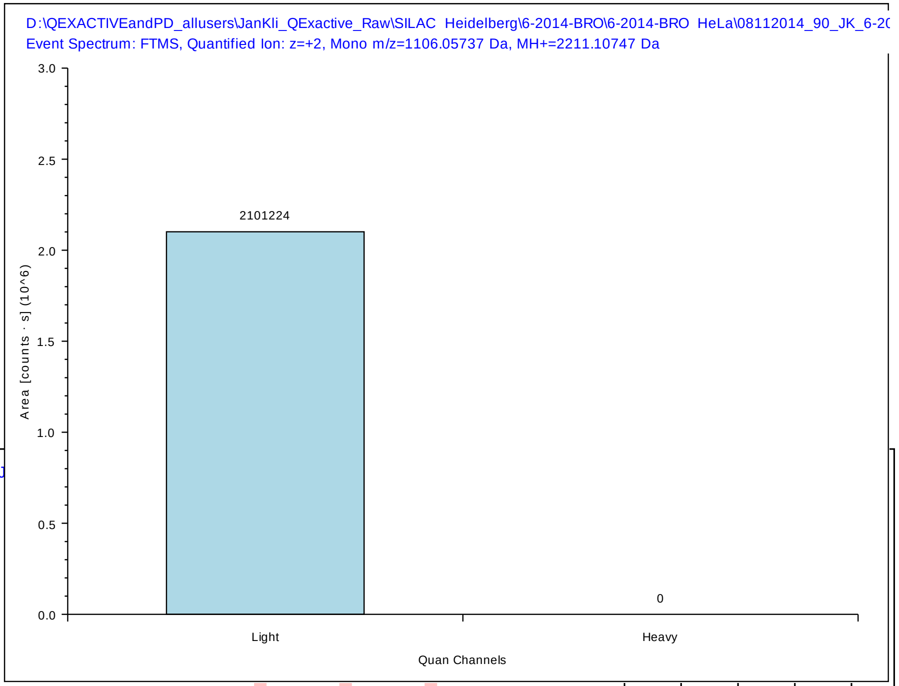

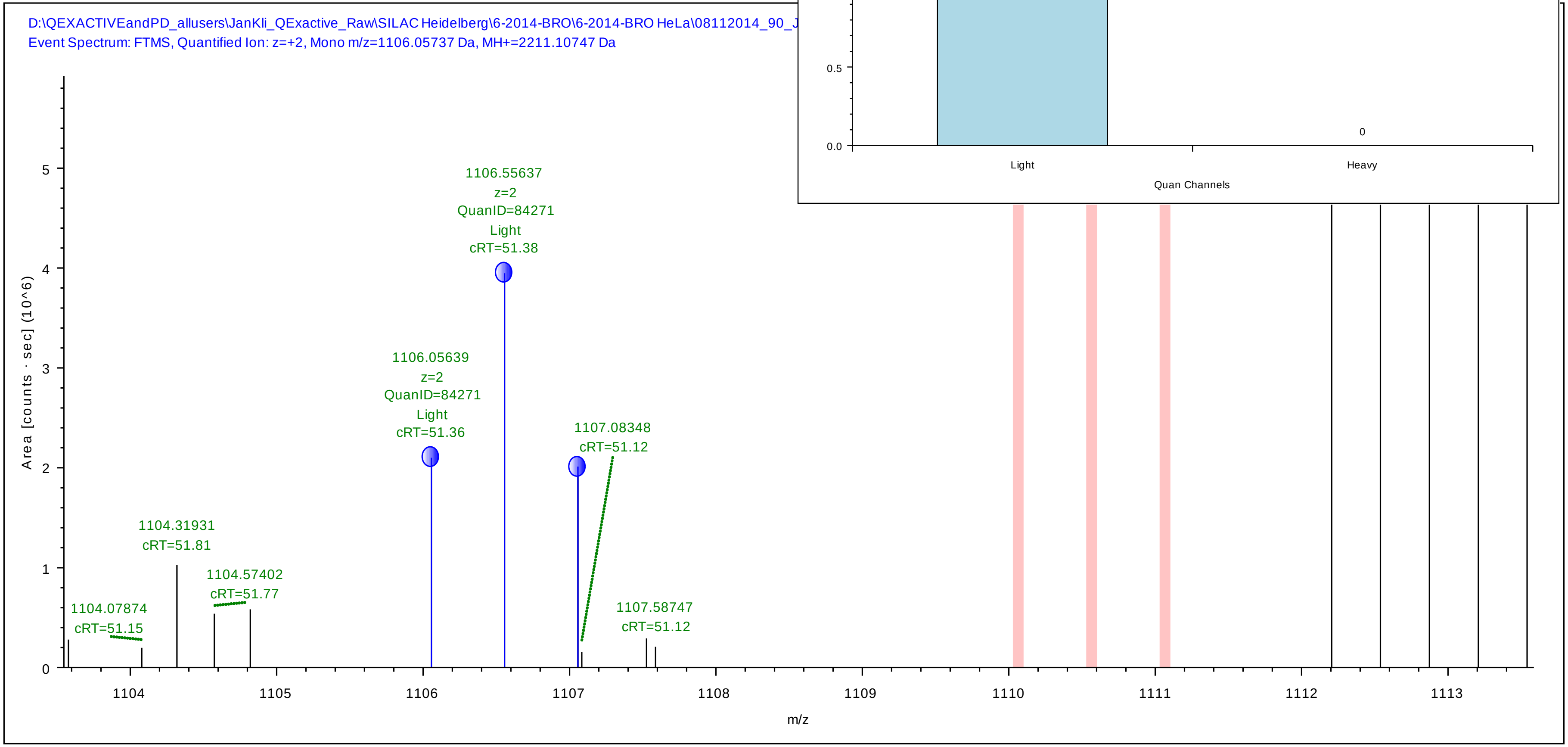

Sequence: GTAEPITVTIVPAYR, Charge: +2, Monoisotopic m/z: 794.44019 Da (-0.53 mmu/-0.67 ppm), MH+: 1587.87309 Da, RT: 38.60 min,
Identified with: Mascot (v1.30); IonScore:59, Exp Value:3.8E-004, Ions matched by search engine: 9/126
Fragment match tolerance used for search: 0.02 Da
Fragments used for search: b; b-H_2O; y; y-H_2O; y-NH_3

#1	b^+	b^{2+}	Seq.	y^+	y^{2+}	#2
1	58.02875	29.51801	G			15
2	159.07643	80.04185	T	1530.85268	765.92998	14
3	230.11355	115.56041	A	1429.80500	715.40614	13
4	359.15615	180.08171	E	1358.76788	679.88758	12
5	456.20892	228.60810	P	1229.72528	615.36628	11
6	569.29299	285.15013	I	1132.67251	566.83989	10
7	670.34067	335.67397	T	1019.58844	510.29786	9
8	769.40909	385.20818	V	918.54076	459.77402	8
9	870.45677	435.73202	T	819.47234	410.23981	7
10	983.54084	492.27406	I	718.42466	359.71597	6
11	1082.60926	541.80827	V	605.34059	303.17393	5
12	1179.66203	590.33465	P	506.27217	253.63972	4
13	1250.69915	625.85321	A	409.21940	205.11334	3
14	1413.76247	707.38487	Y	338.18228	169.59478	2
15			R	175.11896	88.06312	1

GTAEPITVTIVPAYR

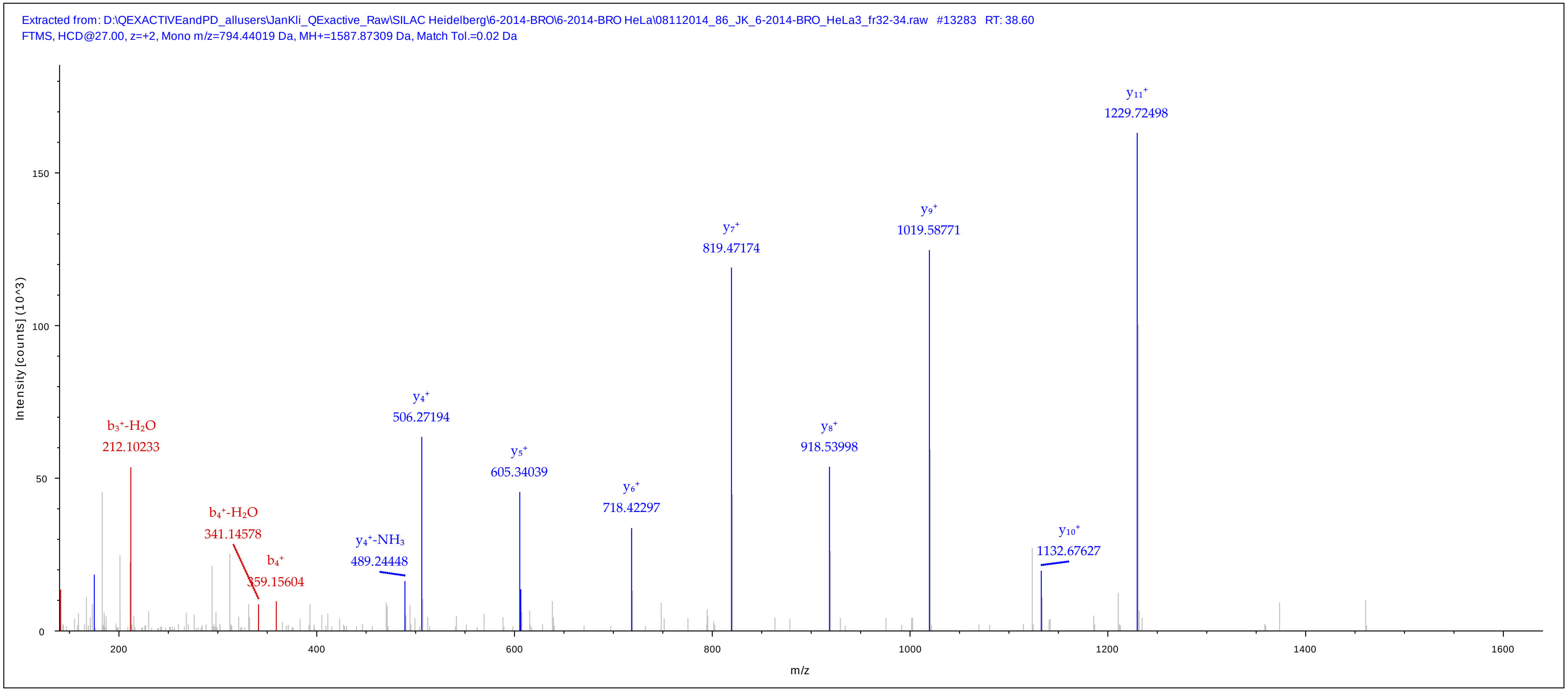

GTAEPITVTIVPAYR

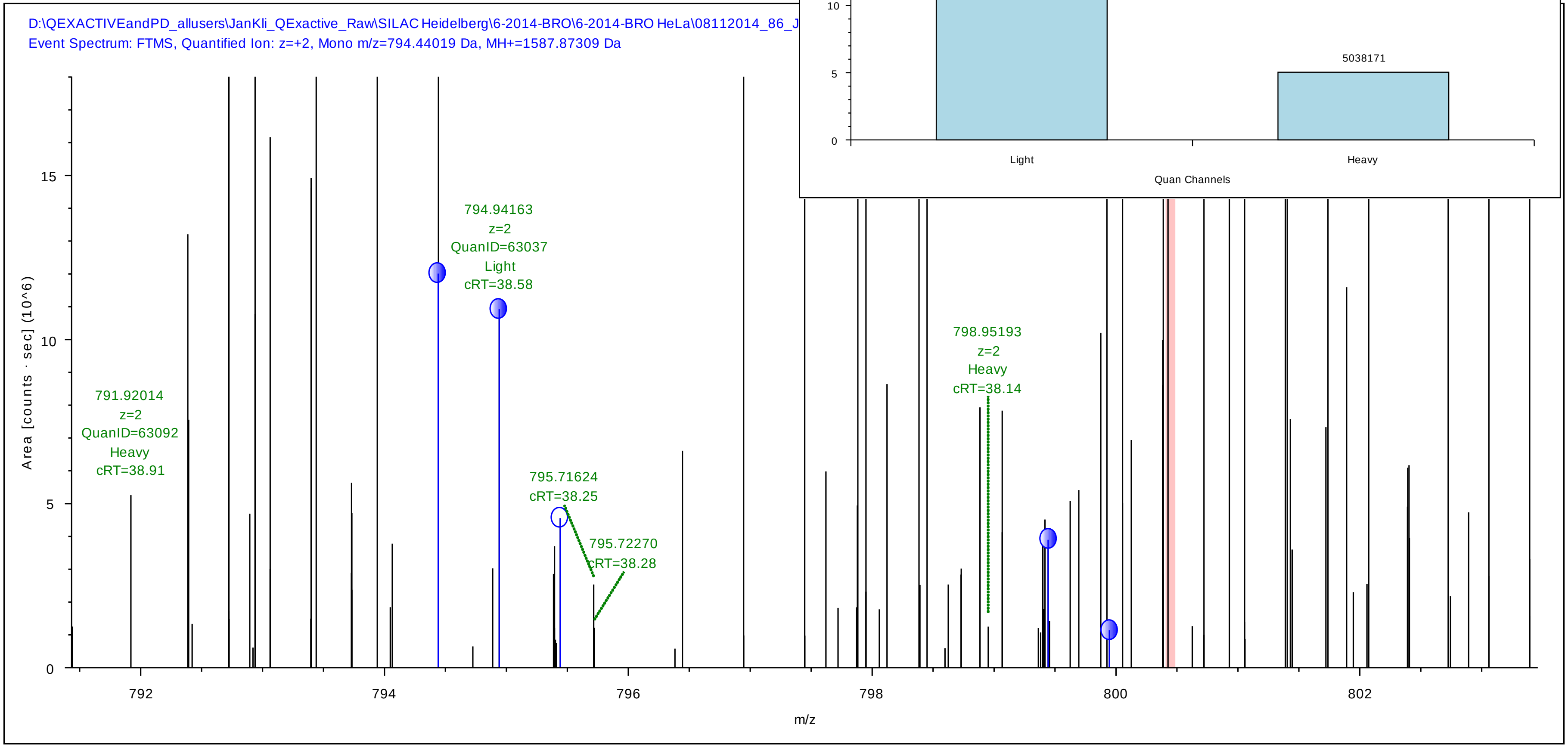

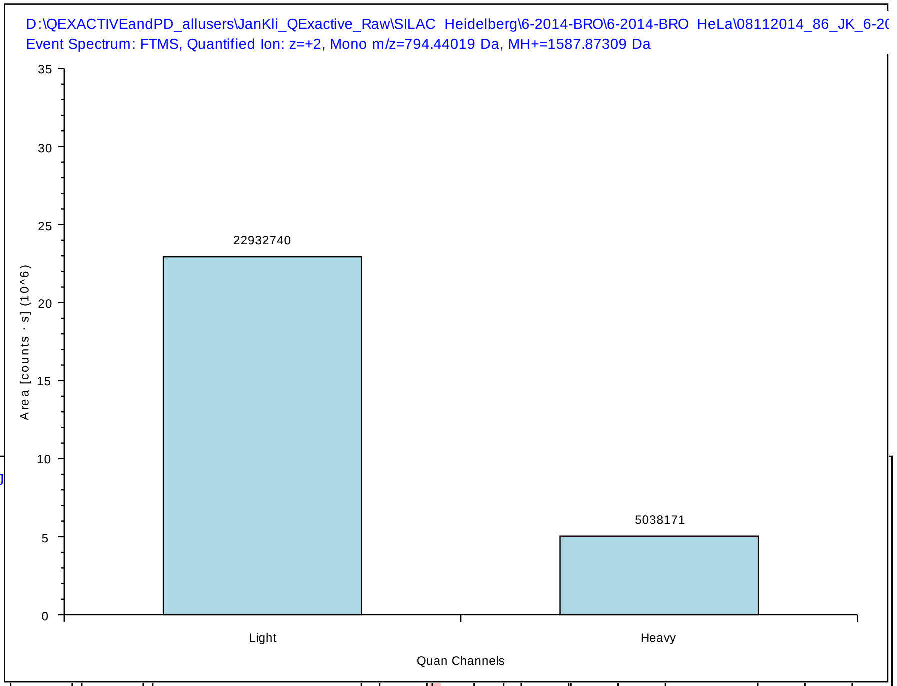

Sequence: EWKEEVLDAVR, Charge: +3, Monoisotopic m/z: 458.57251 Da (-1.02 mmu/-2.22 ppm), MH+: 1373.70298 Da, RT: 32.65 min,
Identified with: Mascot (v1.30); IonScore:49, Exp Value:3.5E-003, Ions matched by search engine: 6/110
Fragment match tolerance used for search: 0.02 Da
Fragments used for search: b; b-H_2O; b-NH_3; y; y-H_2O; y-NH_3

#1	b^+	b^{2+}	b^{3+}	Seq.	y^+	y^{2+}	y^{3+}	#2
1	130.04988	65.52858	44.02148	E				11
2	316.12920	158.56824	106.04792	W	1244.66343	622.83535	415.55933	10
3	444.22417	222.61572	148.74624	K	1058.58411	529.79569	353.53289	9
4	573.26677	287.13702	191.76044	E	930.48914	465.74821	310.83456	8
5	702.30937	351.65832	234.77464	E	801.44654	401.22691	267.82036	7
6	801.37779	401.19253	267.79745	V	672.40394	336.70561	224.80616	6
7	914.46186	457.73457	305.49214	L	573.33552	287.17140	191.78336	5
8	1029.48881	515.24804	343.83445	D	460.25145	230.62936	154.08867	4
9	1100.52593	550.76660	367.51349	A	345.22450	173.11589	115.74635	3
10	1199.59435	600.30081	400.53630	V	274.18738	137.59733	92.06731	2
11				R	175.11896	88.06312	59.04450	1

EWKEEVLDAVR

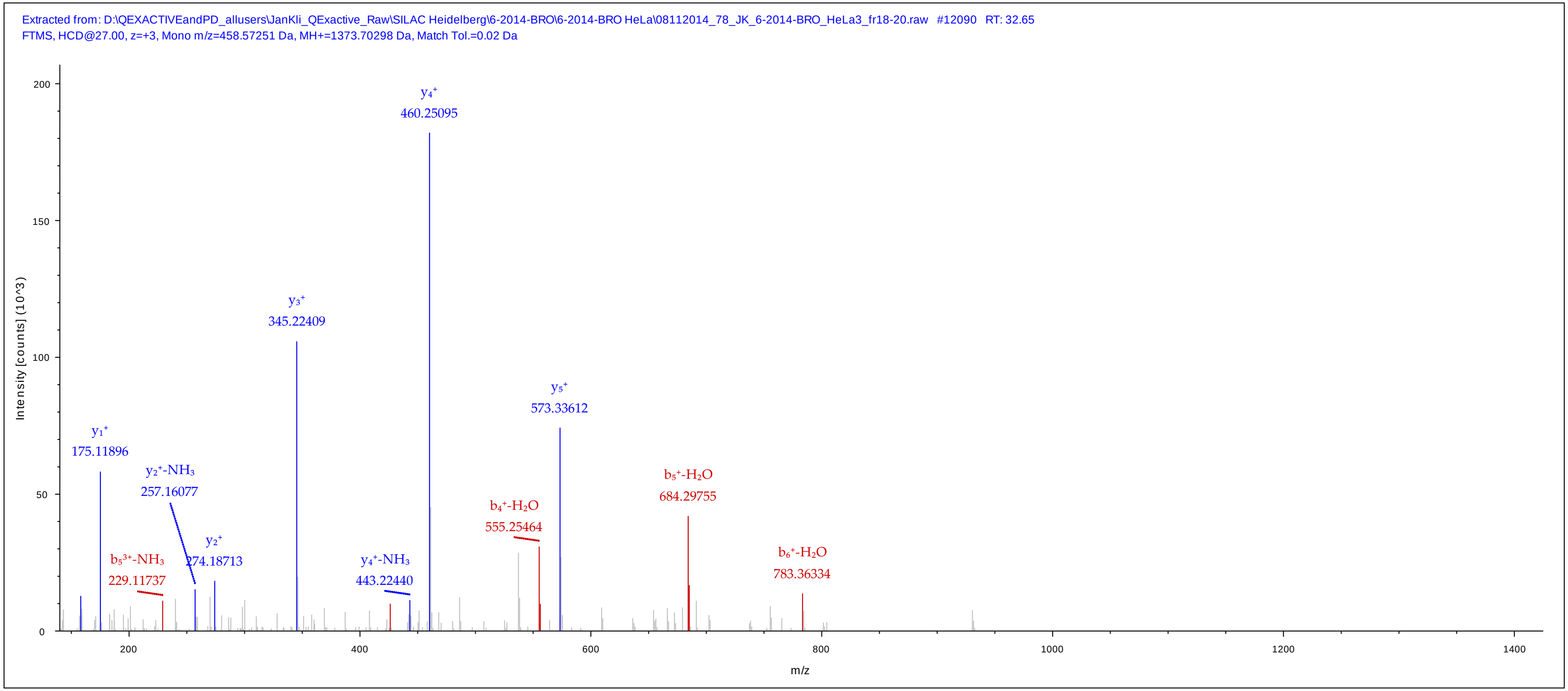

EWKEEVLDAVR

Not used for quantification (not enough quantification values)

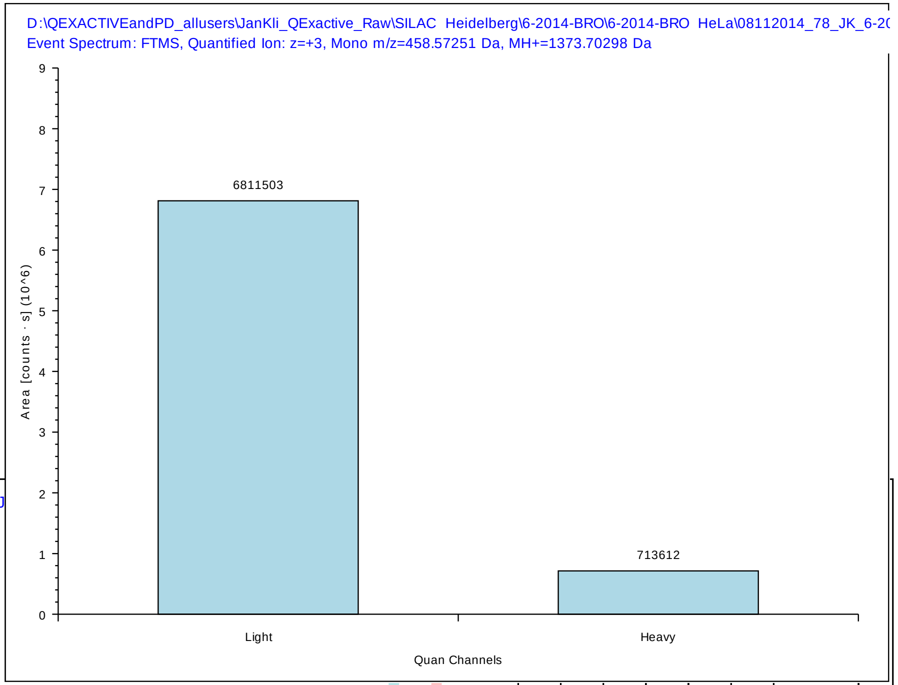

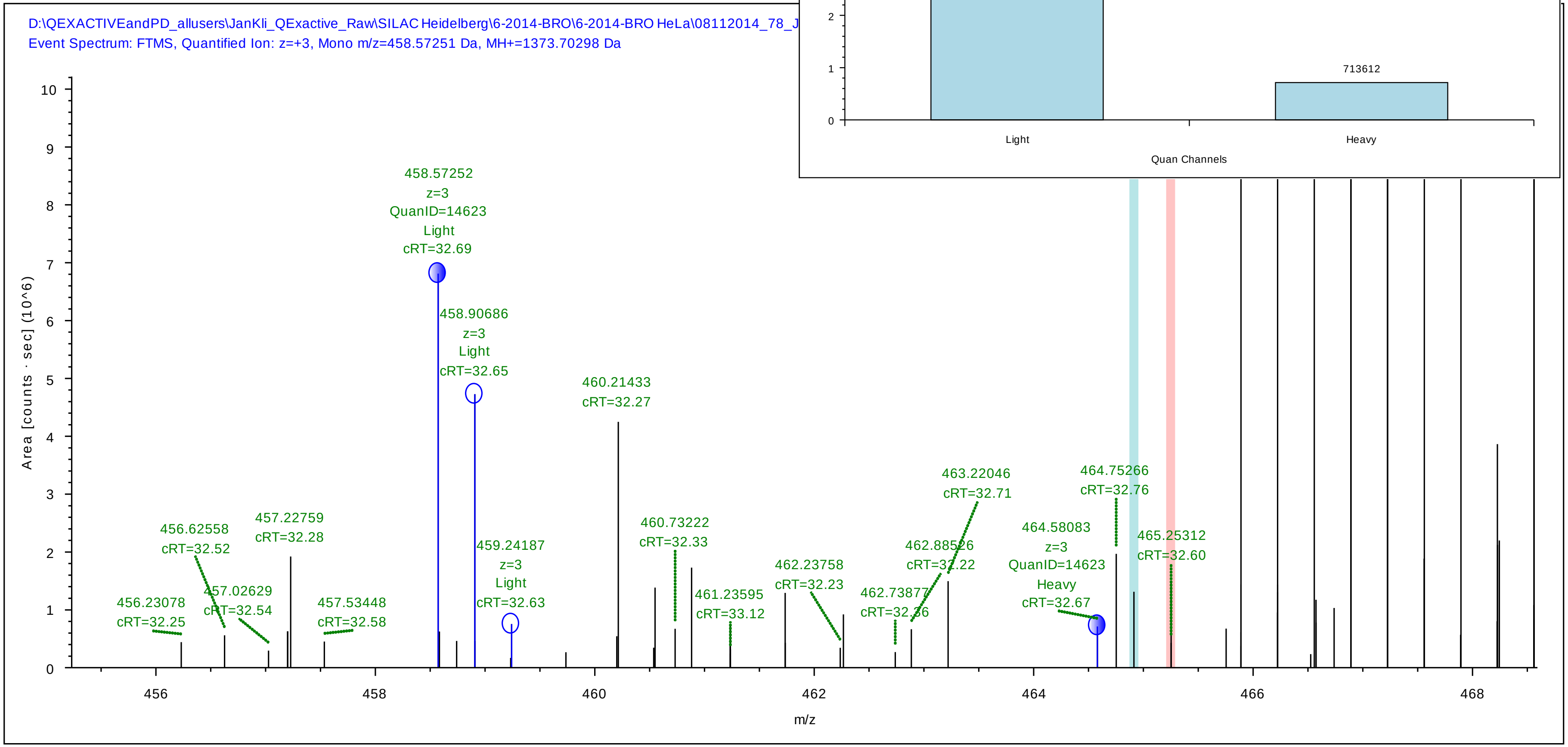

Sequence: ALGPSLPNSQPPPEVYVSLIK, Charge: +2, Monoisotopic m/z: 1103.60876 Da (-0.82 mmu/-0.74 ppm), MH+: 2206.21025 Da, RT: 46.83 min,
Identified with: Mascot (v1.30); IonScore:20, Exp Value:1.9E+000, Ions matched by search engine: 12/212
Fragment match tolerance used for search: 0.02 Da
Fragments used for search: b; b-H_2O; b-NH_3; y; y-H_2O; y-NH_3

#1	b^+	b^{2+}	Seq.	y^+	y^{2+}	#2
1	72.04440	36.52584	A			21
2	185.12847	93.06787	L	2135.17477	1068.09102	20
3	242.14994	121.57861	G	2022.09070	1011.54899	19
4	339.20271	170.10499	P	1965.06923	983.03825	18
5	426.23474	213.62101	S	1868.01646	934.51187	17
6	539.31881	270.16304	L	1780.98443	890.99585	16
7	636.37158	318.68943	P	1667.90036	834.45382	15
8	750.41451	375.71089	N	1570.84759	785.92743	14
9	837.44654	419.22691	S	1456.80466	728.90597	13
10	965.50512	483.25620	Q	1369.77263	685.38995	12
11	1062.55789	531.78258	P	1241.71405	621.36066	11
12	1159.61066	580.30897	P	1144.66128	572.83428	10
13	1256.66343	628.83535	P	1047.60851	524.30789	9
14	1385.70603	693.35665	E	950.55574	475.78151	8
15	1484.77445	742.89086	V	821.51314	411.26021	7
16	1647.83777	824.42252	Y	722.44472	361.72600	6
17	1746.90619	873.95673	V	559.38140	280.19434	5
18	1833.93822	917.47275	S	460.31298	230.66013	4
19	1947.02229	974.01478	L	373.28095	187.14411	3
20	2060.10636	1030.55682	I	260.19688	130.60208	2
21			K	147.11281	74.06004	1

ALGPSLPNSQPPPEVYVSLIK

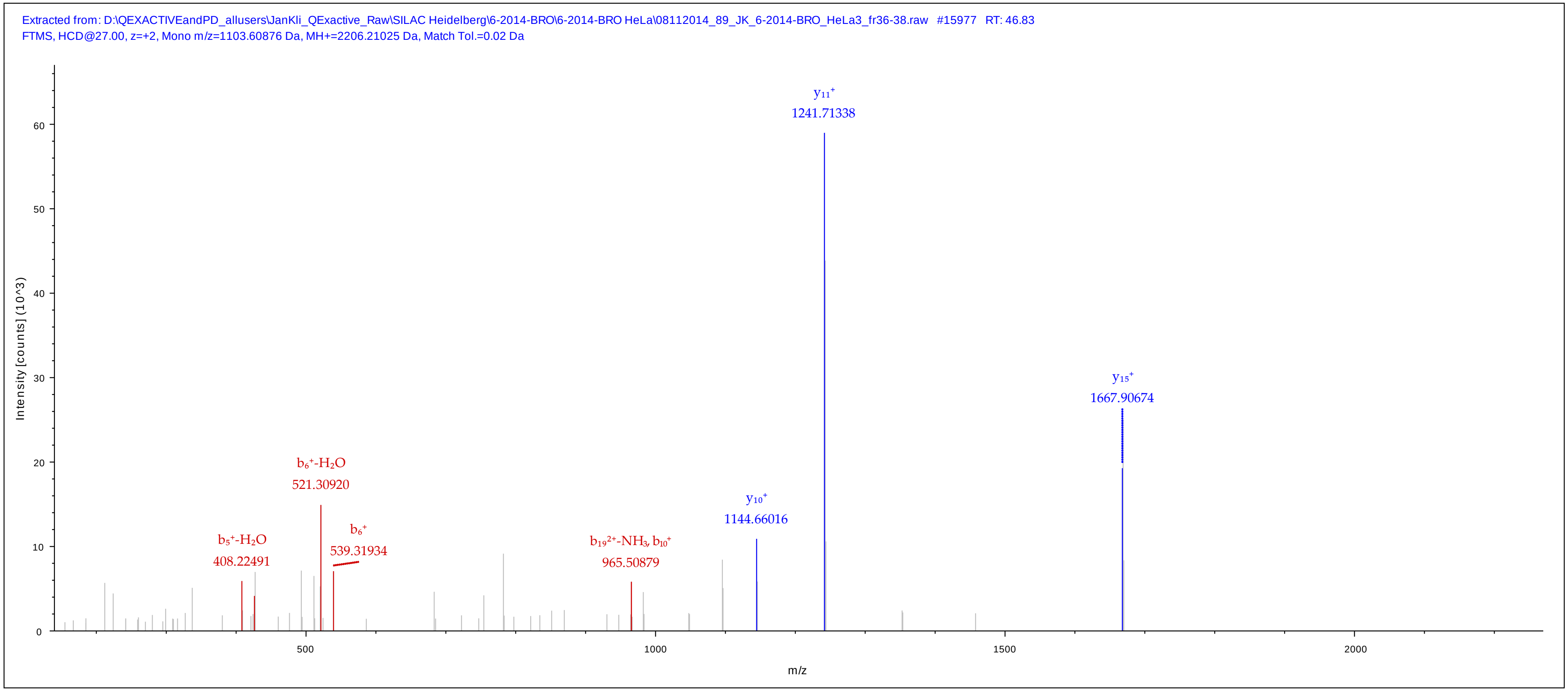

ALGPSLPNSQPPPEVYVSLIK

Not used for quantification
(missing heavy channel)

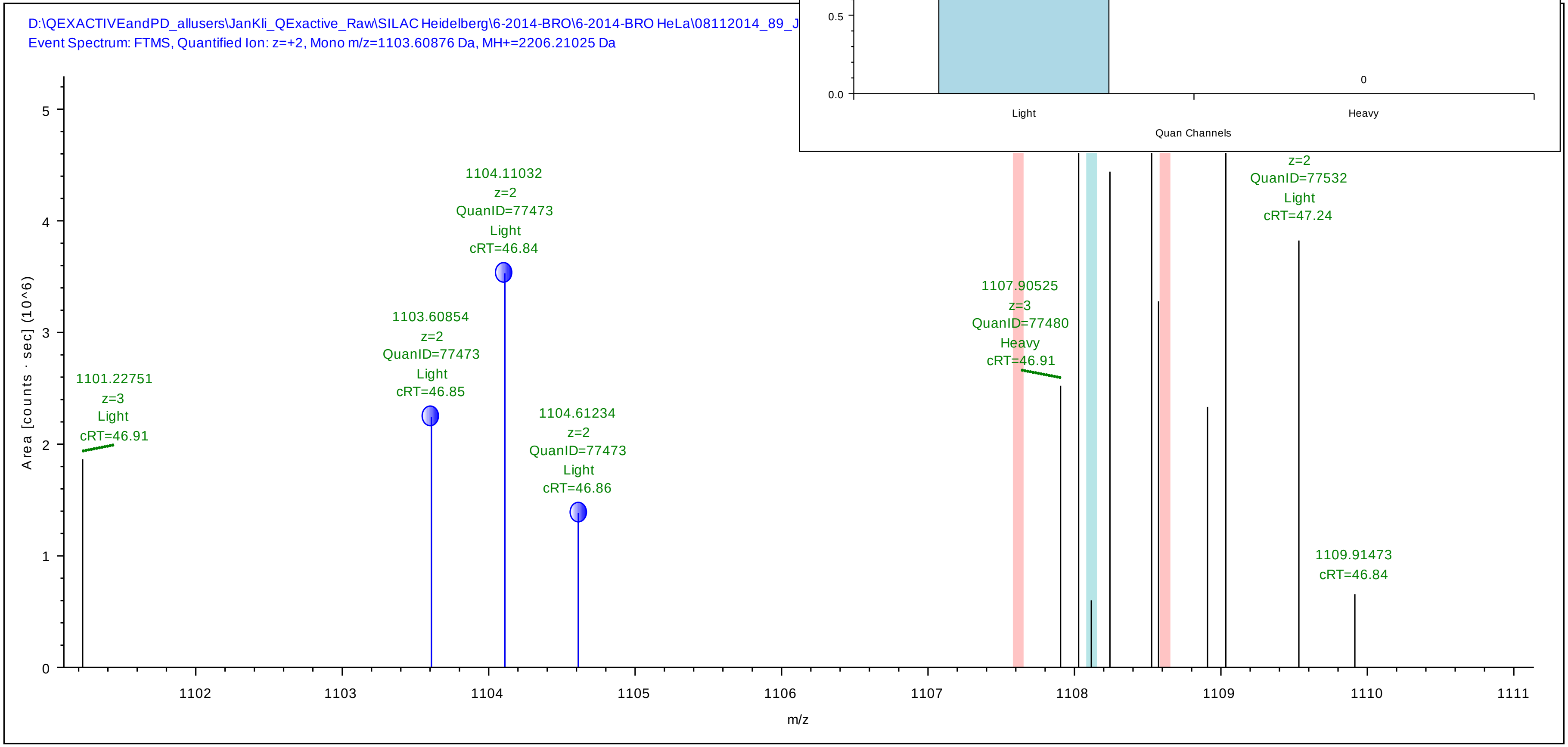

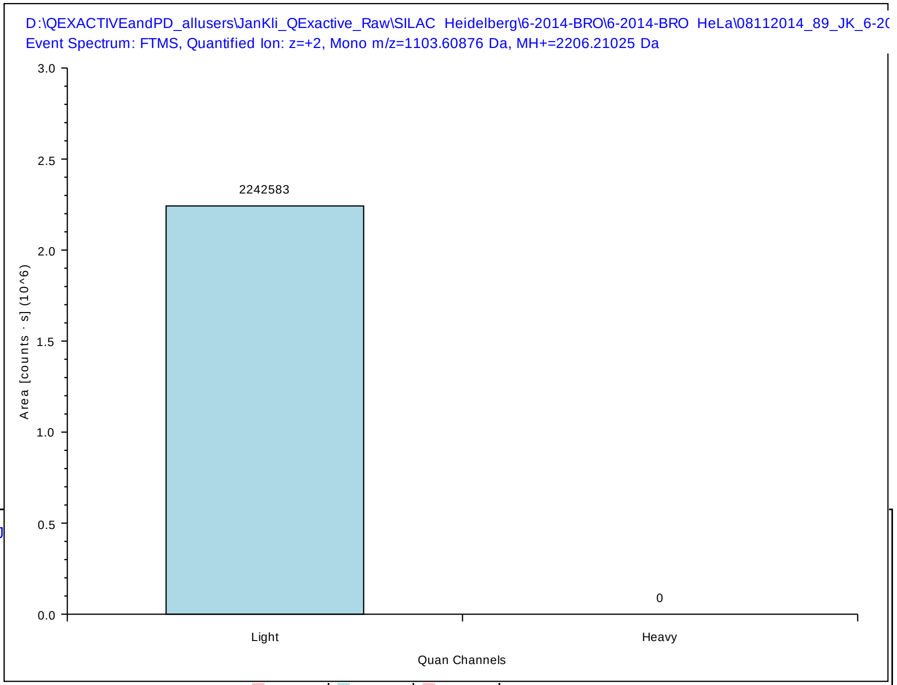

Galectin-7 OS=Homo sapiens GN=LGALS7 PE=1 SV=2 - [LEG7_HUMAN]

Identified peptides:

LDTSEVVFNSK
FHVNLLCGEEQGSDAALHFNPR
GQPFEVLIIASDDGFK
AVVGDAQYHHFR
GPGVPFQR
SSLPEGIRPGTVLR
LVEVGGDVQLDSVR
GLVPPNASR

Coverage | ProteinCard

Galectin-7 OS=Homo sapiens GN=LGALS7 PE=1 SV=2 - [LEG7_HUMAN]

☐ Annotate PTMs reported in Uniprot
☐ Show only PTMs
☐ Include PSMs that are filtered Out

Coverage: 77.94%

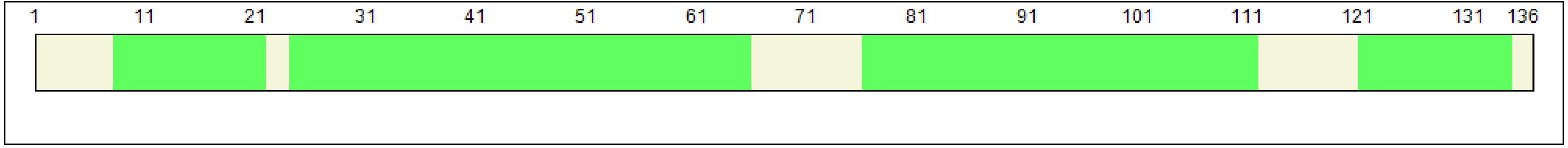

Found Modifications:

**C**	Carbamidomethyl (C)
**L**	Label:13C(6)15N(4) (R)
**M**	Label:13C(6)15N(2) (K)

Sequence | Modification List

```
     1         11        21        31        41        51        61        71        81        91        101
  1            L     L          L      C                  L         M                   L              M
     MSNVPHKSSL PEGIRPGTVL RIRGLVPPNA SRFHVNLLCG EEQGSDAALH FNPRLDTSEV VFNSKEQGSW GREERGPGVP FQRGQPFEVL IIASDDGFKA VVGDAQYHHF

111  L                      L
     RHRLPLARVR LVEVGGDVQL DSVRIF
```

Sequence: LDTSEVVFNSK, K11-Label:13C(6)15N(2) (8.01420 Da)
Charge: +2, Monoisotopic m/z: 623.82092 Da (-3 mmu/-4.81 ppm), MH+: 1246.63457 Da,
RT: 29.15 min,
Identified with: Mascot (v1.30); IonScore:74, Exp Value:9.4E-006, Ions matched by search engine: 8/100
Fragment match tolerance used for search: 0.02 Da

#1	b^+	b^{2+}	Seq.	y^+	y^{2+}	#2
1	114.09135	57.54931	L			11
2	229.11830	115.06279	D	1133.55649	567.28188	10
3	330.16598	165.58663	T	1018.52954	509.76841	9
4	417.19801	209.10264	S	917.48186	459.24457	8
5	546.24061	273.62394	E	830.44983	415.72855	7
6	645.30903	323.15815	V	701.40723	351.20725	6
7	744.37745	372.69236	V	602.33881	301.67304	5
8	891.44587	446.22657	F	503.27039	252.13883	4
9	1005.48880	503.24804	N	356.20197	178.60462	3
10	1092.52083	546.76405	S	242.15904	121.58316	2
11			K-Label:13C(6)15N(2)	155.12701	78.06714	1

LDTSEVVFNSK

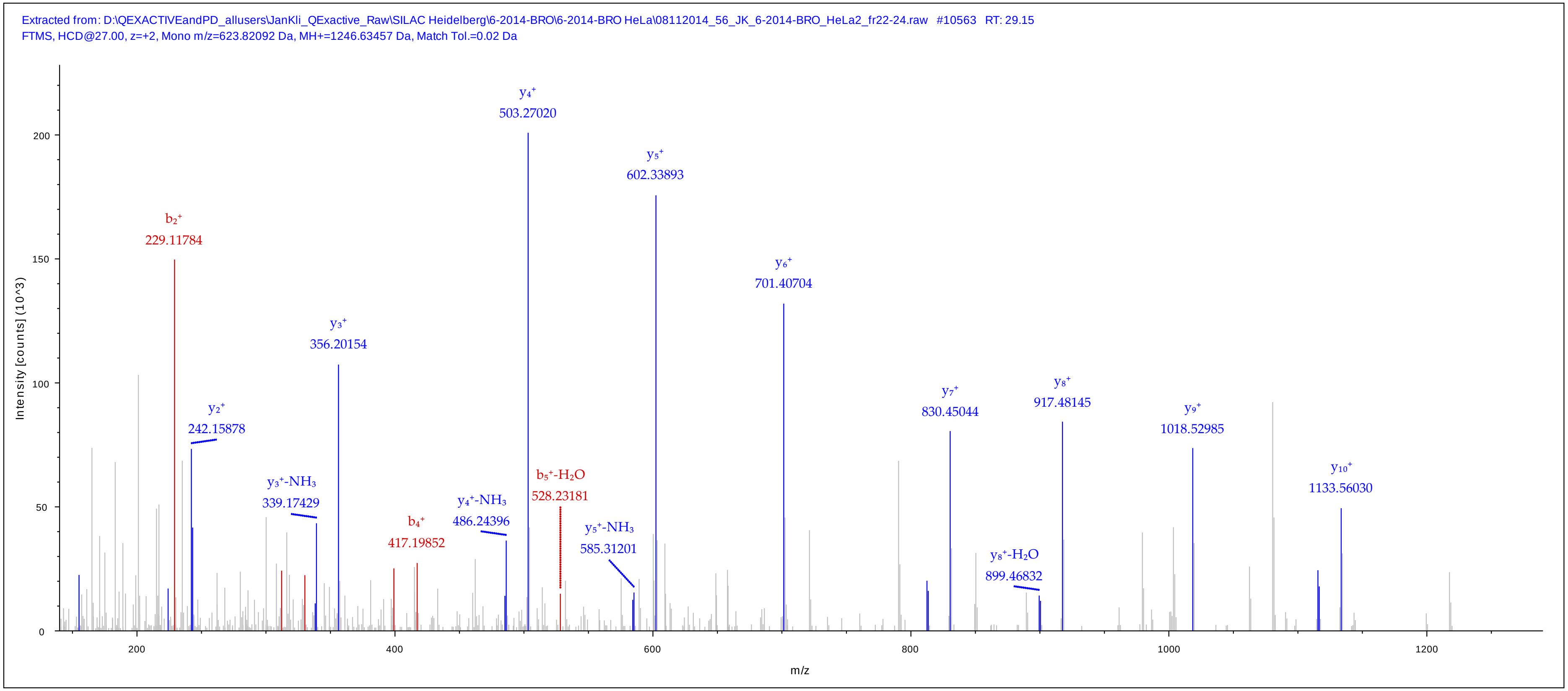

LDTSEVVFNSK

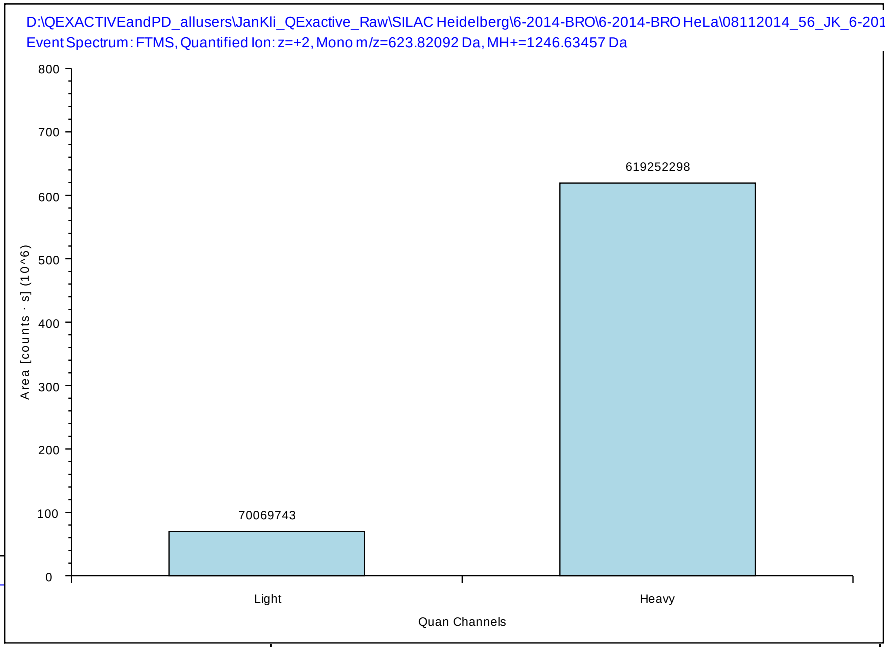

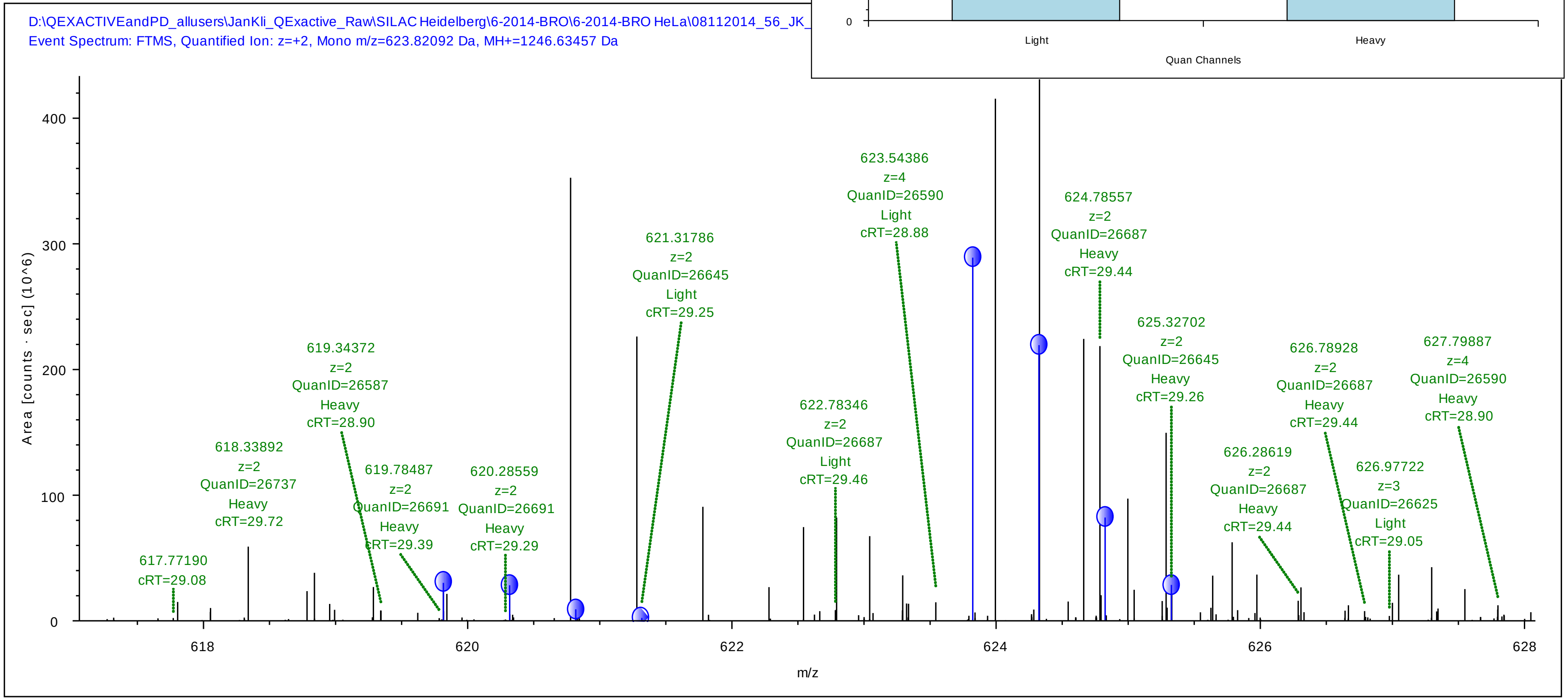

Sequence: GQPFEVLIIASDDGFK, K16-Label:13C(6)15N(2) (8.01420 Da)
Charge: +3, Monoisotopic m/z: 581.97192 Da (-1.07 mmu/-1.84 ppm), MH+: 1743.90122 Da, RT: 51.50 min,
Identified with: Mascot (v1.30); IonScore:56, Exp Value:7.9E-004, Ions matched by search engine: 10/164
Fragment match tolerance used for search: 0.02 Da

#1	b⁺	b²⁺	b³⁺	Seq.	y⁺	y²⁺	y³⁺	#2
1	58.02875	29.51801	20.01443	G				16
2	186.08733	93.54730	62.70063	Q	1686.88295	843.94511	562.96583	15
3	283.14010	142.07369	95.05155	P	1558.82437	779.91582	520.27964	14
4	430.20852	215.60790	144.07436	F	1461.77160	731.38944	487.92872	13
5	559.25112	280.12920	187.08856	E	1314.70318	657.85523	438.90591	12
6	658.31954	329.66341	220.11136	V	1185.66058	593.33393	395.89171	11
7	771.40361	386.20544	257.80605	L	1086.59216	543.79972	362.86890	10
8	884.48768	442.74748	295.50074	I	973.50809	487.25768	325.17421	9
9	997.57175	499.28951	333.19543	I	860.42402	430.71565	287.47952	8
10	1068.60887	534.80807	356.87447	A	747.33995	374.17361	249.78483	7
11	1155.64090	578.32409	385.88515	S	676.30283	338.65505	226.10579	6
12	1270.66785	635.83756	424.22747	D	589.27080	295.13904	197.09512	5
13	1385.69480	693.35104	462.56978	D	474.24385	237.62556	158.75280	4
14	1442.71627	721.86177	481.57694	G	359.21690	180.11209	120.41048	3
15	1589.78469	795.39598	530.59975	F	302.19543	151.60135	101.40333	2
16				K-Label:13C(6)15N(2)	155.12701	78.06714	52.38052	1

GQPFEVLIIASDDGFK

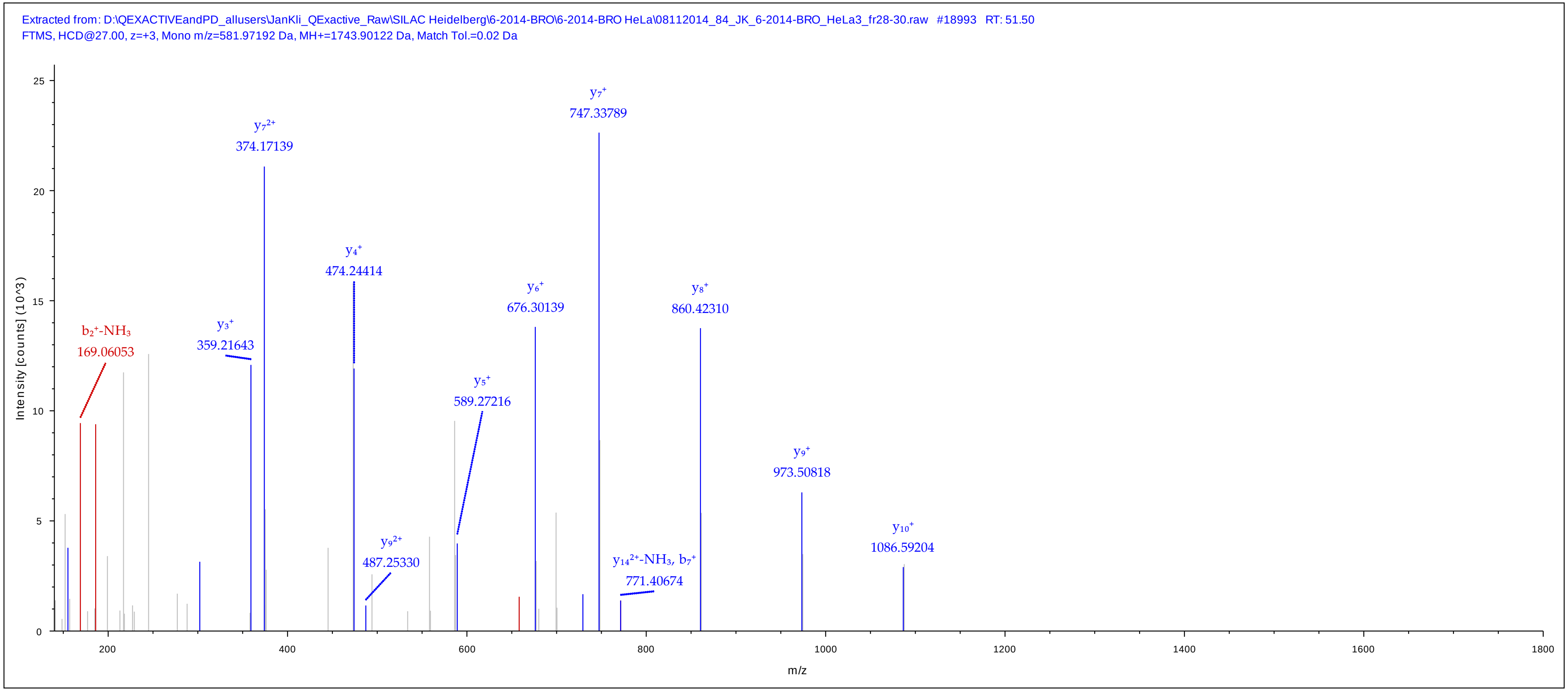

GQPFEVLIIASDDGFK

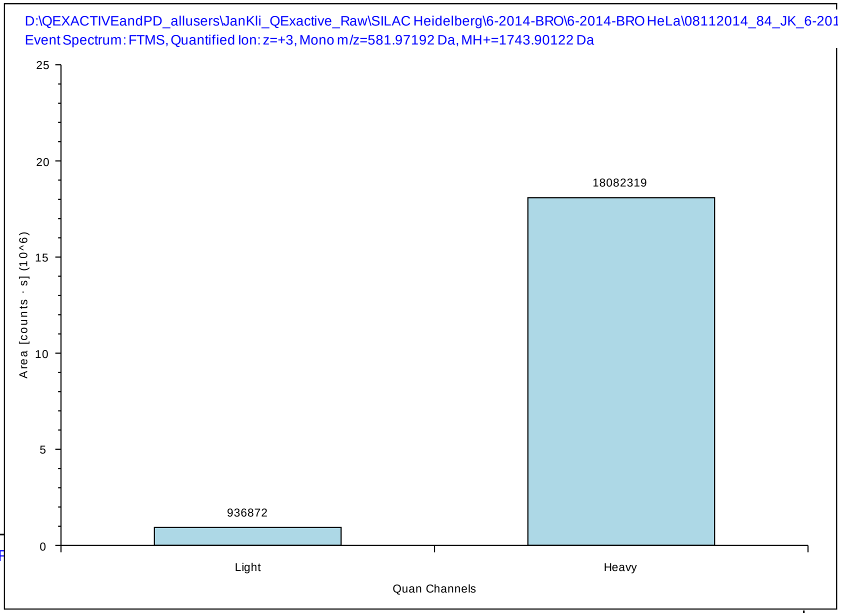

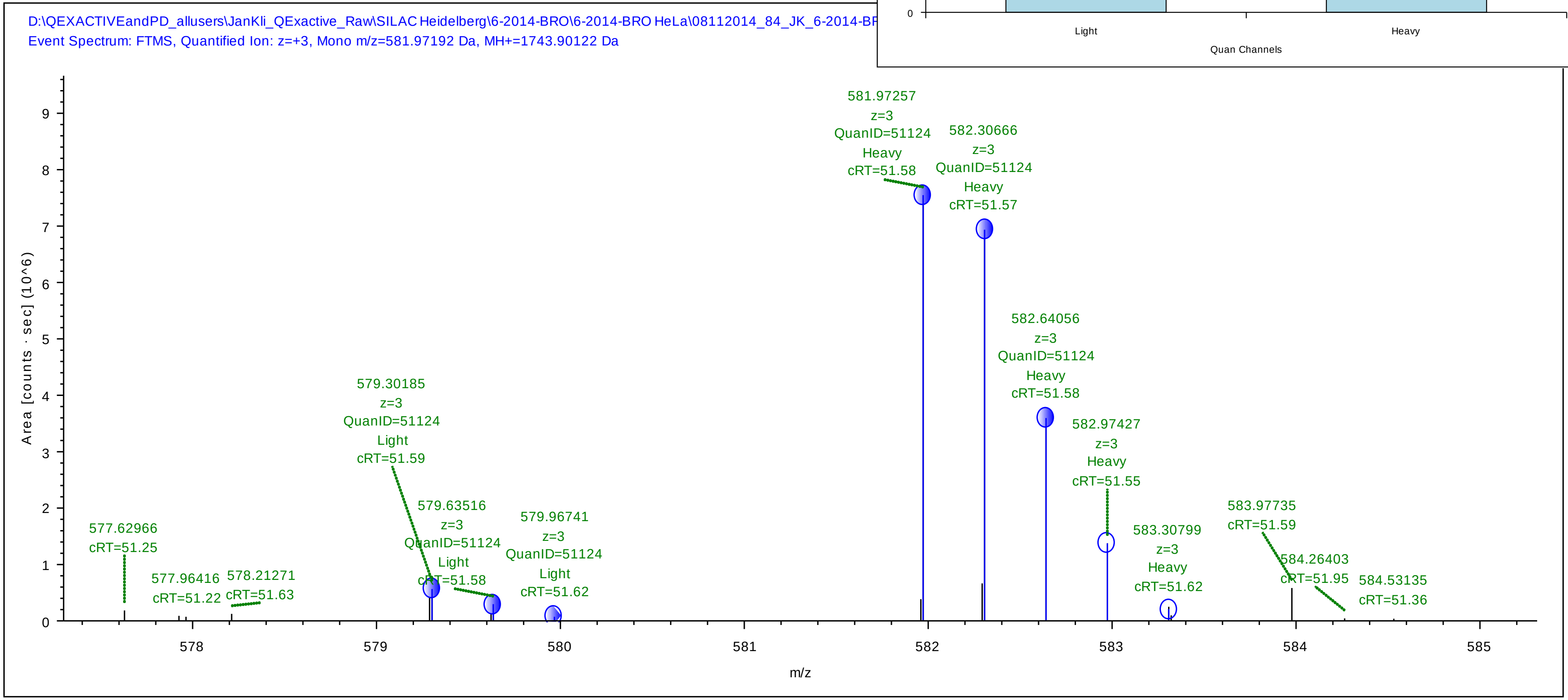

SiHa VS SiHa Gal-7+ Cell lines

B-cell lymphoma 3 protein OS=Homo sapiens GN=BCL3 PE=1 SV=2 - [BCL3_HUMAN]

Identified peptides:

NCHNDTPLMVAR
HGQTAAHLACEHR
LVNLFQQGGR
AAGLPGAALPLR
LLVTAGASPMALDR
QTPLHLAVITTLPSVVR
PEALYYPGALLPLYPTR
CPAGAMDEGPVDLR
VIDILR
GLLPLVR

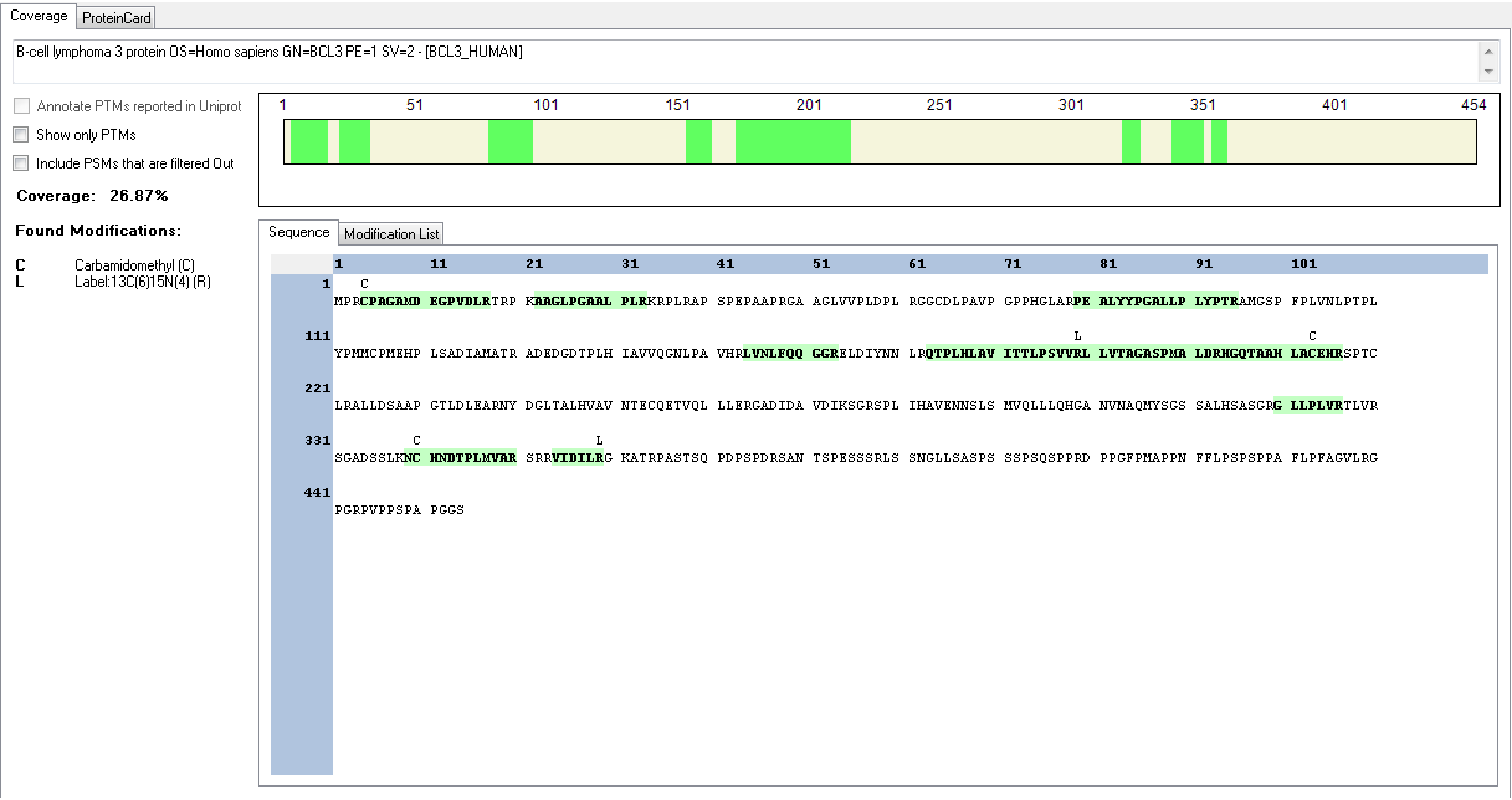
Coverage
ProteinCard
B-cell lymphoma 3 protein OS=Homo sapiens GN=BCL3 PE=1 SV=2 - [BCL3_HUMAN]
Annotate PTMs reported in Uniprot
Show only PTMs
Include PSMs that are filtered Out
Coverage: 26.87%
Found Modifications:
C Carbamidomethyl (C)
L Label:13C(6)15N(4) (R)
1 51 101 151 201 251 301 351 401 454
Sequence
Modification List
1 11 21 31 41 51 61 71 81 91 101
1 MPRCPAGAMD EGPVDLRTRP KAAGLPGAAL PLRKRPLRAP SPEPAAPRGA AGLVVPLDPL RGGCDLPAVP GPPHGLARPE ALYYPGALLP LYPTRAMGSP FPLVNLPTPL
111 YPMMCPMEHP LSADIAMATR ADEDGDTPLH IAVVQGNLPA VHRLVNLFQQ GGRELDIYNN LRQTPLHLAV ITTLPSVVRL LVTAGASPMA LDRHGQTAAH LACEHRSPTC
221 LRALLDSAAP GTLDLEARNY DGLTALHVAV NTECQETVQL LLERGADIDA VDIKSGRSPL IHAVENNSLS MVQLLLQHGA NVNAQMYSGS SALHSASGRG LLPLVRTLVR
331 SGADSSLKNC HNDTPLMVAR SRRVIDILRG KATRPASTSQ PDPSPDRSAN TSPESSSRLS SNGLLSASPS SSPSQSPPRD PPGFPMAPPN FFLPSPSPPA FLPFAGVLRG
441 PGRPVPPSPA PGGS

Sequence: QTPLHLAVITTLPSVVR, R17-Label:13C(6)15N(4) (10.00827 Da)
Charge: +3, Monoisotopic m/z: 619.03882 Da (-0.7 mmu/-1.13 ppm), MH+: 1855.10190 Da, RT: 42.42 min,
Identified with: Mascot (v1.30); IonScore:51, Exp Value:4.4E-004, Ions matched by search engine: 7/184
Fragment match tolerance used for search: 0.02 Da

#1	b^{+}	b^{2+}	b^{3+}	Seq.	y^{+}	y^{2+}	y^{3+}	#2
1	129.06586	65.03657	43.69347	Q				17
2	230.11354	115.56041	77.37603	T	1727.04541	864.02634	576.35332	16
3	327.16631	164.08679	109.72695	P	1625.99773	813.50250	542.67076	15
4	440.25038	220.62883	147.42164	L	1528.94496	764.97612	510.31984	14
5	577.30929	289.15828	193.10795	H	1415.86089	708.43408	472.62515	13
6	690.39336	345.70032	230.80264	L	1278.80198	639.90463	426.93884	12
7	761.43048	381.21888	254.48168	A	1165.71791	583.36259	389.24415	11
8	860.49890	430.75309	287.50448	V	1094.68079	547.84403	365.56511	10
9	973.58297	487.29512	325.19917	I	995.61237	498.30982	332.54231	9
10	1074.63065	537.81896	358.88173	T	882.52830	441.76779	294.84762	8
11	1175.67833	588.34280	392.56429	T	781.48062	391.24395	261.16506	7
12	1288.76240	644.88484	430.25898	L	680.43294	340.72011	227.48250	6
13	1385.81517	693.41122	462.60991	P	567.34887	284.17807	189.78781	5
14	1472.84720	736.92724	491.62058	S	470.29610	235.65169	157.43688	4
15	1571.91562	786.46145	524.64339	V	383.26407	192.13567	128.42621	3
16	1670.98404	835.99566	557.66620	V	284.19565	142.60146	95.40340	2
17				R-Label:13C(6)15N(4)	185.12723	93.06725	62.38059	1

QTPLHLAVITTLPSVVR

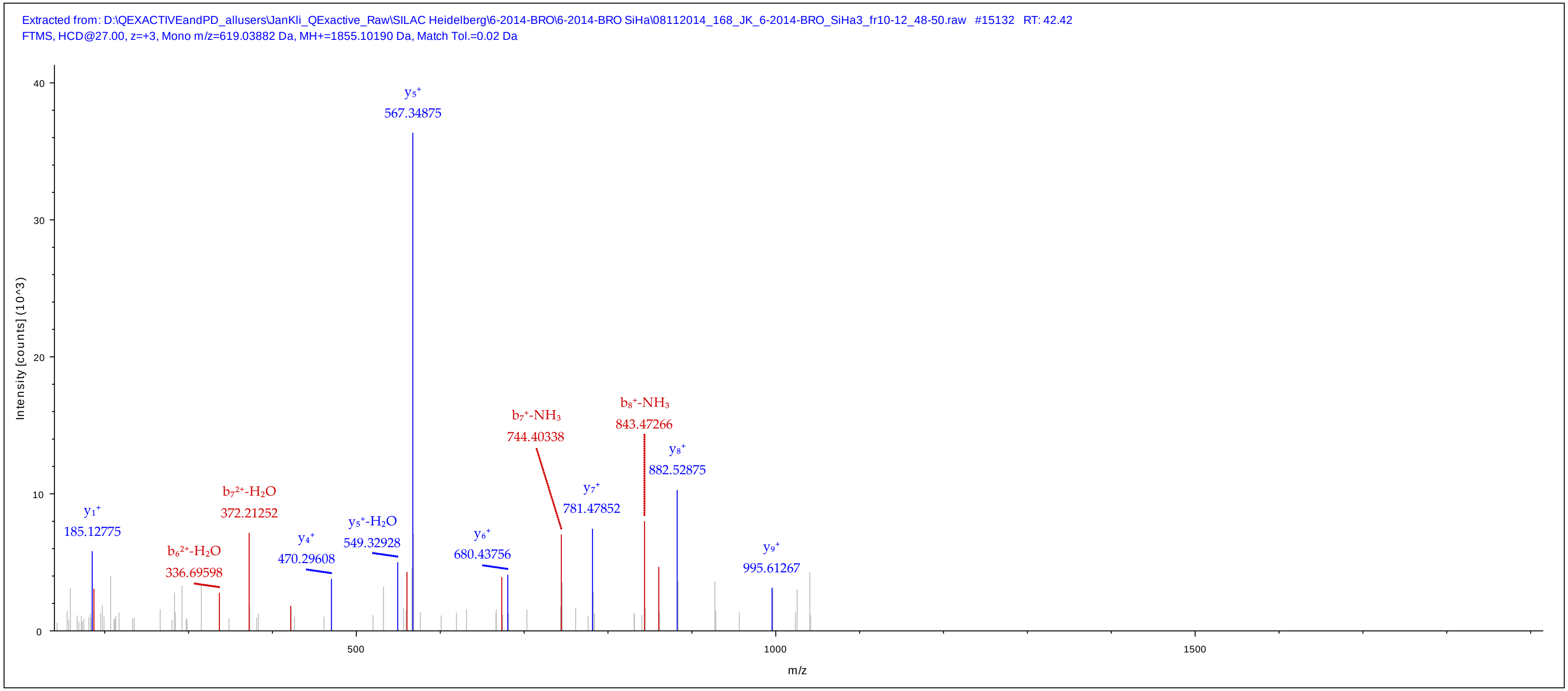

QTPLHLAVITTLPSVVR

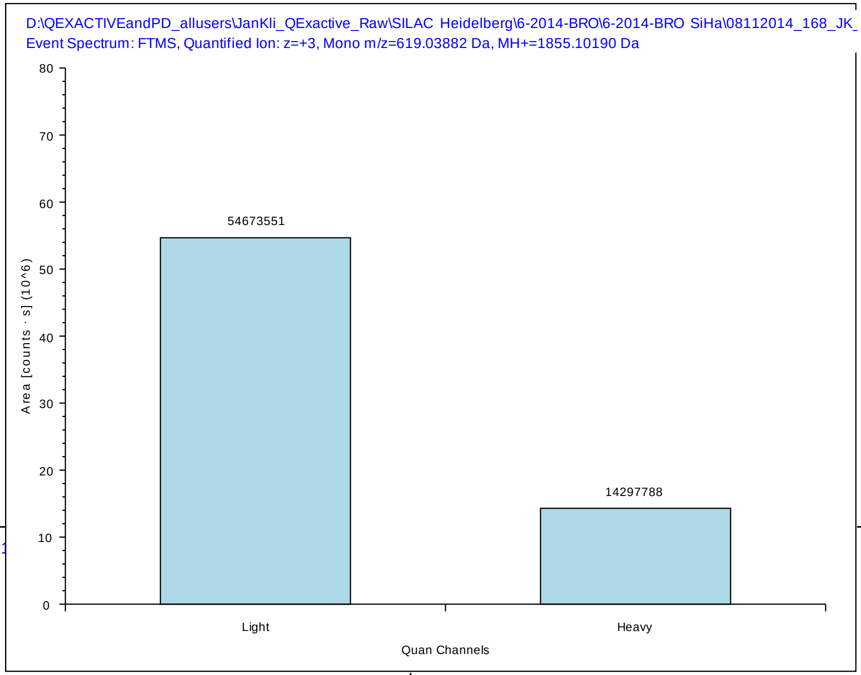

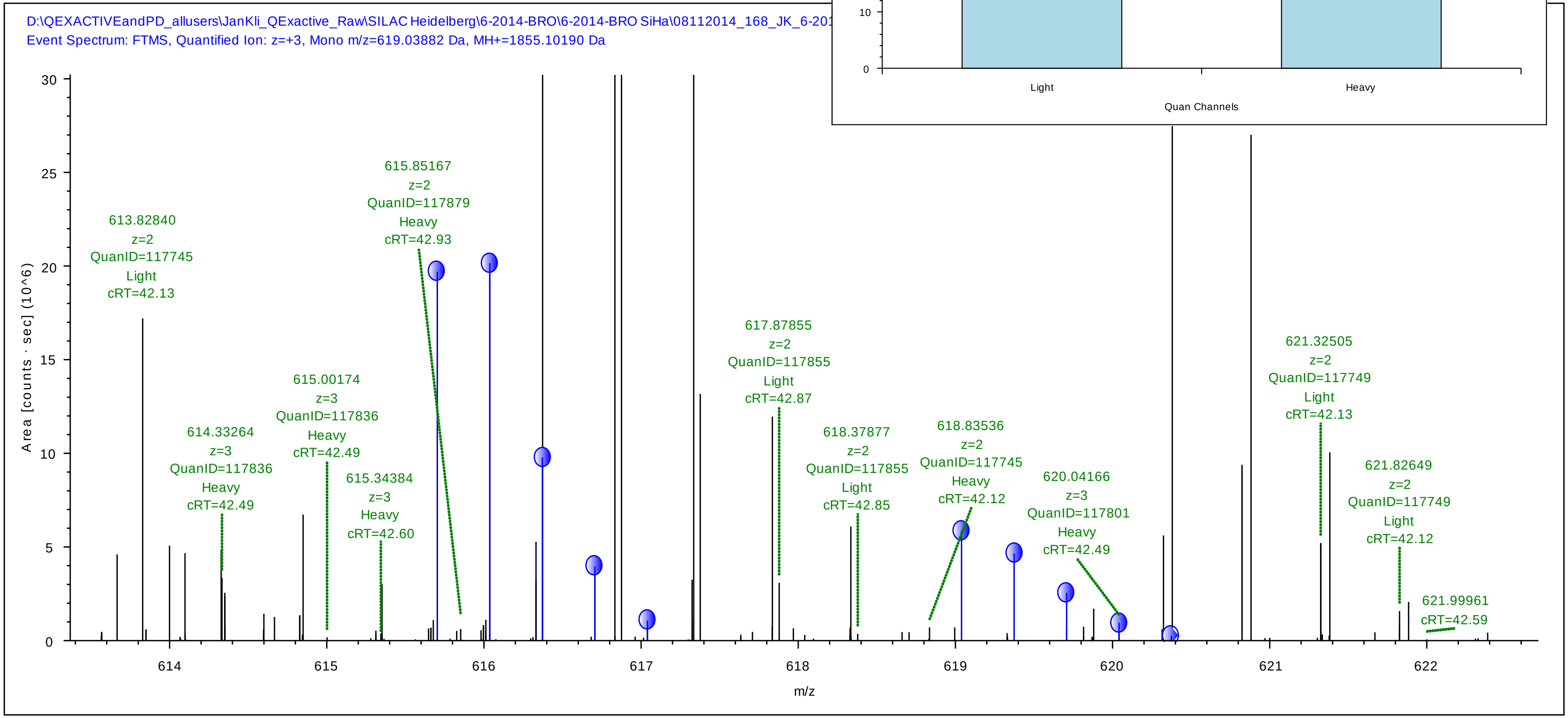

Sequence: GLLPLVR, Charge: +2, Monoisotopic m/z: 384.26016 Da (-0.39 mmu/-1.02 ppm), MH+: 767.51305 Da, RT: 34.44 min,
Identified with: Mascot (v1.30); IonScore:40, Exp Value:6.9E-003, Ions matched by search engine: 5/50
Fragment match tolerance used for search: 0.02 Da

#1	b^+	b^{2+}	Seq.	y^+	y^{2+}	#2
1	58.02875	29.51801	G			7
2	171.11282	86.06005	L	710.49236	355.74982	6
3	284.19689	142.60208	L	597.40829	299.20778	5
4	381.24966	191.12847	P	484.32422	242.66575	4
5	494.33373	247.67050	L	387.27145	194.13936	3
6	593.40215	297.20471	V	274.18738	137.59733	2
7			R	175.11896	88.06312	1

GLLPLVR

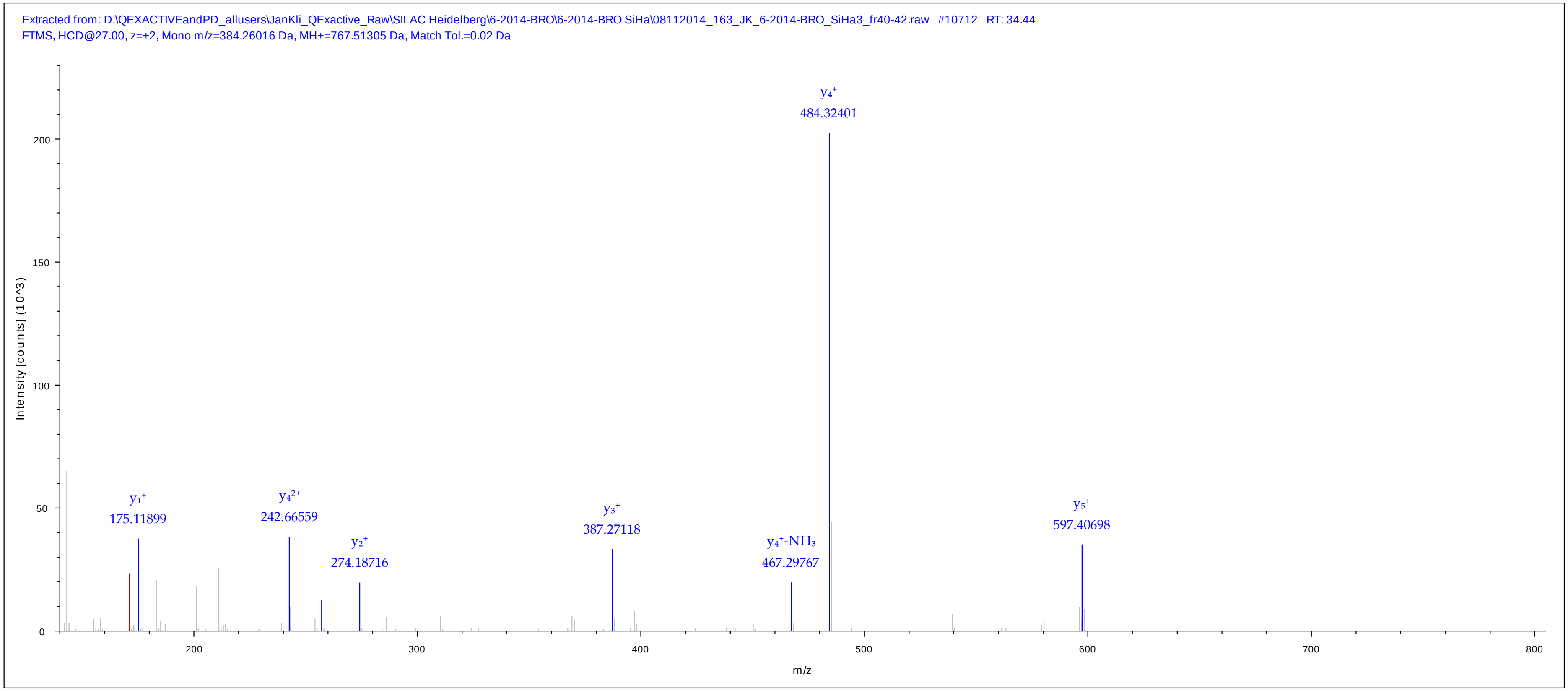

Extracted from: D:\QEXACTIVEandPD_allusers\JanKli_QExactive_Raw\SILAC Heidelberg\6-2014-BRO\6-2014-BRO SiHa\08112014_163_JK_6-2014-BRO_SiHa3_fr40-42.raw #10712 RT: 34.44
FTMS, HCD@27.00, z=+2, Mono m/z=384.26016 Da, MH+=767.51305 Da, Match Tol.=0.02 Da
Intensity [counts] (10^3)
m/z
y4+ 484.32401
y1+ 175.11899
y42+ 242.66559
y2+ 274.18716
y3+ 387.27118
y4+-NH3 467.29767
y5+ 597.40698

GLLPLVR

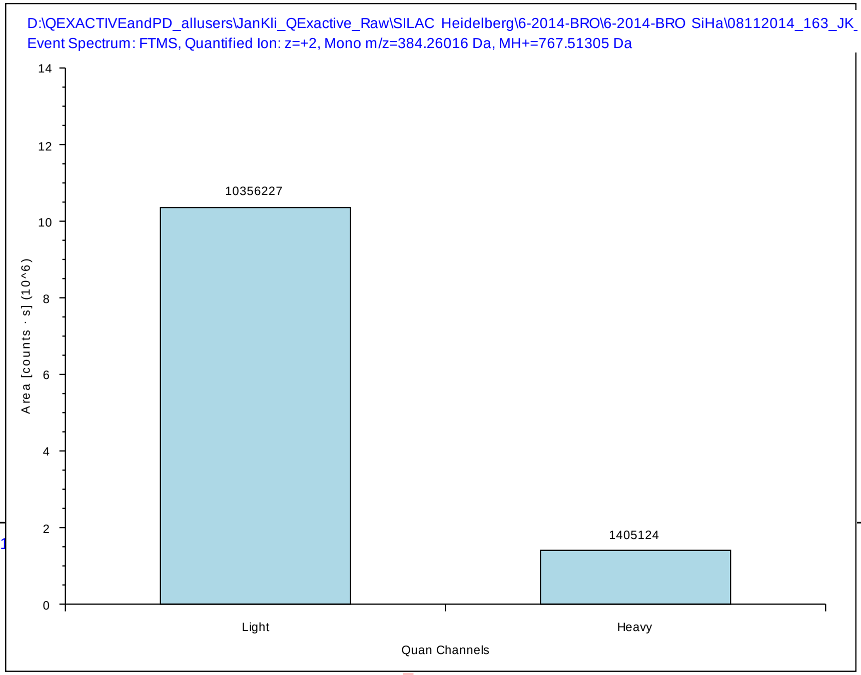

D:\QEXACTIVEandPD_allusers\JanKli_QExactive_Raw\SILAC Heidelberg\6-2014-BRO\6-2014-BRO SiHa\08112014_163_JK_
Event Spectrum: FTMS, Quantified Ion: z=+2, Mono m/z=384.26016 Da, MH+=767.51305 Da
14
12
10
8
6
4
2
0
Area [counts · s] (10^6)
10356227
1405124
Light
Heavy
Quan Channels

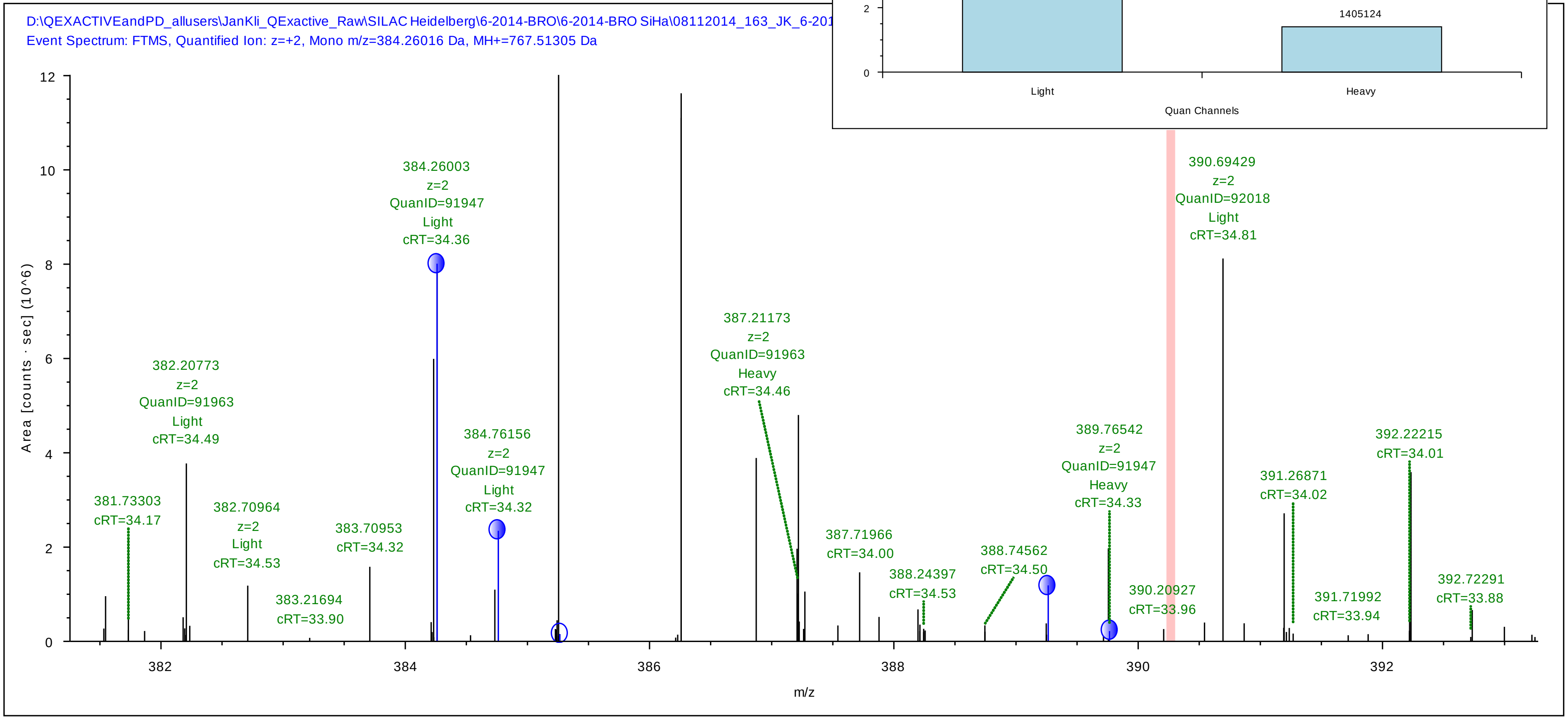

D:\QEXACTIVEandPD_allusers\JanKli_QExactive_Raw\SILAC Heidelberg\6-2014-BRO\6-2014-BRO SiHa\08112014_163_JK_6-201
Event Spectrum: FTMS, Quantified Ion: z=+2, Mono m/z=384.26016 Da, MH+=767.51305 Da
12
10
8
6
4
2
0
Area [counts · sec] (10^6)
384.26003 z=2 QuanID=91947 Light cRT=34.36
390.69429 z=2 QuanID=92018 Light cRT=34.81
387.21173 z=2 QuanID=91963 Heavy cRT=34.46
382.20773 z=2 QuanID=91963 Light cRT=34.49
384.76156 z=2 QuanID=91947 Light cRT=34.32
389.76542 z=2 QuanID=91947 Heavy cRT=34.33
392.22215 cRT=34.01
391.26871 cRT=34.02
381.73303 cRT=34.17
382.70964 z=2 Light cRT=34.53
383.70953 cRT=34.32
387.71966 cRT=34.00
388.24397 cRT=34.53
388.74562 cRT=34.50
392.72291 cRT=33.88
390.20927 cRT=33.96
391.71992 cRT=33.94
383.21694 cRT=33.90
382
384
386
388
390
392
m/z

Cyclin-dependent kinase inhibitor 2A, isoform 4 OS=Homo sapiens GN=CDKN2A PE=1 SV=2 - [CD2A2_HUMAN]

Identified peptides:

VFVVHIPR
RPGHDDGQRPSGGAAAAPR
CPGGLPGHAGGAAPGR
LGQQPLPR
FLVTLR

Coverage | ProteinCard

Cyclin-dependent kinase inhibitor 2A, isoform 4 OS=Homo sapiens GN=CDKN2A PE=1 SV=2 - [CD2A2_HUMAN]

☐ Annotate PTMs reported in Uniprot
☐ Show only PTMs
☐ Include PSMs that are filtered Out

Coverage: 43.18%

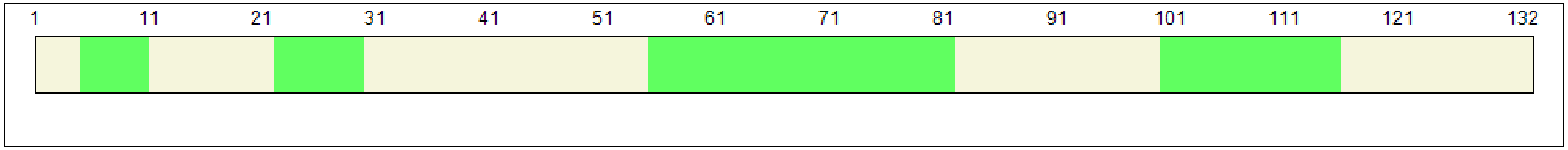

Found Modifications:

C Carbamidomethyl (C)
L Label:13C(6)15N(4) (R)

Sequence | Modification List

```
     1         11         21         31         41         51         61         71         81         91         101
  1          L                                                          LL         L          L                    C
     MVRRFLVTLR IRRACGPPRV RVFVVHIPRL TGEWAAPGAP AAVALVLMLL RSQRLGQQPL PRRPGHDDGQ RPSGGAAAAP RRGAQLRRPR HSHPTRARRC PGGLPGHAGG

111
     AAPGRGAAGR ARCLGPSARG PG
```

Sequence: CPGGLPGHAGGAAPGR, C1-Carbamidomethyl (57.02146 Da)
Charge: +3, Monoisotopic m/z: 477.90076 Da (-1.12 mmu/-2.35 ppm), MH+: 1431.68772 Da, RT: 16.19 min,
Identified with: Mascot (v1.30); IonScore:22, Exp Value:1.0E+000, Ions matched by search engine: 9/122
Fragment match tolerance used for search: 0.02 Da

#1	b^+	b^{2+}	b^{3+}	Seq.	y^+	y^{2+}	y^{3+}	#2
1	161.03793	81.02260	54.35083	C-Carbamidomethyl				16
2	258.09070	129.54899	86.70175	P	1271.66043	636.33385	424.55833	15
3	315.11217	158.05972	105.70891	G	1174.60766	587.80747	392.20740	14
4	372.13364	186.57046	124.71606	G	1117.58619	559.29673	373.20025	13
5	485.21771	243.11249	162.41075	L	1060.56472	530.78600	354.19309	12
6	582.27048	291.63888	194.76168	P	947.48065	474.24396	316.49840	11
7	639.29195	320.14961	213.76883	G	850.42788	425.71758	284.14748	10
8	776.35086	388.67907	259.45514	H	793.40641	397.20684	265.14032	9
9	847.38798	424.19763	283.13418	A	656.34750	328.67739	219.45402	8
10	904.40945	452.70836	302.14133	G	585.31038	293.15883	195.77498	7
11	961.43092	481.21910	321.14849	G	528.28891	264.64809	176.76782	6
12	1032.46804	516.73766	344.82753	A	471.26744	236.13736	157.76066	5
13	1103.50516	552.25622	368.50657	A	400.23032	200.61880	134.08162	4
14	1200.55793	600.78260	400.85749	P	329.19320	165.10024	110.40258	3
15	1257.57940	629.29334	419.86465	G	232.14043	116.57385	78.05166	2
16				R	175.11896	88.06312	59.04450	1

CPGGLPGHAGGAAPGR

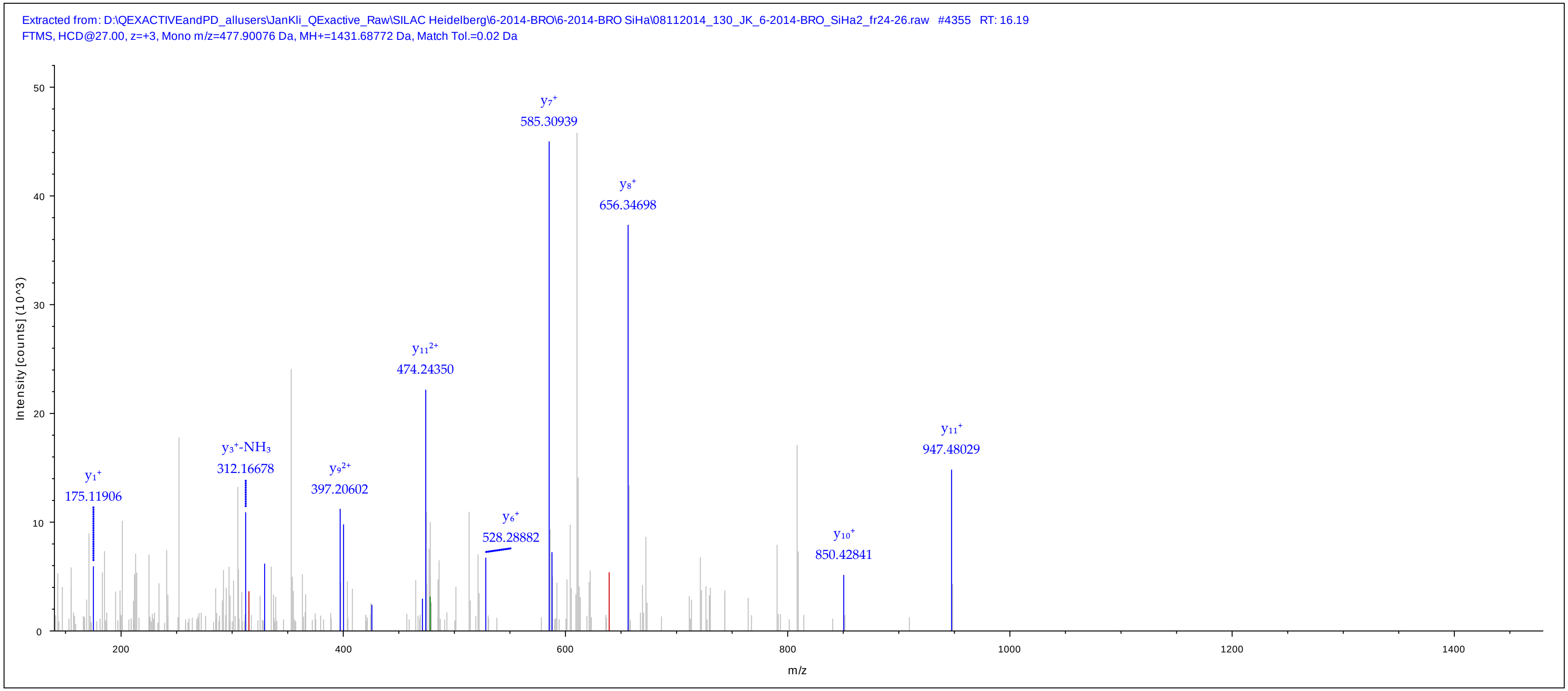

CPGGLPGHAGGAAPGR

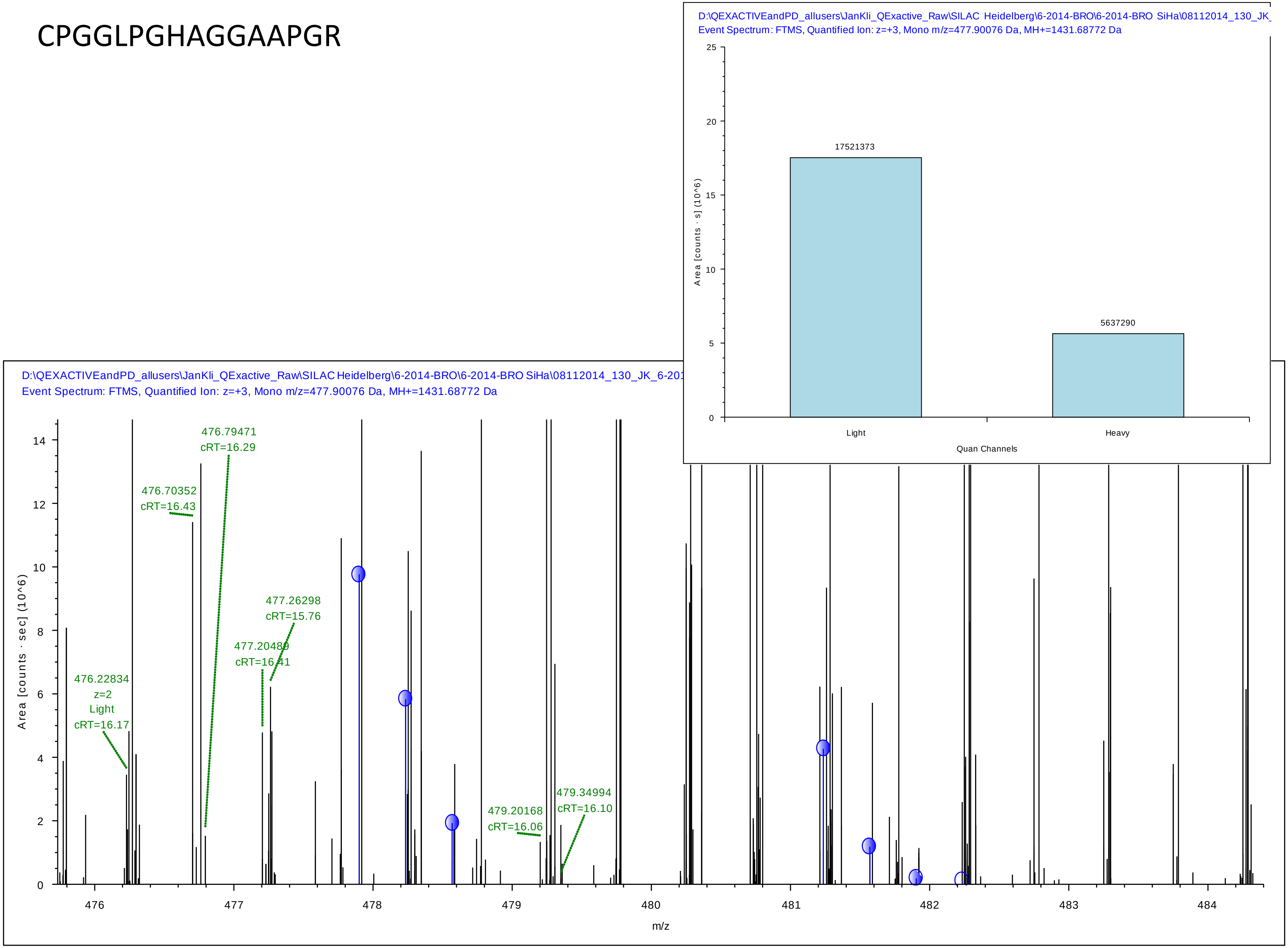

Sequence: VFVVHIPR, Charge: +2, Monoisotopic m/z: 483.79874 Da (+0.9 mmu/+1.87 ppm), MH+: 966.59020 Da, RT: 27.26 min,
Identified with: Mascot (v1.30); IonScore:54, Exp Value:6.2E-004, Ions matched by search engine: 7/58
Fragment match tolerance used for search: 0.02 Da

#1	b^+	b^{2+}	Seq.	y^+	y^{2+}	#2
1	100.07570	50.54149	V			8
2	247.14412	124.07570	F	867.51997	434.26362	7
3	346.21254	173.60991	V	720.45155	360.72941	6
4	445.28096	223.14412	V	621.38313	311.19520	5
5	582.33987	291.67357	H	522.31471	261.66099	4
6	695.42394	348.21561	I	385.25580	193.13154	3
7	792.47671	396.74199	P	272.17173	136.58950	2
8			R	175.11896	88.06312	1

VFVVHIPR

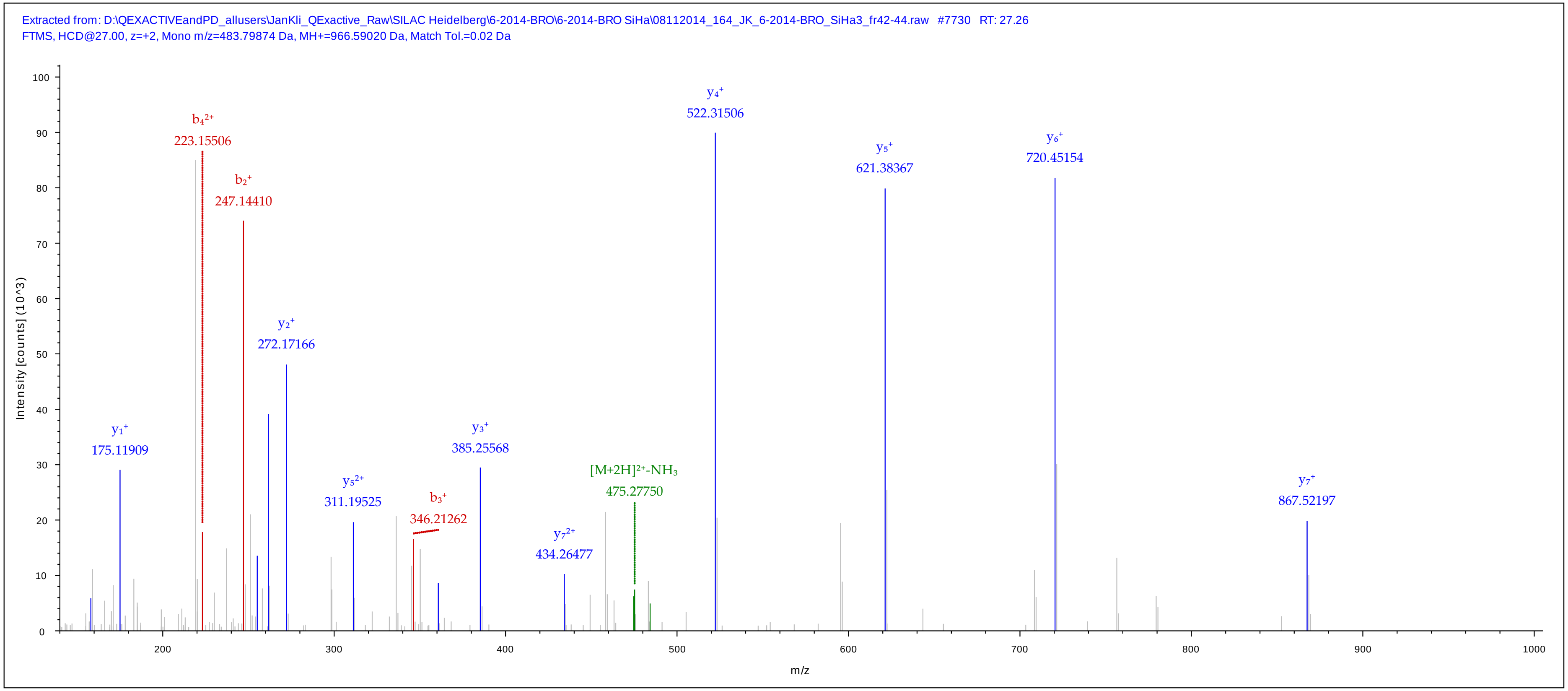

Extracted from: D:\QEXACTIVEandPD_allusers\JanKli_QExactive_Raw\SILAC Heidelberg\6-2014-BRO\6-2014-BRO SiHa\08112014_164_JK_6-2014-BRO_SiHa3_fr42-44.raw #7730 RT: 27.26
FTMS, HCD@27.00, z=+2, Mono m/z=483.79874 Da, MH+=966.59020 Da, Match Tol.=0.02 Da
Intensity [counts] (10^3)
m/z
b4 2+ 223.15506
b2+ 247.14410
y1+ 175.11909
y2+ 272.17166
y5 2+ 311.19525
b3+ 346.21262
y3+ 385.25568
y7 2+ 434.26477
[M+2H]2+-NH3 475.27750
y4+ 522.31506
y5+ 621.38367
y6+ 720.45154
y7+ 867.52197

VFVVHIPR

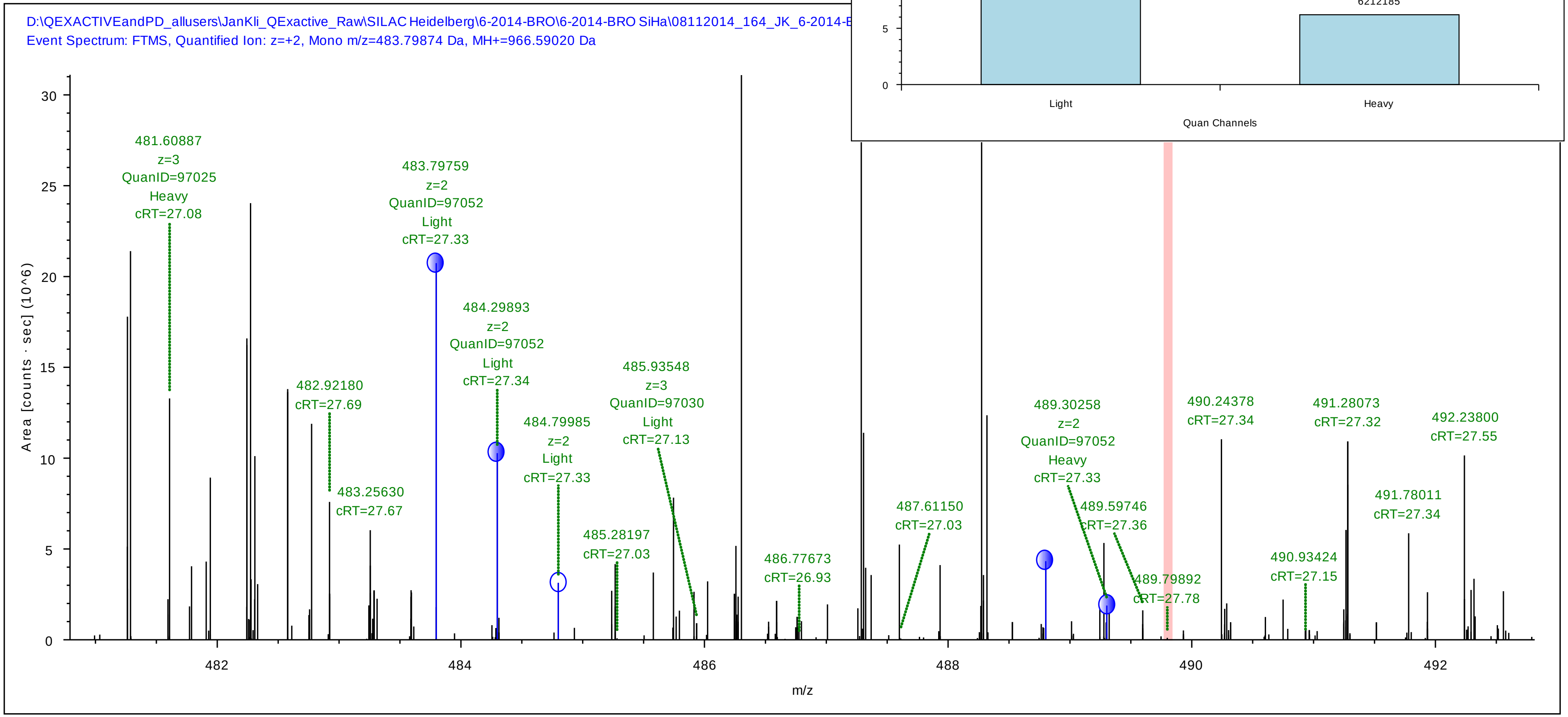
D:\QEXACTIVEandPD_allusers\JanKli_QExactive_Raw\SILAC Heidelberg\6-2014-BRO\6-2014-BRO SiHa\08112014_164_JK_6-2014-B
Event Spectrum: FTMS, Quantified Ion: z=+2, Mono m/z=483.79874 Da, MH+=966.59020 Da
Area [counts · sec] (10^6)
m/z
481.60887 z=3 QuanID=97025 Heavy cRT=27.08
483.79759 z=2 QuanID=97052 Light cRT=27.33
484.29893 z=2 QuanID=97052 Light cRT=27.34
484.79985 z=2 Light cRT=27.33
482.92180 cRT=27.69
483.25630 cRT=27.67
485.28197 cRT=27.03
485.93548 z=3 QuanID=97030 Light cRT=27.13
486.77673 cRT=26.93
487.61150 cRT=27.03
489.30258 z=2 QuanID=97052 Heavy cRT=27.33
489.59746 cRT=27.36
489.79892 cRT=27.78
490.24378 cRT=27.34
490.93424 cRT=27.15
491.28073 cRT=27.32
491.78011 cRT=27.34
492.23800 cRT=27.55

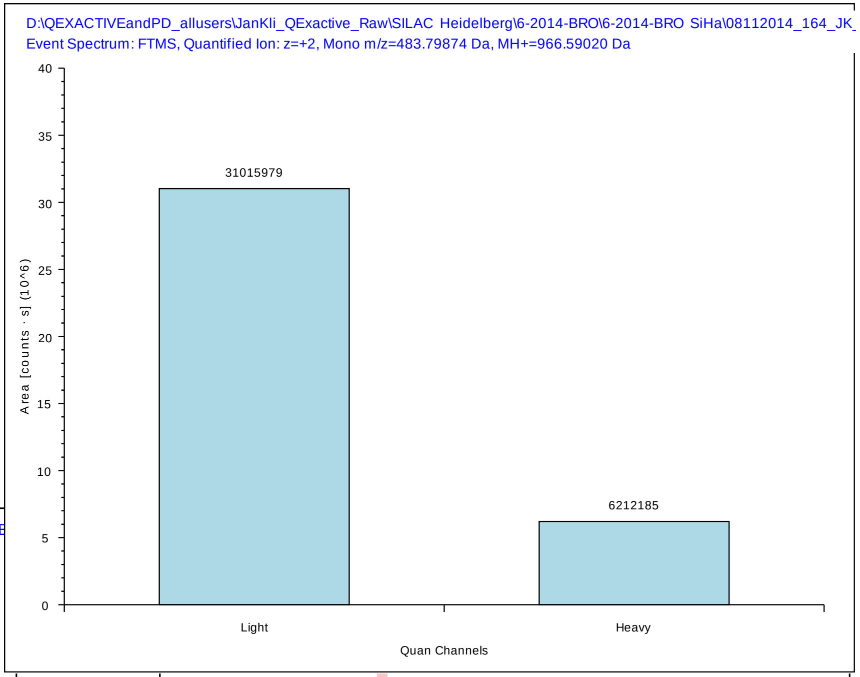
D:\QEXACTIVEandPD_allusers\JanKli_QExactive_Raw\SILAC Heidelberg\6-2014-BRO\6-2014-BRO SiHa\08112014_164_JK_
Event Spectrum: FTMS, Quantified Ion: z=+2, Mono m/z=483.79874 Da, MH+=966.59020 Da
Area [counts · s] (10^6)
31015979
6212185
Light
Heavy
Quan Channels

Sequence: FLVTLR, Charge: +2, Monoisotopic m/z: 374.73914 Da (-0.31 mmu/-0.84 ppm), MH+: 748.47099 Da, RT: 31.35 min,
Identified with: Mascot (v1.30); IonScore:25, Exp Value:4.0E-001, Ions matched by search engine: 5/40
Fragment match tolerance used for search: 0.02 Da

#1	b^+	b^{2+}	Seq.	y^+	y^{2+}	#2
1	148.07570	74.54149	F			6
2	261.15977	131.08352	L	601.40320	301.20524	5
3	360.22819	180.61773	V	488.31913	244.66320	4
4	461.27587	231.14157	T	389.25071	195.12899	3
5	574.35994	287.68361	L	288.20303	144.60515	2
6			R	175.11896	88.06312	1

FLVTLR

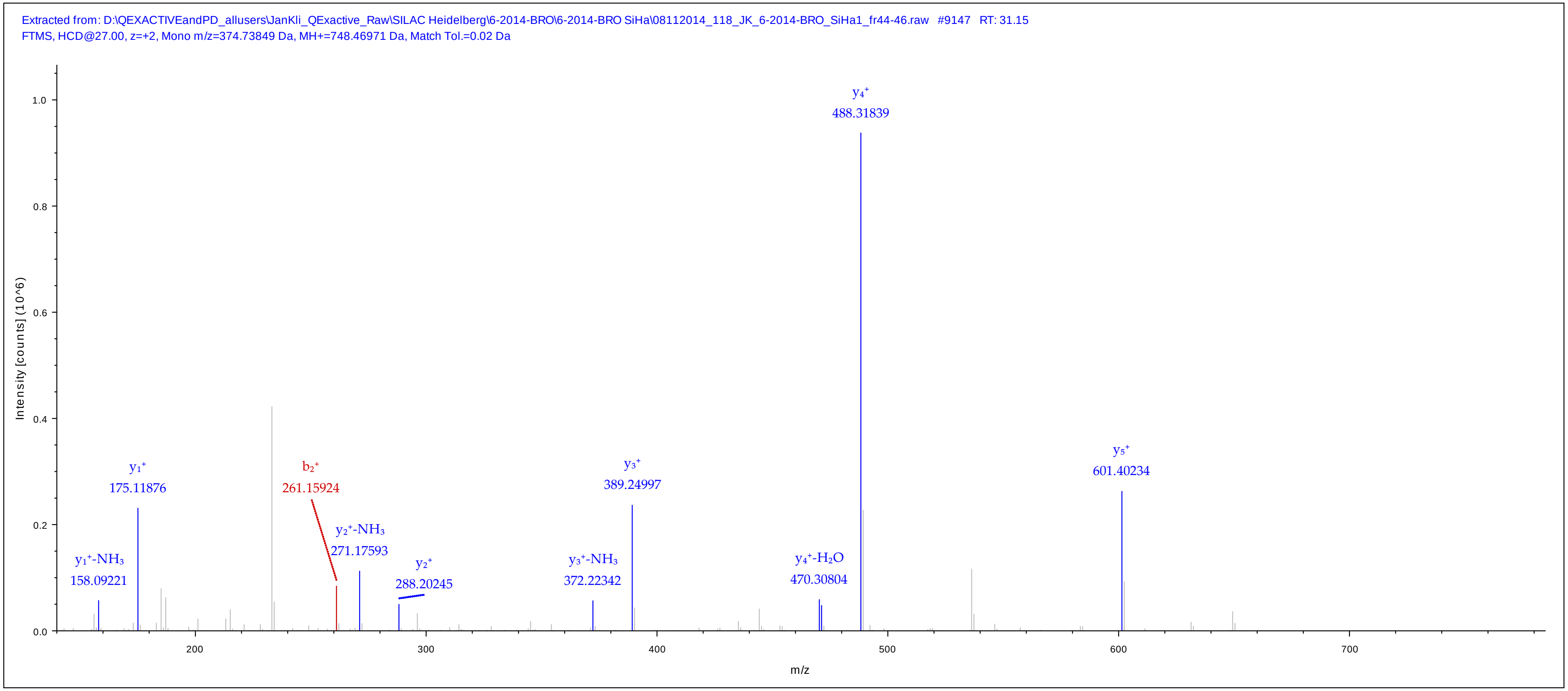

Extracted from: D:\QEXACTIVEandPD_allusers\JanKli_QExactive_Raw\SILAC Heidelberg\6-2014-BRO\6-2014-BRO SiHa\08112014_118_JK_6-2014-BRO_SiHa1_fr44-46.raw #9147 RT: 31.15
FTMS, HCD@27.00, z=+2, Mono m/z=374.73849 Da, MH+=748.46971 Da, Match Tol.=0.02 Da
Intensity [counts] (10^6)
m/z
y4+ 488.31839
y1+ 175.11876
b2+ 261.15924
y3+ 389.24997
y5+ 601.40234
y1+-NH3 158.09221
y2+-NH3 271.17593
y2+ 288.20245
y3+-NH3 372.22342
y4+-H2O 470.30804

FLVTLR

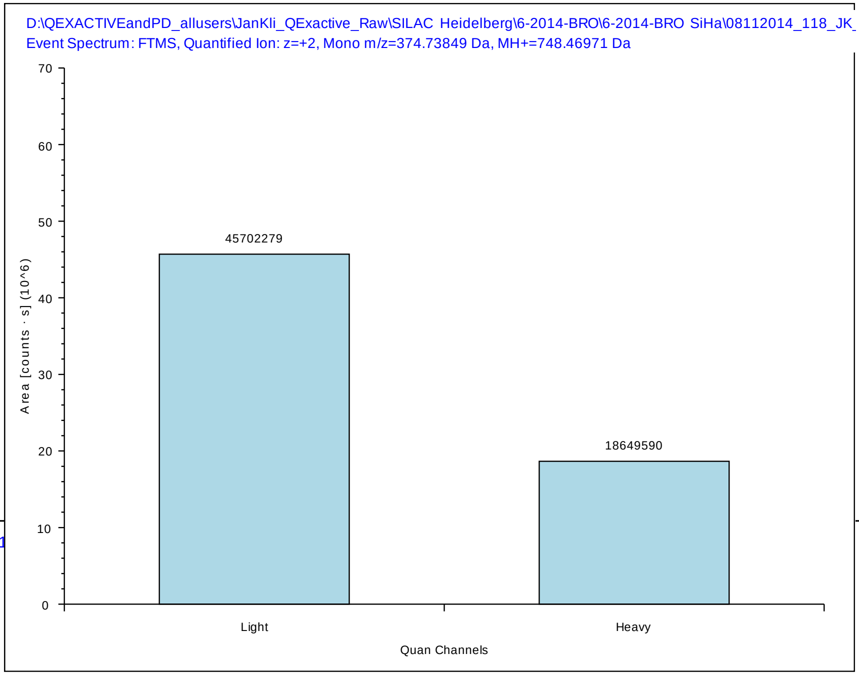

D:\QEXACTIVEandPD_allusers\JanKli_QExactive_Raw\SILAC Heidelberg\6-2014-BRO\6-2014-BRO SiHa\08112014_118_JK_
Event Spectrum: FTMS, Quantified Ion: z=+2, Mono m/z=374.73849 Da, MH+=748.46971 Da
Area [counts · s] (10^6)
70
60
50
40
30
20
10
0
45702279
18649590
Light
Heavy
Quan Channels

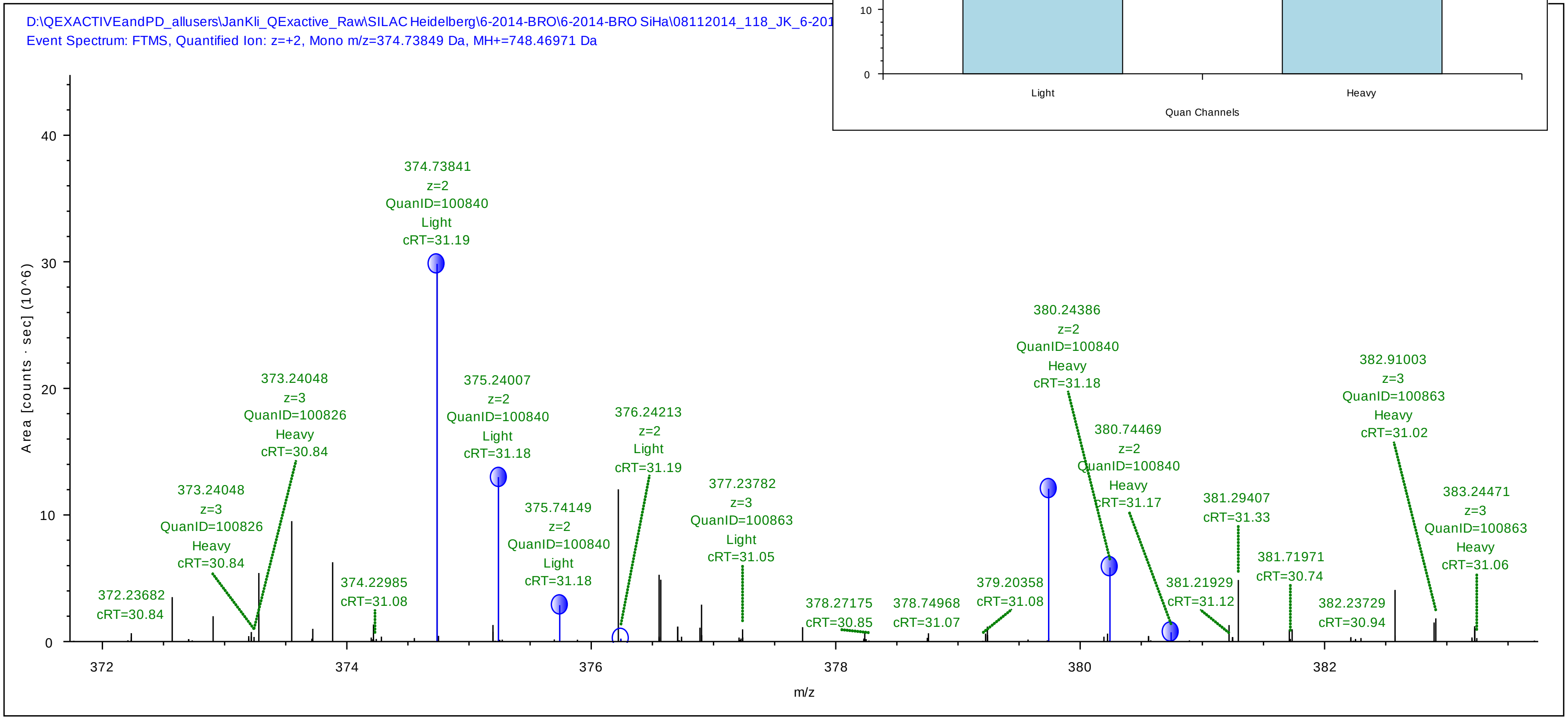

D:\QEXACTIVEandPD_allusers\JanKli_QExactive_Raw\SILAC Heidelberg\6-2014-BRO\6-2014-BRO SiHa\08112014_118_JK_6-201
Event Spectrum: FTMS, Quantified Ion: z=+2, Mono m/z=374.73849 Da, MH+=748.46971 Da
Area [counts · sec] (10^6)
40
30
20
10
0
374.73841 z=2 QuanID=100840 Light cRT=31.19
373.24048 z=3 QuanID=100826 Heavy cRT=30.84
375.24007 z=2 QuanID=100840 Light cRT=31.18
376.24213 z=2 Light cRT=31.19
373.24048 z=3 QuanID=100826 Heavy cRT=30.84
375.74149 z=2 QuanID=100840 Light cRT=31.18
377.23782 z=3 QuanID=100863 Light cRT=31.05
380.24386 z=2 QuanID=100840 Heavy cRT=31.18
380.74469 z=2 QuanID=100840 Heavy cRT=31.17
382.91003 z=3 QuanID=100863 Heavy cRT=31.02
381.29407 cRT=31.33
383.24471 z=3 QuanID=100863 Heavy cRT=31.06
372.23682 cRT=30.84
374.22985 cRT=31.08
378.27175 cRT=30.85
378.74968 cRT=31.07
379.20358 cRT=31.08
381.21929 cRT=31.12
381.71971 cRT=30.74
382.23729 cRT=30.94
372
374
376
378
380
382
m/z

Cytochrome c oxidase assembly protein COX19 OS=Homo sapiens GN=COX19 PE=1 SV=1 - [COX19_HUMAN]

Identified peptides:

LGFGDLTSGK
LMLQEPLEK

Coverage | ProteinCard

Cytochrome c oxidase assembly protein COX19 OS=Homo sapiens GN=COX19 PE=1 SV=1 - [COX19_HUMAN]

- [ ] Annotate PTMs reported in Uniprot
- [ ] Show only PTMs
- [ ] Include PSMs that are filtered Out

Coverage: 21.11%

Found Modifications:

L Label:13C(6)15N(2) (K)

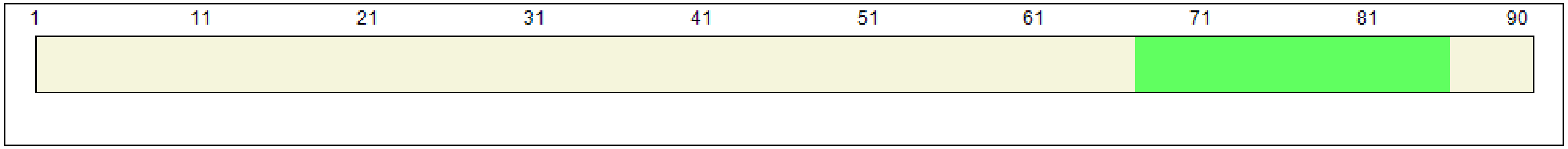

Sequence | Modification List

```
     1         11         21         31         41         51         61         71
1                                                                          L            L
MSTAMNFGTK SFQPRPPDKG SFPLDHLGEC KSFKEKFMKC LHNNNFENAL CRKESKEYLE CRMERKLMLQ EPLEKLGFGD LTSGKSEAKK
```

Sequence: LGFGDLTSGK, Charge: +2, Monoisotopic m/z: 497.76309 Da (-0.77 mmu/-1.54 ppm), MH+: 994.51891 Da, RT: 32.75 min,
Identified with: Mascot (v1.30); IonScore:66, Exp Value:4.7E-005, Ions matched by search engine: 7/78
Fragment match tolerance used for search: 0.02 Da

#1	b^+	b^{2+}	Seq.	y^+	y^{2+}	#2
1	114.09135	57.54931	L			10
2	171.11282	86.06005	G	881.43637	441.22182	9
3	318.18124	159.59426	F	824.41490	412.71109	8
4	375.20271	188.10499	G	677.34648	339.17688	7
5	490.22966	245.61847	D	620.32501	310.66614	6
6	603.31373	302.16050	L	505.29806	253.15267	5
7	704.36141	352.68434	T	392.21399	196.61063	4
8	791.39344	396.20036	S	291.16631	146.08679	3
9	848.41491	424.71109	G	204.13428	102.57078	2
10			K	147.11281	74.06004	1

LGFGDLTSGK

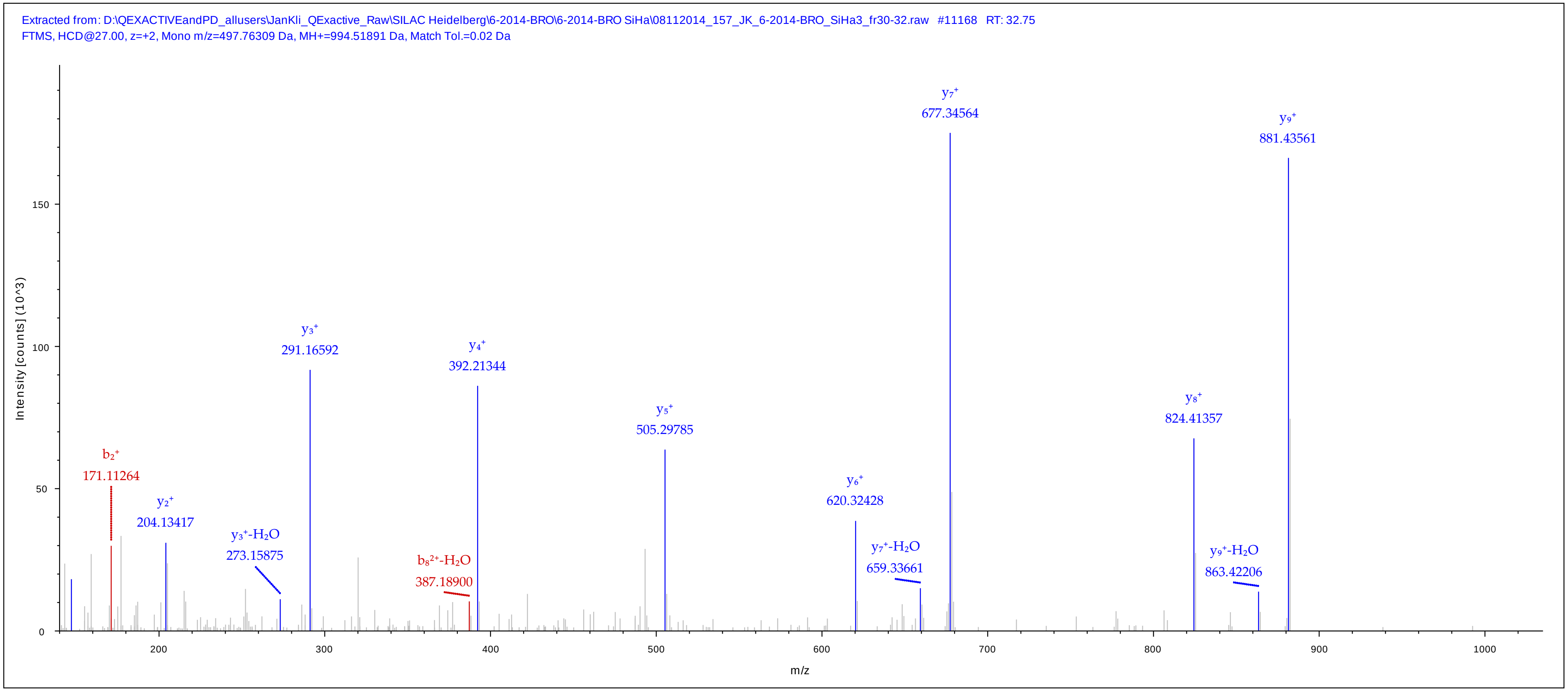

Extracted from: D:\QEXACTIVEandPD_allusers\JanKli_QExactive_Raw\SILAC Heidelberg\6-2014-BRO\6-2014-BRO SiHa\08112014_157_JK_6-2014-BRO_SiHa3_fr30-32.raw #11168 RT: 32.75
FTMS, HCD@27.00, z=+2, Mono m/z=497.76309 Da, MH+=994.51891 Da, Match Tol.=0.02 Da
y7+
677.34564
y9+
881.43561
y3+
291.16592
y4+
392.21344
y5+
505.29785
y8+
824.41357
b2+
171.11264
y2+
204.13417
y6+
620.32428
y3+-H2O
273.15875
b8 2+-H2O
387.18900
y7+-H2O
659.33661
y9+-H2O
863.42206
Intensity [counts] (10^3)
m/z

LGFGDLTSGK

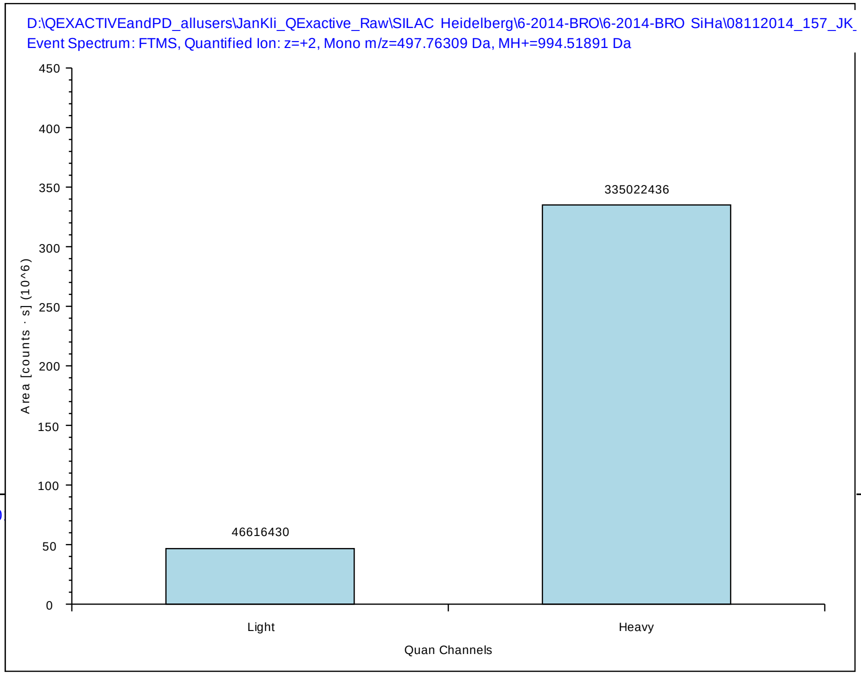
D:\QEXACTIVEandPD_allusers\JanKli_QExactive_Raw\SILAC Heidelberg\6-2014-BRO\6-2014-BRO SiHa\08112014_157_JK_
Event Spectrum: FTMS, Quantified Ion: z=+2, Mono m/z=497.76309 Da, MH+=994.51891 Da
Area [counts · s] (10^6)
450
400
350
300
250
200
150
100
50
0
46616430
335022436
Light
Heavy
Quan Channels

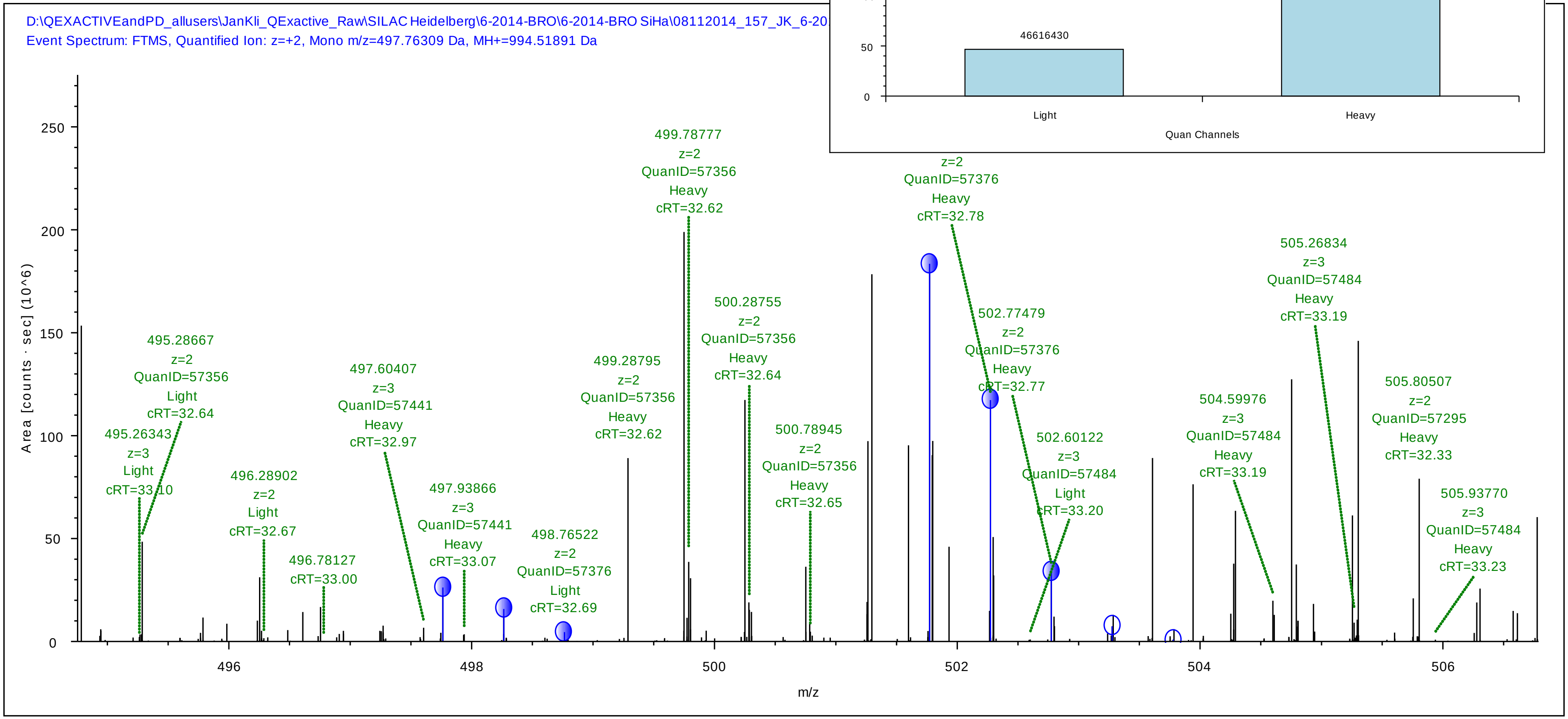
D:\QEXACTIVEandPD_allusers\JanKli_QExactive_Raw\SILAC Heidelberg\6-2014-BRO\6-2014-BRO SiHa\08112014_157_JK_6-20
Event Spectrum: FTMS, Quantified Ion: z=+2, Mono m/z=497.76309 Da, MH+=994.51891 Da
Area [counts · sec] (10^6)
250
200
150
100
50
0
495.26343 z=3 Light cRT=33.10
495.28667 z=2 QuanID=57356 Light cRT=32.64
496.28902 z=2 Light cRT=32.67
496.78127 cRT=33.00
497.60407 z=3 QuanID=57441 Heavy cRT=32.97
497.93866 z=3 QuanID=57441 Heavy cRT=33.07
498.76522 z=2 QuanID=57376 Light cRT=32.69
499.28795 z=2 QuanID=57356 Heavy cRT=32.62
499.78777 z=2 QuanID=57356 Heavy cRT=32.62
500.28755 z=2 QuanID=57356 Heavy cRT=32.64
500.78945 z=2 QuanID=57356 Heavy cRT=32.65
z=2 QuanID=57376 Heavy cRT=32.78
502.77479 z=2 QuanID=57376 Heavy cRT=32.77
502.60122 z=3 QuanID=57484 Light cRT=33.20
504.59976 z=3 QuanID=57484 Heavy cRT=33.19
505.26834 z=3 QuanID=57484 Heavy cRT=33.19
505.80507 z=2 QuanID=57295 Heavy cRT=32.33
505.93770 z=3 QuanID=57484 Heavy cRT=33.23
496
498
500
502
504
506
m/z

Alpha-crystallin B chain OS=Homo sapiens GN=CRYAB PE=1 SV=2 - [CRYAB_HUMAN]

Identified peptides:

QDEHGFISR
RPFFPFHSPSR
APSWFDTGLSEMR
VLGDVIEVHGK
IPADVDPLTITSSLSSDGVLTVNGPR
HFSPEELK

Coverage | ProteinCard

Alpha-crystallin B chain OS=Homo sapiens GN=CRYAB PE=1 SV=2 - [CRYAB_HUMAN]

- [ ] Annotate PTMs reported in Uniprot
- [ ] Show only PTMs
- [ ] Include PSMs that are filtered Out

Coverage: 44.57%

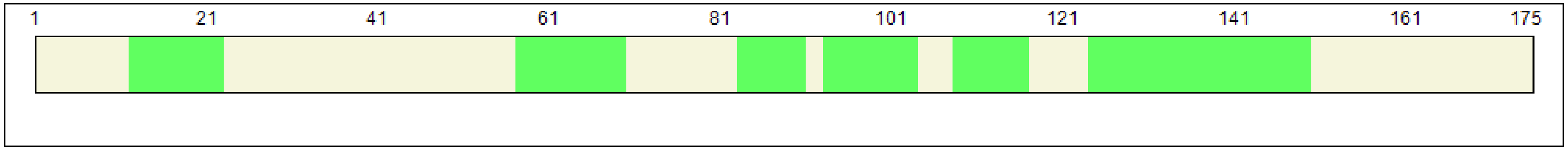

Found Modifications:

Sequence | Modification List

	1	11	21	31	41	51	61	71	81	91	101
1	MDIAIHHPWI	R**RPFFPFHSP**	**SR**LFDQFFGE	HLLESDLFPT	STSLSPFYLR	PPSFLR**APSW**	**FDTGLSEMR**L	EKDRFSVNLD	VK**HFSPEELK**	VK**VLGDVIEV**	**HGK**HEER**QDE**
111	**HGFISR**EFHR	KYR**IPADVDP**	**LTITSSLSSD**	**GVLTVNGPR**K	QVSGPERTIP	ITREEKPAVT	AAPKK				

Sequence: RPFFPFHSPSR, Charge: +3, Monoisotopic m/z: 458.90656 Da (-0.5 mmu/-1.1 ppm), MH+: 1374.70511 Da, RT: 25.11 min,
Identified with: Mascot (v1.30); IonScore:46, Exp Value:6.1E-003, Ions matched by search engine: 13/104
Fragment match tolerance used for search: 0.02 Da

#1	b^{+}	b^{2+}	b^{3+}	Seq.	y^{+}	y^{2+}	y^{3+}	#2
1	157.10840	79.05784	53.04098	R				11
2	254.16117	127.58422	85.39191	P	1218.60550	609.80639	406.87335	10
3	401.22959	201.11843	134.41471	F	1121.55273	561.28000	374.52243	9
4	548.29801	274.65264	183.43752	F	974.48431	487.74579	325.49962	8
5	645.35078	323.17903	215.78844	P	827.41589	414.21158	276.47681	7
6	792.41920	396.71324	264.81125	F	730.36312	365.68520	244.12589	6
7	929.47811	465.24269	310.49755	H	583.29470	292.15099	195.10308	5
8	1016.51014	508.75871	339.50823	S	446.23579	223.62153	149.41678	4
9	1113.56291	557.28509	371.85915	P	359.20376	180.10552	120.40610	3
10	1200.59494	600.80111	400.86983	S	262.15099	131.57913	88.05518	2
11				R	175.11896	88.06312	59.04450	1

RPFFPFHSPSR

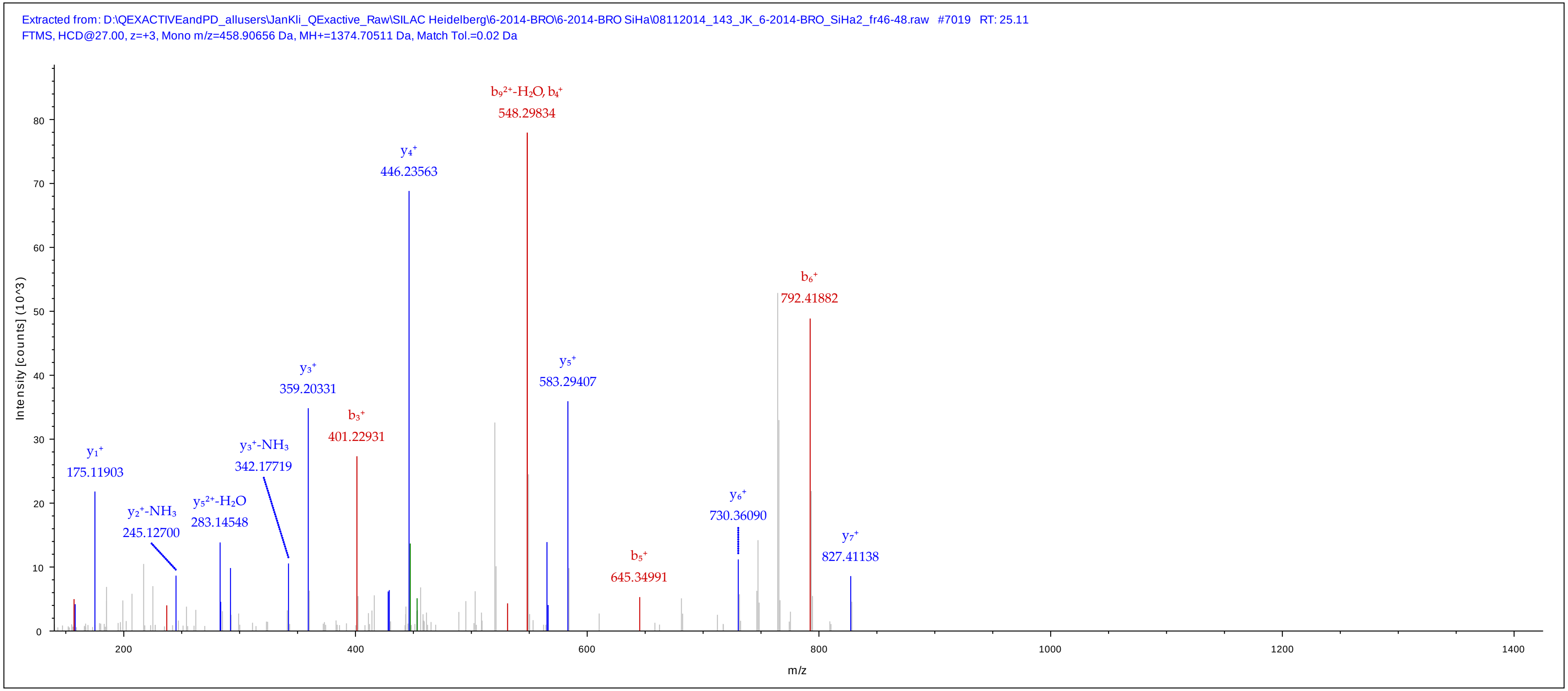

RPFFPFHSPSR

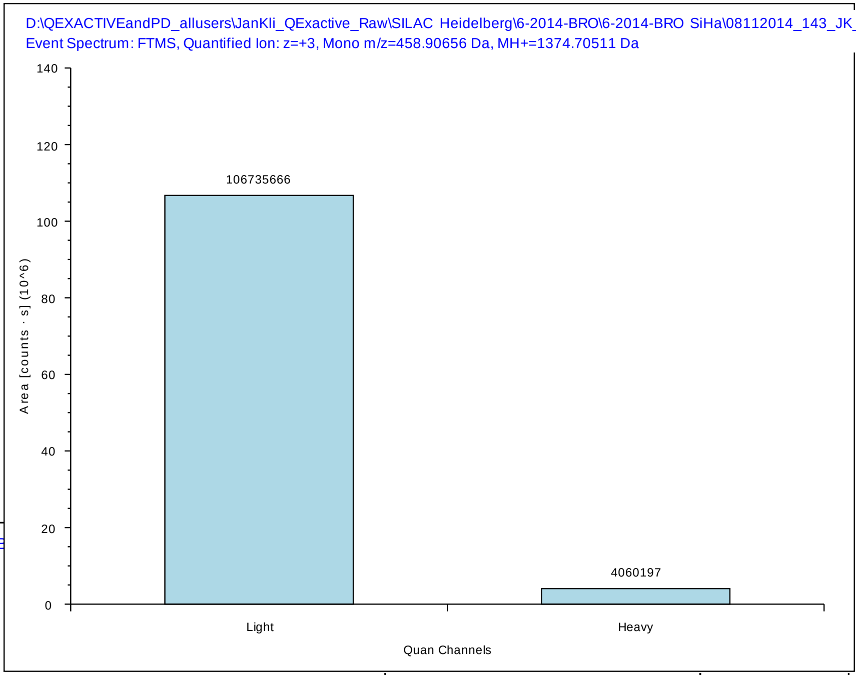

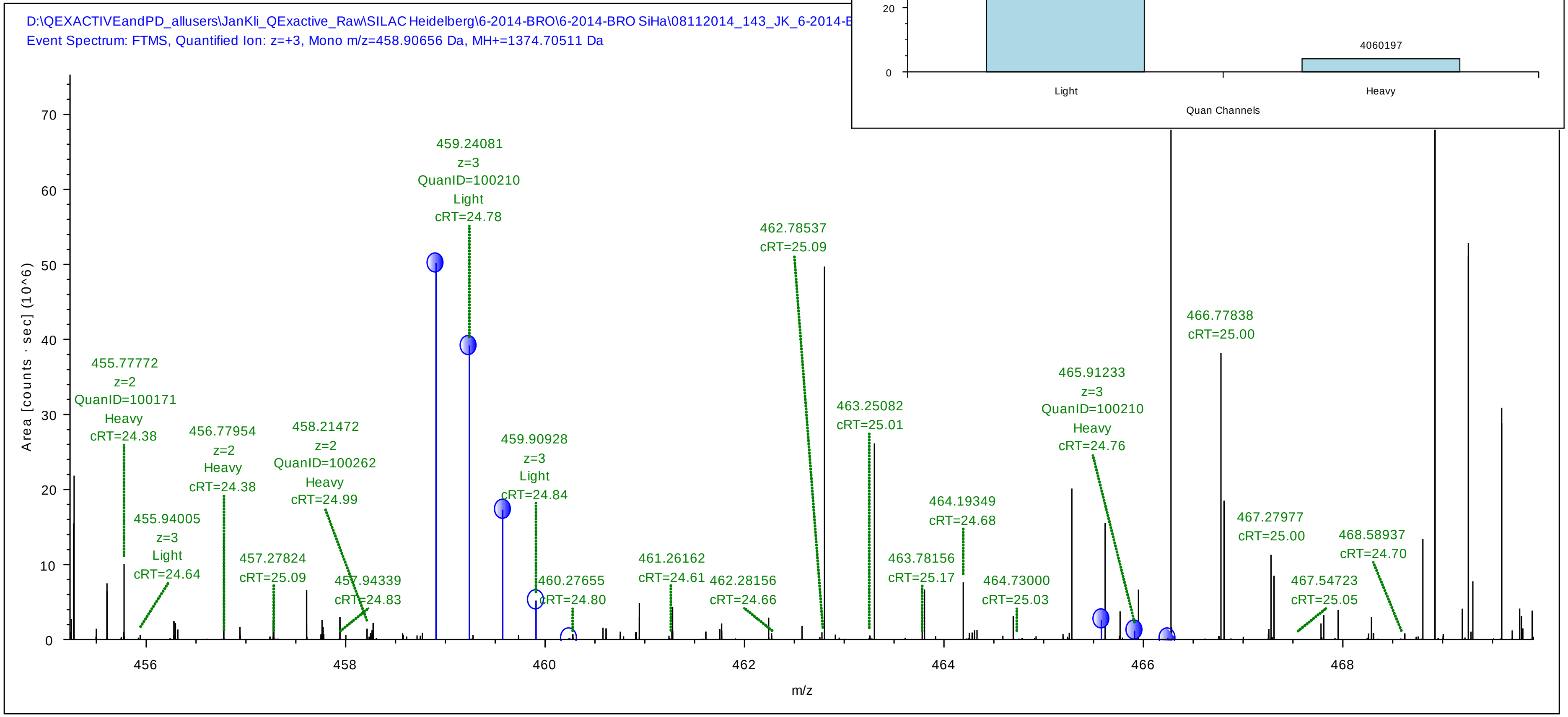

2'-5'-oligoadenylate synthase-like protein OS=Homo sapiens GN=OASL PE=1 SV=2 - [OASL_HUMAN]

Identified peptides:

ACGGPGNFCPSFSELQR
ERPIILDPADPTLNVAEGYR
ALMQELYSTPASR
LDSFVAQWLQPHR
EWKEEVLDAVR
GTAEPITVTIVPAYR
ALGPSLPNSQPPPEVYVSLIK
TMWQSQDLLDLGLEDLR
DIHLTVEQR
NPDGGSYAYAINPNSFILGLK
TVEEFLR

Coverage | ProteinCard

2'-5'-oligoadenylate synthase-like protein OS=Homo sapiens GN=OASL PE=1 SV=2 - [OASL_HUMAN]

- [ ] Annotate PTMs reported in Uniprot
- [ ] Show only PTMs
- [ ] Include PSMs that are filtered Out

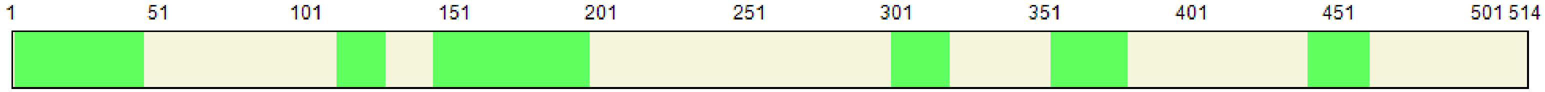

Coverage: 35.21%

Found Modifications:

A Acetyl (Protein N-term)
C Carbamidomethyl (C)
L Label:13C(6)15N(4) (R)

Sequence | Modification List

```
     1          11         21         31         41         51         61         71         81         91         101
  1   A               L
     MALMQELYST PASRLDSFVA QWLQPHREWK EEVLDAVRTV EEFLRQEHFQ GKRGLDQDVR VLKVVKVGSF GNGTVLRSTR EVELVAFLSC FHSFQEAAKH HKDVLRLIWK
111                                                                              C        C
     TMWQSQDLLD LGLEDLRMEQ RVPDALVFTI QTRGTAEPIT VTIVPAYRAL GPSLPNSQPP PEVYVSLIKA CGGPGNFCPS FSELQRNFVK HRPTKLKSLL RLVKHWYQQY
221
     VKARSPRANL PPLYALELLT IYAWEMGTEE DENFMLDEGF TTVMDLLLEY EVICIYWTKY YTLHNAIIED CVRKQLKKER PIILDPADPT LNVAEGYRWD IVAQRASQCL
331
     KQDCCYDNRE NPISSWNVKR ARDIHLTVEQ RGYPDENLIV NPYEPIRKVK EKIRRTRGYS GLQRLSFQVP GSERQLLSSR CSLAKYGIFS HTHIYLLETI PSEIQVFVKN
441
     PDGGSYAYAI NPNSFILGLK QQIEDQQGLP KKQQQLEFQG QVLQDWLGLG IYGIQDSDTL ILSKKKGEAL FPAS
```

Sequence: ALMQELYSTPASR, A1-Acetyl (42.01057 Da), R13-Label:13C(6)15N(4) (10.00827 Da)
Charge: +2, Monoisotopic m/z: 759.87311 Da (-5.38 mmu/-7.08 ppm), MH+: 1518.73894 Da, RT: 49.65 min,
Identified with: Mascot (v1.30); IonScore:48, Exp Value:3.0E-003, Ions matched by search engine: 7/128
Fragment match tolerance used for search: 0.02 Da

#1	b^{+}	b^{2+}	Seq.	y^{+}	y^{2+}	#2
1	114.05496	57.53112	A-Acetyl			13
2	227.13903	114.07315	L	1405.70200	703.35464	12
3	358.17953	179.59340	M	1292.61793	646.81260	11
4	486.23811	243.62269	Q	1161.57743	581.29235	10
5	615.28071	308.14399	E	1033.51885	517.26306	9
6	728.36478	364.68603	L	904.47625	452.74176	8
7	891.42810	446.21769	Y	791.39218	396.19973	7
8	978.46013	489.73370	S	628.32886	314.66807	6
9	1079.50781	540.25754	T	541.29683	271.15205	5
10	1176.56058	588.78393	P	440.24915	220.62821	4
11	1247.59770	624.30249	A	343.19638	172.10183	3
12	1334.62973	667.81850	S	272.15926	136.58327	2
13			R-Label:13C(6)15N(4)	185.12723	93.06725	1

ALMQELYSTPASR

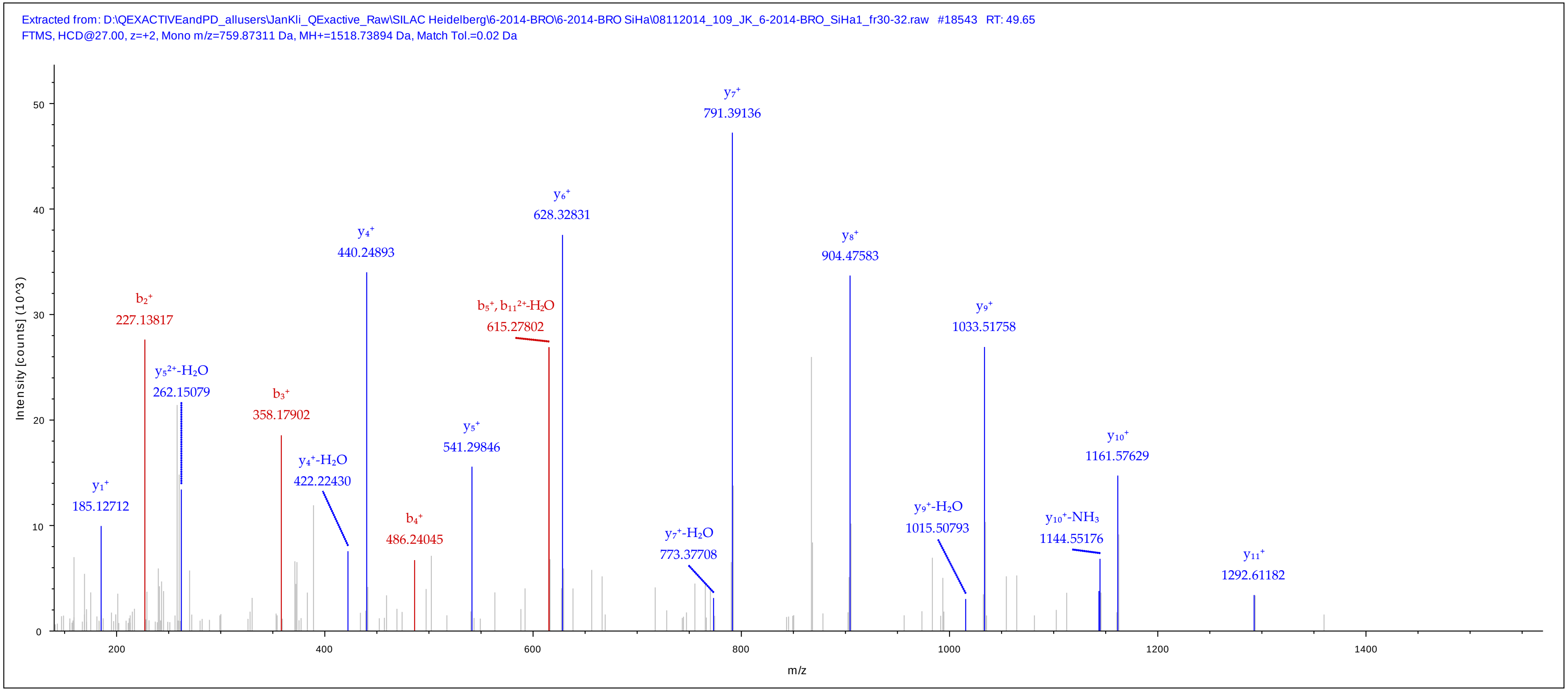

ALMQELYSTPASR

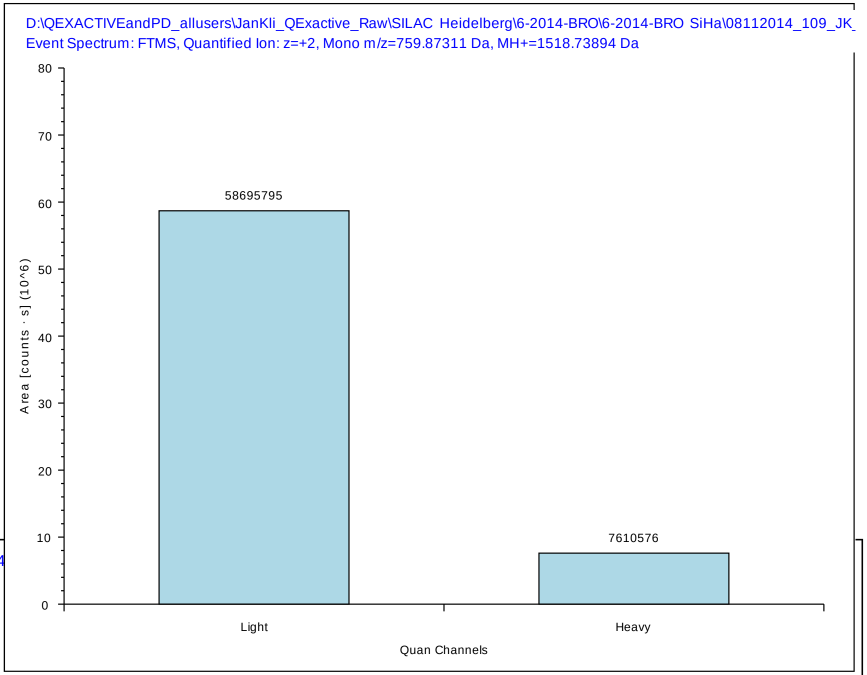

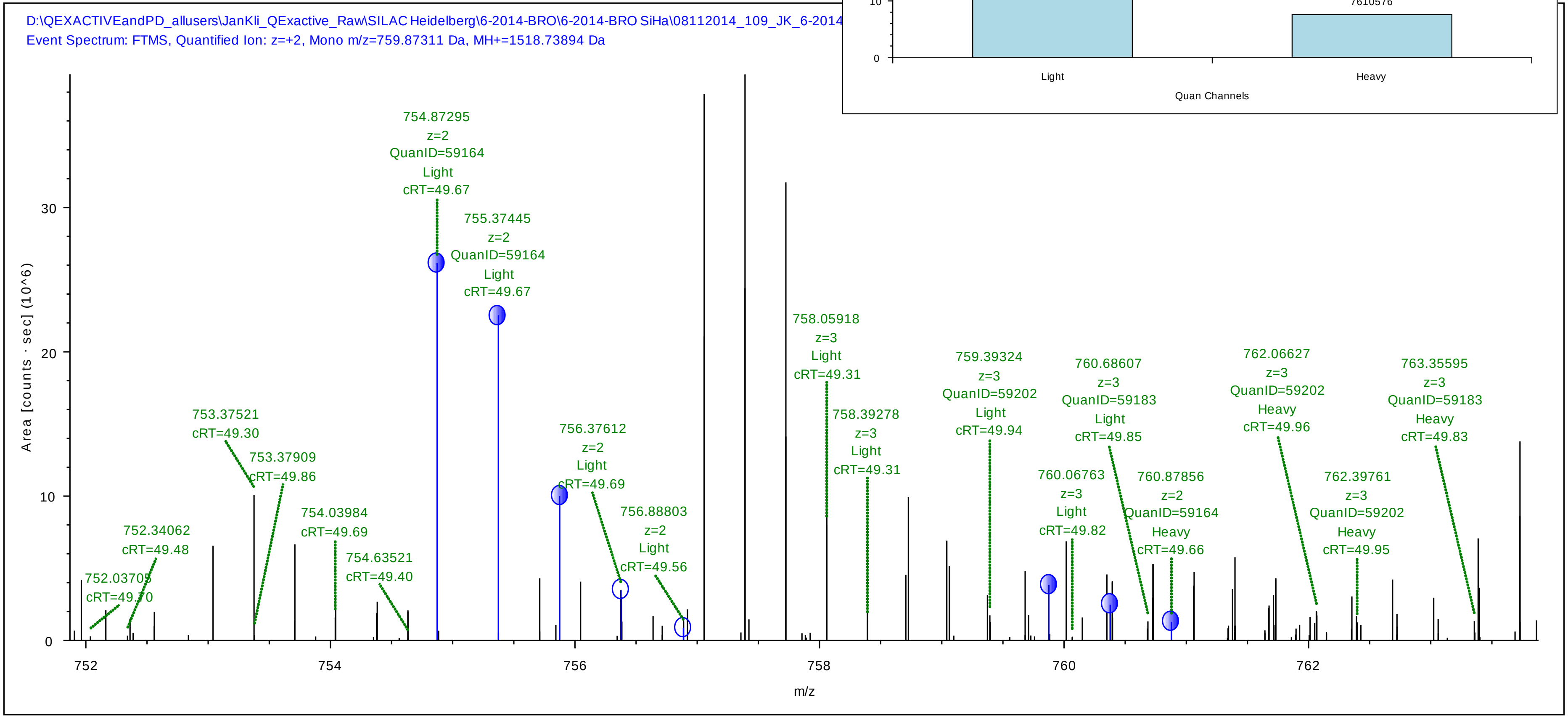

Sequence: LDSFVAQWLQPHR, Charge: +3, Monoisotopic m/z: 532.94666 Da (-0.93 mmu/-1.75 ppm), MH+: 1596.82541 Da, RT: 42.66 min,
Identified with: Mascot (v1.30); IonScore:52, Exp Value:1.8E-003, Ions matched by search engine: 7/110
Fragment match tolerance used for search: 0.02 Da

#1	b^+	b^{2+}	b^{3+}	Seq.	y^+	y^{2+}	y^{3+}	#2
1	114.09135	57.54931	38.70197	L				13
2	229.11830	115.06279	77.04428	D	1483.74413	742.37570	495.25289	12
3	316.15033	158.57880	106.05496	S	1368.71718	684.86223	456.91058	11
4	463.21875	232.11301	155.07777	F	1281.68515	641.34621	427.89990	10
5	562.28717	281.64722	188.10057	V	1134.61673	567.81200	378.87709	9
6	633.32429	317.16578	211.77961	A	1035.54831	518.27779	345.85429	8
7	761.38287	381.19507	254.46581	Q	964.51119	482.75923	322.17525	7
8	947.46219	474.23473	316.49225	W	836.45261	418.72994	279.48905	6
9	1060.54626	530.77677	354.18694	L	650.37329	325.69028	217.46261	5
10	1188.60484	594.80606	396.87313	Q	537.28922	269.14825	179.76792	4
11	1285.65761	643.33244	429.22405	P	409.23064	205.11896	137.08173	3
12	1422.71652	711.86190	474.91036	H	312.17787	156.59257	104.73081	2
13				R	175.11896	88.06312	59.04450	1

LDSFVAQWLQPHR

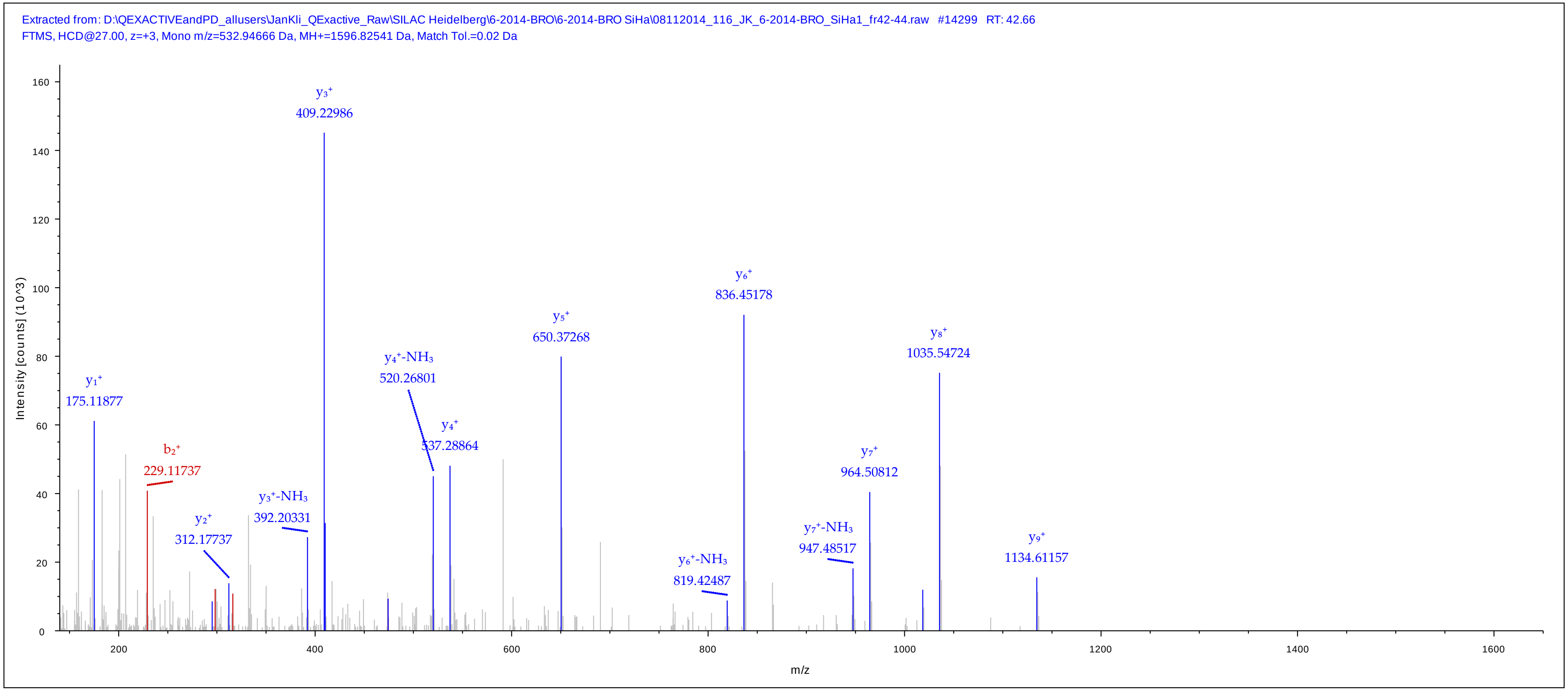

LDSFVAQWLQPHR

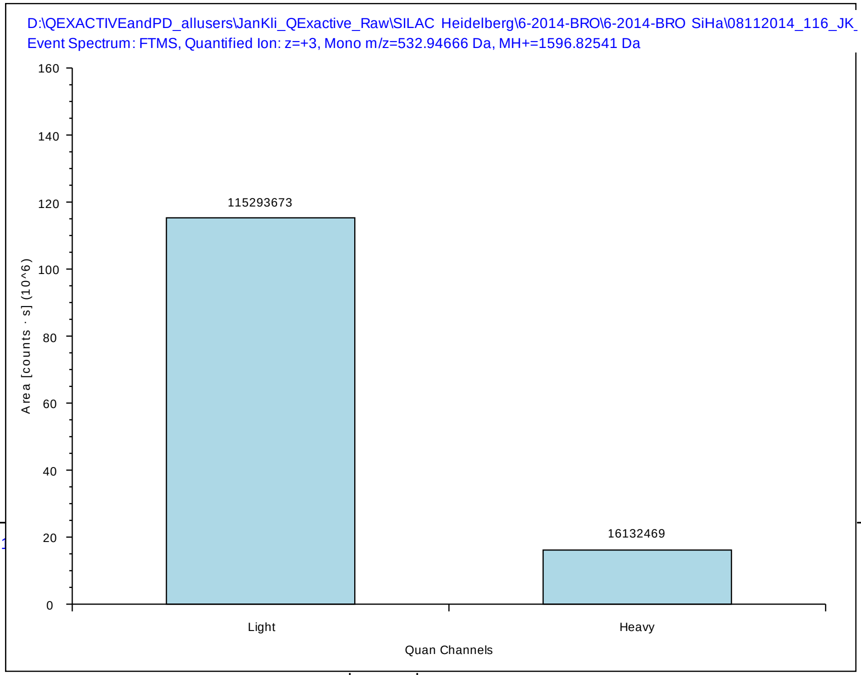

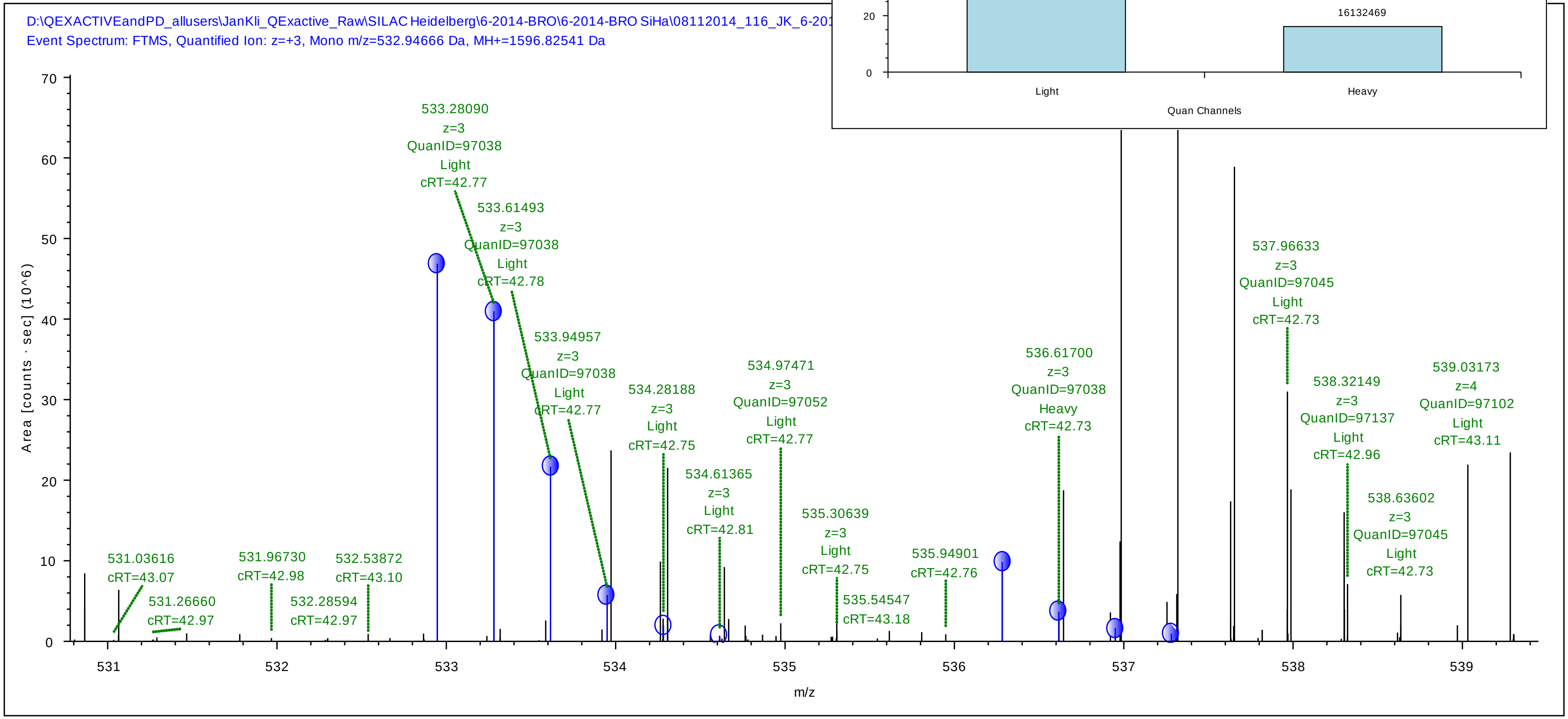

Tumor-associated calcium signal transducer 2 OS=Homo sapiens GN=TACSTD2 PE=1 SV=3 - [TACD2_HUMAN]

Identified peptides:

ALGSGMAVDCSTLTSK
AAGDVDIGDAAYYFER
TLVRPSEHALVDNDGLYDPDCDPEGR
THHILIDLR
VRGEPLQVER

Coverage | ProteinCard

Tumor-associated calcium signal transducer 2 OS=Homo sapiens GN=TACSTD2 PE=1 SV=3 - [TACD2_HUMAN]

- [ ] Annotate PTMs reported in Uniprot
- [ ] Show only PTMs
- [ ] Include PSMs that are filtered Out

Coverage: 23.84%

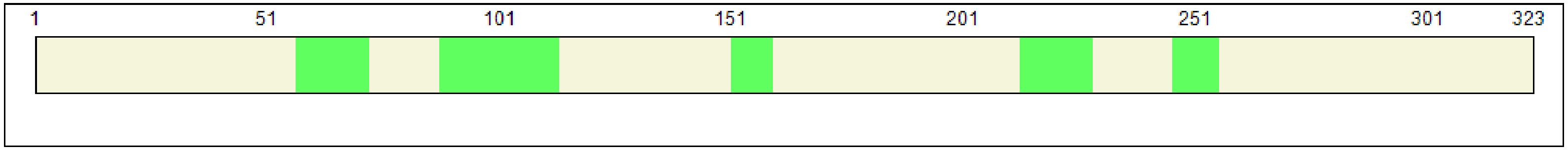

Found Modifications:

**C**	Carbamidomethyl (C)
**L**	Label:13C(6)15N(2) (K)
**M**	Label:13C(6)15N(4) (R)

Sequence | Modification List

```
      1          11         21         31         41         51         61         71         81         91         101
  1                                                                          C      L                                   C
     MARGPGLAPP PLRLPLLLLV LAAVTGHTAA QDNCTCPTNK MTVCSPDGPG GRCQCRALGS GMAVDCSTLT SKCLLLKARM SAPKNARTLV RPSEHALVDN DGLYDPDCDP
111
     EGRFKARQCN QTSVCWCVNS VGVRRTDKGD LSLRCDELVR THHILIDLRH RPTAGAFNHS DLDAELRRLF RERYRLHPKF VAAVHYEQPT IQIELRQNTS QKAAGDVDIG
221         M
     DAAYYFERDI KGESLFQGRG GLDLRVRGEP LQVERTLIYY LDEIPPKFSM KRLTAGLIAV IVVVVVALVA GMAVLVITNR RKSGKYKKVE IKELGELRKE PSL
```

Sequence: ALGSGMAVDCSTLTSK, C10-Carbamidomethyl (57.02146 Da)
Charge: +2, Monoisotopic m/z: 799.38068 Da (-1.02 mmu/-1.27 ppm), MH+: 1597.75408 Da, RT: 29.96 min,
Identified with: Mascot (v1.30); IonScore:93, Exp Value:9.0E-008, Ions matched by search engine: 13/142
Fragment match tolerance used for search: 0.02 Da

#1	b^{+}	b^{2+}	Seq.	y^{+}	y^{2+}	#2
1	72.04440	36.52584	A			16
2	185.12847	93.06787	L	1526.71899	763.86313	15
3	242.14994	121.57861	G	1413.63492	707.32110	14
4	329.18197	165.09462	S	1356.61345	678.81036	13
5	386.20344	193.60536	G	1269.58142	635.29435	12
6	517.24394	259.12561	M	1212.55995	606.78361	11
7	588.28106	294.64417	A	1081.51945	541.26336	10
8	687.34948	344.17838	V	1010.48233	505.74480	9
9	802.37643	401.69185	D	911.41391	456.21059	8
10	962.40708	481.70718	C-Carbamidomethyl	796.38696	398.69712	7
11	1049.43911	525.22319	S	636.35630	318.68179	6
12	1150.48679	575.74703	T	549.32427	275.16577	5
13	1263.57086	632.28907	L	448.27659	224.64193	4
14	1364.61854	682.81291	T	335.19252	168.09990	3
15	1451.65057	726.32892	S	234.14484	117.57606	2
16			K	147.11281	74.06004	1

ALGSGMAVDCSTLTSK

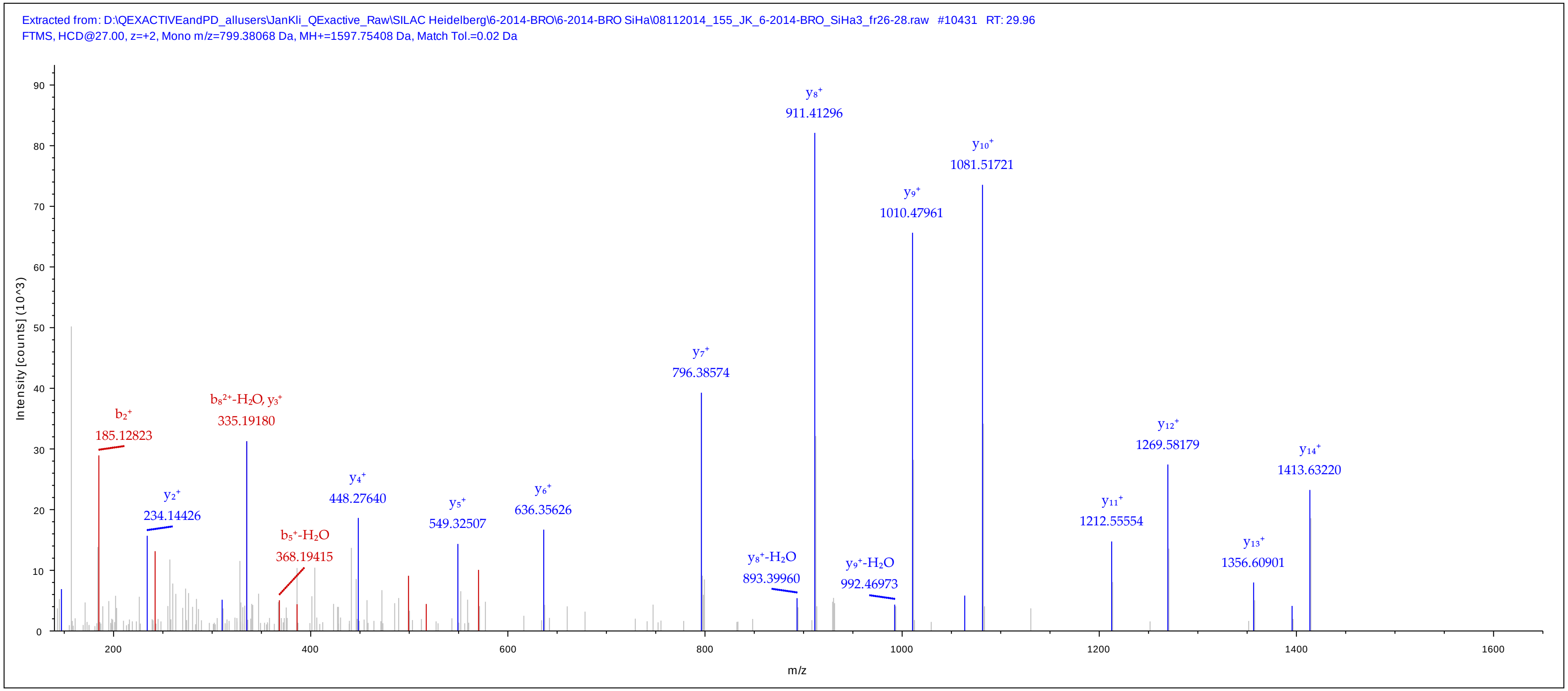

ALGSGMAVDCSTLTSK

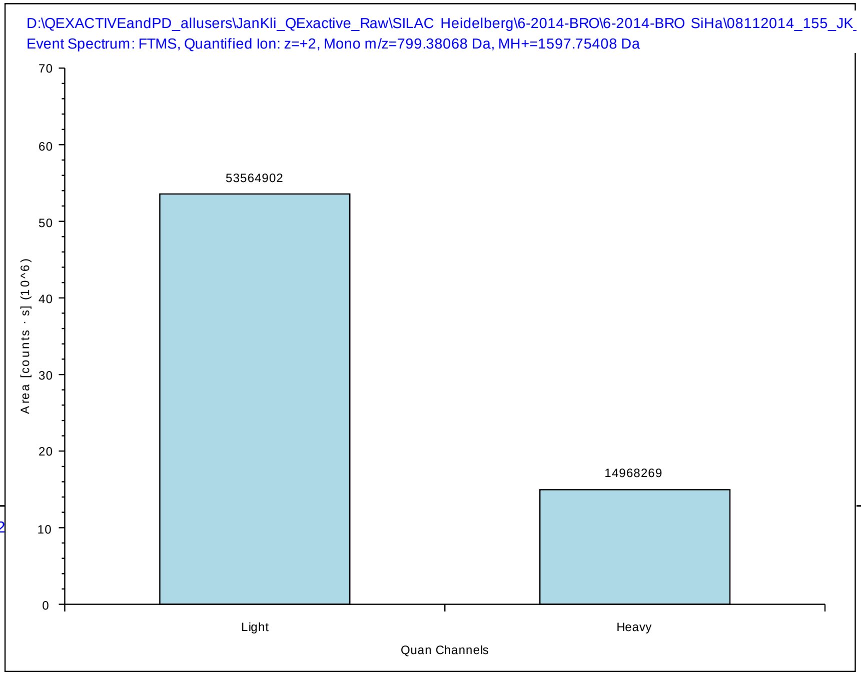

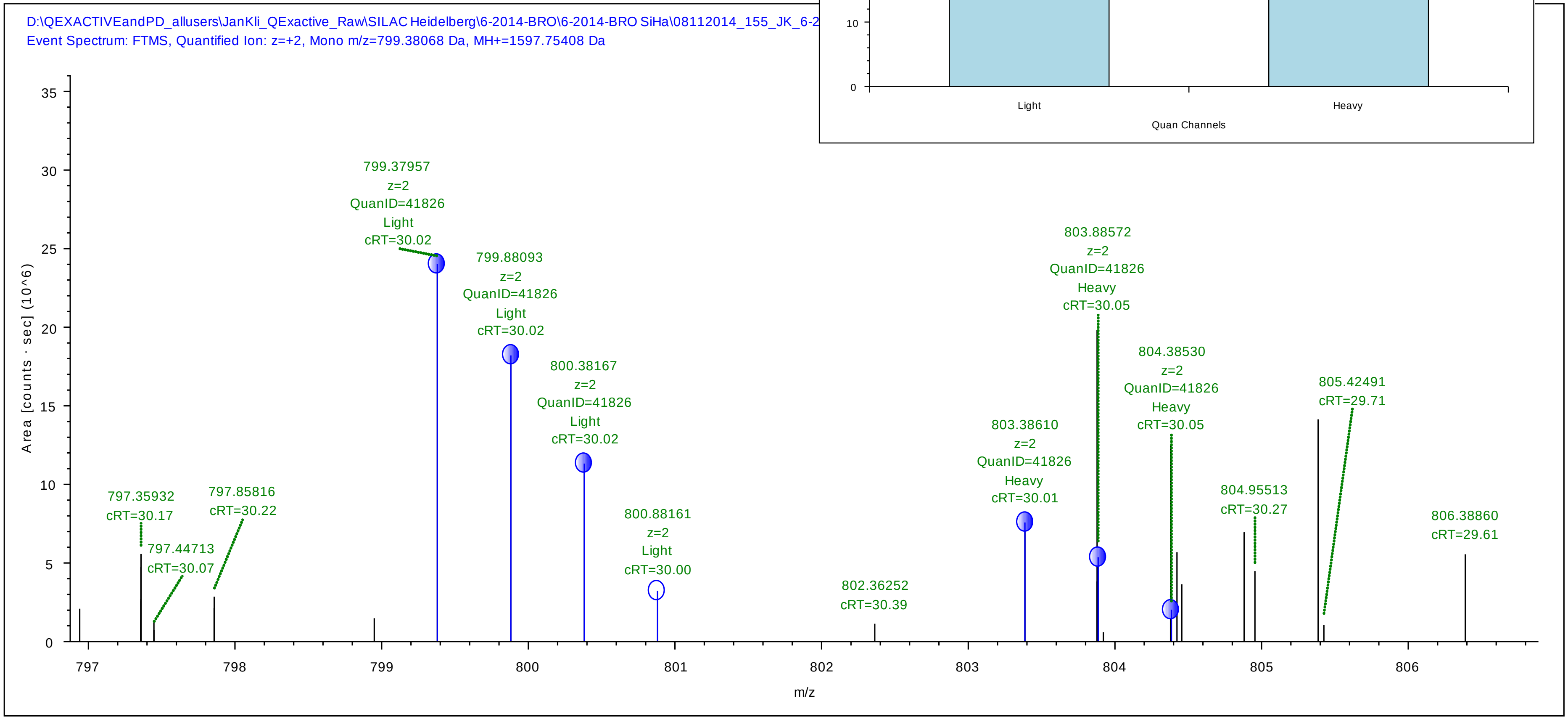

Sequence: AAGDVDIGDAAYYFER, Charge: +2, Monoisotopic m/z: 866.89063 Da (-3.71 mmu/-4.28 ppm), MH+: 1732.77397 Da, RT: 43.76 min,
Identified with: Mascot (v1.30); IonScore:87, Exp Value:1.6E-007, Ions matched by search engine: 12/142
Fragment match tolerance used for search: 0.02 Da

#1	b^{+}	b^{2+}	Seq.	y^{+}	y^{2+}	#2
1	72.04440	36.52584	A			16
2	143.08152	72.04440	A	1661.74426	831.37577	15
3	200.10299	100.55513	G	1590.70714	795.85721	14
4	315.12994	158.06861	D	1533.68567	767.34647	13
5	414.19836	207.60282	V	1418.65872	709.83300	12
6	529.22531	265.11629	D	1319.59030	660.29879	11
7	642.30938	321.65833	I	1204.56335	602.78531	10
8	699.33085	350.16906	G	1091.47928	546.24328	9
9	814.35780	407.68254	D	1034.45781	517.73254	8
10	885.39492	443.20110	A	919.43086	460.21907	7
11	956.43204	478.71966	A	848.39374	424.70051	6
12	1119.49536	560.25132	Y	777.35662	389.18195	5
13	1282.55868	641.78298	Y	614.29330	307.65029	4
14	1429.62710	715.31719	F	451.22998	226.11863	3
15	1558.66970	779.83849	E	304.16156	152.58442	2
16			R	175.11896	88.06312	1

AAGDVDIGDAAYYFER

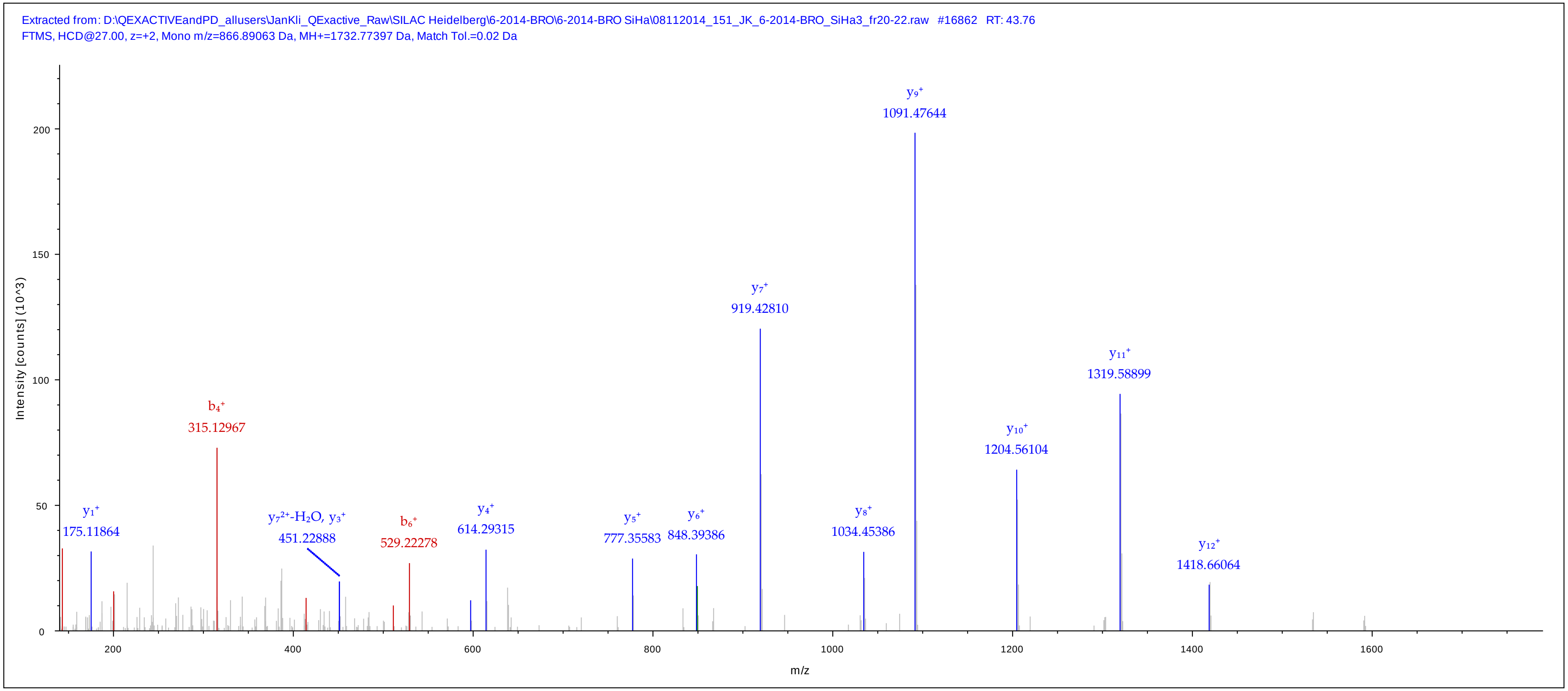

AAGDVDIGDAAYYFER

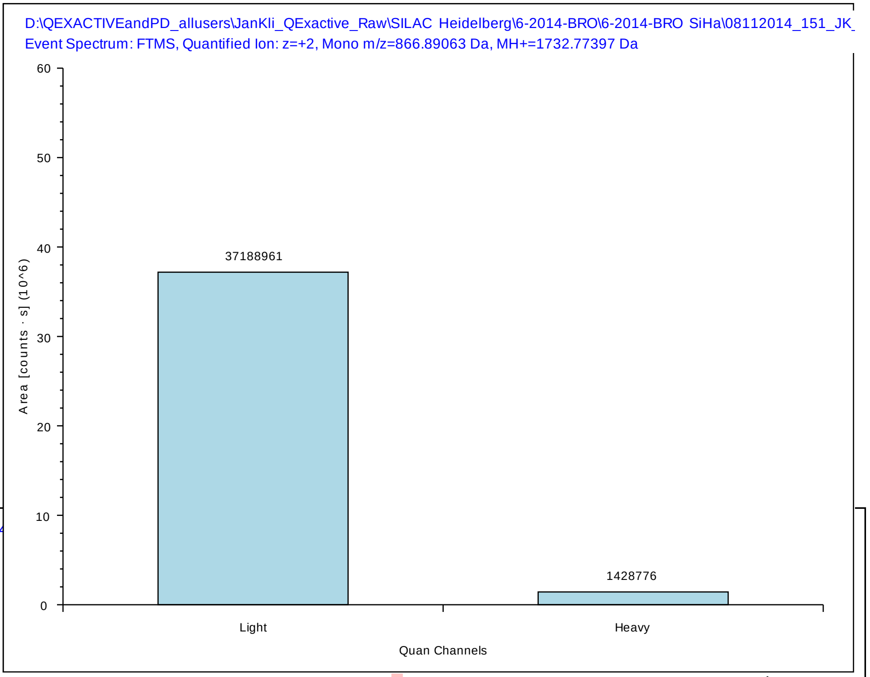

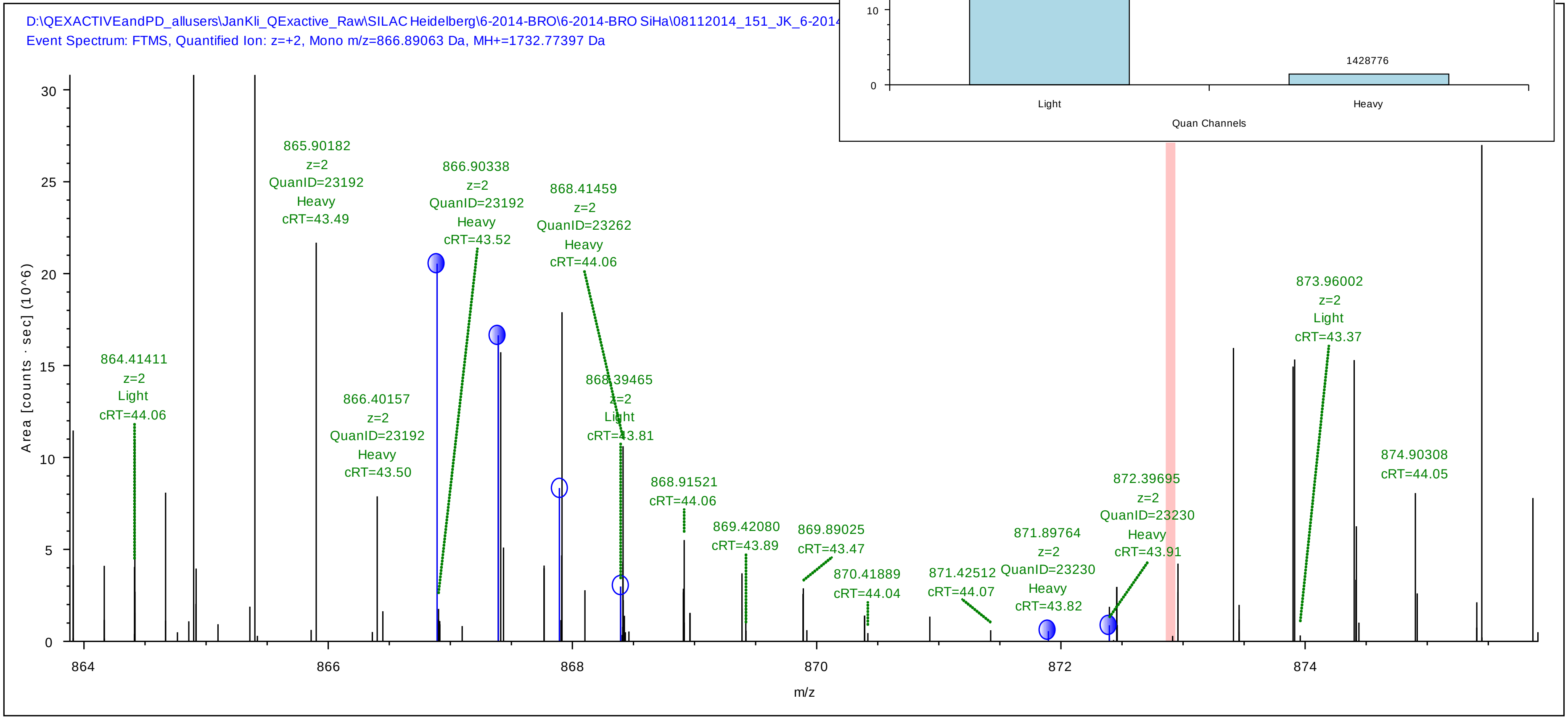

Sequence: TLVRPSEHALVDNDGLYDPDCDPEGR, C21-Carbamidomethyl (57.02146 Da)
Charge: +4,   Monoisotopic m/z: 735.84076 Da (-0.44 mmu/-0.6 ppm),   MH+: 2940.34121 Da,   RT: 31.96 min,
Identified with: Mascot (v1.30); IonScore:46, Exp Value:3.0E-003, Ions matched by search engine: 12/290
Fragment match tolerance used for search: 0.02 Da

#1	b^{+}	b^{2+}	b^{3+}	b^{4+}	Seq.	y^{+}	y^{2+}	y^{3+}	y^{4+}	#2
1	102.05496	51.53112	34.68984	26.26920	T					26
2	215.13903	108.07315	72.38453	54.54021	L	2839.29530	1420.15129	947.10328	710.57928	25
3	314.20745	157.60736	105.40733	79.30732	V	2726.21123	1363.60925	909.40859	682.30826	24
4	470.30857	235.65792	157.44104	118.33260	R	2627.14281	1314.07504	876.38579	657.54116	23
5	567.36134	284.18431	189.79196	142.59579	P	2471.04169	1236.02448	824.35208	618.51588	22
6	654.39337	327.70032	218.80264	164.35380	S	2373.98892	1187.49810	792.00116	594.25269	21
7	783.43597	392.22162	261.81684	196.61445	E	2286.95689	1143.98208	762.99048	572.49468	20
8	920.49488	460.75108	307.50314	230.87918	H	2157.91429	1079.46078	719.97628	540.23403	19
9	991.53200	496.26964	331.18218	248.63846	A	2020.85538	1010.93133	674.28998	505.96930	18
10	1104.61607	552.81167	368.87687	276.90947	L	1949.81826	975.41277	650.61094	488.21002	17
11	1203.68449	602.34588	401.89968	301.67658	V	1836.73419	918.87073	612.91625	459.93900	16
12	1318.71144	659.85936	440.24200	330.43332	D	1737.66577	869.33652	579.89344	435.17190	15
13	1432.75437	716.88082	478.25631	358.94405	N	1622.63882	811.82305	541.55112	406.41516	14
14	1547.78132	774.39430	516.59862	387.70079	D	1508.59589	754.80158	503.53681	377.90443	13
15	1604.80279	802.90503	535.60578	401.95615	G	1393.56894	697.28811	465.19450	349.14769	12
16	1717.88686	859.44707	573.30047	430.22717	L	1336.54747	668.77737	446.18734	334.89232	11
17	1880.95018	940.97873	627.65491	470.99300	Y	1223.46340	612.23534	408.49265	306.62131	10
18	1995.97713	998.49220	665.99723	499.74974	D	1060.40008	530.70368	354.13821	265.85548	9
19	2093.02990	1047.01859	698.34815	524.01293	P	945.37313	473.19020	315.79589	237.09874	8
20	2208.05685	1104.53206	736.69047	552.76967	D	848.32036	424.66382	283.44497	212.83555	7
21	2368.08750	1184.54739	790.03402	592.77733	C-Carbamidomethyl	733.29341	367.15034	245.10265	184.07881	6
22	2483.11445	1242.06086	828.37633	621.53407	D	573.26275	287.13501	191.75910	144.07115	5
23	2580.16722	1290.58725	860.72726	645.79726	P	458.23580	229.62154	153.41678	115.31441	4
24	2709.20982	1355.10855	903.74146	678.05791	E	361.18303	181.09515	121.06586	91.05122	3
25	2766.23129	1383.61928	922.74861	692.31328	G	232.14043	116.57385	78.05166	58.79057	2
26					R	175.11896	88.06312	59.04450	44.53520	1

TLVRPSEHALVDNDGLYDPDCDPEGR

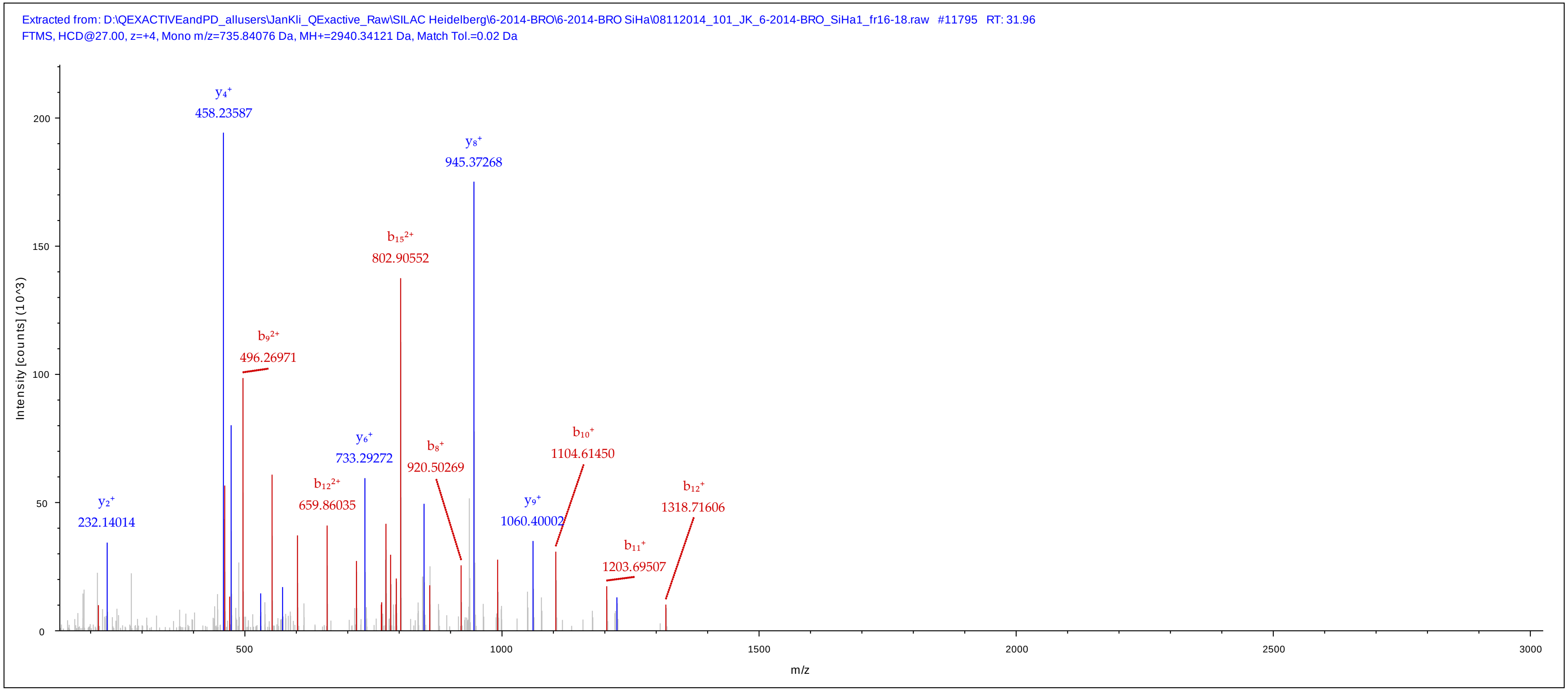

TLVRPSEHALVDNDGLYDPDCDPEGR

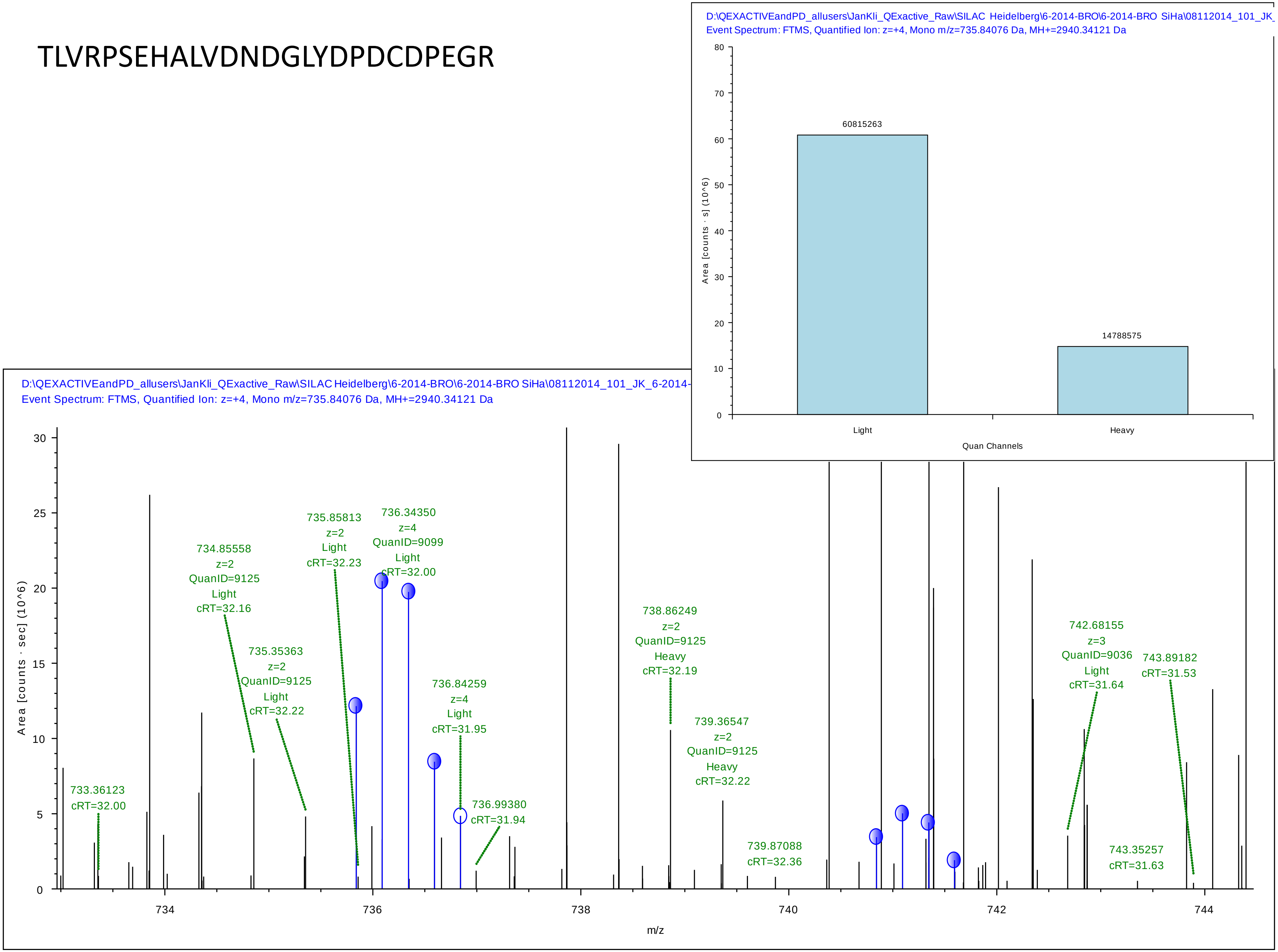

Galectin-7 OS=Homo sapiens GN=LGALS7 PE=1 SV=2 - [LEG7_HUMAN]

Identified peptides:

LVEVGGDVQLDSVR
LDTSEVVFNSK
FHVNLLCGEEQGSDAALHFNPR
GQPFEVLIIASDDGFK
AVVGDAQYHHFR
GLVPPNASR
SSLPEGIRPGTVLR
SSLPEGIR
GPGVPFQR

Coverage | ProteinCard

Galectin-7 OS=Homo sapiens GN=LGALS7 PE=1 SV=2 - [LEG7_HUMAN]

- [ ] Annotate PTMs reported in Uniprot
- [ ] Show only PTMs
- [ ] Include PSMs that are filtered Out

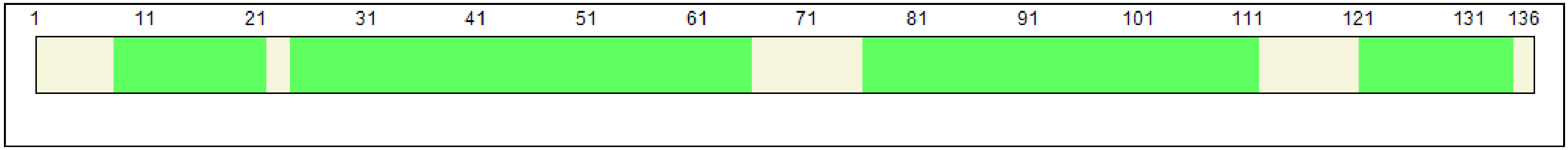

Coverage: 77.94%

Found Modifications:

**C**	Carbamidomethyl (C)
**L**	Label:13C(6)15N(4) (R)
**M**	Label:13C(6)15N(2) (K)

Sequence | Modification List

```
    1    11        21        31        41        51        61        71        81        91        101
  1               L      L           L      C                                M                  L                M
     MSNVPHKSSL PEGIRPGTVL RIRGLVPPNA SRFHVNLLCG EEQGSDAALH FNPRLDTSEV VFNSKEQGSW GREERGPGVP FQRGQPFEVL IIASDDGFKA VVGDAQYHHF

111  L                     L
     RHRLPLARVR LVEVGGDVQL DSVRIF
```

Sequence: LDTSEVVFNSK, K11-Label:13C(6)15N(2) (8.01420 Da)
Charge: +2, Monoisotopic m/z: 623.82233 Da (-1.59 mmu/-2.55 ppm), MH+: 1246.63738 Da, RT: 29.23 min,
Identified with: Mascot (v1.30); IonScore:95, Exp Value:7.9E-008, Ions matched by search engine: 9/100
Fragment match tolerance used for search: 0.02 Da

#1	b^+	b^{2+}	Seq.	y^+	y^{2+}	#2
1	114.09135	57.54931	L			11
2	229.11830	115.06279	D	1133.55649	567.28188	10
3	330.16598	165.58663	T	1018.52954	509.76841	9
4	417.19801	209.10264	S	917.48186	459.24457	8
5	546.24061	273.62394	E	830.44983	415.72855	7
6	645.30903	323.15815	V	701.40723	351.20725	6
7	744.37745	372.69236	V	602.33881	301.67304	5
8	891.44587	446.22657	F	503.27039	252.13883	4
9	1005.48880	503.24804	N	356.20197	178.60462	3
10	1092.52083	546.76405	S	242.15904	121.58316	2
11			K-Label:13C(6)15N(2)	155.12701	78.06714	1

LDTSEVVFNSK

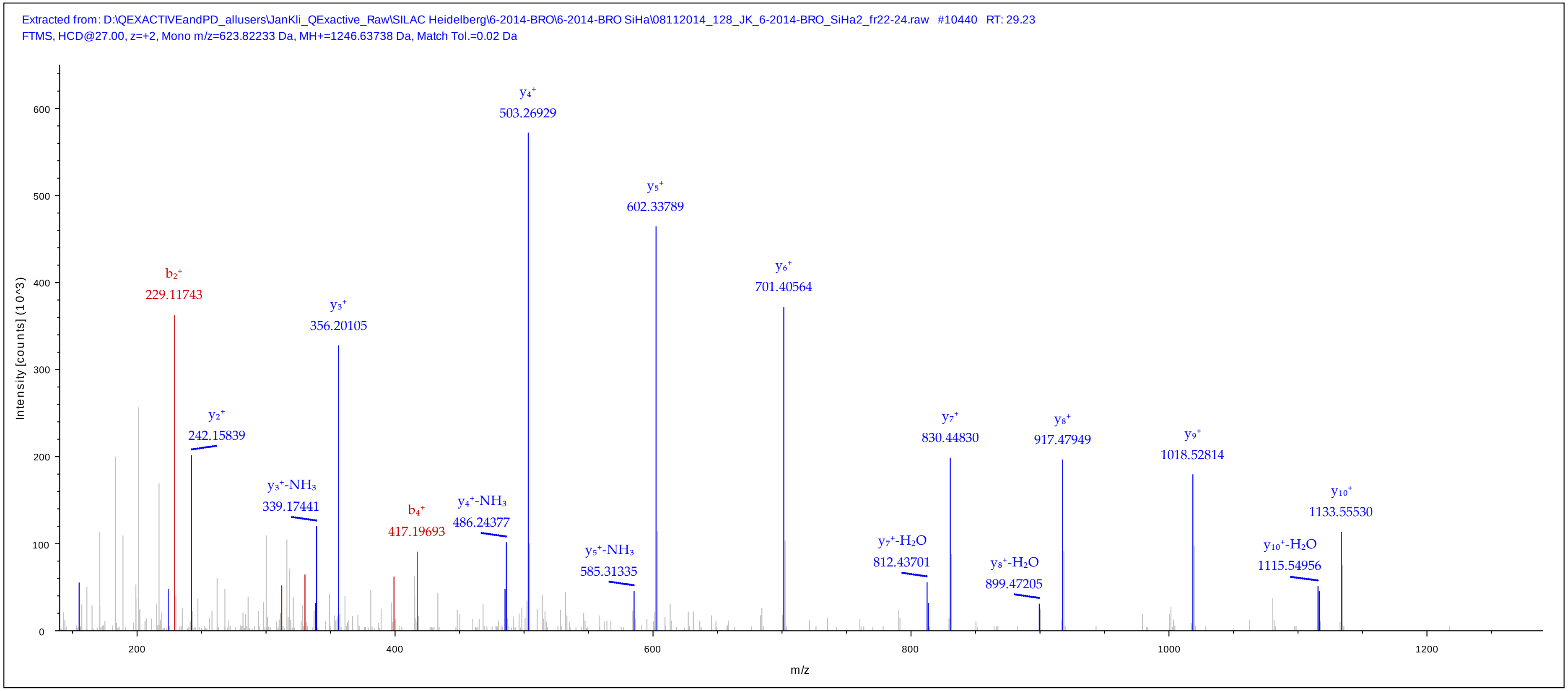

LDTSEVVFNSK

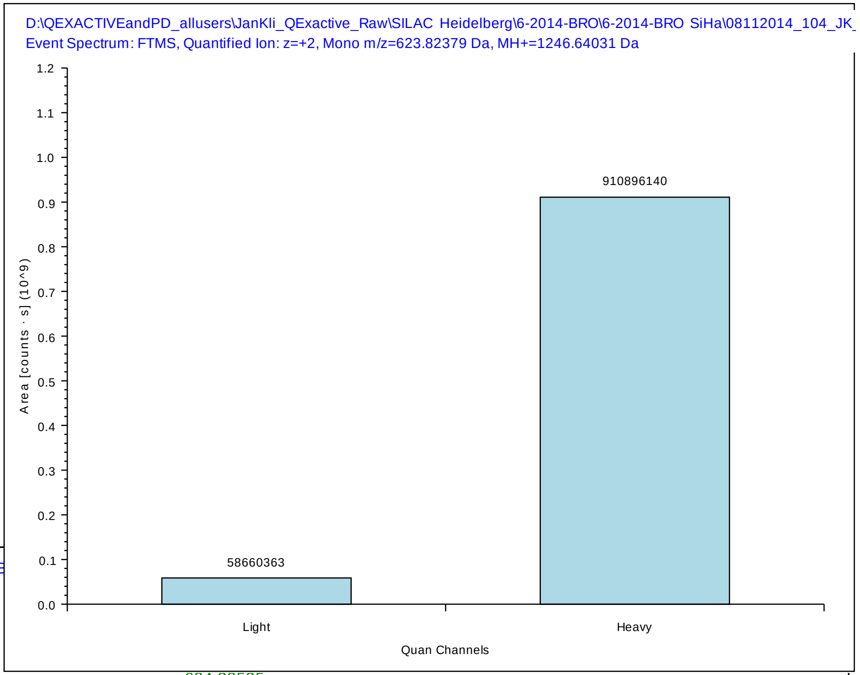

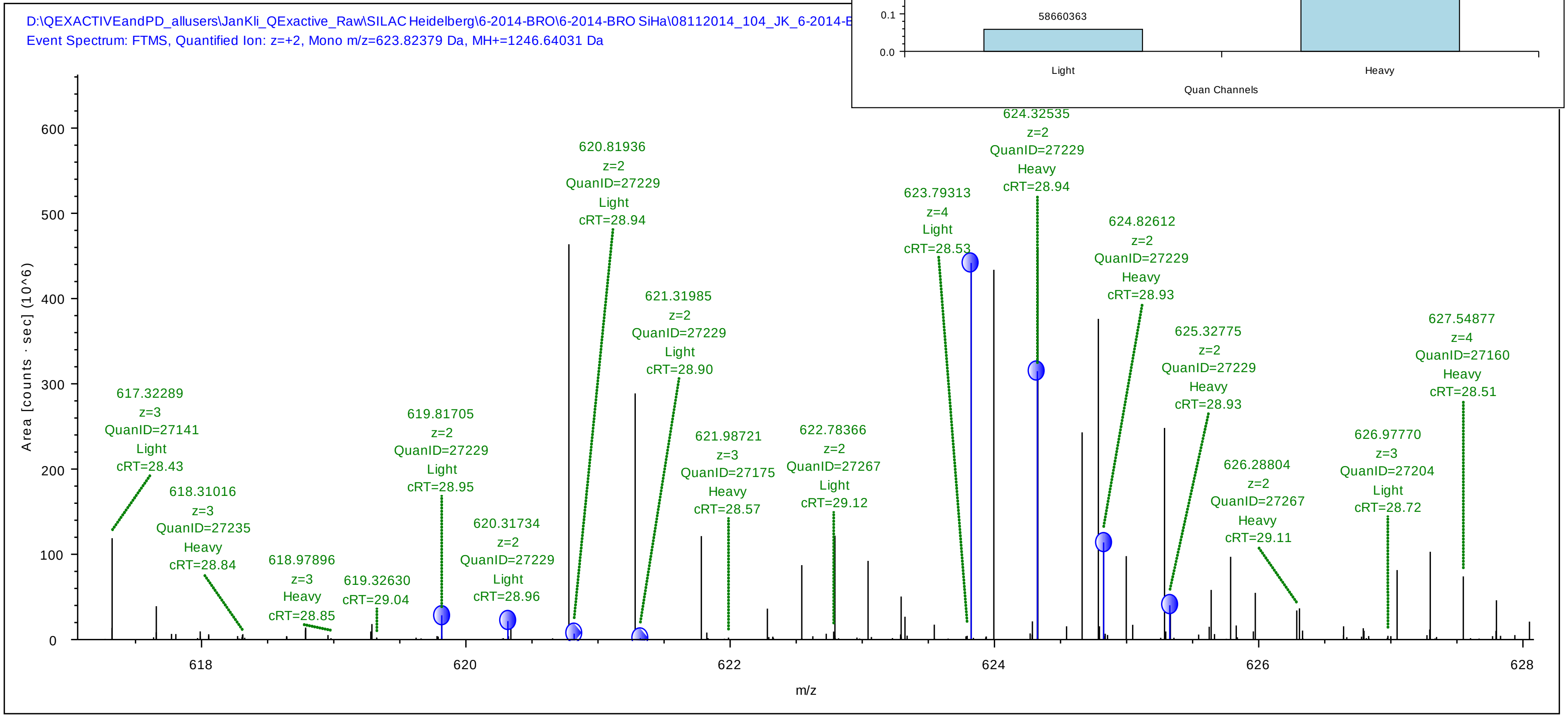

Sequence: GQPFEVLIIASDDGFK, K16-Label:13C(6)15N(2) (8.01420 Da)
Charge: +2, Monoisotopic m/z: 872.45532 Da (-0.53 mmu/-0.6 ppm), MH+: 1743.90337 Da, RT: 51.60 min,
Identified with: Mascot (v1.30); IonScore:81, Exp Value:2.6E-006, Ions matched by search engine: 14/164
Fragment match tolerance used for search: 0.02 Da

#1	b^+	b^{2+}	Seq.	y^+	y^{2+}	#2
1	58.02875	29.51801	G			16
2	186.08733	93.54730	Q	1686.88295	843.94511	15
3	283.14010	142.07369	P	1558.82437	779.91582	14
4	430.20852	215.60790	F	1461.77160	731.38944	13
5	559.25112	280.12920	E	1314.70318	657.85523	12
6	658.31954	329.66341	V	1185.66058	593.33393	11
7	771.40361	386.20544	L	1086.59216	543.79972	10
8	884.48768	442.74748	I	973.50809	487.25768	9
9	997.57175	499.28951	I	860.42402	430.71565	8
10	1068.60887	534.80807	A	747.33995	374.17361	7
11	1155.64090	578.32409	S	676.30283	338.65505	6
12	1270.66785	635.83756	D	589.27080	295.13904	5
13	1385.69480	693.35104	D	474.24385	237.62556	4
14	1442.71627	721.86177	G	359.21690	180.11209	3
15	1589.78469	795.39598	F	302.19543	151.60135	2
16			K-Label:13C(6)15N(2)	155.12701	78.06714	1

GQPFEVLIIASDDGFK

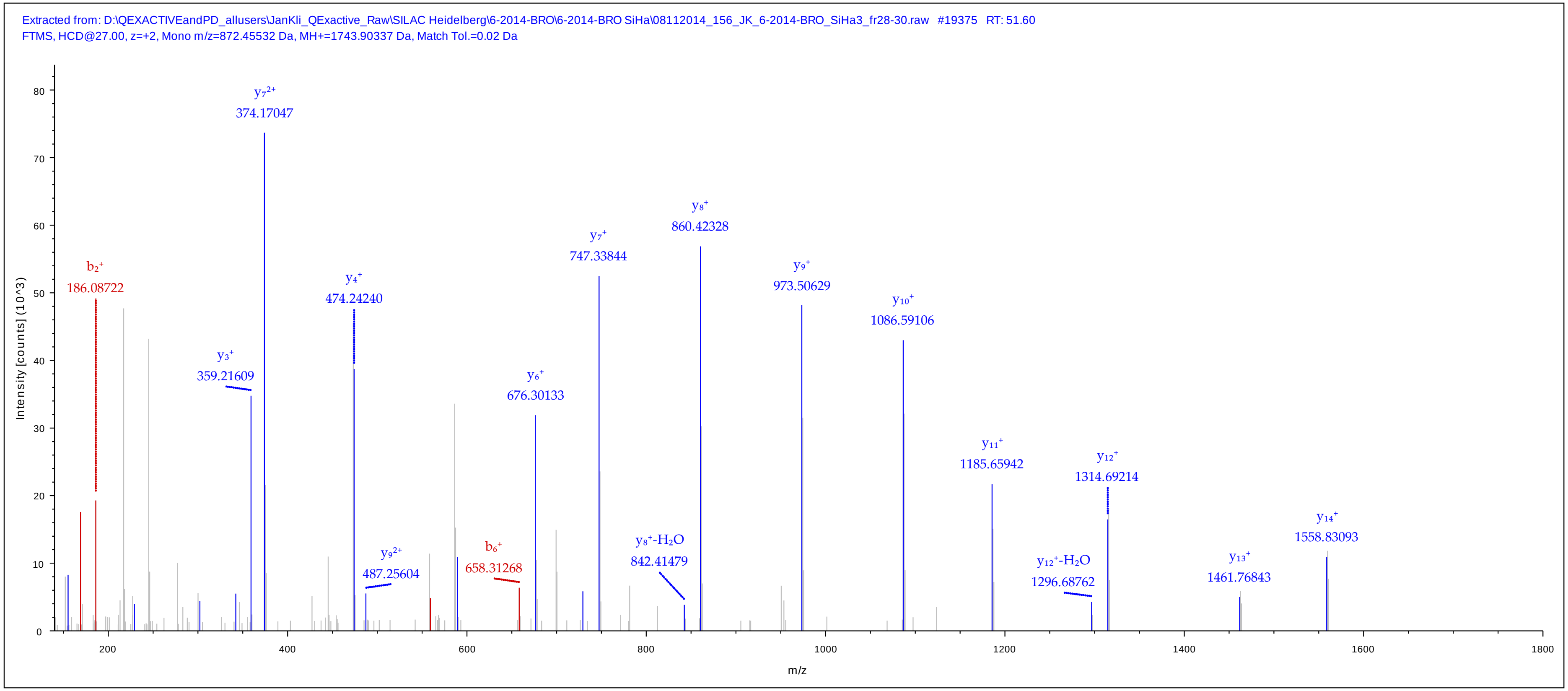

GQPFEVLIIASDDGFK

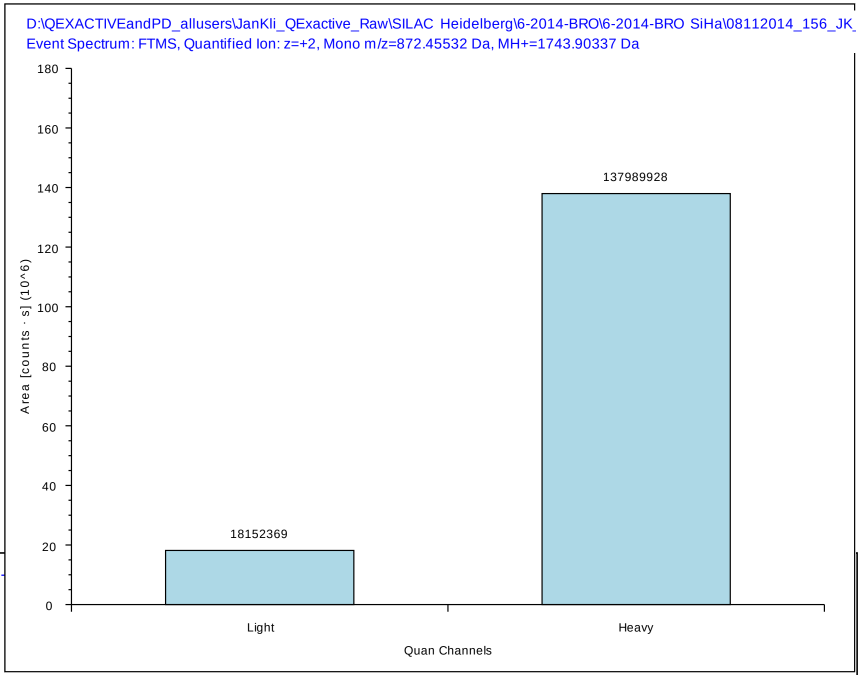

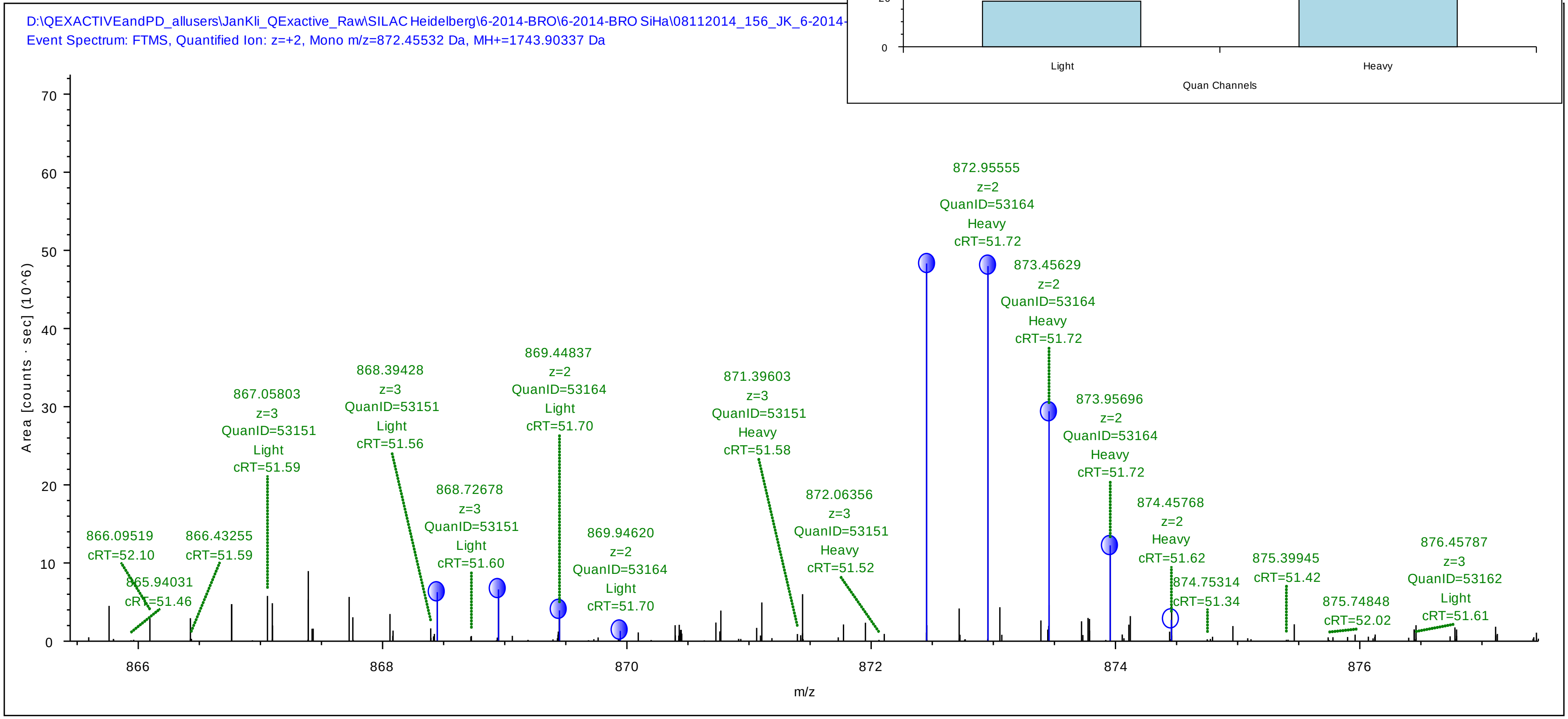

Supplement: Additional file 2: — Supplementary File 1. Ms/Ms identification spectra and SILAC quantification of representative peptides of the common proteins between HeLa Gal-7+ and SiHa Gal-7+ CxCa cells. (PDF 1703 kb) [file 12885_2016_2700_MOESM2_ESM.pdf]
