# Supplementary material for: Systems-level effects of ectopic galectin-7 reconstitution in cervical cancer and its microenvironment
Source: BMC Cancer. 2016 Aug 24;16(1):680. doi: 10.1186/s12885-016-2700-8 (PMC4997669; doi:10.1186/s12885-016-2700-8)
Supplement: Additional file 4: Figure S1. — Patient data of the Scotto and Zhai cohort. (PDF 63 kb) [file 12885_2016_2700_MOESM4_ESM.pdf]

A

Grade	Number of Patients	Average age	HPV status
Normal	24	45 ± 8	Neg
IA	1	40	45
IIA	1	58	16
IIIA	1	Unknown	16
IB	5	50 ± 12	16, 18, 45, Neg
IIB	9	50 ± 14	16, 18, 45, Neg
IIIB	9	43 ± 12	16, 45
IV	1	33	16
Unknown	1	82	16

Cell Line	HPV status
SiHa	HPV16
CaSki	HPV16
HeLa	HPV18
C4-I	HPV18
MS751	HPV18-45
ME-180	HPV65
C-33A	Negative
HT-3	Negative
SW756	HPV18

B

Grade	Number of Patients
Normal	10
HSIL	7
SCC	21

Supplementary Figure 1: (A) Patient data of the Scotto cohort (GEO ID GDS3233) used for microarray analysis: the age, the number of patients, the HPV status (including CxCa cells), and the grading are indicated. (B) Patient data of the Zhai cohort (GEO ID GDS3292) used for microarray analysis: the grading and the number of patients are indicated.
