# Supplementary material for: Systems-level effects of ectopic galectin-7 reconstitution in cervical cancer and its microenvironment
Source: BMC Cancer. 2016 Aug 24;16(1):680. doi: 10.1186/s12885-016-2700-8 (PMC4997669; doi:10.1186/s12885-016-2700-8)
Supplement: Additional file 5: Figure S2. — Transcriptional analysis of the genes identified by the integrative analysis in Gal-7+ CxCa cells. (PDF 343 kb) [file 12885_2016_2700_MOESM5_ESM.pdf]

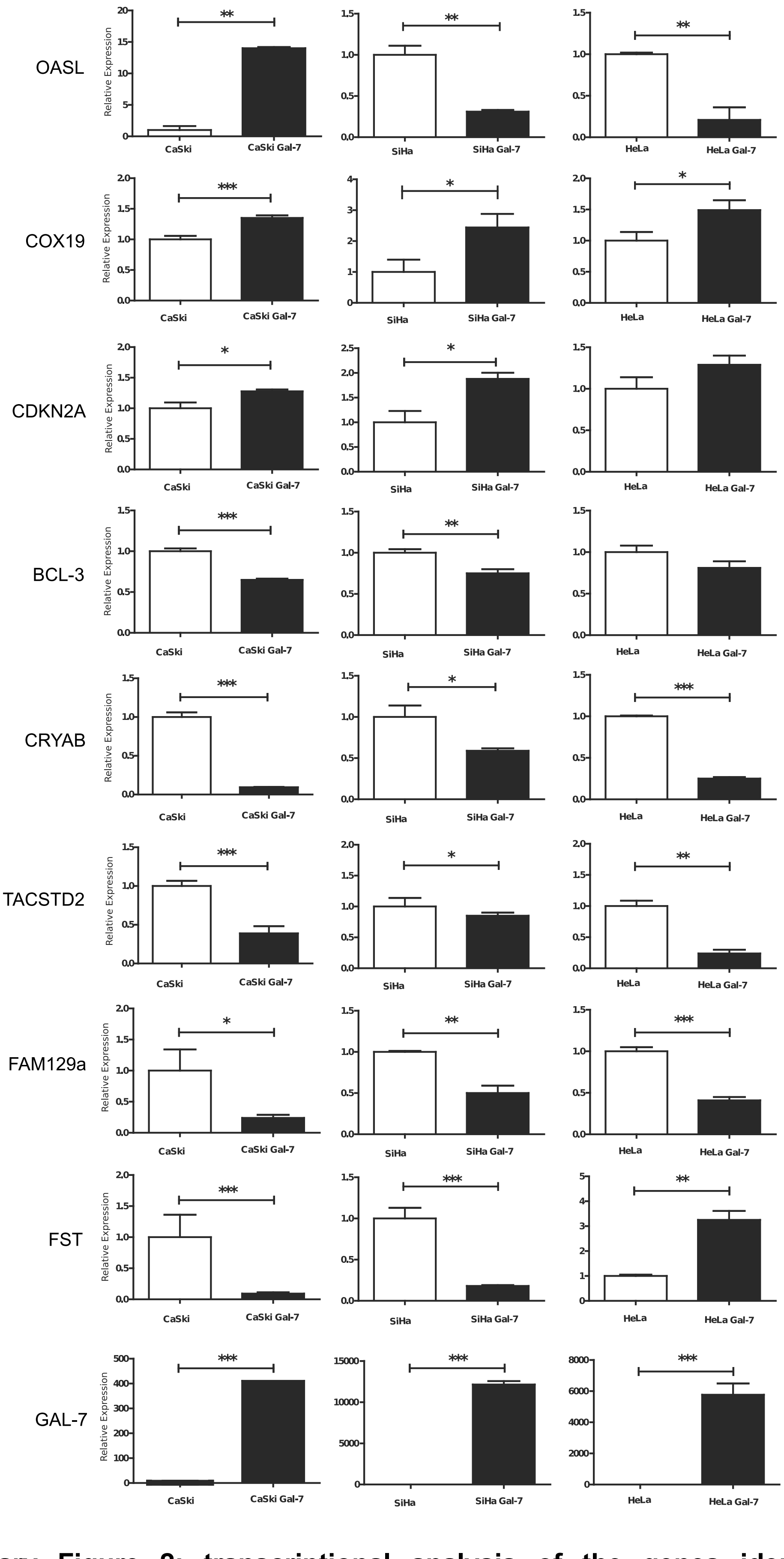

Supplementary Figure 2: transcriptional analysis of the genes identified by the integrative analysis in Gal-7+ CxCa cells. qPCR of OASL, COX19, CDKN2A, BCL-3, CRYAB, TACSTD2, FAM129a, FST and Gal-7 mRNA expression performed in Gal-7-reconstituted and negative CxCa cells (CaSki, SiHa, HeLa). Actin and GAPDH were used as normalization control. (T-Test, with a value of $P < 0.05$. ***= highly significant, **= moderately significant, or *= significant).
