# Supplementary material for: Systems-level effects of ectopic galectin-7 reconstitution in cervical cancer and its microenvironment
Source: BMC Cancer. 2016 Aug 24;16(1):680. doi: 10.1186/s12885-016-2700-8 (PMC4997669; doi:10.1186/s12885-016-2700-8)
Supplement: Additional file 6: Figure S3. — Gene mapping of the mouse microenvironment and immune cells in accordance with The Immunological Genome Project. (PDF 263 kb) [file 12885_2016_2700_MOESM6_ESM.pdf]

A

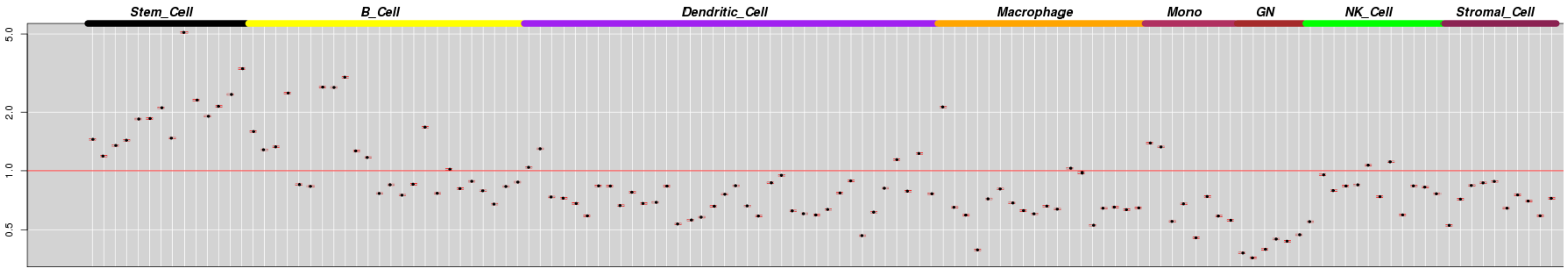

B

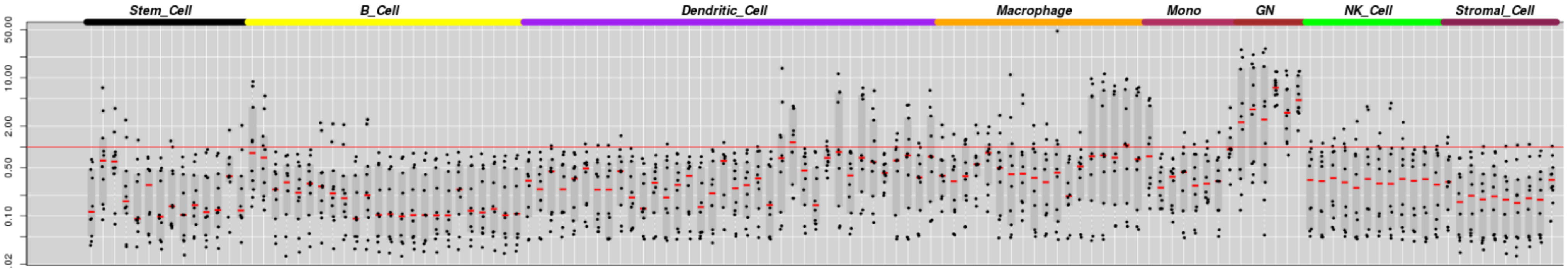

Supplementary Figure 3: Gene mapping of the mouse microenvironment and immune cells. Wplots of mouse genes that were differentially expressed in the microenvironment of Gal-7+ tumors. (A) Wplot representing the expression of Zmynd19 gene. Its expression is highlighted in stem cells and multi-lineage progenitor cells. (B) Wplot representing the expression of Stfa1, Stfa2, Retnlg, S100a8, S100a9, IL1β, CXCL2, CCL3, CCL4, and Arg1genes in granulocytes, dendritic cells and macrophages, moderately enriched in stem cells, β cells and NK cells. The red line shows the basal expression; signals above this threshold can be considered as enriched.
